# Transcriptional Alterations in the Trigeminal Ganglia, Nucleus and Peripheral Blood Mononuclear Cells in a Rat Orofacial Pain Model

**DOI:** 10.3389/fnmol.2018.00219

**Published:** 2018-06-26

**Authors:** Timea Aczél, József Kun, Éva Szőke, Tibor Rauch, Sini Junttila, Attila Gyenesei, Kata Bölcskei, Zsuzsanna Helyes

**Affiliations:** ^1^Department of Pharmacology and Pharmacotherapy, Medical School, University of Pécs, Pécs, Hungary; ^2^Szentágothai Research Centre and Centre for Neuroscience, University of Pécs, Pécs, Hungary; ^3^MTA-PTE Chronic Pain Research Group, Pécs, Hungary; ^4^Section of Molecular Medicine, Rush University Medical Center, Chicago, IL, United States; ^5^Bioinformatics and Scientific Computing, Vienna Biocenter Core Facilities, Vienna, Austria

**Keywords:** orofacial pain, trigeminovascular system, Kisspeptin-1 receptor, *Gpr39*, *Neurod2*, differential gene expression data analysis

## Abstract

Orofacial pain and headache disorders are among the most debilitating pain conditions. While the pathophysiological basis of these disorders may be diverse, it is generally accepted that a common mechanism behind the arising pain is the sensitization of extra- and intracranial trigeminal primary afferents. In the present study we investigated gene expression changes in the trigeminal ganglia (TRG), trigeminal nucleus caudalis (TNC) and peripheral blood mononuclear cells (PBMC) evoked by Complete Freund's Adjuvant (CFA)-induced orofacial inflammation in rats, as a model of trigeminal sensitization. Microarray analysis revealed 512 differentially expressed genes between the ipsi- and contralateral TRG samples 7 days after CFA injection. Time-dependent expression changes of G-protein coupled receptor 39 (*Gpr39*), kisspeptin-1 receptor (*Kiss1r*), kisspeptin (*Kiss1*), as well as synaptic plasticity-associated Lkaaear1 (*Lkr*) and Neurod2 mRNA were described on the basis of qPCR results. The greatest alterations were observed on day 3 ipsilaterally, when orofacial mechanical allodynia reached its maximum. This corresponded well with patterns of neuronal (*Fosb*), microglia (*Iba1*), and astrocyte (*Gfap*) activation markers in both TRG and TNC, and interestingly also in PBMCs. This is the first description of up- and downregulated genes both in primary and secondary sensory neurones of the trigeminovascular system that might play important roles in neuroinflammatory activation mechanisms. We are the first to show transcriptomic alterations in the PBMCs that are similar to the neuronal changes. These results open new perspectives and initiate further investigations in the research of trigeminal pain disorders.

## Introduction

Orofacial pain and headache disorders are among the most debilitating pain conditions. While the pathophysiological basis of these disorders may be diverse, it is generally accepted that a common mechanism behind the arising pain is the sensitization of extra- and intracranial trigeminal primary afferents. The trigeminal nerve provides most of the sensory innervation to the face and oral cavity as well as the meninges where the nociceptive primary afferents are closely associated with the vasculature. The cell bodies of these neurons are located in the trigeminal ganglion (TRG) and their central projections terminate in the trigeminal nucleus caudalis (TNC). It has been described that there is convergence of extra- and intracranial primary afferents in the TNC (Burstein et al., 1998). Sensitization of these secondary nociceptive neurons might be responsible for the phenomenon of the facial allodynia developing in primary headaches (Burstein et al., 2000). A similar mechanism could induce the headache associated with disorders of extracranial structures. Inflammation of the temporal artery, temporomandibular joint, sinuses or orbit can induce headache which could have the same characteristics as the primary disorders. Co-morbidity of migraine and temporomandibular disorders has also been reported (Romero-Reyes and Uyanik, 2014).

Inflammatory pain models adapted to the orofacial area induce trigeminal sensitization and can constitute a possible way to understand the mechanisms of pain associated with orofacial disorders and headaches (Krzyzanowska et al., 2011; Krzyzanowska and Avendaño, 2012; Romero-Reyes et al., 2013). A commonly used model of peripheral inflammation in animals is injection of Complete Freund's Adjuvant (CFA) (Ren and Dubner, 1999; Takeda et al., 2007; Krzyzanowska and Avendaño, 2012; Gregory et al., 2013). Orofacial inflammation induces mechanical hyperalgesia/allodynia on the face by activation/sensitization of trigeminal primary and secondary sensory neurons (Iwata et al., 2017).

Since the mechanisms of trigeminal sensitization are not known, global transcriptomic analysis allows an unbiased approach to reveal key pathways responsible for the pathophysiological changes (Perrino et al., 2017). Gene expression changes in the trigeminal ganglion (TRG) had been assessed by microarray analysis after CFA injection in whisker pad (Okumura et al., 2010) or masseter muscle (Chung et al., 2016). However, no study has evaluated TRG gene expression changes in parallel with the central gene expression variances in the trigeminal nucleus caudalis (TNC) and correlate it with the time course and extent of facial allodynia. This comprehensive approach might facilitate the identification of differentially regulated genes with a relevant role in the cascade of events resulting in the sensitization of primary and secondary trigeminal neurons. Moreover, there is growing evidence that transcriptome changes in the central nervous system could be reflected in peripheral blood cells. Investigation of gene expression changes in migraine patients identified differential expression of major genes from the peripheral blood (Gardiner et al., 1998; Hershey et al., 2004, 2012; Du et al., 2006; Plummer et al., 2011; Gerring et al., 2017). Gene transcription changes of PBMCs have not been analysed in animal models of trigeminal sensitization, although it could provide a good opportunity to compare with human data.

The aim of the present study was to follow the temporal changes of facial mechanonociceptive thresholds and gene expression in TRG, TNC neurones and PBMCs after CFA inflammation using microarray and qPCR analyses in order to get a better insight into the mechanisms of trigeminal pain disorders.

## Materials and methods

### Animals

Twenty male Wistar rats (Toxicoop Zrt., Hungary) weighing between 200–300 g were used. Animals were kept under standard light-dark cycle (12-h light/dark cycle) and temperature (24–25°C) conditions, food and water were provided *ad libitum*, in the local animal house of the Pécs University Department of Pharmacology and Pharmacotherapy. In order to minimise stress, all rats were habituated to handling and the light restraint used for the facial von Frey test for 3 days prior to the start of the experiments.

The study was carried out in accordance with the Ethical Codex of Animal Experiments of the University of Pécs and the 1998/XXVIII Act of the Hungarian Parliament on Animal Protection and Consideration Decree of Scientific Procedures of Animal Experiments (243/1988). The protocol was approved by the local Ethics Committee on Animal Research of University of Pécs (license No.: BA02/2000-9/2011).

### CFA injection

Orofacial inflammation was induced by unilateral s.c. injection of 50 μl complete Freund's adjuvant (CFA; Sigma-Aldrich, Saint Louis, USA; killed mycobacteria suspended in paraffin oil; 1 mg/ml) into the whisker pad of male rats, while under ketamine (72 mg/kg) and xylazine (8 mg/kg) anaesthesia. In the second series of experiments, a control group received the same volume of saline injection.

### Microarray

Orofacial inflammation-associated gene expression was analysed using Agilent microarray platforms. Rat TRG tissue samples were collected from animals 7 days after receiving s.c. CFA injection (*n* = 8). Animals were anaesthetized with thiopental (100 mg/kg i.p.) and sacrificed by exsanguination. TRGs were excised and snap-frozen in liquid nitrogen. Contralateral sides of CFA-injected rats served as controls. Total RNA were isolated from snap-frozen samples using RNeasy Mini Kit (Qiagen, Carlsbad, CA) and high-quality samples (RIN > 8.0) were used for subsequent expression analyses. Sample labelling, array hybridization and primary data analysis was performed by ArrayStar Inc. (Rockville, MD, USA). Briefly, total RNA samples were amplified and labelled with Cy3-dCTP. Labeled amplicons were purified, fragmented and hybridized to rat LncRNA Array v1.0 (4 × 44 K, Arraystar Inc.) slides. One-color microarray-based gene expression analysis was used. After hybridization slides were washed, fixed and scanned. Gene expression data files were deposited to NCBI's Gene Expression Omnibus (Edgar et al., 2002) and are accessible through GEO Series accession number GSE111160.

### Orofacial pain sensitivity tested with von frey filaments

In a second experiment, mechanical pain thresholds of the orofacial region were determined with a series of von Frey filaments. Tests were performed on days 0 (control day) before and 1, 3, 7 after CFA (*n* = 9)/saline (*n* = 3) injection. Animals were lightly restrained using a soft cotton glove in order to allow an easier habituation, then a set of calibrated nylon monofilaments (Stoelting, Wood Dale, Illinois, U.S.A) was used with increasing strengths (0.8–12 g) to measure facial mechanosensitivity. Filaments were applied in ascending order, starting from the 5.2 g filament during control measurements and the 0.8 g filament after CFA treatment. The mechanonociceptive threshold was defined as the lowest force evoking at least two withdrawal responses (face stroking with the forepaw or head shaking) out of five stimulations.

### Experimental setup of the second experiment

At each time point (1, 3, and 7 days) animals (*n* = 3) were anaesthetized with thiopental and blood was collected by cardiac puncture. Tissue samples (TRG, TNC) were quickly frozen in liquid nitrogen and stored at −80°C until RNA extraction and real-time PCR processing.

### Isolation of peripheral blood mononuclear cells

Mononuclear cells were purified from fresh peripheral blood according to Ficoll-PaquePREMIUM (Cat. No. 17-5446-02, GE Healthcare, Budapest, Hungary) manufacturer's instructions. Fresh anticoagulant-treated blood and an equal volume of balanced salt solution (final volume of 8 ml) were transferred to 15 ml sterile centrifuge tubes. The mixture was carefully overlaid on 5 ml Ficoll-PaquePREMIUM and centrifuged 40 min at 2,100 RPM, 20°C. The mononuclear layer was transferred into a new 15 ml centrifuge tube, suspended with approximately 6 ml of salt solution and centrifuged 15 min at 2300 RPM, 20°C. The supernatant was removed and the pellet was resuspended in another 6 ml of salt solution, followed by another centrifugation (10 min, 2300 RPM, 20°C). After the removal of the supernatant the cells were resuspended with 1 ml of TRI Reagent (Molecular Research Center, Inc., Cincinnati, OH, USA) and transferred to Eppendorf and stored at −80°C until use.

### Quantitative real-time RT-PCR (qRT-PCR)

Purification of total RNA was carried out according to the TRI Reagent manufacturer's (Molecular Research Center, Inc., Cincinnati, OH, USA) protocol up to the step of acquiring the aqueous phase. Briefly, tissue samples were homogenized in 1 ml of TRI Reagent, and then, 200 μl of bromo-chloro-propane (Sigma-Aldrich, Saint Louis, USA) was added. RNA was purified from the aqueous phase using the Direct-zol RNA MiniPrep kit (Cat. No. R2052; Zymo Research, Irvine, CA, USA) according to the manufacturer's protocol. Briefly, 400 μl of the aqueous phase was mixed with 400 μl absolute ethanol, the mixture was loaded onto the column, washed, and the RNA was eluted in 50 μl of RNase-free water. The quantity and purity of the extracted RNA were assessed on Nanodrop ND-1000 Spectrophotometer V3.5 (Nano-Drop Technologies, Inc., Wilmington, DE, USA). 200 ng of PBMCs/TRG and 250 ng of TNC total RNA was reverse transcribed using Maxima First Strand cDNA Synthesis Kit (Cat. No. K1642, ThermoScientific, Santa Clara, CA, USA) according to the manufacturer's instructions. qRT-PCR was performed on a Stratagene Mx3000P qPCR System (Agilent Technologies, Santa Clara, USA). PCR amplification was performed using SensiFast SYBR Lo-ROX Kit (Cat. No. BIO-94020). Transcripts of the reference genes glyceraldehyde 3-phosphate dehydrogenase (*Gapdh*), hypoxanthine phosphoribosyltransferase 1 (*Hprt1*), beta-2-microglobulin (β*2m*) and Peptidyl-prolyl cis-trans isomerase (*Ppia*) were detected in all samples. *Ppia* and *Hprt1* for PBMCs and β*2m, Hprt1* for TRG and TNC samples were eventually chosen as internal controls, the geometric mean of their Cq values was calculated. Primers of similar efficiencies were used and 2^−ΔΔCq^ fold change values were calculated. Sequences of primers used for qRT-PCR are given in Supplementary Table 1.

### Statistical analysis

The raw microarray data were analysed using R and Bioconductor (Gentleman et al., 2004; R Development Core Team, 2008) The data were quantile normalised to reduce technical noise with Limma package (Ritchie et al., 2015). The statistical testing for differential expression was also performed using Limma, which applies linear modeling with a modified *t*-test to calculate the *p*-values and fold change values. One-way analysis of variance (ANOVA) followed by Tukeys' multiple comparison tests on RT-PCR data and in case of mechanical pain threshold detection two-way ANOVA with repeated measures followed by Bonferroni's post-test for time-matching samples were performed using GraphPad Prism software (GraphPad Software, Inc., La Jolla, CA, USA). Probability values *p* ≤ 0.05 were accepted as significant. Results are presented as the mean ± standard error of the mean (SEM). Log2 mRNA fold change data measured by qPCR were further analysed by hierarchical cluster analysis (1-Pearson correlation and average linkage method) and then visualised by heat map using the free web tool Morpheus (Morpheus)^1^.

### Functional classification of differentially regulated genes

The functional enrichment analyses against Gene Ontology (GO) (Ashburner et al., 2000; The Gene Ontology Consortium, 2017), KEGG (Kanehisa and Goto, 2000) and Reactome (Fabregat et al., 2018) databases were performed using the topGO (Alexa and Rahnenführer, 2016) and gage (Luo et al., 2009) packages in R.

## Result

### Microarray analysis

**Figure 1:**
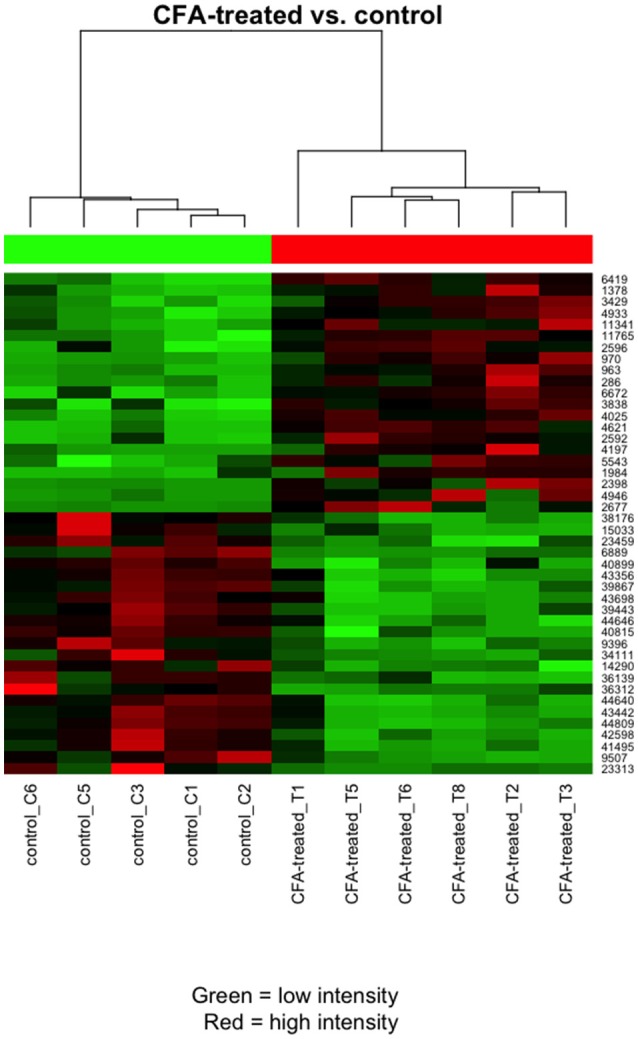
Heat map clustering of the most differentially expressed transcripts for the comparison between CFA-treated and contralateral side TRG samples. Pearson's metrics has been used in hierarchical clustering of the samples and filtered features. The clustering is based on the general expression measurement similarity. Red colour means high expression and green low expression. Each row represents one differentially expressed (DE) feature (microarray feature identifiers) and each column represents one sample.

The microarray data analysis identified 512 differentially expressed (319 up- and 191 downregulated) transcripts between the control (contralateral) and 7-day CFA (ipsilateral) samples from TRG at a statistically significant level (p ≤ 0.05) and with fold change |FC|> 2 (Supplementary Table 2; Supplementary Figures 1, 2). All but 15 of these have absolute fold change values below 4. Original data files have been uploaded to the NCBI GEO Database. The top 25 up- and downregulated genes are included in Table 1. The most upregulated (5.20 fold) transcript was found to be a lncRNA (MRAK049104) with unknown function. The most downregulated transcript (−9.20 fold), Neurod2, is involved in neuronal differentiation. Figure 1 shows the 44 differentially expressed genes at a significance level *p* ≤ 0.001 and |FC|> 2, including a number of olfactory, taste and pheromone receptors, as well as the chemokine receptor (*Ccr7*) and the estrogen receptor 1 (Esr1) genes as well as long non-coding RNAs.

**Table 1:** The top 25 up- and downregulated genes.

**ID**	**FC**	***P*.Value**	**SystematicName**	**GeneSymbol**	**Description**	**EnsemblID**	**EntrezGene**
948	**5.20**	0.0381	MRAK049104	**NA**	**lncRNA (chromosome 1)**	NA	NA
11131	**4.55**	0.0353	TC598318	**NA**	**NA**	NA	NA
9725	**4.35**	0.0214	NM_199489.3	**Ccr7**	**C-C motif chemokine receptor 7**	ENSRNOG00000010665	287673
5534	**4.34**	0.0076	NM_001011951.1	**Sf3b4**	**splicing factor 3b, subunit 4**	ENSRNOG00000021181	295270
12278	**4.29**	0.0010	NM_001037518.1	**Defb23**	**defensin beta 23**	ENSRNOG00000023477	641621
519	**4.28**	0.0218	NM_001000099.1	**Olr1640**	**olfactory receptor 1640**	ENSRNOG00000048857	290049
13893	**4.13**	0.0236	NM_001106821.1	**Atm**	**ATM serine/threonine kinase**	ENSRNOG00000029773	300711
40839	**4.08**	0.0135	NM_134399.2	**Mk1**	**Mk1 protein**	ENSRNOG00000019657	171436
6132	**4.01**	0.0172	NM_001106551.1	**Lkaaear1**	**LKAAEAR motif containing 1**	ENSRNOG00000024815	296483
10158	**3.83**	0.0477	NM_001013956.1	**RGD1309049**	**similar to RIKEN cDNA 4933415F23**	ENSRNOG00000014123	301306
10494	**3.80**	0.0138	NM_001013147.1	**Axl**	**Axl receptor tyrosine kinase**	ENSRNOG00000020716	308444
2571	**3.78**	0.0404	NM_019128.4	**Ina**	**internexin neuronal intermediate filament protein, alpha**	ENSRNOG00000020248	24503
12538	**3.73**	0.0232	NM_198133.2	**Uts2b**	**urotensin 2B**	ENSRNOG00000038512	378939
3346	**3.72**	0.0156	NM_001047878.1	**F5**	**coagulation factor V**	ENSRNOG00000057855	NA
6931	**3.71**	0.0198	uc.339+	**NA**	**lncRNA (chromosome 7)**	NA	NA
5544	**3.70**	0.0316	NM_001001034.1	**Olr199**	**olfactory receptor 199**	ENSRNOG00000029755	405920
9136	**3.69**	0.0250	NM_012735.1	**Hk2**	**hexokinase 2**	ENSRNOG00000006116	25059
4102	**3.67**	0.0212	NM_001001010.1	**Olr283**	**olfactory receptor 283**	ENSRNOG00000030782	405384
3492	**3.67**	0.0159	uc.470+	**NA**	**lncRNA (chromosome X)**	NA	NA
1789	**3.66**	0.0240	XR_005913	**NA**	**lncRNA (chromosome 16)**	NA	NA
4451	**3.64**	0.0064	NM_173305.1	**Hsd17b6**	**hydroxysteroid (17-beta) dehydrogenase 6**	ENSRNOG00000002597	286964
11677	**3.63**	0.0013	NM_001000387.1	**Olr416**	**olfactory receptor 416**	ENSRNOG00000029069	296678
6676	**3.62**	0.0322	NM_001107582.2	**Pdcd1lg2**	**programmed cell death 1 ligand 2**	ENSRNOG00000016136	309304
9724	**3.61**	0.0247	NM_153466.1	**Gzmf**	**granzyme F**	ENSRNOG00000028810	266704
5556	**3.60**	0.0103	NM_001099514.1	**Vom2r48**	**vomeronasal 2 receptor, 48**	ENSRNOG00000028538	686145
39867	−**3.50**	0.0007	NM_001047931.1	**LOC498460**	**LRRGT00055**	ENSRNOG00000028821	498460
41314	−**3.62**	0.0313	NM_001080939.1	**Tas2r109**	**taste receptor, type 2, member 109**	ENSRNOG00000032724	690572
38176	−**3.63**	0.0005	NM_001164826.1	**RT1-Db2**	**RT1 class II, locus Db2**	ENSRNOG00000030431	24981
44381	−**3.64**	0.0049	NM_001130497.1	**Pnpla5**	**patatin-like phospholipase domain containing 5**	ENSRNOG00000022296	300108
35607	−**3.67**	0.0016	MRAK078136	**NA**	**lncRNA (chromosome 1)**	NA	NA
16902	−**3.73**	0.0186	uc.163+	**NA**	**NA**	NA	NA
41319	−**3.76**	0.0102	NM_001012084.1	**Adh6**	**alcohol dehidrogenase 6**	NA	NA
37492	−**3.78**	0.0025	NM_001003979.1	**Tmprss11c**	**transmembrane protease, serine 11C**	ENSRNOG00000033910	408213
36560	−**3.87**	0.0023	NM_001000338.1	**Olr619**	**olfactory receptor 619**	ENSRNOG00000021473	295843
43356	−**4.15**	0.0009	NM_022673.2	**Mecp2**	**methyl CpG binding protein 2**	ENSRNOG00000056659	29386
42598	−**4.17**	0.0001	NM_001017480.1	**Hoxb7**	**homeo box B7**	ENSRNOG00000007611	497985
38934	−**4.62**	0.0036	NM_052808.1	**Bpifa2**	**BPI fold containing family A, member 2**	ENSRNOG00000013540	50585
38765	−**4.64**	0.0056	NM_001000575.1	**Olr741**	**olfactory receptor 741**	ENSRNOG00000053815	366120
37233	−**4.69**	0.0053	NM_012689.1	**Esr1**	**estrogen receptor 1**	ENSRNOG00000019358	24890
43878	−**4.71**	0.0017	NM_001000307.1	**Olr485**	**olfactory receptor 485**	ENSRNOG00000009747	295751
38741	−**4.80**	0.0043	NM_001001355.1	**Olr905**	**olfactory receptor 905**	ENSRNOG00000057325	288875
44809	−**4.86**	0.0006	NM_001109459.1	**LOC685171**	**similar to protein disulfide isomerase-associated 6**	ENSRNOG00000058543	685171
40038	−**4.88**	0.0012	uc.400−	**NA**	**lncRNA (chromosome 19)**	NA	NA
43786	−**5.14**	0.0023	NM_001109285.1	**C2cd4c**	**C2 calcium-dependent domain containing 4C**	ENSRNOG00000008026	500798
43442	−**5.40**	0.0004	uc.225+	**NA**	**lncRNA (chromosome 4)**	NA	NA
40720	−**5.65**	0.0041	NM_001000692.1	**Olr25**	**olfactory receptor 25**	ENSRNOG00000046609	404897
41996	−**6.14**	0.0014	uc.47−	**NA**	**lncRNA (chromosome 6)**	NA	NA
43609	−**6.60**	0.0018	NM_001000394.1	**Olr428**	**olfactory receptor 428**	ENSRNOG00000030460	296689
36293	−**8.03**	0.0021	XR_008902	**NA**	**lncRNA (chromosome 19)**	NA	NA
44472	−**9.20**	0.0012	NM_019326.1	**Neurod2**	**neuronal differentiation 2**	ENSRNOG00000028417	NA

*ID, microarray feature identifier; FC, expression ratio (fold-change) between CFA-treated and contralateral sample groups*.

### Gene ontology

Gene set enrichment analysis was performed on the microarray data to find common features of genes. The most differentially expressed genes (|FC|>2, *p* ≤ 0.001) between the control (contralateral) and 7-day CFA (ipsilateral) samples from TRG were functionally annotated based on gene ontology (GO), KEGG Pathway and Reactome terms to gain an overview of the affected biological processes and pathways (Table 2). The identified enriched GO terms include steroid and carbohydrate metabolism, sensory perception and olfactory transduction.

**Table 2:** Results of gene set enrichment analysis of a subset of genes differentially expressed between the control (contralateral) and 7-day CFA (ipsilateral) samples from TRG as detected by microarray.

	**Term**	**Annotated**	**Significant**	**Expected**	***P*-Value**
**GO.ID**	**Biological process**				
GO:0008202	Steroid metabolic process	223	3	0.3	0.0031
GO:0005975	Carbohydrate metabolic process	400	3	0.54	0.0155
GO:0007600	Sensory perception	1,453	5	1.97	0.0380
GO:0050911	Detection of chemical stimulus involved in sensory perception of smell	1,059	4	1.43	0.0487
**CELLULAR COMPONENT**					
GO:0005576	Extracellular region	3,051	6	3.95	0.181
GO:0005615	Extracellular space	2,639	5	3.41	0.243
GO:0071944	Cell periphery	4,053	7	5.24	0.246
GO:0044421	Extracellular region part	2,763	5	3.57	0.276
GO:0016021	Integral component of membrane	4,420	7	5.72	0.334
GO:0031224	Intrinsic component of membrane	4,505	7	5.83	0.356
GO:0044425	Membrane part	5,350	8	6.92	0.381
GO:0005886	Plasma membrane	3,959	6	5.12	0.407
GO:0044464	Cell part	11,048	15	14.29	0.476
GO:0005623	Cell	11,071	15	14.32	0.486
**MOLECULAR FUNCTION**					
GO:0004984	Olfactory receptor activity	1,059	4	1.21	0.0269
GO:0099600	Transmembrane receptor activity	1,749	5	2	0.0374
**KEGG.ID**	**KEGG pathway term**				
604	Glycosphingolipid biosynthesis - ganglio series	12	1	0.012067578	0.012014279
603	Glycosphingolipid biosynthesis - globo series	13	1	0.01307321	0.013010232
533	Glycosaminoglycan biosynthesis - keratan sulfate	14	1	0.014078842	0.014005382
512	Mucin type O-Glycan biosynthesis	20	1	0.020112631	0.019959439
500	Starch and sucrose metabolism	28	1	0.028157683	0.027853402
4740	Olfactory transduction	842	3	0.846741754	0.036986751
**Reactome.ID**	**Downregulated reactome term**	**GeneRatio**	**BgRatio**	***P*****-Value**	
R-RNO-8957275	Post-translational protein phosphorylation	2/5	74/5483	0.001750444	
R-RNO-381426	Regulation of Insulin-like Growth Factor (IGF) transport and uptake by Insulin-like Growth Factor Binding Proteins (IGFBPs)	2/5	82/5483	0.002145931	

*Enrichment analysis for the differentially expressed filtered gene lists test whether the genes within a certain KEGG or Reactome pathway or GO term are statistically over-represented in a given comparison*.

### Mechanonociceptive threshold

**Figure 2:**
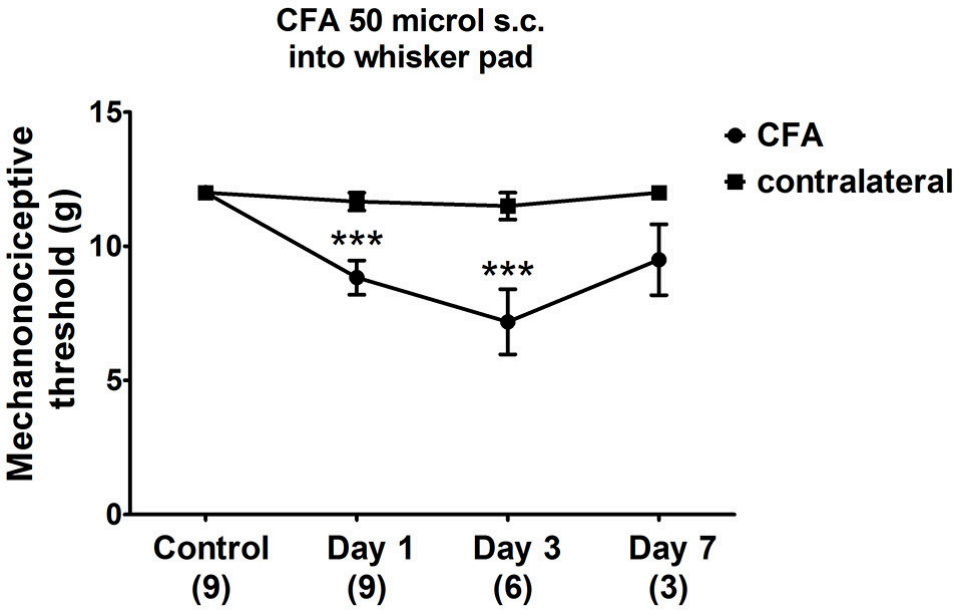
Changes in mechanical threshold in response to von Frey filaments before and 1, 3, 7 days after CFA (50 μl s.c. complete Freund's adjuvant) inflammation. Orofacial thresholds in both ipsilateral and contralateral sides were measured. Data are means ± S.E.M. (*n* = 9 at control and day 1; *n* = 6 at day 3; *n* = 3 at day 7). Asterisks denote statistically significant differences between contralateral (CT) and ipsilateral (CFA) sides (***p ≤ 0.001) as analysed by two-way ANOVA followed by Bonferroni's post-test.

The facial mechanonociceptive threshold of CFA-injected rats was significantly decreased compared to the contralateral side starting from day 1 after injection. The allodynia reached its maximum on day 3 (*p* ≤ 0.001), as the threshold change was lower on day 7 (Figure 2). No significant changes in the contralateral threshold were observed in the whisker pad area.

### RT-PCR analysis

#### Validation of differentially expressed mRNAs in TRG by real-time RT-PCR

**Figure 3:**
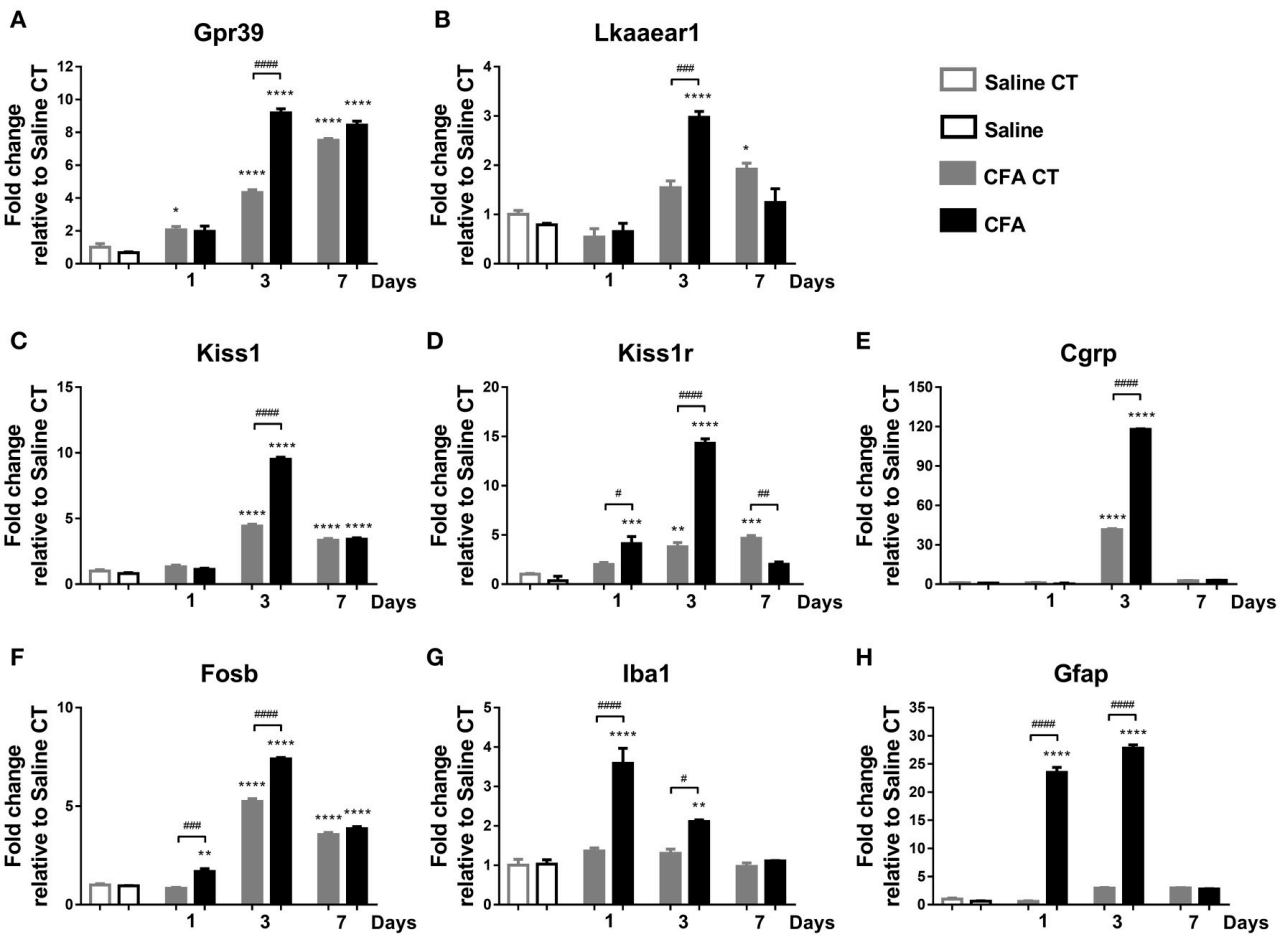
Time course of normalized fold changes in the trigeminal ganglia of Gpr39 **(A)**, Lkaaear1 **(B)**, Kiss1 **(C)**, Kiss1r **(D)**, Cgrp **(E)**, Fosb **(F)**, Iba1 **(G)**, and Gfap **(H)** mRNA expression one, 3 and 7 days after CFA injection. The mRNA levels were normalised to β2m and Hprt1, as detailed in materials and methods. Data are means ± S.E.M. (*n* = 3 at each time point). Asterisks denote statistically significant differences between CT Saline and CT/CFA groups (*p ≤ 0.05, **p ≤ 0.01, ***p ≤ 0.001, ****p ≤ 0.0001), while hash marks label statistically significant differences between respective CT and CFA groups (^#^*p* ≤ 0.05, ^*##*^*p* ≤ 0.01, ^*###*^*p* ≤ 0.001, ^*####*^*p* ≤ 0.0001) as analysed by one-way ANOVA followed by Tukeys' multiple comparison tests.

To validate the microarray results, the transcription levels of five differentially expressed, microarray-identified genes were further determined using quantitative real-time RT-PCR. The following genes were chosen for validation: *Lkaaear1, Neurod2* (Table 1), as well as G-protein coupled receptor 39 (*Gpr39*), kisspeptin (*Kiss1*) and kisspeptin-1 receptor (*Kiss1r*) (microarray data not shown). The relative fold changes (up-regulated) of *Gpr39* and *Lkaaear1* for CFA TRG samples were 3.04 and 4.01 respectively, while the relative fold changes (down-regulated) of *Kiss1, Kiss1r* and *Neurod2* were −1.74, −2.63, and −9.2 (Table 1, Supplementary Table 2). On day 7, *Gpr39* and *Kiss1r* alterations were similar to the microarray data (Figure 3). PCR results could not confirm microarray data on *Lkaaear1, Neurod2* and *Kiss1. Lkaaear1* presented decreased mRNA levels on day 7 compared to contralateral CT side. In addition, we were unable to detect *Neurod2* expression changes in TRG with our PCR protocol. We also chose to investigate the time course of neuronal and activation marker expressions. Although *Fosb*, Ionized calcium binding adaptor molecule 1 (*Iba1*), Glial fibrillary acidic protein (*Gfap*) and Calcitonin gene-related peptide (*Cgrp*) were not listed in microarray data, meaning no significant changes between the two groups of interest, we analysed the variation of these mRNA levels as well. No significant differences were detected on day 7 related to the mentioned genes which further confirmed the consistency and reliability of the microarray data.

#### Gene expression analysis in TRG tissues

We measured mRNA levels of eight genes in TRG tissues on three different time points after CFA injection. On day 1, CFA-induced significant up-regulation of Kiss1r, as well as of neuronal (*Fosb*), glial (*Iba1*), and astrocyte (*Gfap*) activation markers compared to saline-treated control group. By day 3, seven genes reached their maximum at a level of 9.18- (Gpr39), 2.97- (*Lkaaear1*), 9.51- (*Kiss1)*, 14.31- (*Kiss1r*), 117.82- (*Cgrp*), 7.40- (*Fosb*), and 27.80- fold (Gfap). Iba1 reached a 3.6-fold peak at day 1 before declining. mRNA levels of *Lkaaear1, Kiss1r, Iba1* gradually decreased at last time point until reaching a non-significant level compared to saline-treated control side (Figure 3).

#### Gene expression analysis in TNC tissues

**Figure 4:**
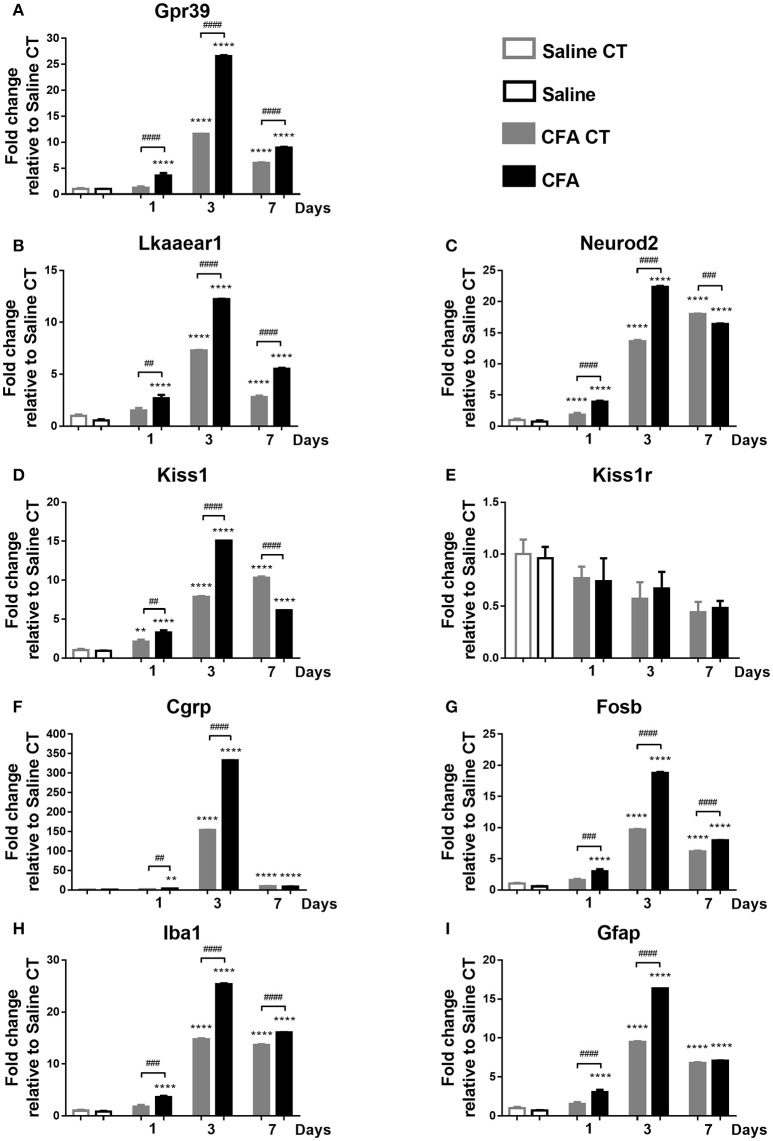
Time course of normalized fold changes in the trigeminal nucleus caudalis of Gpr39 **(A)**, Lkaaear1 **(B)**, Neurod2 **(C)**, Kiss1 **(D)**, Kiss1r **(E)**, Cgrp **(F)**, Fosb **(G)**, Iba1 **(H)**, and Gfap **(I)** mRNA expression one, 3 and 7 days after CFA injection. The mRNA levels were normalised to β2m and Hprt1, as detailed in materials and methods. Data are means ± S.E.M. (*n* = 3 at each time point). Asterisks denote statistically significant differences between CT Saline and CT/CFA groups (**p ≤ 0.01, ****p ≤ 0.0001), while hash marks label statistically significant differences between respective CT and CFA groups (^*##*^*p* ≤ 0.01, ^*###*^*p* ≤ 0.001, ^*####*^*p* ≤ 0.0001) as analysed by one-way ANOVA followed by Tukeys' multiple comparison tests.

Briefly, main changes in the relative gene expression were observed directly 1, 3 and 7 days post-CFA treatment. All measured mRNA levels, except *Kiss1r*, showed significantly altered temporal change in TNC of CFA-injected samples when compared to both CFA CT and Saline CT, presenting a maximum at day 3 (*p* ≤ 0.001 or *p* ≤ 0.0001). There was no significant difference in mRNA abundance of *Kiss1r* on different time points (Figure 4).

#### Gene expression analysis in PBMCs

**Figure 5:**
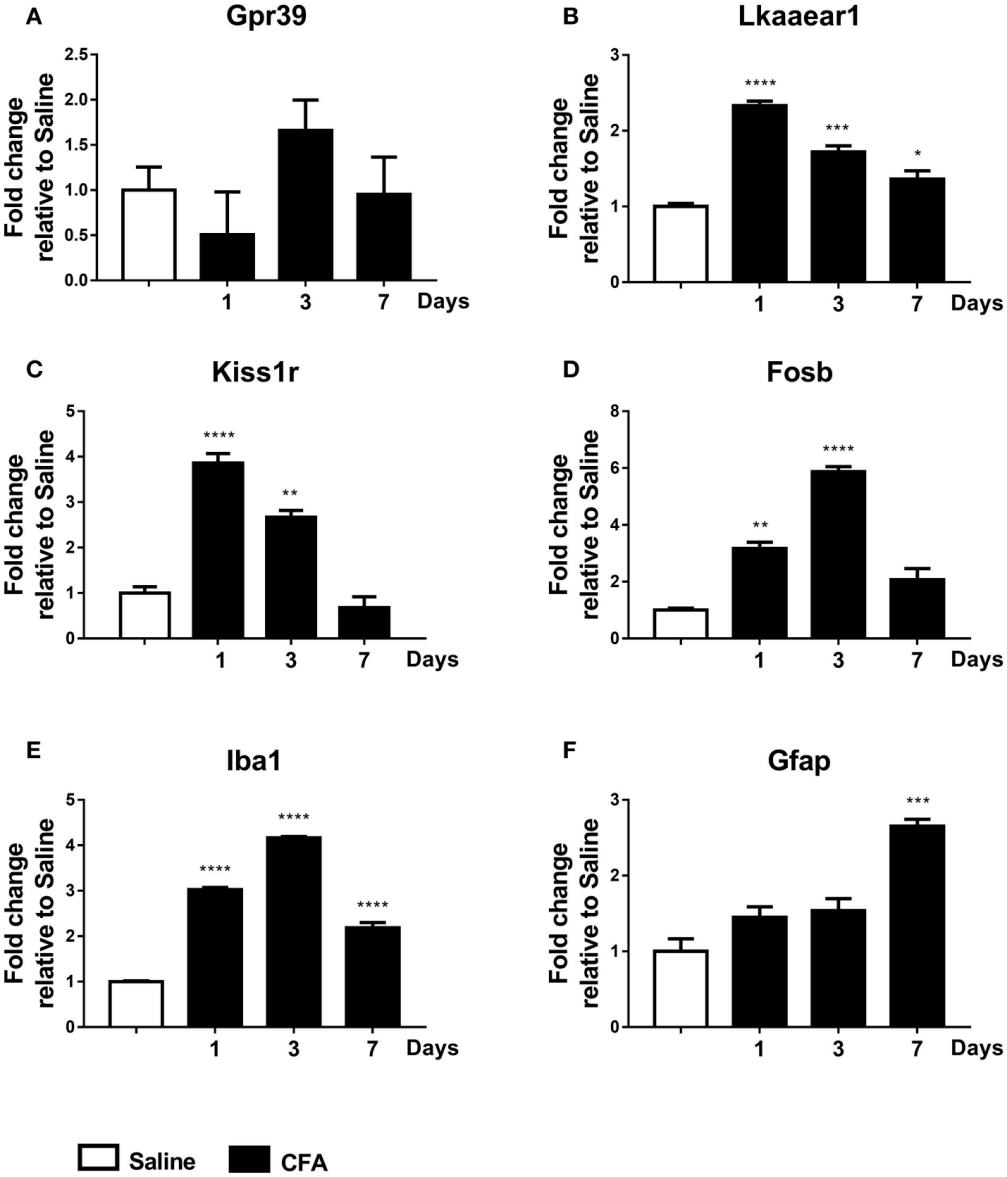
Time course of normalized fold changes in PBMC of Gpr39 **(A)**, Lkaaear1 **(B)**, Kiss1r **(C)**, Fosb **(D)**, Iba1 **(E)**, and Gfap **(F)** mRNA expression one, 3 and 7 days after CFA injection. The mRNA levels were normalised to Ppia and Hprt1, as detailed in materials and methods. Data are means ± S.E.M. (*n* = 3 at each time point). Asterisks denote statistically significant differences between respective Saline and CT/CFA groups (*p ≤ 0.05, **p ≤ 0.01, ***p ≤ 0.001, ****p ≤ 0.0001) as analysed by one-way ANOVA followed by Tukeys' multiple comparison tests.

Finally, low but significant expressional changes of *Lkaaear1* and *Kiss1r* gene mRNA in peripheral blood from CFA-treated rats have been observed. *Lkaaear1* displayed a gene expression pattern similar to *Kiss1r*, where Lkaaear1 presented a maximum of 2.33 and *Kiss1r* a 3.86 fold change at day one. We noted no significant changes in *Gpr39* mRNA levels of PBMCs after CFA exposure. Interestingly, *Fosb* and *Iba1* seem to be up-regulated (*p* ≤ 0.01 or *p* ≤ 0.001) at each time point due to CFA treatment, while Gfap only on day 7 (Figure 5).

#### Heat map plotting

**Figure 6:**
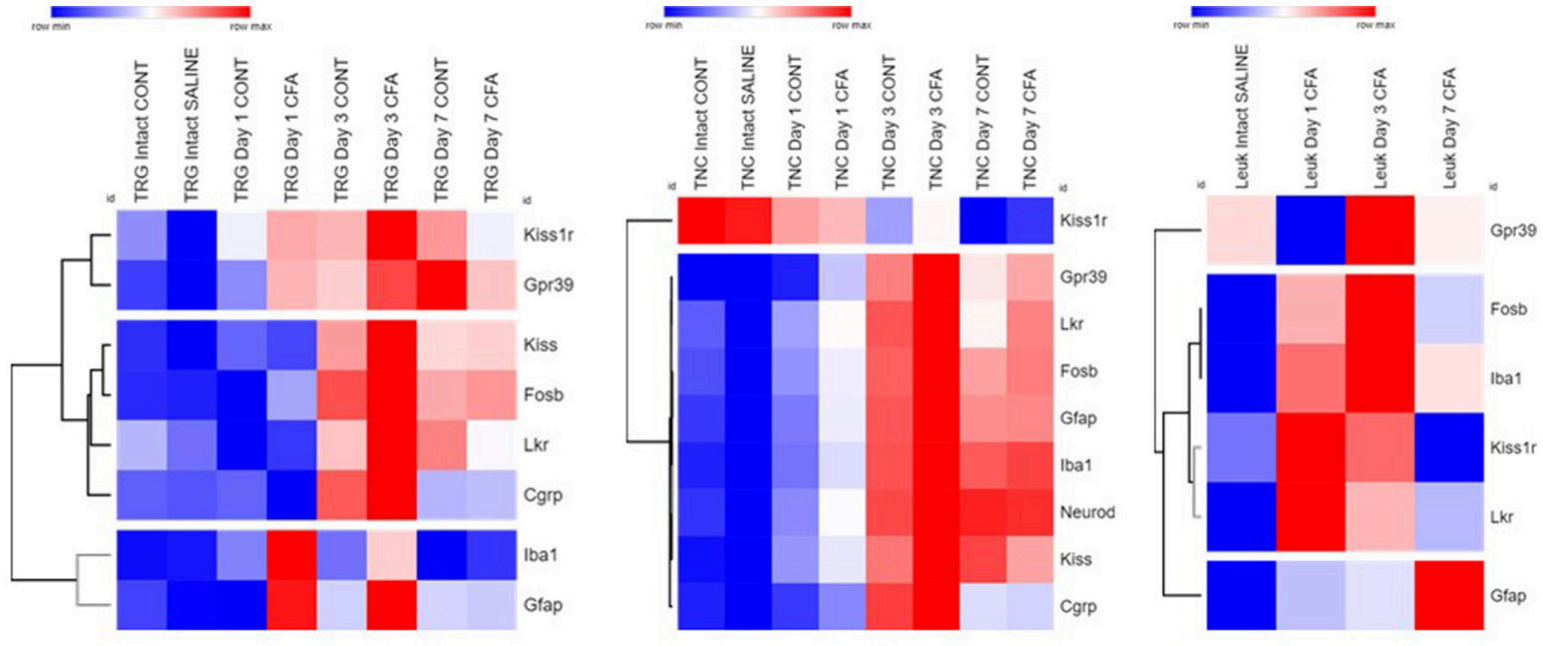
Summary of all qPCR results in TRG, TNC and PBMCs one, 3 and 7 days after CFA injection and in saline-treated controls. Fold change values were log2 transformed and plotted on a heat map. Rows: genes; columns: sample groups; scale: blue (low) to red (high). Hierarchical cluster analysis (1-Pearson correlation and average linkage method) was performed. Dendrograms on the left side of each figure demonstrate similarities between expression patterns of genes.

Fold change data were plotted on a heat map to summarize changes in mRNA levels measured by qPCR (Figure 6). Genes with a similar level of expression were grouped into three major clusters in TRG samples: *1. Kiss1r, Gpr39*; *2. Kiss, Fosb, Lkr, Cgrp*; *3. Gfap, Iba1*. In the TNC, all but one gene fall into a large cluster with highly similar temporal patterns, except for Kiss1r that changed the opposite way, however, its alterations were not found to be significant. Group 3 genes distinctly upregulated in CFA-treated TRG samples on days 1 and 3 but not on respective contralateral sides while they were substantially upregulated in TNC samples from both sides on these days, as well as on day 7. Genes upregulated in TRG and TNC were also elevated in PBMCs, although starting at an earlier time point (day 1) for most genes.

## Discussion

To our knowledge, this is the first comprehensive study which compared gene expression changes in the TRG, TNC and peripheral blood leukocytes in an inflammatory orofacial pain model. Simultaneous measurement of the transcriptional changes of PBMCs had been suggested to reflect alterations in the CNS (Arosio et al., 2014; Gerring et al., 2016; Srinivasan et al., 2017). We described up- and downregulation of distinct genes that are likely to be involved in the activation and sensitization of primary and secondary trigeminal neurons.

The mechanisms of nociceptor sensitization after inflammation have been extensively studied in rodents by electrophysiological, histological and molecular biological approaches (Hucho and Levine, 2007; Coste et al., 2008; Matsumoto et al., 2010; Cady et al., 2011; Bernstein and Burstein, 2012; Weyer et al., 2016). Nevertheless, we cannot extrapolate all the findings to the trigeminovascular system, since it is considerably different from other regions of the somatosensory system. As mentioned before, the central terminals of extra- and intracranial trigeminal primary afferents converge considerably in the TNC. As a consequence, inflammatory sensitization of primary meningeal afferents and secondary trigeminal neurons resulted in an enhanced response to cutaneous stimulation of the face (Burstein et al., 1998; Levy et al., 2004). On the other hand, experimental data also confirm that noxious stimulation (e.g., intranasal capsaicin), inflammation or nerve lesion on the face can induce meningeal vasodilation or neurogenic inflammation (Kunkler et al., 2011; Filipović et al., 2012). Intriguingly, it was revealed that there are trigeminal afferents which project to both the meninges and extracranial tissues (Schueler et al., 2013). Both human and rodent data point out that gene expression of TRGs is distinct from DRGs (Manteniotis et al., 2013; Flegel et al., 2015; Kogelman et al., 2017; LaPaglia et al., 2017). Yet, there have only been few rodent studies investigating gene expression changes in the TRGs after chronic orofacial inflammation (Okumura et al., 2010; Chung et al., 2016).

Our microarray study revealed a high number of differentially expressed olfactory, taste and pheromone receptor genes between the ipsi- and contralateral sides 7 days after CFA treatment. A large number of transcripts of chemoreceptors had been detected in murine and human TRGs using next-generation sequencing (Manteniotis et al., 2013), however their involvement in trigeminal sensitisation is not known. It is appealing to draw parallels between the perturbation of TRG chemoreceptors in our model and the known phenomena of an odour or perfume-triggered migraine, as well as odour hypersensitivity, osmophobia, odour hallucination and taste abnormalities associated with migraine (Schreiber and Calvert, 1986; Kelman, 2004; Goadsby et al., 2017). The microarray analysis implicated thyroid hormone receptor beta which had been previously associated with migraine (Gormley et al., 2015), and chemokine signalling (*Ccr7*), among many others as well as long non-coding RNAs (lncRNA) putatively involved in gene regulation.

On the basis of these microarray results, we further investigated the time-dependent changes of one of the most upregulated genes (*Lkaaear1*) and the most downregulated (*Neurod2*) gene with qPCR. Transcripts of genes with possible roles in nociception, which also have the potential to be future drug targets, were also chosen to be studied, such as two G-protein-coupled receptors (*Gpr39* and *Kiss1r*) and the neuropeptide kisspeptin (*Kiss1*). *Lkaaear1* encodes an LKAAEAR motive containing protein with unclear function. It is highly expressed in the brain and testis and during organ development (NCBI Gene database)^2^ *Neurod2* is involved in neuronal differentiation and has been implicated in synaptic plasticity (Bayam et al., 2015; Chen et al., 2016). Gpr39 is a Zn^2+^-sensing G_α*q*_-coupled receptor which is expressed in a wide range of tissues including some areas of the brain. Activation of Gpr39 induces the release of Ca^2+^ via the IP_3_ pathway. The receptor may play a role in depression, and as a specific and direct sensor of Zn^2+^, in many physiological functions where the cation is involved such as synaptic transmission (Popovics and Stewart, 2011; Sato et al., 2016). Kisspeptin, encoded by the Kiss1 gene, is considered to have an emerging role in the neuroendocrine regulation of reproduction and puberty (de Roux et al., 2003; Seminara, 2006; Kauffman et al., 2007; Colledge, 2009). Kisspeptin-expressing neurons and Kiss1r are found in areas other than the hypothalamus: amygdala, hippocampus, periaqueductal grey (Oakley et al., 2009; Herbison et al., 2010). In addition, DRG and dorsal horns neurons of the spinal cord have been shown to express kisspeptin and Kiss1r, whose expression might be upregulated due to intra-articular injection of CFA (Mi et al., 2009). There are studies showing hyperalgesic effect of peripheral and intrathecal kisspeptin (Spampinato et al., 2011). Likewise, i.c.v. administration of kisspeptin-10 induces both hyperalgesia and opioid antagonistic activity (Elhabazi et al., 2013), suggesting its possible involvement in the regulation of pain sensitivity.

We successfully reproduced the changes detected with the microarray by qPCR in cases of *Gpr39* and *Kiss1r. Neurod2* transcripts were not detected in the TRG and Lkaaear1 expression was higher on day 3 but not on day 7 compared to the contralateral side. It is important to highlight that there was a delayed but considerable increase of mRNA levels on the contralateral side of CFA-treated animals when compared to saline-treated animals. This is consistent with earlier reports found after inflammation or nerve injury of the hind limbs in which structural and biochemical changes appeared both centrally and in the periphery on the contralateral side (Koltzenburg et al., 1999; Shenker et al., 2003) However, we did not only use the contralateral side of CFA injected animals as controls, but we also included a saline-injected group as well. We aimed at keeping the animal number at a minimum level and meanwhile taking into account the possible trauma caused by only the injection itself. In addition, there was no detectable allodynia on the contralateral side in our model, therefore the comparison to the contralateral side is still valid from a functional aspect and provides additional information based on this double comparison.

We added Cgrp to the list of investigated markers to validate the model, since it is a well-known mediator and even a novel pharmacological target of migraine (Durham, 2006; Doods et al., 2007; Benemei et al., 2009; Edvinsson et al., 2012; Bigal et al., 2013; Russo, 2015). Moreover, its expression was shown to be elevated in TRGs in rodent models of orofacial inflammation (Yasuda et al., 2012; Shinoda and Iwata, 2013; Kuzawinska et al., 2014). Our results are consistent with these previous findings, Cgrp transcripts were significantly increased in the TRG at day 3 after CFA treatment corresponding to the peak of the facial allodynia.

In addition to the TRG, we also examined the transcriptional changes in the TNC, reflecting mechanisms involved in central sensitization, as well as PBMCs in the peripheral blood. In the TNC, significant changes were observed for the examined genes with the exception of *Kiss1r*. Intriguingly, the Kiss1 expression in the TNC was mirroring the changes of the receptor expression in the TRG which suggests a presynaptic effect on primary afferents. Lkaaear1 and *Kiss1r* expression were also significantly increased in PBMCs with a similar time course.

Besides allodynia, as the main functional parameter, neuronal and glial activation markers were also assessed by comparing their gene expression profiles. Therefore, we determined the widely-used neuronal activation marker *Fosb, Gfap* for astrocytes and *Iba1* for microglia (Nestler et al., 2001; Alibhai et al., 2007; Knight et al., 2011). *Gfap* has been shown to play a role in astrocyte migration, the function of the blood-brain barrier, signal transduction pathways and neuron-glia interactions (Middeldorp and Hol, 2011). *Iba1*, also known as *AIF1* (Allograft Inflammatory Factor 1) expressed in various cells such as monocyte/macrophages and activated T lymphocytes, is mostly used as a microglia marker (Kelemen and Autieri, 2005; Pawlik et al., 2016). All the three activation markers were significantly increased already at day 1 of the inflammation in both TRGs and TNCs, peaked by day 3 and decreased by day 7 when allodynia was declining. Remarkably and most interestingly, a smaller but significant increase of expression was also detectable in PBMCs which highlights the relevance of blood transcriptomics data in CNS diseases. To our knowledge, this is the first study to determine these transcripts in the peripheral blood of experimental animals, however, there are relevant human data for *Gfap* as a blood biomarker. It was first presented in acute stroke diagnosis in adults (Niebrój-Dobosz et al., 1994) and head trauma (Missler et al., 1999). Recently, it has been suggested that Gfap might be a potential biomarker of intracerebral haemorrhage (IHC) with symptoms of acute stroke (Brunkhorst et al., 2010; Mayer et al., 2013; Foerch et al., 2015). It is also an early marker of traumatic brain injury (Bembea et al., 2011; Lei et al., 2015), during different phases of cardiopulmonary bypass (Vedovelli et al., 2017), with predictiveness of neurological outcome (Lei et al., 2015). It is clear that the measurement of *Gfap* changes at the periphery is not a specific diagnostic tool and it is too early to draw a final conclusion on its utility at this stage. However, it would be interesting to see in future studies whether it could have a prognostic value to predict the conversion of orofacial pain or headache conditions from episodic to chronic. In our model, Gfap expression remained high even at the end of the experiment which could reflect a persistent neuroinflammation.

In conclusion, the main novelty of the present findings is the description of some up- and downregulated genes at the levels of both primary and secondary sensory neurones of the trigeminovascular system that might play important roles in neuroinflammatory activation mechanisms. Furthermore, we are the first to show transcriptomic alterations in the PBMCs that are similar to the changes detected in the neuronal tissues. These results open new perspectives and initiate further investigations in the research of trigeminal pain disorders.

## Author contributions

ZH, KB, and ÉS: Study concept and design; KB, TA, and ÉS: Animal model, behavioural studies and sample collection conducted by; JK, SJ, AG, TR, TA: Microarray/qPCR analysis and interpretation of genetic data; ZH and TR: Funding. All authors contributed to the analysis of the results, drafting of manuscript and approved the final version.

### Conflict of interest statement

The authors declare that the research was conducted in the absence of any commercial or financial relationships that could be construed as a potential conflict of interest.

## Footnotes

^1^https://software.broadinstitute.org/morpheus/ Broad Institute, Cambridge, MA, USA.

^2^https://www.ncbi.nlm.nih.gov/gene/198437

## Abbreviations

TRG: trigeminal ganglion
TNC: trigeminal nucleus caudalis
PBMC: peripheral blood mononuclear cells
CFA: Complete Freund's Adjuvant
Iba1: Ionized calcium binding adaptor molecule 1
Gfap: Glial fibrillary acidic protein.
