# Supplementary material for: Transcriptional Alterations in the Trigeminal Ganglia, Nucleus and Peripheral Blood Mononuclear Cells in a Rat Orofacial Pain Model
Source: Front Mol Neurosci. 2018 Jun 26;11:219. doi: 10.3389/fnmol.2018.00219 (PMC6028693; doi:10.3389/fnmol.2018.00219)
Supplement: Supplementary file 1 [file Table_1.PDF]

Supplementary Table 1. Sequences of primers for real-time PCR.

Gene symbol	Accession number	Forward primer sequence (F, 5' - 3')<br>Reverse primer sequence (R, 5' - 3')
Aif1 (Iba1)	NM_017196;<br>XM_006256062;<br>XM_006256065;<br>XM_006256061;<br>XM_006256063	F: TCC GAG GAG ACG TTC AGT TA<br>R: GTT GGC TTC TGG TGT TCT TTG
B2m	NM_012512	F: CCC ACC CTC ATG GCT ACT TC<br>R: CCA CTT CAC TTC ACT CTG GCA
Calca (Cgrp)	NM_017338;<br>NM_001033955;<br>NM_001033956;<br>XM_008759676	F: TTG TCA GCA TCT TGC TCC TGT AC<br>R: GCC TGG GCT GCT TTC CA
Fosb	NM_001256509	F: CAC TTC CAA CAT GTC TCC TCT C<br>R: CCA CCC AGT CAC ACT TAC TTA C
Gapdh	NM_017008	F: GTA ACC AGG CGT CCG ATA C<br>R: TCC TCT GCT CCT CCC TGT TC
Gfap	NM_017009	F: GAT CCG AGA AAC CAG CCT GGA C<br>R: TGG GCA CAC CTC ACA TCA CAT
Gpr39	NM_001114392;<br>NM_001100943	F: GTC TTC CAG TCC AGC ATC TTT<br>R: GCT TGC TCT TCA TTA GCA CTT TC
Hprt1	NM_012583	F: GCT TTT CCA ACT TTC GCT GAT G<br>R: GGT GAA AAG GAC CTC TCG AAG
Kiss1	NM_181692;<br>XM_008769443;<br>XM_017598697	F: ATG ATC TCG CTG GCT TCT TGG<br>R: GGT TCA CCA CAG GTG CCA TTT T
Kiss1r	NM_001301151;<br>NM_023992	F: TTC TAC ATC GCT AAC CTG GC<br>R: AAA GTG GCA CAT GTG GCT TG

Lkaaear1	NM_001106551;<br>XM_006235763;<br>XM_008762502;<br>XM_006235764;<br>XM_008762501	F: CTC TCC TGA TCC AGA AGC AAA G<br>R: GTC CAA AGG ATC AGG GAT CTT C
Neurod2	NM_019326	F: GGC TCT CTC GGA GAT CTT GC<br>R: TGC TCC GTG AGG AAG TTA CG
Ppia	NM_017101	F: CCA TTA TGG CGT GTG AAG TC<br>R: GCA GAC AAA GTT CCA AAG ACA G
