# Supplementary material for: Transcriptional Alterations in the Trigeminal Ganglia, Nucleus and Peripheral Blood Mononuclear Cells in a Rat Orofacial Pain Model
Source: Front Mol Neurosci. 2018 Jun 26;11:219. doi: 10.3389/fnmol.2018.00219 (PMC6028693; doi:10.3389/fnmol.2018.00219)
Supplement: Supplementary file 2 [file Table_2.PDF]

Supplementary Table 2. List of differentially expressed genes (P value < 0.05; absolute fold change > 2.0) between the control (contralateral) and 7-day CFA (ipsilateral) samples from TRG detected by microarray. ID: microarray feature identifier; mean_CFA-treated: mean expression level for the specified sample group; mean_control: mean expression level for the specified sample group; sd_CFA-treated: standard deviation (of the expression) for the specified sample group; sd_control: Standard deviation (of the expression) for the specified sample group; FC: expression ratio (fold-change) between CFA-treated and contralateral sample groups; logFC: expression ratio (fold-change) between the compared sample groups on the 2-logarithmics scale; P.Value: T-test p-value for the comparison between the sample groups;

ID	mean_CFA-treated	mean_control	sd_CFA-treated	sd_control	FC	logFC	P.Value	SystematicName	ProbeName	SeqID	GeneSymbol	Description	EnsemblID	EntrezGene
948	1842	508	1391	577	5,2	2,379	0,03805659	MRAK049104	MRAK049104_P1	MRAK049104	NA	lncRNA	NA	NA
11131	26	3	28	2	4,547	2,185	0,035263669	TC598318	A_43_P14304	TC598318	NA	NA	NA	NA
9725	81	35	9	33	4,353	2,122	0,021404535	NM_199489.3	CUST_9949_PI421866198	NM_199489	Ccr7	C-C motif chemokine receptor 7	ENSRNOG00000010665	287673
5534	36	10	23	9	4,342	2,118	0,007565042	NM_001011951.1	CUST_382_PI421866198	NM_001011951	Sf3b4	splicing factor 3b, subunit 4	ENSRNOG00000021181	295270
12278	45	13	5	10	4,286	2,1	0,001028331	NM_001037518.1	CUST_11171_PI421866198	NM_001037518	Defb23	defensin beta 23	ENSRNOG00000023477	641621
519	67	28	15	25	4,283	2,099	0,021757277	NM_001000099.1	CUST_2409_PI421866198	NM_001000099	Olr1640	olfactory receptor 1640	ENSRNOG00000048857	290049
13893	22	5	13	5	4,133	2,047	0,02355108	NM_001106821.1	CUST_8917_PI421866198	NM_001106821	Atm	ATM serine/threonine kinase	ENSRNOG00000029773	300711
40839	3082	959	1526	701	4,084	2,03	0,013457019	NM_134399.2	CUST_4141_PI421866198	NM_134399	Mk1	Mk1 protein	ENSRNOG00000019657	171436
6132	213	53	190	38	4,006	2,002	0,017206656	NM_001106551.1	CUST_10795_PI421866198	NM_001106551	Lkaaear1	LKAAEAR motif containing 1	ENSRNOG00000024815	296483
10158	45	24	9	24	3,834	1,939	0,0477078	NM_001013956.1	CUST_8739_PI421866198	NM_001013956	RGD1309049	similar to RIKEN cDNA 4933415F23	ENSRNOG00000014123	301306
10494	57	23	11	18	3,795	1,924	0,013809329	NM_001013147.1	CUST_14090_PI421866198	NM_001013147	Axl	Axl receptor tyrosine kinase	ENSRNOG00000020716	308444
2571	125	65	27	64	3,775	1,916	0,040353257	NM_019128.4	CUST_6718_PI421866198	NM_019128	Ina	internexin neuronal intermediate filament protein, alpha	ENSRNOG00000020248	24503
12538	45	7	75	3	3,728	1,899	0,023237809	NM_198133.2	CUST_335_PI421866198	NM_198133	Uts2b	urotensin 2B	ENSRNOG00000038512	378939
3346	70	29	13	26	3,723	1,896	0,015558789	NM_001047878.1	CUST_634_PI421866198	NM_001047878	F5	coagulation factor V	ENSRNOG00000057855	NA
6931	106	48	15	44	3,709	1,891	0,019766522	uc.339+	uc.339+_P1	uc+	NA	lncRNA	NA	NA
5544	61	30	10	27	3,701	1,888	0,031642178	NM_001001034.1	CUST_14837_PI421866198	NM_001001034	Olr199	olfactory receptor 199	ENSRNOG00000029755	405920
9136	46	22	5	21	3,689	1,883	0,024979557	NM_012735.1	CUST_9605_PI421866198	NM_012735	Hk2	hexokinase 2	ENSRNOG00000006116	25059
4102	35	15	8	13	3,669	1,875	0,021200105	NM_001001010.1	CUST_14907_PI421866198	NM_001001010	Olr283	olfactory receptor 283	ENSRNOG00000030782	405384
3492	31	13	3	11	3,666	1,874	0,01590538	uc.470+	uc.470+_P1	uc+	NA	lncRNA	NA	NA
1789	36	16	4	13	3,661	1,872	0,023975719	XR_005913	XR_005913_P1	XR_005913	NA	lncRNA	NA	NA
4451	23	9	2	8	3,643	1,865	0,006408873	NM_173305.1	CUST_2590_PI421866198	NM_173305	Hsd17b6	hydroxysteroid (17-beta) dehydrogenase 6	ENSRNOG00000002597	286964
11677	121	40	25	26	3,628	1,859	0,001272135	NM_001000387.1	CUST_595_PI421866198	NM_001000387	Olr416	olfactory receptor 416	ENSRNOG00000029069	296678
6676	30	17	4	19	3,621	1,856	0,032176079	NM_001107582.2	CUST_7741_PI421866198	NM_001107582	Pdcd1lg2	programmed cell death 1 ligand 2	ENSRNOG00000016136	309304
9724	125	58	11	47	3,609	1,852	0,024710012	NM_153466.1	CUST_4426_PI421866198	NM_153466	Gzmf	granzyme F	ENSRNOG00000028810	266704
5556	31	13	2	11	3,599	1,848	0,010289419	NM_001099514.1	CUST_12941_PI421866198	NM_001099514	Vom2r48	vomeronasal 2 receptor, 48	ENSRNOG00000028538	686145
10247	94	39	34	31	3,527	1,818	0,01751188	NM_024376.1	CUST_12366_PI421866198	NM_024376	Gja3	gap junction protein, alpha 3	ENSRNOG00000008847	79217
3605	51	24	6	21	3,458	1,79	0,021564138	uc.236+	uc.236+_P1	uc+	NA	lncRNA	NA	NA
521	33	15	6	13	3,45	1,786	0,025895429	NM_173112.2	CUST_3784_PI421866198	NM_173112	NA	NA	NA	NA
2398	9	2	4	0	3,427	1,777	7,99E-05	NM_001008907.1	CUST_1720_PI421866198	NM_001008907	NA	NA	NA	NA
2892	29	13	4	10	3,424	1,776	0,015506206	NM_001000982.1	CUST_12291_PI421866198	NM_001000982	Olr1185	olfactory receptor 1185	ENSRNOG00000039892	405337
12708	9	2	7	0	3,416	1,772	0,003304453	uc.21-	uc.21-_P1	uc-	NA	NA	NA	NA
1471	811	180	835	107	3,408	1,769	0,031379127	NM_001000263.1	CUST_1191_PI421866198	NM_001000263	Olr384	olfactory receptor 384	ENSRNOG00000032117	293817
4197	9	3	4	1	3,402	1,766	0,000190532	NM_001136141.1	CUST_2176_PI421866198	NM_001136141	Treh	trehalase	ENSRNOG00000052010	60576
1727	65	34	8	30	3,378	1,756	0,037722915	AF140345	AF140345_P1	AF140345	NA	lncRNA	NA	NA
7167	135	65	41	57	3,349	1,744	0,032291793	NM_001000934.1	CUST_12425_PI421866198	NM_001000934	Olr463	olfactory receptor 463	ENSRNOG00000060217	405255
1127	66	36	14	36	3,298	1,721	0,0358504	MRuc007grn	MRuc007grn_P1	MRuc007grn	NA	lncRNA	NA	NA
11341	54	17	20	10	3,273	1,711	0,000412202	NM_147144.1	CUST_1930_PI421866198	NM_147144	Casc3	cancer susceptibility candidate 3	ENSRNOG00000009716	NA
7249	36	17	6	16	3,257	1,704	0,021890097	MRAK140563	MRAK140563_P1	MRAK140563	NA	lncRNA	NA	NA
5803	16	6	2	4	3,246	1,699	0,00287844	NM_001008967.1	CUST_1556_PI421866198	NM_001008967	NA	NA	NA	NA
3323	154	80	27	65	3,239	1,695	0,040571417	NM_001000093.1	CUST_2415_PI421866198	NM_001000093	Olr1608	olfactory receptor 1608	ENSRNOG00000012522	290006
11342	84	28	47	17	3,229	1,691	0,006491192	NM_138851.1	CUST_3448_PI421866198	NM_138851	Prok1	prokineticin 1	ENSRNOG00000018201	192205
2476	60	32	6	30	3,222	1,688	0,036906013	NR_002149	NR_002149_P1	NR_002149	NA	lncRNA	NA	NA
6385	31	17	4	14	3,2	1,678	0,044430342	NM_214821.1	CUST_4214_PI421866198	NM_214821	Olr1413	olfactory receptor 1413	ENSRNOG00000026801	252887
1368	27	13	4	11	3,198	1,677	0,024306834	XR_007924	XR_007924_P1	XR_007924	NA	lncRNA	NA	NA
3844	26	12	4	9	3,191	1,674	0,014149267	uc.81+	uc.81+_P1	uc+	NA	lncRNA	NA	NA
8549	40	22	6	22	3,186	1,672	0,039246466	NM_001000995.1	CUST_12256_PI421866198	NM_001000995	Olr1590	olfactory receptor 1590	ENSRNOG00000030059	NA
5139	21	11	3	10	3,173	1,666	0,024082287	XR_006115	XR_006115_P1	XR_006115	NA	lncRNA	NA	NA
2306	64	24	35	16	3,17	1,664	0,013797195	NM_022677.1	CUST_1674_PI421866198	NM_022677	Zic1	Zic family member 1	ENSRNOG00000014644	64618
3587	148	72	19	56	3,167	1,663	0,026556964	NM_001000113.1	CUST_2015_PI421866198	NM_001000113	Olr8	olfactory receptor 8	ENSRNOG00000030070	292563
3093	26	12	2	9	3,155	1,658	0,015025859	NM_031815.2	CUST_9935_PI421866198	NM_031815	Inhbe	inhibin beta E subunit	ENSRNOG00000007601	83711
16427	132	27	136	10	3,15	1,655	0,040214425	NM_012519.2	CUST_11108_PI421866198	NM_012519	Camk2d	calcium/calmodulin-dependent protein kinase II delta	ENSRNOG00000011589	24246
12698	23	12	4	13	3,139	1,65	0,019486378	NM_001000776.1	CUST_13741_PI421866198	NM_001000776	Olr1442	olfactory receptor 1442	ENSRNOG00000031246	405051
3417	61	30	6	23	3,119	1,641	0,028781366	NM_001000389.1	CUST_591_PI421866198	NM_001000389	Olr420	olfactory receptor 420	ENSRNOG00000007965	296682
5476	47	21	18	15	3,103	1,634	0,022062783	NM_001105854.1	CUST_12497_PI421866198	NM_001105854	NA	NA	NA	NA
2648	17	8	2	7	3,102	1,633	0,011151	NM_001014771.1	CUST_7307_PI421866198	NM_001014771	Ervfrd-1	endogenous retrovirus group FRD member 1	ENSRNOG00000050672	288602
15799	153	47	107	34	3,095	1,63	0,049125188	AB074423	AB074423_P1	AB074423	NA	lncRNA	NA	NA
5043	127	62	11	50	3,092	1,628	0,022603618	XR_009248	XR_009248_P1	XR_009248	NA	lncRNA	NA	NA
11001	22	10	4	7	3,083	1,624	0,013512299	NM_001109596.1	CUST_1148_PI421866198	NM_001109596	Tas2r125	taste receptor, type 2, member 125	ENSRNOG00000021397	690481
3345	40	17	6	12	3,079	1,622	0,005877237	NM_021659.1	CUST_5299_PI421866198	NM_021659	Syt7	synaptotagmin 7	ENSRNOG00000026432	59267
2484	73	40	13	36	3,07	1,618	0,043230549	MRAK152421	MRAK152421_P1	MRAK152421	NA	lncRNA	NA	NA
8441	27	13	5	12	3,062	1,615	0,022235748	NM_001000344.1	CUST_740_PI421866198	NM_001000344	Olr658	olfactory receptor 658	ENSRNOG00000046684	295864
9972	7769	4029	2064	3434	3,057	1,612	0,039656513	NM_001109544.1	CUST_1330_PI421866198	NM_001109544	Pdcd2l	programmed cell death 2-like	ENSRNOG00000021119	689637
35554	67	20	53	12	3,044	1,606	0,009510563	NM_001108494.1	CUST_5318_PI421866198	NM_001108494	Fam168a	family with sequence similarity 168, member A	ENSRNOG00000018873	361614
1365	27	14	8	13	3,044	1,606	0,04176614	MRAK010309	MRAK010309_P1	MRAK010309	NA	lncRNA	NA	NA
6044	137	52	28	33	3,042	1,605	0,001296037	NM_031087.2	CUST_15155_PI421866198	NM_031087	Psen2	presenilin 2	ENSRNOG00000002879	81751
2729	755	406	108	317	3,039	1,604	0,043283231	NM_001114392.1	CUST_9281_PI421866198	NM_001114392	Gpr39	G protein-coupled receptor 39	ENSRNOG00000021586	288995
970	8	3	2	0	3,026	1,597	6,86E-06	NM_001001024.1	CUST_14861_PI421866198	NM_001001024	Olr95	olfactory receptor 95	ENSRNOG00000045808	405909
2491	47	26	8	24	3,025	1,597	0,044963114	NM_001008757.1	CUST_3076_PI421866198	NM_001008757	Krt33a	NA	NA	NA
3151	39	23	5	21	3,025	1,597	0,049200837	MRAK020590	MRAK020590_P1	MRAK020590	NA	lncRNA	NA	NA
4034	21	10	2	8	3,021	1,595	0,014384245	MRAK036053	MRAK036053_P1	MRAK036053	NA	lncRNA	NA	NA
3999	123	57	12	42	3,016	1,592	0,01673621	M77835	M77835_P1	M77835	NA	lncRNA	NA	NA
10915	53	24	7	17	3,015	1,592	0,016594932	NM_053423.1	CUST_4016_PI421866198	NM_053423	Tert	telomerase reverse transcriptase	ENSRNOG00000025327	301965
12440	17	6	19	5	3,006	1,588	0,031656138	uc.59+	uc.59+_P1	uc+	NA	NA	NA	NA
4025	8	3	1	1	3,004	1,587	5,55E-06	NM_001047935.1	CUST_218_PI421866198	NM_001047935	LOC498836	LRRG00125	ENSRNOG00000025687	498836
2486	23	12	1	10	3,003	1,587	0,02654604	XR_008403	XR_008403_P1	XR_008403	NA	lncRNA	NA	NA
11601	962	475	191	359	3	1,585	0,028403136	NM_001106764.1	CUST_9302_PI421866198	NM_001106764	Morc2b	microrchidia 2B	ENSRNOG00000004501	299555
1458	80	43	8	37	2,999	1,585	0,03617731	MRuc008dua	MRuc008dua_P1	MRuc008dua	NA	lncRNA	NA	NA
6801	60	27	27	21	2,993	1,582	0,039027716	NM_013005.1	CUST_10883_PI421866198	NM_013005	Pik3r1	phosphoinositide-3-kinase regulatory subunit 1	ENSRNOG00000018903	25513
11267	58	17	41	13	2,982	1,576	0,027791765	NM_001104633.1	CUST_1552_PI421866198	NM_001104633	Sema3d	semaphorin 3D	ENSRNOG00000007202	246262
5631	78	43	16	37	2,981	1,576	0,048166516	MRuc008pze	MRuc008pze_P1	MRuc008pze	NA	lncRNA	NA	NA
4042	42	23	6	21	2,967	1,569	0,033245945	NM_001024881.1	CUST_12798_PI421866198	NM_001024881	Tns4	tensin 4	ENSRNOG00000027350	303517
2893	117	62	23	51	2,964	1,567	0,038696675	MRAK034607	MRAK034607_P1	MRAK034607	NA	lncRNA	NA	NA
2409	29	15	6	14	2,961	1,566	0,034943039	NM_001008852.1	CUST_2303_PI421866198	NM_001008852	RT1-M6-1	RT1 class I, locus M6, gene 1	ENSRNOG00000022107	365527
6396	16	7	2	5	2,957	1,564	0,007506664	NM_001008944.1	CUST_1586_PI421866198	NM_001008944	NA	NA	NA	NA
373	19	9	3	8	2,955	1,563	0,01711468	uc.440+	uc.440+_P1	uc+	NA	lncRNA	NA	NA
13303	16	6	5	4	2,941	1,556	0,003143596	NM_001000392.1	CUST_557_PI421866198	NM_001000392	Olr424	olfactory receptor 424	ENSRNOG00000007977	296686
1645	98	50	7	42	2,932	1,552	0,020550362	NM_001108473.1	CUST_5378_PI421866198	NM_001108473	Pnma8a	NA	NA	NA
3103	34	18	3	14	2,932	1,552	0,032384219	NM_001000546.1	CUST_15035_PI421866198	NM_001000546	Olr152	olfactory receptor 152	ENSRNOG00000017211	365332
3830	19	8	7	5	2,928	1,55	0,011652925	NM_012695.3	CUST_10205_PI421866198	NM_012695	Sult2a6	NA	NA	NA
1984	10	4	3	2	2,925	1,548	0,000581953	NM_001107852.1	CUST_6317_PI421866198	NM_001107852	NA	NA	NA	NA
42888	1475	608	562	396	2,922	1,547	0,013282181	NM_022400.1	CUST_3216_PI421866198	NM_022400	Bcat2	branched chain amino acid transaminase 2	ENSRNOG00000020956	64203
3402	15	6	7	4	2,887	1,529	0,008002987	NM_001000876.1	CUST_13003_PI421866198	NM_001000876	Olr1144	olfactory receptor 1144	ENSRNOG00000032512	405174
1131	52	27	7	22	2,878	1,525	0,022785484	NM_001000081.1	CUST_2462_PI421866198	NM_001000081	Olr1584	olfactory receptor 1584	ENSRNOG00000003498	406005
3954	148	82	19	68	2,877	1,524	0,043117635	NM_001008936.1	CUST_1623_PI421866198	NM_001008936	NA	NA	NA	NA
6131	61	34	5	26	2,877	1,524	0,047404841	NM_017183.1	CUST_12613_PI421866198	NM_017183	Cxcr2	C-X-C motif chemokine receptor 2	ENSRNOG00000014269	29385
963	8	3	2	1	2,875	1,524	1,47E-05	NM_001024366.1	CUST_503_PI421866198	NM_001024366	Arl13a	ADP ribosylation factor like GTPase 13A	ENSRNOG00000055454	501617
12025	12	3	16	2	2,872	1,522	0,031782911	NM_001000126.1	CUST_1984_PI421866198	NM_001000126	Olr41	olfactory receptor 41	ENSRNOG00000012071	293195
8189	149	81	20	69	2,864	1,518	0,033989103	NM_001000777.1	CUST_13740_PI421866198	NM_001000777	Olr1435	olfactory receptor 1435	ENSRNOG00000029289	405053
4024	92	44	31	27	2,856	1,514	0,03952761	NM_001009506.1	CUST_975_PI421866198	NM_001009506	NA	NA	NA	NA
1230	25	12	10	10	2,855	1,513	0,034753896	XR_007096	XR_007096_P1	XR_007096	NA	lncRNA	NA	NA
2838	205	57	161	30	2,852	1,512	0,036838567	AY321332	AY321332_P1	AY321332	NA	lncRNA	NA	NA
6513	326	188	80	173	2,845	1,508	0,049658471	MRAK086453	MRAK086453_P1	MRAK086453	NA	lncRNA	NA	NA
43702	1506	613	484	364	2,837	1,505	0,007871663	NM_001047886.1	CUST_596_PI421866198	NM_001047886	Ilf2	interleukin enhancer binding factor 2	ENSRNOG00000014154	310612
8038	21	12	4	11	2,83	1,501	0,042780242	NM_001106068.1	CUST_14257_PI421866198	NM_001106068	B3gnt3	UDP-GlcNAc:betaGal beta-1,3-N-acetylglucosaminyltransferase 3	ENSRNOG00000018764	290638
39534	15	4	13	3	2,829	1,5	0,027824802	MRuc009uwk	MRuc009uwk_P1	MRuc009uwk	NA	NA	NA	NA
2400	220	115	53	105	2,828	1,5	0,028783616	NM_052829.1	CUST_4958_PI421866198	NM_052829	Rims1	regulating synaptic membrane exocytosis 1	ENSRNOG00000011000	84556
2144	77	43	11	37	2,826	1,499	0,037089925	NM_001108190.1	CUST_7212_PI421866198	NM_001108190	Rtp3	receptor (chemosensory) transporter protein 3	ENSRNOG00000037167	316018
4621	7	2	1	1	2,813	1,492	4,94E-06	NM_001000329.1	CUST_787_PI421866198	NM_001000329	Olr588	olfactory receptor 588	ENSRNOG00000005793	295822
303	19	10	3	7	2,813	1,492	0,020180539	AY383713	AY383713_P1	AY383713	NA	lncRNA	NA	NA
7867	15	7	3	5	2,8	1,485	0,008030219	NM_134385.2	CUST_4188_PI421866198	NM_134385	Prl8a9	prolactin family 8, subfamily a, member 9	ENSRNOG00000024843	171406
3522	22	10	3	6	2,788	1,479	0,00680213	NM_001000524.1	CUST_15060_PI421866198	NM_001000524	Olr1393	olfactory receptor 1393	ENSRNOG00000047749	363585
2072	15	8	2	6	2,782	1,476	0,015152016	MRAK090266	MRAK090266_P1	MRAK090266	NA	lncRNA	NA	NA
7003	393	206	80	163	2,775	1,472	0,032952845	NM_001108392.1	CUST_5980_PI421866198	NM_001108392	Zic2	Zic family member 2	ENSRNOG00000054879	361096
4452	119	61	16	48	2,767	1,469	0,024019635	MRAK014161	MRAK014161_P1	MRAK014161	NA	lncRNA	NA	NA
4268	15	8	2	6	2,761	1,465	0,016319267	NM_001135710.1	CUST_1847_PI421866198	NM_001135710	Sec14l5	SEC14-like lipid binding 5	ENSRNOG00000002917	287060
43826	13	3	14	1	2,755	1,462	0,035648829	NM_053584.2	CUST_3205_PI421866198	NM_053584	Gosr1	golgi SNAP receptor complex member 1	ENSRNOG00000003971	94189
12362	25	12	3	9	2,75	1,46	0,018978651	NM_022930.1	CUST_164_PI421866198	NM_022930	Chrna9	cholinergic receptor nicotinic alpha 9 subunit	ENSRNOG00000002484	65024
3325	20	9	2	5	2,746	1,458	0,006449284	NM_001107885.1	CUST_6256_PI421866198	NM_001107885	Bcl2l13	BCL2 like 13	ENSRNOG00000012394	312682
5999	108	56	34	45	2,74	1,454	0,040241989	NM_001108368.1	CUST_6065_PI421866198	NM_001108368	Dupd1	dual specificity phosphatase and pro isomerase domain containing 1	ENSRNOG00000013034	361003
362	35	18	5	15	2,739	1,454	0,025829001	AY539933	AY539933_P1	AY539933	NA	lncRNA	NA	NA
7064	60	30	7	22	2,737	1,453	0,017313341	NM_001008887.1	CUST_2249_PI421866198	NM_001008887	Serpinb3	serpin family B member 3	ENSRNOG00000031134	304688
4508	20	6	21	4	2,731	1,45	0,043768134	NM_130424.2	CUST_1065_PI421866198	NM_130424	Tmprss2	transmembrane protease, serine 2	ENSRNOG00000001976	156435
10662	19	10	3	7	2,729	1,449	0,023412152	NM_012599.2	CUST_10946_PI421866198	NM_012599	Mbl1	mannose-binding lectin (protein A) 1	ENSRNOG00000011706	24548
1805	31	16	6	12	2,72	1,444	0,028073191	NM_139335.1	CUST_3198_PI421866198	NM_139335	Tas2r126	taste receptor, type 2, member 126	ENSRNOG00000017881	246219
2595	32	18	5	14	2,719	1,443	0,046028142	AY539949	AY539949_P1	AY539949	NA	lncRNA	NA	NA
6849	31	17	4	13	2,701	1,434	0,028821566	NM_130402.2	CUST_1117_PI421866198	NM_130402	Clec2d	C-type lectin domain family 2, member D	ENSRNOG00000007866	113937
867	48	27	6	21	2,701	1,434	0,042407753	AJ286155	AJ286155_P1	AJ286155	NA	lncRNA	NA	NA
4357	80	46	15	40	2,691	1,428	0,047831279	MRAK081379	MRAK081379_P1	MRAK081379	NA	lncRNA	NA	NA
8361	27	14	5	11	2,688	1,427	0,032062167	NM_001108990.1	CUST_2131_PI421866198	NM_001108990	Caps2	calcyphosine 2	ENSRNOG00000026600	366891
4291	6	2	3	0	2,687	1,426	0,001342149	NM_139262.1	CUST_3776_PI421866198	NM_139262	Ctsq	cathepsin Q	ENSRNOG00000017946	246147
532	15	7	3	5	2,678	1,421	0,017126324	uc.301-	uc.301-_P1	uc-	NA	NA	NA	NA
1033	41	23	3	18	2,675	1,419	0,045418994	NM_012616.1	CUST_10428_PI421866198	NM_012616	Omp	NA	NA	NA
7425	24	13	3	10	2,668	1,416	0,033625367	XR_007907	XR_007907_P1	XR_007907	NA	lncRNA	NA	NA
6960	32	11	22	7	2,667	1,415	0,048646056	NM_001106356.1	CUST_12181_PI421866198	NM_001106356	Zfand5	zinc finger AN1-type containing 5	ENSRNOG00000018107	293960
11603	168	91	20	69	2,654	1,408	0,03357509	NM_001037509.1	CUST_11197_PI421866198	NM_001037509	Defb9	defensin beta 9	ENSRNOG00000038149	641633
13469	8	4	1	2	2,652	1,407	0,001214462	NM_001106554.1	CUST_10790_PI421866198	NM_001106554	Il36a	interleukin 36, alpha	ENSRNOG00000005722	296541
3429	11	4	3	2	2,64	1,401	0,000331686	uc.193+	uc.193+_P1	uc+	NA	NA	NA	NA
7677	84	41	9	30	2,637	1,399	0,012056045	NM_001007719.3	CUST_6587_PI421866198	NM_001007719	Hsd3b1	NA	NA	NA
2509	12	6	1	4	2,635	1,398	0,006976189	NM_001107663.1	CUST_7414_PI421866198	NM_001107663	Egfem1	EGF-like and EMI domain containing 1	ENSRNOG00000023908	NA
1642	1734	836	409	543	2,632	1,396	0,016879397	NM_001109405.1	CUST_2076_PI421866198	NM_001109405	Pex10	peroxisomal biogenesis factor 10	ENSRNOG00000024012	680424
4298	26	13	1	10	2,62	1,39	0,020849203	NM_001000504.1	CUST_15083_PI421866198	NM_001000504	Olr37	olfactory receptor 37	ENSRNOG00000018078	308853
1731	19	10	4	8	2,618	1,388	0,03062166	NM_177935.2	CUST_1031_PI421866198	NM_177935	Il1rapl1	interleukin 1 receptor accessory protein-like 1	ENSRNOG00000029663	317553
13298	10	4	1	3	2,617	1,388	0,002836893	NM_001099485.1	CUST_13425_PI421866198	NM_001099485	Vom2r67	vomeronasal 2 receptor, 67	ENSRNOG00000031928	689485
354	38	19	6	14	2,606	1,382	0,024320138	NM_001000409.1	CUST_262_PI421866198	NM_001000409	Olr855	olfactory receptor 855	ENSRNOG00000049373	298468
2916	20	10	2	8	2,603	1,38	0,022487035	NM_001105711.1	CUST_13210_PI421866198	NM_001105711	Grm2	glutamate metabotropic receptor 2	ENSRNOG00000013171	24415
2727	10	4	2	2	2,595	1,376	0,001978757	NM_001105756.1	CUST_13138_PI421866198	NM_001105756	Pbp2	phosphatidylethanolamine binding protein 2	ENSRNOG00000008667	246145
8084	10	4	5	3	2,582	1,369	0,008680986	uc.370-	uc.370-_P1	uc-	NA	NA	NA	NA
12199	117	68	17	58	2,582	1,368	0,045035821	NM_001001080.1	CUST_14719_PI421866198	NM_001001080	Olr1138	olfactory receptor 1138	ENSRNOG00000050182	405970
6672	8	3	1	1	2,577	1,366	0,00011732	NM_001000760.1	CUST_13777_PI421866198	NM_001000760	Olr331	NA	NA	NA
10051	62	36	13	30	2,577	1,366	0,049496979	NM_001109367.1	CUST_2624_PI421866198	NM_001109367	Mageb7	NA	NA	NA
447	27	14	7	10	2,574	1,364	0,033919052	NM_001013067.2	CUST_14674_PI421866198	NM_001013067	Klk1c8	kallikrein 1-related peptidase C8	ENSRNOG00000049279	292866
10747	110	59	4	42	2,573	1,364	0,025801367	NM_001000442.1	CUST_204_PI421866198	NM_001000442	Olr1226	olfactory receptor 1226	ENSRNOG00000048285	300551
2693	29	17	3	15	2,565	1,359	0,042907833	U81826	U81826_P1	U81826	NA	lncRNA	NA	NA
7784	195	96	42	72	2,563	1,358	0,011759672	NM_001024784.2	CUST_13484_PI421866198	NM_001024784	Nfx1	nuclear transcription factor, X-box binding 1	ENSRNOG00000009015	313166
3254	65	36	11	26	2,563	1,358	0,039575787	uc.48+	uc.48+_P1	uc+	NA	lncRNA	NA	NA
3264	491	273	95	216	2,554	1,353	0,036086061	NM_053305.1	CUST_4799_PI421866198	NM_053305	Tas1r1	taste 1 receptor member 1	ENSRNOG00000009708	29407
2677	6	2	3	0	2,55	1,351	0,000975842	NM_019261.1	CUST_5936_PI421866198	NM_019261	Klrc2	NA	NA	NA
8679	60	29	15	21	2,545	1,348	0,008290787	NM_001047926.1	CUST_231_PI421866198	NM_001047926	LOC498236	LRRGT00186	ENSRNOG00000025987	498236
13467	30	16	3	11	2,545	1,348	0,035884147	NM_001008915.1	CUST_1684_PI421866198	NM_001008915	NA	NA	NA	NA
7570	44	25	7	19	2,545	1,348	0,04624328	NM_001134549.1	CUST_6631_PI421866198	NM_001134549	Zzz3	zinc finger, ZZ-type containing 3	ENSRNOG00000050321	310958
3759	80	45	11	35	2,539	1,345	0,042584834	NM_052980.2	CUST_4224_PI421866198	NM_052980	Nr1i2	nuclear receptor subfamily 1, group I, member 2	ENSRNOG00000002906	84385
3161	41	21	6	12	2,537	1,343	0,030633254	MRAK157002	MRAK157002_P1	MRAK157002	NA	lncRNA	NA	NA
20762	16	9	3	6	2,53	1,339	0,021863824	NM_001000865.1	CUST_13018_PI421866198	NM_001000865	NA	NA	NA	NA
3239	20	9	5	5	2,524	1,336	0,010362027	NM_053984.1	CUST_755_PI421866198	NM_053984	Gjb4	gap junction protein, beta 4	ENSRNOG00000026910	117055
2000	14	7	3	6	2,523	1,335	0,024673548	XR_006924	XR_006924_P1	XR_006924	NA	lncRNA	NA	NA
3838	8	4	1	2	2,522	1,334	0,000953593	MRAK154416	MRAK154416_P1	MRAK154416	NA	NA	NA	NA
2153	50	29	9	24	2,506	1,325	0,037864965	NM_173148.1	CUST_3694_PI421866198	NM_173148	Obp2b	odorant binding protein 2B	ENSRNOG00000048417	286933
2752	15	7	3	5	2,501	1,322	0,004270235	NM_017047.1	CUST_13395_PI421866198	NM_017047	Slc10a1	solute carrier family 10 member 1	ENSRNOG00000005794	24777
10068	40	21	8	15	2,493	1,318	0,026594339	NM_001014224.2	CUST_9529_PI421866198	NM_001014224	Tekt5	tektin 5	ENSRNOG00000002571	363538
3232	27	13	7	8	2,492	1,317	0,010851053	NM_001000323.1	CUST_796_PI421866198	NM_001000323	Olr554	olfactory receptor 554	ENSRNOG00000010035	295797
4893	13	8	2	7	2,485	1,313	0,038508398	uc.311-	uc.311-_P1	uc-	NA	lncRNA	NA	NA
20676	118	56	28	36	2,475	1,307	0,006049075	NM_001105969.1	CUST_11646_PI421866198	NM_001105969	Vangl2	VANGL planar cell polarity protein 2	ENSRNOG00000004889	289229
3688	52	27	16	20	2,475	1,307	0,027456983	M18842	M18842_P1	M18842	NA	lncRNA	NA	NA
1983	224	127	51	96	2,467	1,303	0,046522008	MRAK039409	MRAK039409_P1	MRAK039409	NA	lncRNA	NA	NA
15771	72	26	45	13	2,461	1,299	0,03121797	NM_013137.2	CUST_10062_PI421866198	NM_013137	Ddr1	discoidin domain receptor tyrosine kinase 1	ENSRNOG00000057125	25678
39852	404	142	265	63	2,46	1,299	0,047194481	NM_001129880.2	CUST_12262_PI421866198	NM_001129880	Lbh	limb bud and heart development	ENSRNOG00000039902	683626
2724	11	5	2	4	2,457	1,297	0,004870774	MRAK016144	MRAK016144_P1	MRAK016144	NA	lncRNA	NA	NA
4711	16	8	3	6	2,44	1,287	0,016846046	NM_170667.2	CUST_9831_PI421866198	NM_170667	Rln3	relaxin 3	ENSRNOG00000005911	266997
873	220	128	31	94	2,426	1,278	0,045848376	NM_199291.1	CUST_11333_PI421866198	NM_199291	Doxl2	diamine oxidase-like protein 2	ENSRNOG00000032432	312317
10590	450	228	66	141	2,423	1,277	0,013796979	NM_001115040.1	CUST_8252_PI421866198	NM_001115040	LOC683469	similar to RNA polymerase II transcription factor SIII subunit A2 (Elongin A2) (EloA2) (Transcription elongation factor B polypeptide 3B)	ENSRNOG00000039601	683469
3165	10	5	2	4	2,421	1,276	0,013074275	MRAK046413	MRAK046413_P1	MRAK046413	NA	NA	NA	NA
11939	46	21	7	12	2,417	1,273	0,002360232	NM_024354.1	CUST_12409_PI421866198	NM_024354	Chrna4	cholinergic receptor nicotinic alpha 4 subunit	ENSRNOG00000011202	25590
624	73	40	9	29	2,414	1,271	0,029211885	MRAK153872	MRAK153872_P1	MRAK153872	NA	lncRNA	NA	NA
7486	15	9	4	7	2,414	1,272	0,040391753	NM_001000987.1	CUST_12284_PI421866198	NM_001000987	Olr1128	olfactory receptor 1128	ENSRNOG00000029054	405345
2592	6	3	1	1	2,407	1,267	0,000171955	uc.151+	uc.151+_P1	uc+	NA	NA	NA	NA
1152	84	41	41	31	2,405	1,266	0,046170025	XR_006267	XR_006267_P1	XR_006267	NA	lncRNA	NA	NA
43214	10	3	8	1	2,398	1,262	0,026908944	NM_001107003.1	CUST_10610_PI421866198	NM_001107003	Rapgef6	Rap guanine nucleotide exchange factor 6	ENSRNOG00000009995	303141
6159	37	18	7	10	2,397	1,261	0,00443327	MRX99253	MRX99253_P1	MRX99253	NA	lncRNA	NA	NA
4741	13	5	10	2	2,396	1,26	0,043925925	NM_001109376.2	CUST_2569_PI421866198	NM_001109376	Lpgat1	lysophosphatidylglycerol acyltransferase 1	ENSRNOG00000004402	679692
1375	17	8	10	7	2,39	1,257	0,033186841	XR_006715	XR_006715_P1	XR_006715	NA	NA	NA	NA
453	22	12	3	8	2,388	1,256	0,033075262	uc.110-	uc.110-_P1	uc-	NA	lncRNA	NA	NA
4611	14	8	3	5	2,384	1,253	0,0216079	NM_175594.2	CUST_9354_PI421866198	NM_175594	Igf2bp1	insulin-like growth factor 2 mRNA binding protein 1	ENSRNOG00000006122	303477
2671	76	43	5	32	2,38	1,251	0,030014562	NM_001106359.1	CUST_12174_PI421866198	NM_001106359	Wnt8b	wingless-type MMTV integration site family, member 8B	ENSRNOG00000013817	293990
1133	10	5	4	3	2,37	1,245	0,012368231	NM_001000606.1	CUST_14530_PI421866198	NM_001000606	Olr780	olfactory receptor 780	ENSRNOG00000048669	404795
37536	67	26	39	12	2,368	1,244	0,030788894	NM_001166022.1	CUST_7148_PI421866198	NM_001166022	Ddr1	discoidin domain receptor tyrosine kinase 1	ENSRNOG00000057125	25678
3524	13	7	2	5	2,365	1,242	0,017577839	MRAK135260	MRAK135260_P1	MRAK135260	NA	lncRNA	NA	NA
21855	6467	2908	2433	1571	2,362	1,24	0,02633348	NM_153307.1	CUST_5159_PI421866198	NM_153307	NA	NA	NA	NA
5648	43	23	3	15	2,355	1,236	0,021787784	MRuc008pyu	MRuc008pyu_P1	MRuc008pyu	NA	lncRNA	NA	NA
6932	12	6	4	5	2,355	1,236	0,033293279	NM_001008877.1	CUST_2270_PI421866198	NM_001008877	LOC362901	Ac1254	ENSRNOG00000031383	362901
6896	14	8	4	7	2,353	1,235	0,042922444	NM_001108132.1	CUST_7302_PI421866198	NM_001108132	Tbx20	T-box 20	ENSRNOG00000016181	315497
32261	166	67	76	34	2,352	1,234	0,048288051	NM_023989.1	CUST_11830_PI421866198	NM_023989	Senp2	Sumo1/sentrin/SMT3 specific peptidase 2	ENSRNOG00000001773	78973
4768	42	23	4	15	2,351	1,233	0,028715081	NM_001008359.2	CUST_5570_PI421866198	NM_001008359	Spata31a5	SPATA31 subfamily A, member 5	ENSRNOG00000018202	353306
286	7	3	2	0	2,35	1,233	5,91E-05	uc.237+	uc.237+_P1	uc+	NA	NA	NA	NA
2061	105	61	12	43	2,35	1,232	0,043023722	AJ783965	AJ783965_P1	AJ783965	NA	lncRNA	NA	NA
3941	12	6	2	4	2,345	1,23	0,015077772	MRAK136612	MRAK136612_P1	MRAK136612	NA	lncRNA	NA	NA
7181	14	8	2	6	2,34	1,227	0,021532803	uc.410+	uc.410+_P1	uc+	NA	NA	NA	NA
4107	23	12	4	6	2,338	1,226	0,013710915	MRAK089283	MRAK089283_P1	MRAK089283	NA	lncRNA	NA	NA
7416	14	7	2	5	2,337	1,225	0,02278068	NM_001106864.1	CUST_8873_PI421866198	NM_001106864	Ltf	lactotransferrin	ENSRNOG00000031779	301034
11686	123	74	14	61	2,334	1,223	0,040697487	NM_001012221.1	CUST_1216_PI421866198	NM_001012221	Oxct2a	NA	NA	NA
4359	2205	1224	308	808	2,327	1,218	0,029402872	MRAK010033	MRAK010033_P1	MRAK010033	NA	lncRNA	NA	NA
2748	10	4	2	2	2,324	1,217	0,001376518	NM_001000545.1	CUST_15036_PI421866198	NM_001000545	Olr145	olfactory receptor 145	ENSRNOG00000048582	365331
10237	302	167	46	113	2,322	1,216	0,027148187	NM_144753.2	CUST_7983_PI421866198	NM_144753	Fev	FEV, ETS transcription factor	ENSRNOG00000017856	NA
7411	13	8	3	6	2,32	1,214	0,045241247	NM_080773.1	CUST_472_PI421866198	NM_080773	Chrm1	cholinergic receptor, muscarinic 1	ENSRNOG00000018385	25229
472	14	8	2	5	2,316	1,211	0,02590348	MRAK084123	MRAK084123_P1	MRAK084123	NA	lncRNA	NA	NA
1378	7	3	2	1	2,315	1,211	0,000361736	NM_001044274.1	CUST_14184_PI421866198	NM_001044274	Odam	odontogenic, ameloblast asssociated	ENSRNOG00000023372	641555

10797	1165	570	399	265	2,312	1,209	0,023370051	NM_001012218.1	CUST_1238_PI421866198	NM_001012218	Trim55	tripartite motif-containing 55	ENSRNOG00000012723	365751
2841	35	21	4	15	2,311	1,208	0,046656584	NM_001106995.1	CUST_8316_PI421866198	NM_001106995	Nipal4	NIPA-like domain containing 4	ENSRNOG00000006255	303070
15163	31	17	4	9	2,309	1,207	0,04164942	NM_001000050.1	CUST_2612_PI421866198	NM_001000050	Olr1557	olfactory receptor 1557	ENSRNOG00000046622	288190
16547	39	18	13	8	2,308	1,207	0,007129978	NM_001015017.1	CUST_7987_PI421866198	NM_001015017	Olfm2	olfactomedin 2	ENSRNOG00000020519	313783
7440	31	15	10	9	2,308	1,206	0,009738569	XR_008018	XR_008018_P1	XR_008018	NA	lncRNA	NA	NA
3371	63	31	10	15	2,305	1,205	0,004229318	MRAK137812	MRAK137812_P1	MRAK137812	NA	lncRNA	NA	NA
2758	18	10	3	8	2,297	1,2	0,035457874	NM_001109554.1	CUST_1283_PI421866198	NM_001109554	Gpr21	G protein-coupled receptor 21	ENSRNOG00000042269	689917
890	7	3	3	1	2,296	1,199	0,001497102	uc.90+	uc.90+_P1	uc+	NA	NA	NA	NA
31915	9	4	4	3	2,295	1,198	0,034528745	NM_001108350.1	CUST_6109_PI421866198	NM_001108350	Klhdc9	kelch domain containing 9	ENSRNOG00000004022	360878
20757	15	7	5	4	2,294	1,198	0,019059158	NM_022617.1	CUST_1905_PI421866198	NM_022617	Mpeg1	NA	NA	NA
9226	191	109	30	80	2,289	1,195	0,031916613	NM_001109261.1	CUST_3164_PI421866198	NM_001109261	Pax5	paired box 5	ENSRNOG00000024729	500453
294	16	10	1	8	2,288	1,194	0,043272419	XR_006847	XR_006847_P1	XR_006847	NA	lncRNA	NA	NA
11426	12	6	1	4	2,287	1,194	0,009129508	XR_008925	XR_008925_P1	XR_008925	NA	lncRNA	NA	NA
370	33	17	6	11	2,286	1,193	0,017638821	NM_001009501.2	CUST_980_PI421866198	NM_001009501	Ly49i5	Ly49 inhibitory receptor 5	ENSRNOG00000051661	494211
11765	27	12	4	4	2,282	1,191	0,000141895	NM_001037534.1	CUST_11125_PI421866198	NM_001037534	Defb17	defensin beta 17	ENSRNOG00000039640	641658
8750	3328	1357	2461	714	2,282	1,191	0,026970654	XR_007411	XR_007411_P1	XR_007411	NA	lncRNA	NA	NA
4863	31	15	12	8	2,274	1,185	0,028841178	uc.407+	uc.407+_P1	uc+	NA	lncRNA	NA	NA
8080	38	16	24	8	2,27	1,183	0,033771372	XR_007100	XR_007100_P1	XR_007100	NA	lncRNA	NA	NA
14305	22	10	7	4	2,263	1,178	0,004544738	NM_001109342.1	CUST_2723_PI421866198	NM_001109342	Pramel6	preferentially expressed antigen in melanoma-like 6	ENSRNOG00000021920	502648
181	17	9	5	6	2,254	1,172	0,03138569	uc.200+	uc.200+_P1	uc+	NA	lncRNA	NA	NA
4070	445	229	201	156	2,249	1,169	0,042940214	BC169115	BC169115_P1	BC169115	NA	lncRNA	NA	NA
1043	12	6	3	3	2,246	1,167	0,001357298	NM_001134495.1	CUST_7137_PI421866198	NM_001134495	Gsdmc	gasdermin C	ENSRNOG00000054576	299908
28626	26219	11482	16057	6287	2,246	1,168	0,039554888	NM_017027.1	CUST_13486_PI421866198	NM_017027	Mpz	myelin protein zero	ENSRNOG00000003171	24564
3889	89	47	6	25	2,244	1,166	0,01485284	MRAK138051	MRAK138051_P1	MRAK138051	NA	lncRNA	NA	NA
5146	12	7	2	6	2,236	1,161	0,04571537	NM_001099486.1	CUST_13423_PI421866198	NM_001099486	Vom2r73	vomeronasal 2 receptor, 73	ENSRNOG00000047358	689072
4301	80	34	40	18	2,233	1,159	0,032697383	NM_031780.1	CUST_10545_PI421866198	NM_031780	Apba2	amyloid beta precursor protein binding family A member 2	ENSRNOG00000016358	83610
14487	74	36	7	14	2,231	1,158	0,004163902	NM_198727.1	CUST_11742_PI421866198	NM_198727	Tmem132d	transmembrane protein 132D	ENSRNOG00000008447	288750
4946	5	2	2	0	2,228	1,156	0,000644833	uc.438-	uc.438-_P1	uc-	NA	NA	NA	NA
1935	772	356	350	178	2,224	1,153	0,024895273	NM_134359.1	CUST_4267_PI421866198	NM_134359	Ppp4c	protein phosphatase 4, catalytic subunit	ENSRNOG00000019813	171366
12962	128	75	13	50	2,224	1,153	0,043183437	uc.16+	uc.16+_P1	uc+	NA	lncRNA	NA	NA
4698	13	7	1	5	2,222	1,152	0,027793345	NM_012585.1	CUST_10976_PI421866198	NM_012585	Htr1a	5-hydroxytryptamine receptor 1A	ENSRNOG00000010254	24473
1815	154	87	17	59	2,22	1,151	0,0240894	NM_001077675.1	CUST_6164_PI421866198	NM_001077675	Trim72	tripartite motif containing 72	ENSRNOG00000022099	365377
6152	6	3	2	1	2,212	1,146	0,007103845	NM_001107388.1	CUST_8593_PI421866198	NM_001107388	NA	NA	NA	NA
9023	33	20	6	15	2,212	1,145	0,039034668	NM_001009488.1	CUST_1443_PI421866198	NM_001009488	Ly49s6	Ly49 stimulatory receptor 6	ENSRNOG00000053337	494196
3922	49	28	8	18	2,206	1,142	0,029085298	MRAK090254	MRAK090254_P1	MRAK090254	NA	lncRNA	NA	NA
5308	6	2	2	1	2,202	1,139	0,004167009	NM_001099517.1	CUST_12935_PI421866198	NM_001099517	Vom2r72	NA	NA	NA
4110	94	55	8	32	2,201	1,138	0,045754396	BC059119	BC059119_P1	BC059119	NA	lncRNA	NA	NA
6791	194	77	115	21	2,2	1,137	0,019293987	NM_030993.1	CUST_12897_PI421866198	NM_030993	Ddn	dendrin	ENSRNOG00000059605	25113
9219	24	13	3	7	2,195	1,134	0,026583859	NM_001105904.1	CUST_11796_PI421866198	NM_001105904	Cd209a	CD209a molecule	ENSRNOG00000040128	288375
1141	16	9	3	5	2,19	1,131	0,009482912	MRAK172199	MRAK172199_P1	MRAK172199	NA	lncRNA	NA	NA
11081	43	25	6	16	2,182	1,125	0,037879911	NM_020106.1	CUST_11159_PI421866198	NM_020106	Olr414	olfactory receptor 414	ENSRNOG00000033799	56821
9615	39	23	8	16	2,18	1,124	0,046809389	NM_001012749.1	CUST_13712_PI421866198	NM_001012749	Ly49s5	NA	NA	NA
5627	29	17	2	10	2,179	1,124	0,027947693	uc.145-	uc.145-_P1	uc-	NA	lncRNA	NA	NA
5552	27	17	3	11	2,172	1,119	0,046505486	uc.353+	uc.353+_P1	uc+	NA	lncRNA	NA	NA
954	21	12	5	9	2,17	1,118	0,03587999	MRAK081740	MRAK081740_P1	MRAK081740	NA	lncRNA	NA	NA
2070	19	8	9	3	2,166	1,115	0,024440555	DQ294402	DQ294402_P1	DQ294402	NA	lncRNA	NA	NA
1803	9	6	1	4	2,161	1,112	0,023931184	NM_001000953.1	CUST_12379_PI421866198	NM_001000953	Olr1352	olfactory receptor 1352	ENSRNOG00000037404	405301
9392	11	7	2	6	2,156	1,108	0,049765462	NM_145083.1	CUST_8576_PI421866198	NM_145083	Lrrc15	leucine rich repeat containing 15	ENSRNOG00000038539	246296
4093	15	7	2	3	2,152	1,106	0,001136574	NM_001106976.1	CUST_8335_PI421866198	NM_001106976	Ppl	periplakin	ENSRNOG00000002930	302934
20934	25	15	7	12	2,15	1,104	0,048198773	NM_001000900.1	CUST_12492_PI421866198	NM_001000900	Olr830	olfactory receptor 830	ENSRNOG00000013367	405210
6340	54	27	25	15	2,149	1,104	0,043214657	NM_001112713.1	CUST_14309_PI421866198	NM_001112713	Grk6	G protein-coupled receptor kinase 6	ENSRNOG00000014615	59076
11339	80	43	16	22	2,145	1,101	0,013127637	NM_214833.1	CUST_4175_PI421866198	NM_214833	Olr1385	olfactory receptor 1385	ENSRNOG00000046800	287236
38506	381	153	238	52	2,145	1,101	0,049071149	NM_017216.1	CUST_12135_PI421866198	NM_017216	Slc3a1	solute carrier family 3 member 1	ENSRNOG00000007006	29484
12200	118	74	14	59	2,144	1,1	0,043711695	NM_173301.1	CUST_2598_PI421866198	NM_173301	LOC286960	preprotrypsinogen IV	ENSRNOG00000013245	286960
2596	7	3	1	1	2,142	1,099	0,000932523	uc.388+	uc.388+_P1	uc+	NA	NA	NA	NA
7767	16	8	4	3	2,127	1,089	0,005542324	NM_022627.1	CUST_1860_PI421866198	NM_022627	Prkab2	protein kinase AMP-activated non-catalytic subunit beta 2	ENSRNOG00000018166	64562
2667	7	3	3	1	2,126	1,088	0,005610129	NM_001000878.1	CUST_13001_PI421866198	NM_001000878	Olr1135	olfactory receptor 1135	ENSRNOG00000049064	405177
7924	10	6	2	4	2,124	1,087	0,036037492	NM_031761.1	CUST_10603_PI421866198	NM_031761	Vegfd	vascular endothelial growth factor D	ENSRNOG00000003587	360457
2816	9	5	1	3	2,122	1,085	0,016220086	XR_007063	XR_007063_P1	XR_007063	NA	NA	NA	NA
11051	14	9	2	6	2,122	1,085	0,034920563	NM_001044271.1	CUST_14186_PI421866198	NM_001044271	Prl2c1	Prolactin family 2, subfamily c, member 1	ENSRNOG00000039058	502122
6419	5	3	1	1	2,121	1,085	2,31E-05	NM_022227.1	CUST_4511_PI421866198	NM_022227	Asic5	acid sensing ion channel subunit family member 5	ENSRNOG00000011842	63866
5543	8	4	2	1	2,12	1,084	0,000834417	MRAK158695	MRAK158695_P1	MRAK158695	NA	NA	NA	NA
4933	11	5	2	1	2,118	1,083	0,000120036	MRAK089684	MRAK089684_P1	MRAK089684	NA	lncRNA	NA	NA
4353	43	25	4	15	2,116	1,081	0,033439546	MRuc007jeg	MRuc007jeg_P1	MRuc007jeg	NA	lncRNA	NA	NA
3347	19	9	6	4	2,112	1,078	0,005433642	NM_001106399.1	CUST_12023_PI421866198	NM_001106399	Spata9	spermatogenesis associated 9	ENSRNOG00000012770	294594
1546	11	7	3	5	2,11	1,077	0,032800848	MRuc009cbo	MRuc009cbo_P1	MRuc009cbo	NA	lncRNA	NA	NA
7402	8	4	3	2	2,109	1,076	0,01890593	uc.158+	uc.158+_P1	uc+	NA	NA	NA	NA
6680	11	7	2	5	2,104	1,073	0,04492981	uc.39-	uc.39-_P1	uc-	NA	NA	NA	NA
2659	34	18	5	8	2,1	1,07	0,005701189	NM_001008943.1	CUST_1587_PI421866198	NM_001008943	NA	NA	NA	NA
26630	18	8	6	3	2,1	1,071	0,013718215	XR_005427	XR_005427_P1	XR_005427	NA	lncRNA	NA	NA
9788	10	6	2	4	2,094	1,067	0,028134679	MRBC019958	MRBC019958_P1	MRBC019958	NA	lncRNA	NA	NA
8629	66	40	4	30	2,091	1,065	0,035574725	NM_001107198.1	CUST_9586_PI421866198	NM_001107198	Exo1	exonuclease 1	ENSRNOG00000056209	305000
10124	1056	569	357	325	2,09	1,063	0,048362587	NM_001009710.1	CUST_15172_PI421866198	NM_001009710	Lrrc41	leucine rich repeat containing 41	ENSRNOG00000013642	362566
3490	56	31	10	17	2,089	1,063	0,016277678	NM_001047873.1	CUST_642_PI421866198	NM_001047873	Cntnap5b	contactin associated protein-like 5B	ENSRNOG00000032206	301650
5790	36	21	10	13	2,089	1,063	0,039500333	NM_017233.1	CUST_12065_PI421866198	NM_017233	Hpd	4-hydroxyphenylpyruvate dioxygenase	ENSRNOG00000001338	29531
1900	48	30	8	20	2,079	1,056	0,04659043	uc.132-	uc.132-_P1	uc-	NA	lncRNA	NA	NA
941	15	9	4	6	2,077	1,054	0,03654322	XR_005808	XR_005808_P1	XR_005808	NA	lncRNA	NA	NA
11004	20	12	1	8	2,072	1,051	0,030208309	uc.233+	uc.233+_P1	uc+	NA	lncRNA	NA	NA
9975	9	5	2	4	2,071	1,051	0,027210908	uc.205+	uc.205+_P1	uc+	NA	NA	NA	NA
3514	80	47	7	30	2,067	1,047	0,023078281	XR_006577	XR_006577_P1	XR_006577	NA	lncRNA	NA	NA
3174	47	24	10	11	2,064	1,045	0,002749203	MRAK084995	MRAK084995_P1	MRAK084995	NA	lncRNA	NA	NA
14	36	20	6	10	2,062	1,044	0,01435384	NM_001012115.1	CUST_1884_PI421866198	NM_001012115	Creb3l3	cAMP responsive element binding protein 3-like 3	ENSRNOG00000032202	314638
5141	104	61	13	34	2,062	1,044	0,040488932	MRAK036969	MRAK036969_P1	MRAK036969	NA	lncRNA	NA	NA
11818	40	22	17	13	2,052	1,037	0,030259175	NM_001108345.1	CUST_6114_PI421866198	NM_001108345	NA	NA	NA	NA
11593	6	3	1	2	2,049	1,035	0,007914346	NM_001000228.1	CUST_1340_PI421866198	NM_001000228	Olr267	olfactory receptor 267	ENSRNOG00000049197	293435
7450	296	138	147	58	2,045	1,032	0,027447869	NM_001107077.2	CUST_10405_PI421866198	NM_001107077	Fn3krp	fructosamine-3-kinase-related protein	ENSRNOG00000036660	303755
1307	70	41	8	26	2,037	1,027	0,023176372	D26502	D26502_P1	D26502	NA	lncRNA	NA	NA
4606	34	20	3	14	2,033	1,024	0,025969862	XR_006296	XR_006296_P1	XR_006296	NA	lncRNA	NA	NA
8106	34	20	5	11	2,032	1,023	0,021121564	MRAK149681	MRAK149681_P1	MRAK149681	NA	lncRNA	NA	NA
7175	121	67	27	31	2,031	1,022	0,024318192	MRAK157559	MRAK157559_P1	MRAK157559	NA	lncRNA	NA	NA
8364	52	30	5	14	2,03	1,022	0,031063084	L11036	L11036_P1	L11036	NA	lncRNA	NA	NA
6056	501	256	183	113	2,026	1,019	0,010959238	XR_006319	XR_006319_P1	XR_006319	NA	lncRNA	NA	NA
2761	26	15	7	9	2,026	1,019	0,03228481	MRAK044409	MRAK044409_P1	MRAK044409	NA	lncRNA	NA	NA
30675	390	189	233	91	2,026	1,019	0,032530345	XR_009256	XR_009256_P1	XR_009256	NA	lncRNA	NA	NA
27421	25	10	17	2	2,023	1,017	0,03765006	NM_001009502.1	CUST_979_PI421866198	NM_001009502	Eogt	EGF domain specific O-linked N-acetylglucosamine transferase	ENSRNOG00000024533	494219
5387	71	43	8	28	2,022	1,016	0,035095725	uc.17+	uc.17+_P1	uc+	NA	lncRNA	NA	NA
9554	51	28	4	15	2,02	1,014	0,007928991	NM_001108706.1	CUST_3592_PI421866198	NM_001108706	Efcab10	EF-hand calcium binding domain 10	ENSRNOG00000010730	NA
1386	7	3	4	0	2,015	1,011	0,007868201	MRuc007xsf	MRuc007xsf_P1	MRuc007xsf	NA	NA	NA	NA
2063	13	8	2	5	2,012	1,009	0,036150923	AJ286314	AJ286314_P1	AJ286314	NA	lncRNA	NA	NA
1393	54	32	7	22	2,011	1,008	0,024299375	MRAK138874	MRAK138874_P1	MRAK138874	NA	lncRNA	NA	NA
5137	23	15	3	10	2,011	1,008	0,04423061	BC092589	BC092589_P1	BC092589	NA	lncRNA	NA	NA
7944	17	10	3	6	2,008	1,006	0,01484136	NM_001134884.1	CUST_4698_PI421866198	NM_001134884	Scube1	signal peptide, CUB domain and EGF like domain containing 1	ENSRNOG00000010617	315174
7779	50	30	12	21	2,008	1,006	0,045109025	NM_199497.1	CUST_9911_PI421866198	NM_199497	Slc4a8	solute carrier family 4 member 8	ENSRNOG00000028879	315311
20409	8	5	1	3	2	1	0,014262903	uc.303+	uc.303+_P1	uc+	NA	NA	NA	NA
13449	12	6	4	3	2	1	0,018869067	NM_001105896.1	CUST_12412_PI421866198	NM_001105896	Krtap15-1	keratin associated protein 15-1	ENSRNOG00000049399	288297
3947	6	2	5	0	1,995	0,997	0,045235602	NM_001000328.1	CUST_789_PI421866198	NM_001000328	Olr576	olfactory receptor 576	ENSRNOG00000047926	295814
7234	42	21	17	7	1,993	0,995	0,009078142	NM_001109605.1	CUST_886_PI421866198	NM_001109605	LOC690617	hypothetical protein LOC690617	ENSRNOG00000039861	690617
4376	14	7	5	2	1,992	0,994	0,002063915	AJ286149	AJ286149_P1	AJ286149	NA	lncRNA	NA	NA
32533	160	78	69	34	1,99	0,993	0,030487317	NM_053842.1	CUST_1441_PI421866198	NM_053842	Mapk1	mitogen activated protein kinase 1	ENSRNOG00000001849	116590
5120	35	18	11	8	1,986	0,99	0,012915501	MRBC028667	MRBC028667_P1	MRBC028667	NA	lncRNA	NA	NA
1241	12	7	3	5	1,984	0,989	0,046209042	NM_021839.1	CUST_4119_PI421866198	NM_021839	Hist1h2aa	histone cluster 1 H2A family member a	ENSRNOG00000048122	24828
9564	232	140	19	91	1,982	0,987	0,021240206	NM_001047923.1	CUST_237_PI421866198	NM_001047923	LOC498152	hypothetical protein LOC498152	ENSRNOG00000030354	498152
6763	14	8	2	4	1,977	0,983	0,008105118	MRAK048492	MRAK048492_P1	MRAK048492	NA	lncRNA	NA	NA
7407	1028	471	652	137	1,975	0,982	0,02474782	NM_001135008.1	CUST_6402_PI421866198	NM_001135008	Pabpn1	poly(A) binding protein, nuclear 1	ENSRNOG00000042195	116697
2077	16	10	2	5	1,975	0,982	0,026147227	MRAK051515	MRAK051515_P1	MRAK051515	NA	lncRNA	NA	NA
10732	48	26	13	12	1,974	0,981	0,009455192	NM_031124.1	CUST_14821_PI421866198	NM_031124	Stx3	syntaxin 3	ENSRNOG00000021013	81802
1296	5	2	4	0	1,969	0,977	0,020476788	NM_001106207.1	CUST_12992_PI421866198	NM_001106207	Myct1	myc target 1	ENSRNOG00000018858	292264
11178	17	10	1	6	1,967	0,976	0,022768874	NM_001107040.1	CUST_10502_PI421866198	NM_001107040	Dlx4	distal-less homeobox 4	ENSRNOG00000004399	303469
6814	14	8	2	5	1,965	0,974	0,011971839	MRAK015499	MRAK015499_P1	MRAK015499	NA	lncRNA	NA	NA
13541	6	3	3	1	1,964	0,974	0,027496137	NM_001000913.1	CUST_12469_PI421866198	NM_001000913	Olr1587	olfactory receptor 1587	ENSRNOG00000050941	405224
5050	21	12	3	5	1,96	0,971	0,005245936	MRAK085669	MRAK085669_P1	MRAK085669	NA	lncRNA	NA	NA
6767	5	3	1	1	1,958	0,969	0,000906848	NM_022544.2	CUST_2502_PI421866198	NM_022544	Defb4	defensin beta 4	ENSRNOG00000013939	64389
4547	9	4	3	2	1,958	0,969	0,032151947	NM_023993.1	CUST_11808_PI421866198	NM_023993	Tas2r119	taste receptor, type 2, member 119	ENSRNOG00000011963	78979
11338	41	22	8	10	1,954	0,967	0,00719015	NM_001106178.1	CUST_13493_PI421866198	NM_001106178	Exoc3l1	exocyst complex component 3-like 1	ENSRNOG00000015641	291961
9977	24	13	4	7	1,95	0,963	0,006825996	XR_007761	XR_007761_P1	XR_007761	NA	lncRNA	NA	NA
3828	74	38	30	14	1,945	0,96	0,011096075	NM_080785.2	CUST_446_PI421866198	NM_080785	Manea	mannosidase, endo-alpha	ENSRNOG00000008626	140808
8699	51	28	12	14	1,943	0,958	0,009967399	NM_001005549.1	CUST_14047_PI421866198	NM_001005549	Tinag	tubulointerstitial nephritis antigen	ENSRNOG00000058120	NA
11428	7	5	2	4	1,941	0,957	0,033515831	NM_001000423.1	CUST_238_PI421866198	NM_001000423	Olr1106	olfactory receptor 1106	ENSRNOG00000033022	300199
11852	7	4	3	1	1,937	0,954	0,03778744	AY598353	AY598353_P1	AY598353	NA	NA	NA	NA
5989	92	56	9	33	1,936	0,953	0,032005305	AJ286820	AJ286820_P1	AJ286820	NA	lncRNA	NA	NA
16950	1389	719	705	339	1,931	0,95	0,032056477	XR_006513	XR_006513_P1	XR_006513	NA	lncRNA	NA	NA
42326	6	3	2	1	1,928	0,947	0,018753564	NM_001012360.1	CUST_544_PI421866198	NM_001012360	Fut10	fucosyltransferase 10	ENSRNOG00000050857	497619
2674	5	3	1	1	1,924	0,944	0,002481165	MRAK053878	MRAK053878_P1	MRAK053878	NA	NA	NA	NA
1486	9	6	2	3	1,921	0,942	0,034872597	Y09174	Y09174_P1	Y09174	NA	NA	NA	NA
2423	11	6	2	2	1,92	0,941	0,00470769	MRNR_002839	MRNR_002839_P1	MRNR_002839	NA	lncRNA	NA	NA
41	5	3	2	1	1,917	0,939	0,003404768	NM_001000728.1	CUST_13850_PI421866198	NM_001000728	Olr1541	olfactory receptor 1541	ENSRNOG00000029104	404983
9641	86	53	11	34	1,917	0,939	0,031146791	NM_001005896.1	CUST_11753_PI421866198	NM_001005896	Clec4b2	C-type lectin domain family 4, member B2	ENSRNOG00000027655	450222
19892	11	6	5	2	1,914	0,937	0,010839303	XR_006463	XR_006463_P1	XR_006463	NA	lncRNA	NA	NA
2629	9	5	5	2	1,91	0,933	0,027347502	MRuc009lbc	MRuc009lbc_P1	MRuc009lbc	NA	NA	NA	NA
2332	16	9	2	3	1,909	0,933	0,003494862	NM_145087.1	CUST_8566_PI421866198	NM_145087	Spag11a	sperm associated antigen 11A	ENSRNOG00000013957	246305
22118	106	56	40	25	1,907	0,931	0,023344247	NM_001135718.1	CUST_1825_PI421866198	NM_001135718	Prex1	phosphatidylinositol-3,4,5-trisphosphate-dependent Rac exchange factor 1	ENSRNOG00000006952	311647
22213	40	23	5	11	1,899	0,925	0,005907828	NM_001105802.1	CUST_12574_PI421866198	NM_001105802	Dhx33	DEAH-box helicase 33	ENSRNOG00000007054	287464
6929	85	54	9	34	1,892	0,92	0,034633579	NM_031839.3	CUST_9857_PI421866198	NM_031839	Cyp2c23	cytochrome P450, family 2, subfamily c, polypeptide 23	ENSRNOG00000013291	83790
545	19	12	2	7	1,891	0,919	0,037702566	XR_007517	XR_007517_P1	XR_007517	NA	lncRNA	NA	NA
5216	665	422	67	241	1,885	0,914	0,042116444	NM_022284.2	CUST_4379_PI421866198	NM_022284	Guca2b	guanylate cyclase activator 2B	ENSRNOG00000008979	64055
3411	207	135	25	92	1,878	0,909	0,047785864	MRAK090145	MRAK090145_P1	MRAK090145	NA	lncRNA	NA	NA
4748	643	389	174	227	1,876	0,908	0,045328106	NM_001009714.1	CUST_15167_PI421866198	NM_001009714	Mitd1	microtubule interacting and trafficking domain containing 1	ENSRNOG00000018467	363219
3063	9	5	2	2	1,875	0,907	0,021056691	MRAK158356	MRAK158356_P1	MRAK158356	NA	lncRNA	NA	NA
4705	29	16	5	4	1,874	0,906	0,000320757	NM_182675.1	CUST_9724_PI421866198	NM_182675	Rab40c	Rab40c, member RAS oncogene family	ENSRNOG00000020067	359728
3842	42	27	3	17	1,871	0,904	0,024117278	NM_001009492.1	CUST_1417_PI421866198	NM_001009492	Oas1e	2-5 oligoadenylate synthetase 1E	ENSRNOG00000031726	494201
18232	21	12	3	4	1,868	0,902	0,007074006	NM_031710.1	CUST_10699_PI421866198	NM_031710	Olr226	olfactory receptor 226	ENSRNOG00000029142	100911376
3581	266	169	30	106	1,868	0,902	0,032559142	EF061226	EF061226_P1	EF061226	NA	lncRNA	NA	NA
5108	306	178	41	75	1,867	0,901	0,008188649	MRAK002255	MRAK002255_P1	MRAK002255	NA	lncRNA	NA	NA
1508	1908	1055	531	424	1,867	0,901	0,009372405	XR_006417	XR_006417_P1	XR_006417	NA	lncRNA	NA	NA
11089	6	4	1	2	1,866	0,9	0,021975498	NM_001029922.1	CUST_11129_PI421866198	NM_001029922	Zbbx	zinc finger, B-box domain containing	ENSRNOG00000009792	361964
3086	114	67	12	32	1,865	0,899	0,00890711	MRAK158258	MRAK158258_P1	MRAK158258	NA	lncRNA	NA	NA
793	107	63	14	30	1,863	0,898	0,011988423	NM_023967.1	CUST_11869_PI421866198	NM_023967	Gfra4	GDNF family receptor alpha 4	ENSRNOG00000021241	66023
15337	23	12	5	4	1,862	0,897	0,003229402	NM_001025061.1	CUST_14601_PI421866198	NM_001025061	Tas2r143	taste receptor, type 2, member 143	ENSRNOG00000053213	103690198
1384	57	35	8	20	1,862	0,896	0,025530763	NM_001109132.1	CUST_3909_PI421866198	NM_001109132	Marveld3	MARVEL domain containing 3	ENSRNOG00000016291	498950
12275	78	46	6	22	1,858	0,894	0,007185288	NM_001105860.1	CUST_12486_PI421866198	NM_001105860	Mzt2b	mitotic spindle organizing protein 2B	ENSRNOG00000001834	287929
885	12	7	2	4	1,858	0,894	0,022127985	NM_001000040.1	CUST_2660_PI421866198	NM_001000040	Olr1522	olfactory receptor 1522	ENSRNOG00000030148	404962
16364	38	19	17	4	1,854	0,89	0,011199716	MRAK049911	MRAK049911_P1	MRAK049911	NA	lncRNA	NA	NA
1720	17	11	2	6	1,852	0,889	0,049455327	MRBC006183	MRBC006183_P1	MRBC006183	NA	lncRNA	NA	NA
8375	150	83	59	37	1,85	0,888	0,019755347	NM_053886.1	CUST_1297_PI421866198	NM_053886	Lman1	lectin, mannose-binding, 1	ENSRNOG00000024470	116666
6615	8163	4275	3576	1455	1,85	0,887	0,021002364	XR_008096	XR_008096_P1	XR_008096	NA	lncRNA	NA	NA
1536	70	33	44	9	1,85	0,888	0,040756444	MRAK079955	MRAK079955_P1	MRAK079955	NA	lncRNA	NA	NA
2089	6	3	1	1	1,849	0,887	0,00250849	AY169303	AY169303_P1	AY169303	NA	NA	NA	NA
6510	32	21	4	13	1,848	0,886	0,0474699	NM_019312.2	CUST_5399_PI421866198	NM_019312	Itpkb	inositol-trisphosphate 3-kinase B	ENSRNOG00000002969	54260
2059	792	427	329	155	1,847	0,885	0,029538372	NM_024146.1	CUST_13843_PI421866198	NM_024146	Fgfr1	Fibroblast growth factor receptor 1	ENSRNOG00000016050	79114
14483	104	61	12	24	1,844	0,883	0,011165286	NM_001107871.1	CUST_6275_PI421866198	NM_001107871	Fbxo41	F-box protein 41	ENSRNOG00000033202	312504
19123	5943	3031	3082	992	1,843	0,882	0,025297312	XR_005519	XR_005519_P1	XR_005519	NA	lncRNA	NA	NA
8085	72	47	6	28	1,843	0,882	0,047620789	NM_001034144.1	CUST_8081_PI421866198	NM_001034144	Nkx3-1	NK3 homeobox 1	ENSRNOG00000015477	305999
5111	1738	879	884	279	1,841	0,88	0,035579803	NM_012673.1	CUST_10249_PI421866198	NM_012673	Thy1	Thy-1 cell surface antigen	ENSRNOG00000006604	24832
1313	7	4	2	2	1,836	0,876	0,030230961	NM_138977.1	CUST_2914_PI421866198	NM_138977	Prokr1	prokineticin receptor 1	ENSRNOG00000009556	192648
5736	9	5	1	2	1,835	0,876	0,014426009	NM_001000975.1	CUST_12326_PI421866198	NM_001000975	Olr810	olfactory receptor 810	ENSRNOG00000005398	405330
15250	50	30	8	12	1,834	0,875	0,026651907	NM_013033.2	CUST_10820_PI421866198	NM_013033	Slc5a1	solute carrier family 5 member 1	ENSRNOG00000017775	25552
17	115	72	11	33	1,831	0,872	0,036726049	MRAK007077	MRAK007077_P1	MRAK007077	NA	lncRNA	NA	NA
1282	5	3	3	1	1,825	0,868	0,041919021	AJ286228	AJ286228_P1	AJ286228	NA	NA	NA	NA
5205	8	5	2	2	1,822	0,866	0,018645969	NM_001105850.1	CUST_12500_PI421866198	NM_001105850	Ush1g	Usher syndrome 1G	ENSRNOG00000028456	287819
10654	50	31	5	16	1,821	0,865	0,029322431	NM_001107370.1	CUST_8623_PI421866198	NM_001107370	Cd226	CD226 molecule	ENSRNOG00000038197	307199
14653	56	33	8	14	1,819	0,864	0,010592669	NM_001037514.1	CUST_11177_PI421866198	NM_001037514	Defb12	defensin beta 12	ENSRNOG00000038155	641643
1415	5	3	2	1	1,819	0,863	0,015500446	NM_031347.1	CUST_13315_PI421866198	NM_031347	Ppargc1a	PPARG coactivator 1 alpha	ENSRNOG00000004473	83516
35626	454	229	247	46	1,818	0,862	0,019064575	NM_001105843.1	CUST_12516_PI421866198	NM_001105843	Lsm12	LSM12 homolog	ENSRNOG00000020894	287731
7334	41	25	4	13	1,817	0,862	0,01239295	MRAK044617	MRAK044617_P1	MRAK044617	NA	lncRNA	NA	NA
9656	269	167	51	92	1,817	0,862	0,031545769	NM_001037632.1	CUST_10453_PI421866198	NM_001037632	Sp7	Sp7 transcription factor	ENSRNOG00000014082	300260
710	107	62	27	26	1,815	0,86	0,012474039	NM_001098793.1	CUST_15042_PI421866198	NM_001098793	Prkrip1	Prkr interacting protein 1 (IL11 inducible)	ENSRNOG00000001426	498171
6397	76	49	8	26	1,815	0,86	0,039954392	NM_001107142.1	CUST_9721_PI421866198	NM_001107142	Anapc7	anaphase promoting complex subunit 7	ENSRNOG00000001283	304490
6335	17	10	3	5	1,813	0,859	0,014136149	uc.253+	uc.253+_P1	uc+	NA	lncRNA	NA	NA
26078	2047	1156	787	500	1,812	0,857	0,045862147	NM_001033066.1	CUST_11810_PI421866198	NM_001033066	Ddhd1	DDHD domain containing 1	ENSRNOG00000009481	305816
1811	6	4	3	2	1,81	0,856	0,039049088	MRuc008lpy	MRuc008lpy_P1	MRuc008lpy	NA	lncRNA	NA	NA
5114	18	12	2	6	1,809	0,855	0,045145455	AY660012	AY660012_P1	AY660012	NA	lncRNA	NA	NA
7982	55	34	6	15	1,807	0,854	0,021750013	XR_005716	XR_005716_P1	XR_005716	NA	lncRNA	NA	NA
13222	40	24	5	12	1,806	0,852	0,018529478	NM_001113183.1	CUST_14516_PI421866198	NM_001113183	Tesb	testis specific basic protein	ENSRNOG00000039398	414826
6410	131	81	32	37	1,804	0,851	0,044890951	NM_022927.1	CUST_184_PI421866198	NM_022927	Mid1	midline 1	ENSRNOG00000003613	54252
13548	43	24	5	6	1,803	0,85	0,000383899	MRAK142643	MRAK142643_P1	MRAK142643	NA	lncRNA	NA	NA
2975	194	106	89	42	1,801	0,849	0,035868128	XR_005977	XR_005977_P1	XR_005977	NA	lncRNA	NA	NA
5546	580	317	209	90	1,799	0,847	0,007885382	MRAK135480	MRAK135480_P1	MRAK135480	NA	lncRNA	NA	NA
128	5	2	3	0	1,799	0,847	0,029062168	NM_053551.1	CUST_3284_PI421866198	NM_053551	Pdk4	pyruvate dehydrogenase kinase 4	ENSRNOG00000009565	89813
8463	119	66	48	24	1,796	0,845	0,015592307	NM_001014793.1	CUST_7273_PI421866198	NM_001014793	Kpna4	karyopherin subunit alpha 4	ENSRNOG00000010768	361959
2302	237	147	40	75	1,794	0,843	0,026140258	NM_001017464.1	CUST_14582_PI421866198	NM_001017464	Btbd16	BTB domain containing 16	ENSRNOG00000036675	361658
8708	25	16	4	9	1,794	0,844	0,047044256	NM_001001048.1	CUST_14808_PI421866198	NM_001001048	Olr372	olfactory receptor 372	ENSRNOG00000047343	405934
1633	6	3	2	1	1,792	0,841	0,030098251	uc.175+	uc.175+_P1	uc+	NA	NA	NA	NA
7787	8	5	1	3	1,79	0,84	0,02521827	AB115746	AB115746_P1	AB115746	NA	NA	NA	NA
13711	430	256	153	113	1,788	0,838	0,047852023	NM_001108102.1	CUST_7338_PI421866198	NM_001108102	Xpot	exportin for tRNA	ENSRNOG00000007088	314879
42265	720	407	247	152	1,786	0,837	0,027286391	NM_012755.1	CUST_9546_PI421866198	NM_012755	Fyn	FYN proto-oncogene, Src family tyrosine kinase	ENSRNOG00000000596	25150
13465	13	8	2	4	1,784	0,835	0,027778427	AJ286266	AJ286266_P1	AJ286266	NA	lncRNA	NA	NA
39789	278	159	93	68	1,783	0,834	0,031048817	NM_001164297.1	CUST_11727_PI421866198	NM_001164297	Plec	plectin	ENSRNOG00000023781	64204
7319	6	4	1	3	1,777	0,829	0,040804885	NM_001130097.1	CUST_7192_PI421866198	NM_001130097	Atxn2l	ataxin 2-like	ENSRNOG00000018686	361649
4119	36	23	7	12	1,771	0,825	0,022211181	MRAK155528	MRAK155528_P1	MRAK155528	NA	lncRNA	NA	NA
4329	7	4	2	2	1,768	0,822	0,013667144	MRAK053376	MRAK053376_P1	MRAK053376	NA	NA	NA	NA
11761	8	5	3	3	1,768	0,822	0,047024094	MRuc008sxd	MRuc008sxd_P1	MRuc008sxd	NA	NA	NA	NA
4620	14	8	3	4	1,766	0,821	0,016535365	NM_012723.2	CUST_9626_PI421866198	NM_012723	Pigr	polymeric immunoglobulin receptor	ENSRNOG00000004405	NA
201	7	4	2	2	1,765	0,82	0,027658921	NM_001109332.1	CUST_2751_PI421866198	NM_001109332	Dkk4	dickkopf WNT signaling pathway inhibitor 4	ENSRNOG00000019267	502097
184	5	3	2	1	1,764	0,819	0,019077253	MRAK020156	MRAK020156_P1	MRAK020156	NA	NA	NA	NA
8099	33	20	6	8	1,759	0,815	0,012957676	NM_001166578.1	CUST_3855_PI421866198	NM_001166578	Cnih3	cornichon family AMPA receptor auxiliary protein 3	ENSRNOG00000022724	690252
18142	34	20	8	7	1,758	0,814	0,004227518	NM_001008972.1	CUST_1550_PI421866198	NM_001008972	NA	NA	NA	NA
1894	2512	1392	1149	521	1,758	0,814	0,035090874	XR_006671	XR_006671_P1	XR_006671	NA	lncRNA	NA	NA
36	49	31	7	14	1,757	0,813	0,033935168	BC097273	BC097273_P1	BC097273	NA	lncRNA	NA	NA

10231	49	31	8	15	1,756	0,812	0,02355959	NM_001105782.1	CUST_13073_PI421866198	NM_001105782	Mrpl55	mitochondrial ribosomal protein L55	ENSRNOG00000002943	287356
14746	22	14	2	5	1,754	0,81	0,025882756	NM_001166352.1	CUST_5170_PI421866198	NM_001166352	Serpina11	serpin family A member 11	ENSRNOG00000011141	362774
6218	28	18	4	8	1,754	0,811	0,026565147	MRuc007fft	MRuc007fft_P1	MRuc007fft	NA	lncRNA	NA	NA
39235	11	6	6	2	1,754	0,811	0,049922654	AF487544	AF487544_P1	AF487544	NA	lncRNA	NA	NA
466	33	21	5	12	1,753	0,81	0,03650848	U54551	U54551_P1	U54551	NA	lncRNA	NA	NA
9652	392	207	191	50	1,748	0,806	0,032008988	NM_134351.1	CUST_4274_PI421866198	NM_134351	Mat2a	methionine adenosyltransferase 2A	ENSRNOG00000013520	171347
2064	49	31	16	20	1,744	0,802	0,039739127	NM_053429.1	CUST_4009_PI421866198	NM_053429	Fgfr3	fibroblast growth factor receptor 3	ENSRNOG00000016818	84489
12448	29	18	6	7	1,741	0,8	0,020475931	NM_053390.1	CUST_4541_PI421866198	NM_053390	Il12a	interleukin 12A	ENSRNOG00000009468	84405
5041	5	3	2	1	1,741	0,8	0,032462227	AJ278271	AJ278271_P1	AJ278271	NA	NA	NA	NA
6162	23	15	3	6	1,741	0,8	0,042590868	MRAK087269	MRAK087269_P1	MRAK087269	NA	lncRNA	NA	NA
4007	4	2	1	0	1,731	0,792	0,005358776	NM_017128.1	CUST_12786_PI421866198	NM_017128	Inhba	inhibin beta A subunit	ENSRNOG00000014320	29200
3745	23	14	7	4	1,731	0,792	0,011050836	NM_001017484.1	CUST_14562_PI421866198	NM_001017484	Lca5l	LCA5L, lebercilin like	ENSRNOG00000028248	498065
2164	13	8	3	3	1,731	0,791	0,012728132	NM_001025281.1	CUST_13177_PI421866198	NM_001025281	Slc9a8	solute carrier family 9 member A8	ENSRNOG00000008354	311651
14396	5	3	2	1	1,73	0,79	0,01457184	MRAK035730	MRAK035730_P1	MRAK035730	NA	NA	NA	NA
8305	7	4	2	2	1,725	0,787	0,027183071	MRAK148578	MRAK148578_P1	MRAK148578	NA	NA	NA	NA
7774	43	28	6	12	1,722	0,784	0,034952062	NM_001106028.1	CUST_14372_PI421866198	NM_001106028	Cdkn3	cyclin-dependent kinase inhibitor 3	ENSRNOG00000009785	289993
21276	15	9	4	3	1,72	0,782	0,007799164	NM_001044250.1	CUST_14252_PI421866198	NM_001044250	Stat6	signal transducer and activator of transcription 6	ENSRNOG00000025023	362896
10838	101	64	8	30	1,72	0,782	0,016124467	NM_001109537.2	CUST_1352_PI421866198	NM_001109537	Sssca1	Sjogren syndrome/scleroderma autoantigen 1	ENSRNOG00000012736	689397
9360	765	487	198	234	1,715	0,778	0,04065862	NM_031777.2	CUST_10566_PI421866198	NM_031777	Usf1	upstream transcription factor 1	ENSRNOG00000004255	83586
21360	125	81	23	44	1,714	0,777	0,032586692	uc.38-	uc.38-_P1	uc-	NA	lncRNA	NA	NA
7441	111	69	14	28	1,71	0,774	0,009941737	NM_001134971.1	CUST_4007_PI421866198	NM_001134971	Man2b2	mannosidase, alpha, class 2B, member 2	ENSRNOG00000061779	360955
42403	272	164	86	75	1,71	0,774	0,039768803	NM_001164299.1	CUST_11720_PI421866198	NM_001164299	Plec	plectin	ENSRNOG00000023781	64204
14748	4	2	2	0	1,709	0,773	0,042926951	XR_006354	XR_006354_P1	XR_006354	NA	NA	NA	NA
3438	25	15	10	5	1,707	0,772	0,029294342	AY057065	AY057065_P1	AY057065	NA	lncRNA	NA	NA
5474	182	118	35	59	1,705	0,769	0,037887262	NM_001001110.1	CUST_14242_PI421866198	NM_001001110	Olr1694	olfactory receptor 1694	ENSRNOG00000000747	406008
13641	45	29	6	11	1,703	0,768	0,019415545	NM_019270.3	CUST_5917_PI421866198	NM_019270	Kcna3	potassium voltage-gated channel subfamily A member 3	ENSRNOG00000062002	29731
42174	16232	9354	6150	2968	1,7	0,765	0,02974724	NM_017138.1	CUST_12737_PI421866198	NM_017138	Rpsa	ribosomal protein SA	ENSRNOG00000018645	29236
1913	28	18	4	9	1,7	0,766	0,03518445	XR_007846	XR_007846_P1	XR_007846	NA	lncRNA	NA	NA
4623	4	2	1	0	1,695	0,762	0,000135354	AF493158	AF493158_P1	AF493158	NA	NA	NA	NA
4613	34	22	4	9	1,694	0,761	0,020561938	MRuc009rbp	MRuc009rbp_P1	MRuc009rbp	NA	lncRNA	NA	NA
2585	4	2	2	0	1,688	0,755	0,034254038	NM_001025004.1	CUST_14755_PI421866198	NM_001025004	Vsig4	V-set and immunoglobulin domain containing 4	ENSRNOG00000038132	312102
3412	121	73	34	28	1,683	0,751	0,029778272	NM_181089.1	CUST_1594_PI421866198	NM_181089	MAST1	microtubule associated serine/threonine kinase 1	ENSRNOG00000003469	353118
792	4	2	2	0	1,681	0,75	0,014890958	NM_001007093.1	CUST_9788_PI421866198	NM_001007093	Creb3l4	cAMP responsive element binding protein 3-like 4	ENSRNOG00000023493	310616
6150	7	4	1	1	1,678	0,746	0,00065941	NM_172329.1	CUST_5983_PI421866198	NM_172329	Ccr9	C-C motif chemokine receptor 9	ENSRNOG00000006311	282832
3331	27	17	7	7	1,676	0,745	0,030199453	uc.183+	uc.183+_P1	uc+	NA	lncRNA	NA	NA
12028	25	15	8	7	1,675	0,744	0,040579603	NM_001134797.1	CUST_5184_PI421866198	NM_001134797	Dydc2	DPY30 domain containing 2	ENSRNOG00000039591	688422
8430	64	43	9	22	1,674	0,743	0,033696445	NM_022711.4	CUST_1276_PI421866198	NM_022711	Srd5a2	steroid 5 alpha-reductase 2	ENSRNOG00000027042	64677
3565	6	4	2	2	1,672	0,742	0,039648199	MRAK043522	MRAK043522_P1	MRAK043522	NA	NA	NA	NA
6580	16	11	3	5	1,672	0,742	0,048774531	uc.26+	uc.26+_P1	uc+	NA	lncRNA	NA	NA
12785	38	24	8	8	1,667	0,738	0,010189997	MRAK138585	MRAK138585_P1	MRAK138585	NA	lncRNA	NA	NA
16533	3680	2156	1426	657	1,666	0,736	0,034214807	AF531427	AF531427_P1	AF531427	NA	lncRNA	NA	NA
8959	759	491	140	191	1,666	0,737	0,034408074	NM_012768.1	CUST_9515_PI421866198	NM_012768	Drd5	dopamine receptor D5	ENSRNOG00000005338	25195
2001	7	4	1	2	1,664	0,735	0,018831536	MRAK082386	MRAK082386_P1	MRAK082386	NA	NA	NA	NA
16702	15	9	3	2	1,661	0,732	0,003402778	NM_001037441.1	CUST_11747_PI421866198	NM_001037441	Klrc1	killer cell lectin like receptor C1	ENSRNOG00000055196	29683
5896	71	43	21	12	1,659	0,731	0,024258971	NM_001130538.1	CUST_4215_PI421866198	NM_001130538	Slc30a8	solute carrier family 30 member 8	ENSRNOG00000004747	299903
351	17	10	6	4	1,653	0,725	0,043930749	MRAK131730	MRAK131730_P1	MRAK131730	NA	NA	NA	NA
6898	48	32	9	15	1,652	0,724	0,043007007	NM_001101675.1	CUST_11098_PI421866198	NM_001101675	Krt83	keratin 83	ENSRNOG00000032859	681126
6161	10	7	2	3	1,652	0,724	0,049263433	uc.352-	uc.352-_P1	uc-	NA	NA	NA	NA
9640	47	33	5	18	1,651	0,723	0,049638859	J02649	J02649_P1	J02649	NA	lncRNA	NA	NA
3781	5	3	1	1	1,649	0,721	0,017372443	AY383709	AY383709_P1	AY383709	NA	NA	NA	NA
17040	12	7	3	2	1,645	0,718	0,010434449	NM_001014062.1	CUST_10943_PI421866198	NM_001014062	RGD1306195	similar to RAN protein	ENSRNOG00000031013	313163
6427	6	4	1	2	1,642	0,715	0,046328093	uc.225-	uc.225-_P1	uc-	NA	NA	NA	NA
18222	41	26	5	7	1,64	0,714	0,002305187	NM_001107833.1	CUST_6358_PI421866198	NM_001107833	Lrsam1	leucine rich repeat and sterile alpha motif containing 1	ENSRNOG00000022312	311866
18817	14	9	1	4	1,637	0,711	0,016651384	uc.241-	uc.241-_P1	uc-	NA	lncRNA	NA	NA
9812	312	196	31	54	1,634	0,708	0,001816878	NM_001108831.2	CUST_2999_PI421866198	NM_001108831	Kctd11	potassium channel tetramerization domain containing 11	ENSRNOG00000015669	363634
2577	113	76	16	36	1,633	0,707	0,036536328	NM_133521.1	CUST_6463_PI421866198	NM_133521	Fbxo32	F-box protein 32	ENSRNOG00000006738	171043
42268	478	295	159	100	1,627	0,702	0,040112848	NM_012806.1	CUST_9091_PI421866198	NM_012806	Mapk10	mitogen activated protein kinase 10	ENSRNOG00000002079	25272
40959	12551	7282	5244	1450	1,626	0,701	0,031729642	NM_057140.1	CUST_7871_PI421866198	NM_057140	Ppp2r1a	protein phosphatase 2 scaffold subunit A alpha	ENSRNOG00000011282	117281
4187	2489	1494	1044	480	1,624	0,7	0,034275287	XR_005438	XR_005438_P1	XR_005438	NA	lncRNA	NA	NA
378	6	4	1	2	1,622	0,698	0,049753336	MRuc009byg	MRuc009byg_P1	MRuc009byg	NA	lncRNA	NA	NA
5532	13	9	2	3	1,619	0,695	0,036538147	NM_020976.1	CUST_6833_PI421866198	NM_020976	Tmem27	transmembrane protein 27	ENSRNOG00000003960	57395
947	8	5	2	2	1,618	0,695	0,032204527	MRAK135938	MRAK135938_P1	MRAK135938	NA	lncRNA	NA	NA
21258	1507	919	554	296	1,617	0,693	0,043822571	NM_001163477.1	CUST_14286_PI421866198	NM_001163477	Tmem89	transmembrane protein 89	ENSRNOG00000031141	501056
14834	53	34	12	12	1,615	0,692	0,015318889	NM_021680.2	CUST_5225_PI421866198	NM_021680	Nxph4	neurexophilin 4	ENSRNOG00000025059	59316
6973	6	4	2	1	1,615	0,691	0,031752384	NM_013150.1	CUST_10027_PI421866198	NM_013150	Nrcam	neuronal cell adhesion molecule	ENSRNOG00000004067	497815
7347	13	8	2	2	1,614	0,691	0,003552709	MRAK042506	MRAK042506_P1	MRAK042506	NA	lncRNA	NA	NA
2237	16	11	2	4	1,614	0,69	0,010430454	BC169044	BC169044_P1	BC169044	NA	lncRNA	NA	NA
43275	16183	10418	3744	4138	1,613	0,69	0,025666435	NM_001014221.1	CUST_9532_PI421866198	NM_001014221	LOC363337	similar to RIKEN cDNA 1700081O22	ENSRNOG00000042971	363337
9433	2518	1597	590	533	1,61	0,687	0,015259142	BC091320	BC091320_P1	BC091320	NA	lncRNA	NA	NA
1390	7	5	3	1	1,608	0,685	0,023935654	uc.347+	uc.347+_P1	uc+	NA	NA	NA	NA
17723	13	8	2	2	1,601	0,679	0,002040229	NM_001047887.1	CUST_594_PI421866198	NM_001047887	Arsj	NA	NA	NA
1336	8	5	2	2	1,595	0,673	0,046642039	NM_133302.1	CUST_7528_PI421866198	NM_133302	Adarb2	adenosine deaminase, RNA-specific, B2	ENSRNOG00000030775	117088
5023	15	9	4	1	1,59	0,669	0,008242692	NM_153467.1	CUST_4424_PI421866198	NM_153467	Ppp1r17	protein phosphatase 1, regulatory subunit 17	ENSRNOG00000012235	266705
7912	4	2	1	1	1,59	0,669	0,019158899	MRAK142377	MRAK142377_P1	MRAK142377	NA	NA	NA	NA
13722	10	6	1	1	1,587	0,666	0,000408317	MRAK046865	MRAK046865_P1	MRAK046865	NA	lncRNA	NA	NA
3687	4	2	1	0	1,587	0,667	0,01472201	NM_001008803.1	CUST_2426_PI421866198	NM_001008803	Krt82	keratin 82	ENSRNOG00000033403	366991
8639	119	81	8	37	1,586	0,665	0,021258095	NM_017090.2	CUST_13245_PI421866198	NM_017090	Gucy1a3	guanylate cyclase 1 soluble subunit alpha 3	ENSRNOG00000012302	497757
4125	4	2	2	0	1,58	0,66	0,030653713	MRAK050831	MRAK050831_P1	MRAK050831	NA	NA	NA	NA
2931	6	4	1	2	1,58	0,66	0,048634446	MRAK045298	MRAK045298_P1	MRAK045298	NA	NA	NA	NA
1093	65	41	8	4	1,579	0,659	5,54E-05	MRAK138762	MRAK138762_P1	MRAK138762	NA	lncRNA	NA	NA
12704	67	43	7	7	1,578	0,659	0,000221924	NM_001108730.1	CUST_3528_PI421866198	NM_001108730	Mfsd12	major facilitator superfamily domain containing 12	ENSRNOG00000039858	362824
8025	12	8	2	2	1,576	0,656	0,001033678	DQ897665	DQ897665_P1	DQ897665	NA	lncRNA	NA	NA
6395	1590	1094	271	474	1,575	0,655	0,046020613	NM_001109124.1	CUST_3937_PI421866198	NM_001109124	Ccdc112	coiled-coil domain containing 112	ENSRNOG00000003559	498858
11187	7	5	1	2	1,574	0,655	0,029592024	MRuc007xyx	MRuc007xyx_P1	MRuc007xyx	NA	NA	NA	NA
4823	56	36	15	9	1,57	0,651	0,014631122	L16995	L16995_P1	L16995	NA	lncRNA	NA	NA
18647	359	242	59	86	1,57	0,651	0,029480252	NM_001000243.1	CUST_1269_PI421866198	NM_001000243	Olr315	olfactory receptor 315	ENSRNOG00000045841	293609
41332	5925	3608	2143	802	1,569	0,65	0,043171598	NM_017038.1	CUST_13440_PI421866198	NM_017038	Ppm1a	protein phosphatase, Mg2+/Mn2+ dependent, 1A	ENSRNOG00000005916	NA
1472	12	8	2	3	1,566	0,647	0,0102475	NM_001107508.1	CUST_7875_PI421866198	NM_001107508	Klk12	kallikrein related-peptidase 12	ENSRNOG00000034012	308564
4696	3369	2241	462	718	1,566	0,647	0,012787413	NM_001105907.1	CUST_11792_PI421866198	NM_001105907	Trrap	transformation/transcription domain-associated protein	ENSRNOG00000025244	NA
6501	98	67	9	26	1,564	0,645	0,026794316	NM_031736.1	CUST_10637_PI421866198	NM_031736	Slc27a2	solute carrier family 27 member 2	ENSRNOG00000010128	65192
36046	506	317	185	88	1,563	0,644	0,04387336	NM_022684.1	CUST_1647_PI421866198	NM_022684	Bid	BH3 interacting domain death agonist	ENSRNOG00000012439	64625
5536	50	34	4	14	1,561	0,643	0,020411882	NM_001106275.1	CUST_12805_PI421866198	NM_001106275	Abhd2	abhydrolase domain containing 2	ENSRNOG00000017120	293050
42187	999	632	284	123	1,559	0,64	0,007379717	NM_001115046.2	CUST_8242_PI421866198	NM_001115046	Denr	density regulated reinitiation and release factor	ENSRNOG00000001106	687565
1964	8	5	3	1	1,556	0,638	0,046856151	MRAK038464	MRAK038464_P1	MRAK038464	NA	NA	NA	NA
7351	28	19	4	8	1,554	0,636	0,027763483	MRAK085682	MRAK085682_P1	MRAK085682	NA	lncRNA	NA	NA
4878	15	10	4	2	1,553	0,635	0,013242253	AY539901	AY539901_P1	AY539901	NA	lncRNA	NA	NA
2920	24	16	4	4	1,551	0,633	0,002727523	XR_005518	XR_005518_P1	XR_005518	NA	lncRNA	NA	NA
4332	27	17	10	4	1,551	0,634	0,021272159	NM_031034.1	CUST_15238_PI421866198	NM_031034	Gna12	G protein subunit alpha 12	ENSRNOG00000001235	81663
6313	91	61	11	17	1,55	0,632	0,005641339	NM_173127.1	CUST_3740_PI421866198	NM_173127	Prss3	protease, serine 3	ENSRNOG00000031385	362347
398	4	2	2	0	1,548	0,63	0,022191522	uc.103+	uc.103+_P1	uc+	NA	NA	NA	NA
8768	28	18	7	6	1,548	0,63	0,03824449	NM_031132.3	CUST_14804_PI421866198	NM_031132	Tgfbr2	transforming growth factor, beta receptor 2	ENSRNOG00000013265	81810
9937	10482	6533	3668	1502	1,546	0,628	0,038391741	M31322	M31322_P1	M31322	NA	lncRNA	NA	NA
1466	29	19	8	6	1,545	0,627	0,023804995	MRAK165386	MRAK165386_P1	MRAK165386	NA	lncRNA	NA	NA
8208	555	357	164	92	1,544	0,626	0,020164922	NM_031770.3	CUST_10579_PI421866198	NM_031770	Gnb5	G protein subunit beta 5	ENSRNOG00000047799	83579
14137	21	14	4	3	1,543	0,626	0,003013191	NM_032075.3	CUST_11528_PI421866198	NM_032075	Ghsr	growth hormone secretagogue receptor	ENSRNOG00000024119	84022
3083	71	47	8	12	1,54	0,623	0,004138389	MRX95075	MRX95075_P1	MRX95075	NA	lncRNA	NA	NA
2067	4	2	1	0	1,54	0,623	0,006697204	MRAK020557	MRAK020557_P1	MRAK020557	NA	NA	NA	NA
4446	3	2	1	0	1,54	0,622	0,007255695	uc.15-	uc.15-_P1	uc-	NA	NA	NA	NA
42850	1462	936	441	221	1,539	0,622	0,026822478	NM_001107378.1	CUST_8615_PI421866198	NM_001107378	Zfp608	zinc finger protein 608	ENSRNOG00000023197	307296
40236	4004	2474	1502	478	1,539	0,622	0,041290069	NM_001128184.1	CUST_4789_PI421866198	NM_001128184	B3gat3	beta-1,3-glucuronyltransferase 3	ENSRNOG00000019804	293722
8130	96	67	14	26	1,538	0,621	0,041416802	AM040976	AM040976_P1	AM040976	NA	lncRNA	NA	NA
13466	10	6	3	2	1,537	0,62	0,044912722	XR_006866	XR_006866_P1	XR_006866	NA	lncRNA	NA	NA
4719	45	31	8	10	1,533	0,616	0,042043706	NM_001047885.1	CUST_598_PI421866198	NM_001047885	Arse	arylsulfatase E	ENSRNOG00000028350	310326
32921	62	41	17	14	1,531	0,614	0,035126322	MRAK031342	MRAK031342_P1	MRAK031342	NA	lncRNA	NA	NA
2098	4	2	1	0	1,528	0,612	0,007800633	NM_001130542.1	CUST_4191_PI421866198	NM_001130542	Sntb1	syntrophin, beta 1	ENSRNOG00000004821	299940
17467	29	19	5	6	1,527	0,611	0,014151584	uc.152-	uc.152-_P1	uc-	NA	lncRNA	NA	NA
711	24	16	4	6	1,527	0,611	0,025754023	AY169320	AY169320_P1	AY169320	NA	lncRNA	NA	NA
4956	454	310	101	118	1,527	0,611	0,038622625	NM_001037979.1	CUST_8542_PI421866198	NM_001037979	Adipor2	adiponectin receptor 2	ENSRNOG00000007990	312670
3403	146	100	21	35	1,526	0,61	0,020947939	MRAK048034	MRAK048034_P1	MRAK048034	NA	lncRNA	NA	NA
41064	3996	2525	1518	581	1,524	0,608	0,043260474	NM_017327.1	CUST_11371_PI421866198	NM_017327	Gnao1	G protein subunit alpha o1	ENSRNOG00000019482	50664
9981	12	8	3	3	1,523	0,607	0,03206862	XR_006168	XR_006168_P1	XR_006168	NA	lncRNA	NA	NA
40984	11132	7134	3359	1508	1,521	0,605	0,028006869	NM_001014253.2	CUST_9452_PI421866198	NM_001014253	Selenot	selenoprotein T	ENSRNOG00000013507	NA
672	12	8	3	2	1,519	0,603	0,021906862	MRAK137807	MRAK137807_P1	MRAK137807	NA	lncRNA	NA	NA
44495	573	371	168	94	1,518	0,602	0,038346848	NM_053341.1	CUST_4697_PI421866198	NM_053341	Gipc1	GIPC PDZ domain containing family, member 1	ENSRNOG00000003864	83823
17550	22	15	4	4	1,517	0,601	0,006732257	NM_001000260.1	CUST_1194_PI421866198	NM_001000260	Olr376	olfactory receptor 376	ENSRNOG00000013192	293809
17875	5	3	2	1	1,515	0,599	0,043144709	XR_006400	XR_006400_P1	XR_006400	NA	NA	NA	NA
40906	2022	1292	657	295	1,515	0,599	0,049887239	NM_001013069.1	CUST_14672_PI421866198	NM_001013069	Stard10	StAR-related lipid transfer domain containing 10	ENSRNOG00000019491	293150
13633	146	102	23	39	1,514	0,599	0,036690174	uc.238+	uc.238+_P1	uc+	NA	lncRNA	NA	NA
16751	21	14	3	4	1,512	0,596	0,026446292	MRAK052957	MRAK052957_P1	MRAK052957	NA	lncRNA	NA	NA
17197	88	58	26	15	1,512	0,596	0,030900591	MRAK155260	MRAK155260_P1	MRAK155260	NA	lncRNA	NA	NA
4902	113	79	18	28	1,511	0,596	0,035424139	NM_001000571.1	CUST_15000_PI421866198	NM_001000571	Olr710	olfactory receptor 710	ENSRNOG00000059642	366113
44228	399	258	123	68	1,511	0,596	0,041886395	NM_024138.1	CUST_13860_PI421866198	NM_024138	Gng7	G protein subunit gamma 7	ENSRNOG00000019857	58979
20609	44	30	2	9	1,51	0,595	0,016856185	NM_001034081.1	CUST_8401_PI421866198	NM_001034081	Paqr7	progestin and adipoQ receptor family member 7	ENSRNOG00000022054	313615
28342	18	11	8	1	1,51	0,595	0,049695295	NM_053567.1	CUST_3253_PI421866198	NM_053567	Ftcd	formimidoyltransferase cyclodeaminase	ENSRNOG00000001261	89833
2394	26	17	6	6	1,509	0,593	0,029322809	NM_021588.2	CUST_5900_PI421866198	NM_021588	Mb	myoglobin	ENSRNOG00000004583	59108
7237	37	23	18	2	1,508	0,593	0,043150473	MRAK017128	MRAK017128_P1	MRAK017128	NA	lncRNA	NA	NA
1587	7	5	2	2	1,507	0,592	0,041088724	NM_001126303.1	CUST_9351_PI421866198	NM_001126303	Usp43	ubiquitin specific peptidase 43	ENSRNOG00000003785	100147704
16105	18549	12696	4198	4505	1,505	0,59	0,04229922	XR_008916	XR_008916_P1	XR_008916	NA	lncRNA	NA	NA
11318	11	7	4	2	1,505	0,59	0,043716804	K01676	K01676_P1	K01676	NA	NA	NA	NA
4891	4	3	1	1	1,504	0,589	0,026390918	MRAK086912	MRAK086912_P1	MRAK086912	NA	NA	NA	NA
5127	64	42	17	12	1,504	0,589	0,031257272	NM_001130504.1	CUST_4296_PI421866198	NM_001130504	Cenpm	centromere protein M	ENSRNOG00000023262	315164
3799	236	159	66	46	1,504	0,589	0,046897966	MRAK052526	MRAK052526_P1	MRAK052526	NA	lncRNA	NA	NA
18231	63	43	11	12	1,503	0,588	0,027224792	NM_053317.1	CUST_4760_PI421866198	NM_053317	Nr0b1	nuclear receptor subfamily 0, group B, member 1	ENSRNOG00000003765	58850
33962	6729	4273	2457	771	1,503	0,588	0,0472027	NM_017284.1	CUST_11931_PI421866198	NM_017284	Psmb2	proteasome subunit beta 2	ENSRNOG00000011463	29675
2874	12	8	2	1	1,502	0,587	0,003334655	uc.243-	uc.243-_P1	uc-	NA	NA	NA	NA
7853	139	94	16	22	1,501	0,586	0,002699637	NM_172035.1	CUST_7597_PI421866198	NM_172035	Fzd2	frizzled class receptor 2	ENSRNOG00000021962	64512
18224	191	131	24	33	1,501	0,586	0,010615577	NM_001001092.1	CUST_14688_PI421866198	NM_001001092	Olr1395	olfactory receptor 1395	ENSRNOG00000046182	405986
67	59	41	5	15	1,501	0,585	0,018709026	MRAK036098	MRAK036098_P1	MRAK036098	NA	lncRNA	NA	NA
4776	13	9	2	3	1,501	0,586	0,032712399	MRAK032714	MRAK032714_P1	MRAK032714	NA	lncRNA	NA	NA
5099	1418	928	504	228	1,497	0,582	0,032985272	NM_001100658.1	CUST_14933_PI421866198	NM_001100658	Hspa9	heat shock protein family A member 9	ENSRNOG00000019525	291671
29824	102	66	33	14	1,497	0,582	0,039459861	NM_212520.1	CUST_12201_PI421866198	NM_212520	Tomm40	translocase of outer mitochondrial membrane 40	ENSRNOG00000018556	308416
11400	28	20	4	8	1,497	0,582	0,047035753	uc.470-	uc.470-_P1	uc-	NA	lncRNA	NA	NA
43153	20	13	5	3	1,496	0,581	0,02181111	NM_012524.2	CUST_11096_PI421866198	NM_012524	Cebpa	CCAAT/enhancer binding protein alpha	ENSRNOG00000010918	24252
1631	64	43	6	10	1,492	0,577	0,001761089	MRAK015637	MRAK015637_P1	MRAK015637	NA	lncRNA	NA	NA
4368	72	50	8	14	1,491	0,576	0,007539657	NM_001109530.1	CUST_1360_PI421866198	NM_001109530	Spdef	SAM pointed domain containing ets transcription factor	ENSRNOG00000000491	689210
2653	4	3	1	1	1,486	0,571	0,038081383	XR_006843	XR_006843_P1	XR_006843	NA	NA	NA	NA
8950	509	341	104	59	1,485	0,57	0,004967445	MRAK158751	MRAK158751_P1	MRAK158751	NA	lncRNA	NA	NA
35571	7525	5142	1610	1371	1,483	0,569	0,025678228	NM_053849.1	CUST_1433_PI421866198	NM_053849	Pdia4	protein disulfide isomerase family A, member 4	ENSRNOG00000006228	116598
945	37	26	5	9	1,483	0,569	0,02813432	NM_053320.1	CUST_4744_PI421866198	NM_053320	Hgfac	HGF activator	ENSRNOG00000009572	58947
11514	41	28	11	9	1,481	0,566	0,027009012	NM_012975.1	CUST_8491_PI421866198	NM_012975	Lgals4	galectin 4	ENSRNOG00000020338	25474
15929	9	7	2	2	1,48	0,565	0,042384913	XR_005920	XR_005920_P1	XR_005920	NA	lncRNA	NA	NA
2420	4	2	1	0	1,478	0,564	0,041888632	MRAK158207	MRAK158207_P1	MRAK158207	NA	NA	NA	NA
1794	4	2	1	0	1,477	0,562	0,035395596	X91892	X91892_P1	X91892	NA	NA	NA	NA
20606	44	31	9	9	1,476	0,561	0,020017079	NM_001108409.1	CUST_5535_PI421866198	NM_001108409	Fgd3	FYVE, RhoGEF and PH domain containing 3	ENSRNOG00000016225	361223
14240	29	20	4	4	1,473	0,559	0,003614158	NM_133611.1	CUST_5848_PI421866198	NM_133611	Slc2a13	solute carrier family 2 member 13	ENSRNOG00000015741	171147
11741	37	26	6	7	1,473	0,559	0,024177089	NM_001109573.1	CUST_1203_PI421866198	NM_001109573	LOC690276	hypothetical protein LOC690276	ENSRNOG00000024338	690276
494	4	3	1	1	1,472	0,558	0,047874705	MRAK048103	MRAK048103_P1	MRAK048103	NA	NA	NA	NA
38475	86	59	21	19	1,471	0,557	0,033158351	NM_001134845.1	CUST_4803_PI421866198	NM_001134845	LOC688613	hypothetical protein LOC688613	ENSRNOG00000043197	688613
3091	15	11	2	4	1,47	0,556	0,038246051	U76836	U76836_P1	U76836	NA	lncRNA	NA	NA
3787	123	86	33	27	1,47	0,556	0,046462081	MRAK087528	MRAK087528_P1	MRAK087528	NA	lncRNA	NA	NA
13965	126	90	21	29	1,468	0,554	0,043499405	NM_001109171.1	CUST_3800_PI421866198	NM_001109171	Lrrc20	leucine rich repeat containing 20	ENSRNOG00000000560	499430
19480	74	52	17	16	1,466	0,552	0,037448948	NM_001106368.1	CUST_12138_PI421866198	NM_001106368	Pik3ap1	phosphoinositide-3-kinase adaptor protein 1	ENSRNOG00000013309	294048
19746	504	339	164	83	1,464	0,55	0,036467797	NM_001000157.1	CUST_1851_PI421866198	NM_001000157	Olr124	olfactory receptor 124	ENSRNOG00000016018	293260
10263	2278	1509	857	287	1,459	0,545	0,036055519	MRAK155976	MRAK155976_P1	MRAK155976	NA	lncRNA	NA	NA
40821	900	610	184	56	1,456	0,542	0,003205557	NM_033097.2	CUST_8309_PI421866198	NM_033097	Vps52	VPS52 GARP complex subunit	ENSRNOG00000000470	25218
2467	41	30	4	11	1,456	0,542	0,039332209	AF079843	AF079843_P1	AF079843	NA	lncRNA	NA	NA
875	15	11	3	2	1,455	0,541	0,008153788	MRAK029296	MRAK029296_P1	MRAK029296	NA	lncRNA	NA	NA
1144	713	515	74	175	1,454	0,54	0,029611021	NM_019336.1	CUST_5342_PI421866198	NM_019336	Rgs1	regulator of G-protein signaling 1	ENSRNOG00000003895	54289
21361	927	652	175	186	1,453	0,539	0,027655715	NM_001012016.1	CUST_2634_PI421866198	NM_001012016	Tbccd1	TBCC domain containing 1	ENSRNOG00000026458	303830
17036	71	51	8	15	1,448	0,534	0,030913609	NM_001000591.1	CUST_14966_PI421866198	NM_001000591	Olr1070	olfactory receptor 1070	ENSRNOG00000008476	366816
5538	574	382	190	54	1,447	0,533	0,031077275	NM_017340.2	CUST_11329_PI421866198	NM_017340	Acox1	acyl-CoA oxidase 1	ENSRNOG00000008755	50681
1077	52	36	12	9	1,446	0,532	0,024755159	MRAK033142	MRAK033142_P1	MRAK033142	NA	lncRNA	NA	NA
11589	26	18	6	5	1,446	0,532	0,028927316	NM_001002803.2	CUST_6946_PI421866198	NM_001002803	Btnl3	butyrophilin-like 3	ENSRNOG00000039396	407781
37	187	134	20	42	1,445	0,531	0,01657588	NM_001109570.1	CUST_1210_PI421866198	NM_001109570	Prelid3a	PRELI domain containing 3A	ENSRNOG00000017954	690253
14499	39	27	6	8	1,444	0,53	0,012196046	NM_053665.1	CUST_2757_PI421866198	NM_053665	Akap1	A-kinase anchoring protein 1	ENSRNOG00000002373	114124
9559	286	208	40	78	1,444	0,53	0,038831309	NM_001012351.1	CUST_588_PI421866198	NM_001012351	Cpsf4	cleavage and polyadenylation specific factor 4	ENSRNOG00000000985	304277
2468	25	18	6	6	1,441	0,527	0,035840606	XR_009605	XR_009605_P1	XR_009605	NA	lncRNA	NA	NA
22640	1144	807	198	225	1,44	0,527	0,015306523	NM_130416.2	CUST_1088_PI421866198	NM_130416	Anxa7	annexin A7	ENSRNOG00000007136	155423
6247	32	22	8	7	1,439	0,525	0,029148298	NM_001014209.1	CUST_9574_PI421866198	NM_001014209	Plet1	placenta expressed transcript 1	ENSRNOG00000024346	363060
2929	41	29	2	5	1,436	0,522	0,001532774	MRAK047258	MRAK047258_P1	MRAK047258	NA	lncRNA	NA	NA
17041	31	22	4	3	1,436	0,522	0,001985626	NM_001108911.1	CUST_2245_PI421866198	NM_001108911	Upk1a	uroplakin 1A	ENSRNOG00000024331	365227
1502	611	413	201	74	1,434	0,52	0,036444156	MRAK018098	MRAK018098_P1	MRAK018098	NA	lncRNA	NA	NA
18569	128	94	13	28	1,434	0,52	0,038870553	NM_001135607.1	CUST_2629_PI421866198	NM_001135607	Rhox3	reproductive homeobox on X chromosome 3	ENSRNOG00000040010	100190895
4423	29	21	4	5	1,432	0,518	0,011933593	AJ297794	AJ297794_P1	AJ297794	NA	lncRNA	NA	NA
7101	240	175	39	63	1,432	0,518	0,037531429	NM_001108235.1	CUST_6791_PI421866198	NM_001108235	Fbxl17	F-box and leucine-rich repeat protein 17	ENSRNOG00000013875	316663
4608	3	2	1	0	1,431	0,517	0,012404921	NM_001047979.1	CUST_112_PI421866198	NM_001047979	LOC689840	LRRGT00142	ENSRNOG00000031522	689840
7339	41	30	7	9	1,431	0,517	0,040806885	NM_199230.1	CUST_11474_PI421866198	NM_199230	Acvr1b	activin A receptor type 1B	ENSRNOG00000006934	29381
42843	18127	12304	4950	1515	1,43	0,516	0,027065084	NM_001033663.2	CUST_8224_PI421866198	NM_001033663	Araf	A-Raf proto-oncogene, serine/threonine kinase	ENSRNOG00000010838	64363
16749	71	51	16	17	1,43	0,516	0,049713303	M91234	M91234_P1	M91234	NA	lncRNA	NA	NA
19311	12	9	2	3	1,428	0,514	0,048872297	NM_017155.2	CUST_12680_PI421866198	NM_017155	Adora1	adenosine A1 receptor	ENSRNOG00000003442	29290
3594	46	33	6	8	1,424	0,51	0,008818422	NM_022678.1	CUST_1673_PI421866198	NM_022678	Zbtb18	NA	NA	NA
4819	3	2	1	0	1,421	0,507	0,035003464	NM_001106559.2	CUST_10785_PI421866198	NM_001106559	Glt6d1	glycosyltransferase 6 domain containing 1	ENSRNOG00000027900	296577
32219	9735	7074	2030	2411	1,415	0,501	0,041686586	NM_080896.1	CUST_15146_PI421866198	NM_080896	Hnrnph1	heterogeneous nuclear ribonucleoprotein H1	ENSRNOG00000003399	140931
11592	27624	19587	6233	4410	1,414	0,499	0,025041025	NM_022511.2	CUST_2666_PI421866198	NM_022511	Pfn1	profilin 1	ENSRNOG00000003975	64303
19732	700	479	237	75	1,414	0,499	0,04032349	NM_021865.1	CUST_4043_PI421866198	NM_021865	Batf3	basic leucine zipper ATF-like transcription factor 3	ENSRNOG00000003716	60462
14909	31	22	4	5	1,413	0,499	0,011819334	uc.404+	uc.404+_P1	uc+	NA	lncRNA	NA	NA
10935	38	28	7	8	1,406	0,491	0,03689997	NM_022797.1	CUST_1044_PI421866198	NM_022797	Grin2d	glutamate ionotropic receptor NMDA type subunit 2D	ENSRNOG00000021063	24412
6911	11	8	2	1	1,404	0,489	0,004647616	AM293755	AM293755_P1	AM293755	NA	lncRNA	NA	NA
35877	2052	1469	388	300	1,401	0,487	0,019921462	NM_001107323.1	CUST_8719_PI421866198	NM_001107323	Tubgcp3	tubulin, gamma complex associated protein 3	ENSRNOG00000017085	306599
4369	248	182	23	47	1,4	0,486	0,014411648	MRAK087787	MRAK087787_P1	MRAK087787	NA	lncRNA	NA	NA
5519	11	8	2	2	1,399	0,484	0,023255262	MRNR_002862	MRNR_002862_P1	MRNR_002862	NA	lncRNA	NA	NA
8991	61	45	3	16	1,398	0,484	0,025512024	MRAK089474	MRAK089474_P1	MRAK089474	NA	lncRNA	NA	NA
7523	3	2	1	0	1,398	0,483	0,049951251	MRAK133673	MRAK133673_P1	MRAK133673	NA	NA	NA	NA
41160	1112	780	252	49	1,397	0,483	0,008554741	NM_001135260.1	CUST_5069_PI421866198	NM_001135260	Tmem167b	transmembrane protein 167B	ENSRNOG00000000376	108349010
43574	73	53	12	16	1,397	0,482	0,021765446	NM_001080148.1	CUST_7584_PI421866198	NM_001080148	Dhcr24	24-dehydrocholesterol reductase	ENSRNOG00000006787	298298
8777	35	25	8	7	1,397	0,483	0,039142516	MRuc009gpm	MRuc009gpm_P1	MRuc009gpm	NA	lncRNA	NA	NA
20167	20	15	2	3	1,396	0,481	0,010804483	AM293827	AM293827_P1	AM293827	NA	lncRNA	NA	NA
17884	190	139	18	31	1,396	0,482	0,011425259	X94186	X94186_P1	X94186	NA	lncRNA	NA	NA

3249	18	13	4	2	1,396	0,481	0,014925214	AB042271	AB042271_P1	AB042271	NA	lncRNA	NA	NA
43730	74	54	17	13	1,392	0,478	0,034458008	NM_001126272.1	CUST_9901_PI421866198	NM_001126272	Zfp692	zinc finger protein 692	ENSRNOG00000002682	303164
11508	182	131	42	31	1,392	0,478	0,039912687	XR_005502	XR_005502_P1	XR_005502	NA	lncRNA	NA	NA
44805	30838	22075	4654	1901	1,388	0,473	0,001976259	NM_001039378.1	CUST_6308_PI421866198	NM_001039378	Trappc1	trafficking protein particle complex 1	ENSRNOG00000037627	287427
12703	12	9	1	3	1,388	0,473	0,034081307	MRAK011816	MRAK011816_P1	MRAK011816	NA	lncRNA	NA	NA
7277	224	160	57	34	1,388	0,473	0,036312908	BC099096	BC099096_P1	BC099096	NA	lncRNA	NA	NA
41840	10909	7833	2114	1235	1,385	0,47	0,013216964	NM_174864.3	CUST_11168_PI421866198	NM_174864	Ncstn	nicastrin	ENSRNOG00000005355	289231
8271	6877	5079	941	1329	1,385	0,47	0,024569118	NM_001105828.2	CUST_12544_PI421866198	NM_001105828	Supt4h1	SPT4 homolog, DSIF elongation factor subunit	ENSRNOG00000007845	287608
17033	78	58	11	15	1,385	0,47	0,032656322	uc.286-	uc.286-_P1	uc-	NA	lncRNA	NA	NA
14833	37	28	5	8	1,382	0,467	0,026085993	AF145795	AF145795_P1	AF145795	NA	lncRNA	NA	NA
4307	16	11	2	3	1,381	0,465	0,010370914	MRuc009sno	MRuc009sno_P1	MRuc009sno	NA	lncRNA	NA	NA
44064	18282	13080	3308	890	1,38	0,464	0,004794571	NM_017088.2	CUST_13259_PI421866198	NM_017088	Gdi1	GDP dissociation inhibitor 1	ENSRNOG00000056870	25183
6614	3248	2350	708	478	1,38	0,465	0,032118071	NM_017249.2	CUST_12020_PI421866198	NM_017249	Esyt1	extended synaptotagmin 1	ENSRNOG00000060753	29579
985	242	175	51	32	1,379	0,464	0,024270994	MRAK049388	MRAK049388_P1	MRAK049388	NA	lncRNA	NA	NA
38264	4649	3357	993	642	1,378	0,463	0,024205489	NM_030987.2	CUST_12915_PI421866198	NM_030987	Gnb1	G protein subunit beta 1	ENSRNOG00000016638	24400
6727	13	10	1	1	1,376	0,461	0,001430569	MRAK036896	MRAK036896_P1	MRAK036896	NA	lncRNA	NA	NA
2828	44	33	4	7	1,376	0,461	0,00501313	BC091694	BC091694_P1	BC091694	NA	lncRNA	NA	NA
38768	2832	2059	380	258	1,374	0,458	0,002920468	NM_001014785.1	CUST_7288_PI421866198	NM_001014785	Ncbp1	nuclear cap binding protein subunit 1	ENSRNOG00000023605	298075
19081	15	11	2	3	1,374	0,458	0,041828561	NM_001000979.1	CUST_12322_PI421866198	NM_001000979	Olr1370	olfactory receptor 1370	ENSRNOG00000046347	405334
4239	6946	5081	1419	1135	1,374	0,458	0,042717557	NM_001012195.1	CUST_1596_PI421866198	NM_001012195	Rnf41	ring finger protein 41	ENSRNOG00000023456	362814
23070	70	51	13	12	1,373	0,457	0,02901707	NM_001100497.1	CUST_488_PI421866198	NM_001100497	Hoxb9	homeo box B9	ENSRNOG00000007573	287647
4699	1306	941	363	193	1,373	0,457	0,049196475	NM_001106259.2	CUST_12857_PI421866198	NM_001106259	Akt1s1	AKT1 substrate 1	ENSRNOG00000020289	292887
22644	81	59	13	12	1,371	0,455	0,014319632	NM_024383.1	CUST_12347_PI421866198	NM_024383	Hes5	hes family bHLH transcription factor 5	ENSRNOG00000013850	79225
16094	2815	2015	726	288	1,367	0,451	0,037338948	NM_001107889.1	CUST_6252_PI421866198	NM_001107889	Mlf2	myeloid leukemia factor 2	ENSRNOG00000016589	312709
292	22	16	4	3	1,365	0,449	0,019106545	XR_008794	XR_008794_P1	XR_008794	NA	lncRNA	NA	NA
28310	284	206	62	32	1,365	0,449	0,028311665	BC089925	BC089925_P1	BC089925	NA	lncRNA	NA	NA
20253	53	40	9	11	1,364	0,448	0,046431537	NM_001099506.1	CUST_12970_PI421866198	NM_001099506	Vom2r47	vomeronasal 2 receptor, 47	ENSRNOG00000028345	365807
7187	124	92	18	20	1,358	0,442	0,017838235	NM_001108252.1	CUST_6772_PI421866198	NM_001108252	Usp51	ubiquitin specific peptidase 51	ENSRNOG00000021434	317398
30908	35	25	9	2	1,355	0,438	0,039722585	NM_001110319.1	CUST_7708_PI421866198	NM_001110319	Maz	MYC associated zinc finger protein	ENSRNOG00000055082	NA
2493	3	2	1	0	1,354	0,437	0,013976084	MRAK048196	MRAK048196_P1	MRAK048196	NA	NA	NA	NA
2065	60	46	7	15	1,354	0,438	0,048514802	MRAK160296	MRAK160296_P1	MRAK160296	NA	lncRNA	NA	NA
25763	629	449	177	37	1,354	0,437	0,049173455	NM_001012470.1	CUST_48_PI421866198	NM_001012470	Irf2bpl	interferon regulatory factor 2 binding protein-like	ENSRNOG00000011026	314329
4126	75	57	10	15	1,353	0,436	0,032679449	MRAK031176	MRAK031176_P1	MRAK031176	NA	lncRNA	NA	NA
16266	39	29	6	5	1,352	0,435	0,010355924	NM_001167664.1	CUST_15259_PI421866198	NM_001167664	Cd300lb	CD300 molecule-like family member b	ENSRNOG00000036728	501738
43303	19787	14489	4039	1962	1,352	0,435	0,015180288	NM_001107698.1	CUST_7353_PI421866198	NM_001107698	Hist2h3c2	histone cluster 2, H3c2	ENSRNOG00000053081	310678
2982	85	63	7	11	1,351	0,434	0,0048719	AB087406	AB087406_P1	AB087406	NA	lncRNA	NA	NA
831	354	265	60	69	1,351	0,434	0,025352564	NM_134376.1	CUST_4223_PI421866198	NM_134376	Clstn3	calsyntenin 3	ENSRNOG00000011156	171393
3749	7865	5673	2382	697	1,351	0,434	0,037727714	MRAK003448	MRAK003448_P1	MRAK003448	NA	lncRNA	NA	NA
40322	12782	9438	2039	1272	1,348	0,431	0,00969095	NM_001082540.1	CUST_14031_PI421866198	NM_001082540	Hnrnpd	heterogeneous nuclear ribonucleoprotein D	ENSRNOG00000002292	79256
40212	21662	16196	2635	3054	1,348	0,431	0,009974032	NM_001037287.1	CUST_13108_PI421866198	NM_001037287	Hnrnpf	heterogeneous nuclear ribonucleoprotein F	ENSRNOG00000014562	64200
13018	16	12	2	3	1,347	0,43	0,032197454	NM_170787.1	CUST_9185_PI421866198	NM_170787	Erc2	ELKS/RAB6-interacting/CAST family member 2	ENSRNOG00000015148	259269
20596	103	76	21	15	1,347	0,43	0,038667028	NM_001135084.1	CUST_6242_PI421866198	NM_001135084	Zfp322a	zinc finger protein 322a	ENSRNOG00000017318	680201
3630	439	326	100	73	1,346	0,428	0,045692509	AY310158	AY310158_P1	AY310158	NA	lncRNA	NA	NA
361	62	47	5	9	1,345	0,428	0,009971996	XR_006574	XR_006574_P1	XR_006574	NA	lncRNA	NA	NA
4312	3	2	1	0	1,343	0,425	0,020656381	MRAK035699	MRAK035699_P1	MRAK035699	NA	NA	NA	NA
3767	119	89	23	17	1,341	0,423	0,0229062	NM_001005762.1	CUST_12584_PI421866198	NM_001005762	Sorbs3	sorbin and SH3 domain containing 3	ENSRNOG00000008829	282843
20062	22	17	4	4	1,341	0,423	0,048049033	NM_001105792.1	CUST_13052_PI421866198	NM_001105792	Hes7	hes family bHLH transcription factor 7	ENSRNOG00000007391	287423
10727	63	48	7	14	1,34	0,423	0,035625821	NM_001129775.1	CUST_12985_PI421866198	NM_001129775	NA	NA	NA	NA
2286	3891	2842	1128	415	1,339	0,421	0,040426445	NM_001044275.1	CUST_14183_PI421866198	NM_001044275	Elovl1	ELOVL fatty acid elongase 1	ENSRNOG00000028448	679532
39366	842	619	194	88	1,337	0,419	0,043859649	NM_134468.1	CUST_3505_PI421866198	NM_134468	Camk1	calcium/calmodulin-dependent protein kinase I	ENSRNOG00000021781	171503
8787	441	335	46	72	1,336	0,418	0,012451209	NM_001109215.1	CUST_3290_PI421866198	NM_001109215	Xrcc2	X-ray repair cross complementing 2	ENSRNOG00000007493	499966
41570	15270	11408	2896	2134	1,336	0,417	0,028239007	NM_138877.1	CUST_3396_PI421866198	NM_138877	Cyb5r3	cytochrome b5 reductase 3	ENSRNOG00000009592	25035
1138	29	22	4	6	1,336	0,418	0,038955928	NM_031799.1	CUST_10503_PI421866198	NM_031799	Pax4	paired box 4	ENSRNOG00000008020	83630
42936	12662	9440	2008	1233	1,335	0,417	0,012514554	NM_198771.1	CUST_11640_PI421866198	NM_198771	Fam3c	family with sequence similarity 3, member C	ENSRNOG00000060349	312159
5365	13	10	3	1	1,335	0,417	0,025457648	XR_009202	XR_009202_P1	XR_009202	NA	lncRNA	NA	NA
9570	16	12	2	3	1,335	0,417	0,047271599	XR_006049	XR_006049_P1	XR_006049	NA	lncRNA	NA	NA
6692	6154	4642	699	827	1,334	0,416	0,0072304	MRAK049761	MRAK049761_P1	MRAK049761	NA	lncRNA	NA	NA
4684	64	48	5	9	1,334	0,416	0,008284192	NM_001134504.1	CUST_6759_PI421866198	NM_001134504	Klhl23	kelch-like family member 23	ENSRNOG00000007981	311114
8905	48	36	10	8	1,333	0,415	0,037123129	NM_001108598.1	CUST_4819_PI421866198	NM_001108598	Dusp15	dual specificity phosphatase 15	ENSRNOG00000008534	362238
3352	20	15	4	3	1,332	0,414	0,036098307	NM_001013930.1	CUST_8785_PI421866198	NM_001013930	LOC298139	similar to RIKEN cDNA 2310003M01	ENSRNOG00000045733	298139
43447	1239	928	128	55	1,331	0,413	0,000663133	NM_198734.2	CUST_11726_PI421866198	NM_198734	Yif1b	Yip1 interacting factor homolog B, membrane trafficking protein	ENSRNOG00000055286	NA
16609	14	11	3	2	1,331	0,412	0,016691213	NM_001109154.1	CUST_3863_PI421866198	NM_001109154	Apobr	apolipoprotein B receptor	ENSRNOG00000017403	499264
3019	169	128	34	31	1,329	0,411	0,042874742	MRAK089363	MRAK089363_P1	MRAK089363	NA	lncRNA	NA	NA
42171	1479	1109	188	100	1,328	0,41	0,00266464	NM_001134519.1	CUST_6728_PI421866198	NM_001134519	Ttc39a	tetratricopeptide repeat domain 39A	ENSRNOG00000047573	298366
8756	62	46	9	2	1,327	0,408	0,003051214	AY310152	AY310152_P1	AY310152	NA	lncRNA	NA	NA
42168	19068	14317	2650	1530	1,327	0,408	0,00491518	NM_020099.2	CUST_11560_PI421866198	NM_020099	Leprot	leptin receptor overlapping transcript	ENSRNOG00000050849	56766
4932	13	10	2	2	1,327	0,408	0,019922241	MRAK079949	MRAK079949_P1	MRAK079949	NA	lncRNA	NA	NA
12269	16	12	4	3	1,327	0,408	0,047784966	XR_009317	XR_009317_P1	XR_009317	NA	lncRNA	NA	NA
41746	4945	3738	568	445	1,324	0,404	0,003709601	NM_012517.2	CUST_11111_PI421866198	NM_012517	Cacna1c	calcium voltage-gated channel subunit alpha1 C	ENSRNOG00000007090	24239
5775	276	211	38	47	1,324	0,405	0,025327372	MRAK033428	MRAK033428_P1	MRAK033428	NA	lncRNA	NA	NA
15427	9	7	2	1	1,322	0,403	0,027268429	MRAK135112	MRAK135112_P1	MRAK135112	NA	lncRNA	NA	NA
12908	243	182	59	29	1,321	0,402	0,048434378	NM_031005.3	CUST_15271_PI421866198	NM_031005	Actn1	actinin, alpha 1	ENSRNOG00000056756	81634
1119	3	2	1	0	1,32	0,401	0,01708879	uc.143+	uc.143+_P1	uc+	NA	NA	NA	NA
7656	162	123	29	24	1,32	0,4	0,026590697	NM_001109540.1	CUST_1334_PI421866198	NM_001109540	Nkain3	Sodium/potassium transporting ATPase interacting 3	ENSRNOG00000013085	689576
14712	71	54	15	9	1,319	0,399	0,045146288	D26498	D26498_P1	D26498	NA	lncRNA	NA	NA
8489	155	116	29	13	1,317	0,398	0,015129739	MRuc008avr	MRuc008avr_P1	MRuc008avr	NA	lncRNA	NA	NA
12296	265	205	35	50	1,317	0,398	0,039926206	NM_001134833.2	CUST_4832_PI421866198	NM_001134833	Ccdc89	coiled-coil domain containing 89	ENSRNOG00000022766	293107
38013	7530	5729	1520	1232	1,316	0,396	0,048369316	NM_001109291.1	CUST_3117_PI421866198	NM_001109291	H2afx	H2A histone family, member X	ENSRNOG00000010386	500987
7296	13	10	4	1	1,315	0,395	0,045232844	NM_001099495.1	CUST_13378_PI421866198	NM_001099495	Vom2r55	NA	NA	NA
36563	63341	48362	3621	4540	1,313	0,392	0,000570175	NM_022669.1	CUST_1710_PI421866198	NM_022669	Scg2	secretogranin II	ENSRNOG00000015055	24765
6693	39	29	8	6	1,312	0,392	0,048462675	NM_139327.1	CUST_3227_PI421866198	NM_139327	Hmga1	high mobility group AT-hook 1	ENSRNOG00000000488	117062
8528	21	16	3	3	1,31	0,39	0,01918362	MRAK047503	MRAK047503_P1	MRAK047503	NA	lncRNA	NA	NA
751	47	35	10	5	1,31	0,389	0,03205536	BC088332	BC088332_P1	BC088332	NA	lncRNA	NA	NA
43032	28444	21789	3438	3136	1,309	0,388	0,008337598	NM_053973.2	CUST_786_PI421866198	NM_053973	Rraga	Ras-related GTP binding A	ENSRNOG00000007051	117044
43784	5187	3926	968	361	1,306	0,385	0,016878203	NM_012796.2	CUST_9439_PI421866198	NM_012796	Gstt2	glutathione S-transferase, theta 2	ENSRNOG00000052415	29487
12283	111	87	13	20	1,306	0,385	0,025815185	NM_001106531.1	CUST_10833_PI421866198	NM_001106531	Bpifb2	BPI fold containing family B, member 2	ENSRNOG00000012197	296290
1177	69	52	14	9	1,306	0,385	0,043507007	XR_009252	XR_009252_P1	XR_009252	NA	lncRNA	NA	NA
41858	619	473	126	84	1,306	0,386	0,044873152	NM_017220.1	CUST_12117_PI421866198	NM_017220	Pts	6-pyruvoyl-tetrahydropterin synthase	ENSRNOG00000009250	29498
5698	490	374	95	59	1,305	0,384	0,036793999	NM_017063.1	CUST_13337_PI421866198	NM_017063	Kpnb1	karyopherin subunit beta 1	ENSRNOG00000009275	24917
37847	507	385	105	55	1,305	0,384	0,042475743	NM_139260.1	CUST_3782_PI421866198	NM_139260	Il3ra	interleukin 3 receptor subunit alpha	ENSRNOG00000001325	246144
12678	25	20	3	4	1,304	0,383	0,02822615	MRAK132815	MRAK132815_P1	MRAK132815	NA	lncRNA	NA	NA
40725	9834	7461	1819	721	1,303	0,382	0,019416274	NM_053490.1	CUST_3853_PI421866198	NM_053490	Xpo1	exportin 1	ENSRNOG00000009935	85252
43762	96	74	13	15	1,301	0,379	0,020766672	NM_001002829.1	CUST_6892_PI421866198	NM_001002829	Rasl11a	RAS-like family 11 member A	ENSRNOG00000000956	304268
42438	22212	17233	1622	2831	1,3	0,379	0,006424426	NM_001008289.1	CUST_6176_PI421866198	NM_001008289	Sbds	SBDS ribosome assembly guanine nucleotide exchange factor	ENSRNOG00000000888	288615
39872	145893	111173	23915	8044	1,3	0,379	0,008802497	NM_001008558.1	CUST_4348_PI421866198	NM_001008558	Ndn	necdin, MAGE family member	ENSRNOG00000010146	308690
15473	3	2	1	0	1,3	0,378	0,030118451	XR_005704	XR_005704_P1	XR_005704	NA	NA	NA	NA
43950	46415	35722	7257	5638	1,298	0,376	0,022402384	NM_013156.1	CUST_10018_PI421866198	NM_013156	Ctsl	cathepsin L	ENSRNOG00000018566	25697
634	2586	1966	595	284	1,298	0,377	0,036763232	MRAK140938	MRAK140938_P1	MRAK140938	NA	lncRNA	NA	NA
44463	1804	1367	386	146	1,298	0,377	0,038904404	NM_001107921.1	CUST_5813_PI421866198	NM_001107921	Casp8ap2	caspase 8 associated protein 2	ENSRNOG00000006487	313128
11956	31	24	3	2	1,293	0,37	0,001259671	MRAK018620	MRAK018620_P1	MRAK018620	NA	lncRNA	NA	NA
6147	492	382	68	67	1,293	0,371	0,016277221	NM_001106058.1	CUST_14291_PI421866198	NM_001106058	Cldn10	claudin 10	ENSRNOG00000010085	290485
10147	731	573	91	123	1,292	0,369	0,029367669	NM_001025028.2	CUST_14692_PI421866198	NM_001025028	Fam35a	family with sequence similarity 35, member A	ENSRNOG00000059057	364514
41916	262	200	60	24	1,292	0,37	0,041054469	NM_001107511.1	CUST_7861_PI421866198	NM_001107511	Ap2a1	adaptor-related protein complex 2, alpha 1 subunit	ENSRNOG00000026243	308578
9917	185	147	13	40	1,292	0,369	0,045568369	NM_022191.1	CUST_5015_PI421866198	NM_022191	Syt6	synaptotagmin 6	ENSRNOG00000019163	60565
38514	2883	2243	203	282	1,291	0,368	0,002786193	NM_001107286.1	CUST_9040_PI421866198	NM_001107286	Tgds	TDP-glucose 4,6-dehydratase	ENSRNOG00000009661	306164
42766	57340	43942	9872	2822	1,29	0,367	0,014787714	NM_001109205.1	CUST_3311_PI421866198	NM_001109205	Eid1	EP300 interacting inhibitor of differentiation 1	ENSRNOG00000008452	499882
2389	71	56	11	9	1,288	0,365	0,02390408	uc.282+	uc.282+_P1	uc+	NA	lncRNA	NA	NA
43954	37521	28835	6830	3479	1,288	0,365	0,030858553	NM_001106924.1	CUST_8393_PI421866198	NM_001106924	Cab39	calcium binding protein 39	ENSRNOG00000017297	301574
9421	37	29	5	6	1,285	0,362	0,043107785	MRAK137921	MRAK137921_P1	MRAK137921	NA	lncRNA	NA	NA
7668	31	24	5	5	1,285	0,362	0,048244912	NM_001108956.1	CUST_2183_PI421866198	NM_001108956	Elf5	E74-like factor 5	ENSRNOG00000008282	366142
14670	85	67	12	14	1,284	0,361	0,033363822	NM_001001093.1	CUST_14687_PI421866198	NM_001001093	Olr1396	olfactory receptor 1396	ENSRNOG00000048331	405987
43440	20140	15623	2429	983	1,283	0,36	0,003248758	NM_172321.1	CUST_5998_PI421866198	NM_172321	Slc6a15	solute carrier family 6 member 15	ENSRNOG00000027468	282712
44208	35615	27639	4673	2112	1,282	0,359	0,005470676	NM_001106157.2	CUST_13580_PI421866198	NM_001106157	Srp19	signal recognition particle 19	ENSRNOG00000020204	291685
44128	2269	1771	181	137	1,281	0,358	0,001343923	NM_001033702.1	CUST_7928_PI421866198	NM_001033702	Rnf126	ring finger protein 126	ENSRNOG00000009028	314613
3511	17	14	1	2	1,281	0,358	0,010358782	MRAK018440	MRAK018440_P1	MRAK018440	NA	lncRNA	NA	NA
42082	21353	16654	3183	2424	1,281	0,357	0,019362352	NM_001163164.1	CUST_513_PI421866198	NM_001163164	Mettl9	methyltransferase like 9	ENSRNOG00000025940	100302453
44808	7960	6176	1169	518	1,28	0,356	0,01056834	NM_001106268.1	CUST_12833_PI421866198	NM_001106268	Chsy1	chondroitin sulfate synthase 1	ENSRNOG00000012698	292999
12372	145	116	15	26	1,279	0,355	0,045735472	NM_001012228.1	CUST_1204_PI421866198	NM_001012228	Xkr5	XK related 5	ENSRNOG00000028636	497083
11857	98	78	9	14	1,277	0,352	0,020351704	NM_001108606.1	CUST_4327_PI421866198	NM_001108606	Tp53rk	TP53 regulating kinase	ENSRNOG00000007285	362272
3035	12	9	3	1	1,277	0,352	0,034581236	NM_024486.1	CUST_11509_PI421866198	NM_024486	Acvr1	activin A receptor type 1	ENSRNOG00000005033	79558
44897	6453	5016	920	198	1,276	0,352	0,005791136	NM_001007756.1	CUST_6479_PI421866198	NM_001007756	Gemin8	gem (nuclear organelle) associated protein 8	ENSRNOG00000029038	363462
5649	76	61	8	13	1,276	0,352	0,033918114	MRAK049379	MRAK049379_P1	MRAK049379	NA	lncRNA	NA	NA
6030	33	26	2	2	1,275	0,351	0,001189186	NM_001108482.1	CUST_5358_PI421866198	NM_001108482	Prrg2	proline rich and Gla domain 2	ENSRNOG00000020517	361570
43276	2754	2145	442	206	1,275	0,351	0,016294516	NM_001109529.1	CUST_1377_PI421866198	NM_001109529	Kin	Kin17 DNA and RNA binding protein	ENSRNOG00000026690	689197
30831	3	2	1	0	1,275	0,35	0,049542127	MRAK052171	MRAK052171_P1	MRAK052171	NA	NA	NA	NA
41148	772	605	91	63	1,274	0,35	0,007115425	NM_001108339.1	CUST_6120_PI421866198	NM_001108339	Gatc	glutamyl-tRNA amidotransferase subunit C	ENSRNOG00000001161	360821
42427	22091	17356	2664	1837	1,271	0,346	0,008301064	NM_001126089.2	CUST_11275_PI421866198	NM_001126089	Cdc42se2	CDC42 small effector 2	ENSRNOG00000042449	691031
43288	6564	5109	1214	456	1,27	0,345	0,024511284	NM_001047883.1	CUST_601_PI421866198	NM_001047883	Mrpl49	mitochondrial ribosomal protein L49	ENSRNOG00000020975	309176
39871	72428	57164	6825	6449	1,269	0,343	0,005224099	NM_001113751.1	CUST_10343_PI421866198	NM_001113751	Cdk2ap1	cyclin-dependent kinase 2 associated protein 1	ENSRNOG00000001070	360804
41994	140646	109861	24378	5066	1,266	0,34	0,015496919	NM_013177.1	CUST_9958_PI421866198	NM_013177	Got2	glutamic-oxaloacetic transaminase 2	ENSRNOG00000011782	25721
43711	1013	797	181	123	1,265	0,339	0,040970261	NM_001079893.1	CUST_13483_PI421866198	NM_001079893	Dusp14	dual specificity phosphatase 14	ENSRNOG00000030091	360580
3588	1196	946	189	159	1,265	0,339	0,041002054	AF247812	AF247812_P1	AF247812	NA	lncRNA	NA	NA
42765	17112	13753	383	2689	1,263	0,337	0,016552379	NM_183402.1	CUST_8126_PI421866198	NM_183402	Polr2m	NA	NA	NA
42506	13377	10503	2322	1137	1,263	0,337	0,026493502	NM_001025027.1	CUST_14693_PI421866198	NM_001025027	Fundc1	FUN14 domain containing 1	ENSRNOG00000003470	363442
4343	22	18	1	4	1,263	0,337	0,045100193	AF196254	AF196254_P1	AF196254	NA	lncRNA	NA	NA
39896	4977	3929	723	475	1,262	0,336	0,019938351	NM_145678.1	CUST_5647_PI421866198	NM_145678	Vps4a	vacuolar protein sorting 4 homolog A	ENSRNOG00000020351	NA
6985	27	22	3	5	1,262	0,335	0,042059026	MRAK132853	MRAK132853_P1	MRAK132853	NA	lncRNA	NA	NA
15736	195	155	22	22	1,261	0,335	0,014653139	NM_001037368.1	CUST_12503_PI421866198	NM_001037368	Defb29	defensin beta 29	ENSRNOG00000023195	641519
1802	134	108	9	18	1,259	0,332	0,011064814	NM_001025060.1	CUST_14603_PI421866198	NM_001025060	LOC502684	hypothetical protein LOC502684	ENSRNOG00000023721	502684
42472	2937	2297	617	221	1,259	0,333	0,046024798	NM_019133.1	CUST_6690_PI421866198	NM_019133	Syn1	synapsin I	ENSRNOG00000010365	24949
41066	196	158	24	34	1,257	0,33	0,049049022	NM_001044241.1	CUST_14284_PI421866198	NM_001044241	Atl3	atlastin GTPase 3	ENSRNOG00000021203	309187
38854	5138	4096	364	298	1,254	0,327	0,001681631	NM_198774.2	CUST_11632_PI421866198	NM_198774	Tmem110	transmembrane protein 110	ENSRNOG00000017051	361110
1960	3	2	1	0	1,254	0,327	0,028383737	MRAK088552	MRAK088552_P1	MRAK088552	NA	NA	NA	NA
5479	138	111	19	19	1,254	0,326	0,042966471	NM_001106301.1	CUST_12312_PI421866198	NM_001106301	Nsmce4a	NSE4 homolog A, SMC5-SMC6 complex component	ENSRNOG00000020452	NA
16021	71	57	5	7	1,253	0,325	0,007912803	NM_001107000.1	CUST_10613_PI421866198	NM_001107000	Fstl4	follistatin-like 4	ENSRNOG00000006565	303130
39710	12512	10017	1345	1427	1,253	0,325	0,015770998	NM_057131.1	CUST_7898_PI421866198	NM_057131	Prpsap2	phosphoribosyl pyrophosphate synthetase-associated protein 2	ENSRNOG00000002724	117272
40243	7367	5862	1080	701	1,253	0,326	0,018912523	NM_001107954.1	CUST_5764_PI421866198	NM_001107954	Reck	reversion-inducing-cysteine-rich protein with kazal motifs	ENSRNOG00000014863	313488
8778	25	20	3	2	1,251	0,323	0,015396727	AF481945	AF481945_P1	AF481945	NA	lncRNA	NA	NA
6107	616	491	99	69	1,251	0,323	0,03829817	MRuc008xkb	MRuc008xkb_P1	MRuc008xkb	NA	lncRNA	NA	NA
14772	51167	40902	7402	6258	1,251	0,323	0,040321452	XR_006831	XR_006831_P1	XR_006831	NA	lncRNA	NA	NA
42436	3011	2409	118	82	1,25	0,322	0,000303349	NM_001037356.1	CUST_12526_PI421866198	NM_001037356	Pak1ip1	PAK1 interacting protein 1	ENSRNOG00000023799	361232
41153	30858	24689	1091	1150	1,25	0,322	0,000352294	NM_031785.1	CUST_10536_PI421866198	NM_031785	Atp6ap1	ATPase H+ transporting accessory protein 1	ENSRNOG00000054352	83615
10185	82396	65582	10607	4503	1,25	0,322	0,008760837	MRAK156057	MRAK156057_P1	MRAK156057	NA	lncRNA	NA	NA
44126	90592	72900	6951	10212	1,25	0,322	0,010350225	NM_031102.2	CUST_14874_PI421866198	NM_031102	Rpl18	ribosomal protein L18	ENSRNOG00000021035	81766
4326	190	152	25	9	1,246	0,317	0,010522451	NM_153731.1	CUST_2560_PI421866198	NM_153731	Zfp709	zinc finger protein 709	ENSRNOG00000016186	266773
35465	4115	3300	717	533	1,246	0,317	0,045314801	NM_001012120.1	CUST_1849_PI421866198	NM_001012120	Naga	N-acetyl galactosaminidase, alpha	ENSRNOG00000008064	315165
442	55	44	11	4	1,246	0,317	0,047530326	MRAK011652	MRAK011652_P1	MRAK011652	NA	lncRNA	NA	NA
15451	3	2	1	0	1,244	0,315	0,035854504	MRAK082311	MRAK082311_P1	MRAK082311	NA	NA	NA	NA
2374	6476	5130	1346	278	1,243	0,314	0,035637391	MRNR_027323	MRNR_027323_P1	MRNR_027323	NA	lncRNA	NA	NA
9022	6184	4919	1204	510	1,243	0,314	0,046656927	NM_022394.1	CUST_3648_PI421866198	NM_022394	Safb	scaffold attachment factor B	ENSRNOG00000050543	64196
44296	2385	1915	274	154	1,242	0,313	0,008124451	NM_001107524.1	CUST_7836_PI421866198	NM_001107524	Rgma	repulsive guidance molecule family member A	ENSRNOG00000012874	308739
4949	2185	1737	404	152	1,242	0,313	0,045768229	XR_007919	XR_007919_P1	XR_007919	NA	lncRNA	NA	NA
5380	15	12	2	1	1,241	0,312	0,022326335	AF217571	AF217571_P1	AF217571	NA	lncRNA	NA	NA
16957	38	31	3	5	1,241	0,311	0,023789101	NM_147137.2	CUST_1966_PI421866198	NM_147137	LOC257643	cystatin SC	ENSRNOG00000004858	257643
2682	73698	59281	11295	7481	1,24	0,31	0,035194977	NM_031114.1	CUST_14847_PI421866198	NM_031114	S100a10	S100 calcium binding protein A10	ENSRNOG00000023226	81778
33327	7692	6239	583	771	1,238	0,308	0,009907761	NM_001004415.1	CUST_2584_PI421866198	NM_001004415	Tacc2	transforming, acidic coiled-coil containing protein 2	ENSRNOG00000020457	309025
6575	649	531	54	105	1,238	0,308	0,038486438	BC159426	BC159426_P1	BC159426	NA	lncRNA	NA	NA
37542	12632	10135	2108	1115	1,237	0,306	0,046112619	NM_001082539.1	CUST_14065_PI421866198	NM_001082539	Hnrnpd	heterogeneous nuclear ribonucleoprotein D	ENSRNOG00000002292	79256
38510	75774	61757	4665	9217	1,236	0,306	0,012815822	NM_001110345.2	CUST_7673_PI421866198	NM_001110345	Ube2v1	NA	NA	NA
40461	12523	10235	859	1827	1,236	0,306	0,022292417	NM_153303.2	CUST_5163_PI421866198	NM_153303	Tor1a	torsin family 1, member A	ENSRNOG00000006894	266606
40895	8478	6892	283	720	1,235	0,305	0,003359599	NM_001012152.2	CUST_1738_PI421866198	NM_001012152	Tbc1d14	TBC1 domain family, member 14	ENSRNOG00000006400	360956
41932	4048	3284	305	355	1,235	0,305	0,006444087	NM_001107600.1	CUST_7497_PI421866198	NM_001107600	Fbxw4	F-box and WD repeat domain containing 4	ENSRNOG00000046211	309444
43785	19228	15473	2463	458	1,235	0,304	0,008680136	NM_001017375.1	CUST_15110_PI421866198	NM_001017375	Mphosph8	M-phase phosphoprotein 8	ENSRNOG00000051238	290270
42772	17653	14291	1975	1430	1,234	0,303	0,012841413	NM_001024267.1	CUST_1023_PI421866198	NM_001024267	MGC109340	similar to Microsomal signal peptidase 23 kDa subunit (SPase 22 kDa subunit) (SPC22/23)	ENSRNOG00000049355	100360464
33773	5317	4365	232	784	1,234	0,303	0,025686169	NM_001134581.1	CUST_6521_PI421866198	NM_001134581	C2cd5	C2 calcium-dependent domain containing 5	ENSRNOG00000014382	362461
2482	5136	4150	654	319	1,233	0,302	0,011040176	NM_001009974.1	CUST_13917_PI421866198	NM_001009974	NA	NA	NA	NA
41491	126463	102182	15885	8119	1,233	0,302	0,012365438	NM_001033679.1	CUST_8199_PI421866198	NM_001033679	Prkar1b	protein kinase cAMP-dependent type 1 regulatory subunit beta	ENSRNOG00000028733	25521
43120	39123	31679	4843	3329	1,233	0,302	0,015904047	NM_001105837.1	CUST_12527_PI421866198	NM_001105837	Eif1	eukaryotic translation initiation factor 1	ENSRNOG00000033765	287703
40750	1310	1061	207	161	1,233	0,302	0,048158171	NM_001040128.1	CUST_12331_PI421866198	NM_001040128	Rmnd1	required for meiotic nuclear division 1 homolog	ENSRNOG00000019501	292268
8800	94	77	11	14	1,231	0,299	0,039709075	NM_001107907.2	CUST_5828_PI421866198	NM_001107907	Fam46b	family with sequence similarity 46, member B	ENSRNOG00000056153	313019
4511	3	2	0	0	1,23	0,299	0,014654257	MRAK028953	MRAK028953_P1	MRAK028953	NA	NA	NA	NA
10175	34	28	4	5	1,23	0,299	0,035616445	NM_001003929.1	CUST_2926_PI421866198	NM_001003929	Cntfr	ciliary neurotrophic factor receptor	ENSRNOG00000047307	313173
13520	974	791	146	122	1,23	0,299	0,045629444	NM_173337.1	CUST_2440_PI421866198	NM_173337	Camk2n1	calcium/calmodulin-dependent protein kinase II inhibitor 1	ENSRNOG00000016322	287005
1637	2459	1994	380	258	1,228	0,296	0,043525199	NM_001025684.1	CUST_10398_PI421866198	NM_001025684	Pex11b	peroxisomal biogenesis factor 11 beta	ENSRNOG00000021216	310682
41907	1095	890	143	98	1,227	0,295	0,028053168	NM_030829.1	CUST_13815_PI421866198	NM_030829	Grk5	G protein-coupled receptor kinase 5	ENSRNOG00000011439	59075
42690	2900	2381	269	409	1,227	0,296	0,029739476	NM_001115047.2	CUST_8240_PI421866198	NM_001115047	Elp4	NA	NA	NA
2279	23447	19120	3236	2720	1,227	0,295	0,039609086	NM_022536.1	CUST_2555_PI421866198	NM_022536	Ppib	peptidylprolyl isomerase B	ENSRNOG00000016781	64367
38090	3879	3192	399	546	1,226	0,294	0,042001631	NM_001107924.1	CUST_5810_PI421866198	NM_001107924	Slc35a1	solute carrier family 35 member A1	ENSRNOG00000008908	313139
37681	20657	16786	3208	2164	1,226	0,294	0,046581665	NM_001009542.1	CUST_929_PI421866198	NM_001009542	Pdcd10	programmed cell death 10	ENSRNOG00000010147	494345
2430	64	52	7	5	1,224	0,292	0,016855337	NM_001107764.2	CUST_6935_PI421866198	NM_001107764	Pla2g4b	phospholipase A2 group IVB	ENSRNOG00000007447	311341
42088	31508	25831	2313	2627	1,222	0,289	0,007267155	NM_001011890.1	CUST_881_PI421866198	NM_001011890	Rnps1	RNA binding protein with serine rich domain 1	ENSRNOG00000008703	287113
42503	5531	4539	557	600	1,222	0,289	0,022063434	NM_001024747.1	CUST_13620_PI421866198	NM_001024747	Phf10	PHD finger protein 10	ENSRNOG00000061312	292404
36558	1969	1602	305	143	1,221	0,288	0,027901841	NM_053541.1	CUST_3303_PI421866198	NM_053541	Lrp3	LDL receptor related protein 3	ENSRNOG00000011451	89787
5980	60	49	7	7	1,221	0,289	0,029498497	NM_001106877.1	CUST_8858_PI421866198	NM_001106877	Rfx2	regulatory factor X2	ENSRNOG00000045846	301121
37669	1703	1389	233	145	1,221	0,288	0,033508323	NM_001004277.2	CUST_3654_PI421866198	NM_001004277	Pla2g15	phospholipase A2, group XV	ENSRNOG00000019859	361401
40378	143671	117365	22655	15080	1,221	0,288	0,042740753	NM_022949.1	CUST_126_PI421866198	NM_022949	Rpl14	ribosomal protein L14	ENSRNOG00000019007	65043
41496	16256	13411	1031	1996	1,22	0,287	0,016154419	NM_001042354.1	CUST_4921_PI421866198	NM_001042354	Camk2b	calcium/calmodulin-dependent protein kinase II beta	ENSRNOG00000052080	24245
4687	6335	5223	501	782	1,22	0,287	0,020313788	NM_212458.1	CUST_12765_PI421866198	NM_212458	Agpat1	1-acylglycerol-3-phosphate O-acyltransferase 1	ENSRNOG00000000437	406165
42261	16627	13629	1540	1069	1,219	0,285	0,008070316	NM_001012098.1	CUST_2319_PI421866198	NM_001012098	Recql	RecQ like helicase	ENSRNOG00000012602	312824
33508	7413	6089	642	608	1,219	0,286	0,010650776	NM_001004418.1	CUST_2575_PI421866198	NM_001004418	Tacc2	transforming, acidic coiled-coil containing protein 2	ENSRNOG00000020457	309025
16286	1824	1484	317	137	1,218	0,284	0,048427031	NM_030989.3	CUST_12913_PI421866198	NM_030989	Tp53	tumor protein p53	ENSRNOG00000010756	24842
42262	17699	14472	2073	549	1,217	0,283	0,010314944	NM_001135813.1	CUST_1212_PI421866198	NM_001135813	Med31	mediator complex subunit 31	ENSRNOG00000014618	287475
44652	9680	7918	1207	648	1,217	0,284	0,021280217	NM_001014236.1	CUST_9500_PI421866198	NM_001014236	Ssbp4	single stranded DNA binding protein 4	ENSRNOG00000019698	364534
34860	22197	18356	1930	2804	1,216	0,282	0,022781725	NM_013106.1	CUST_10128_PI421866198	NM_013106	Gnai3	G protein subunit alpha i3	ENSRNOG00000019465	25643
4624	1084	883	180	73	1,216	0,282	0,047720125	NM_001008346.1	CUST_5585_PI421866198	NM_001008346	Rrp8	ribosomal RNA processing 8, methyltransferase, homolog (yeast)	ENSRNOG00000018766	308911
11099	1690	1383	255	163	1,216	0,283	0,048379667	NM_001106550.1	CUST_10797_PI421866198	NM_001106550	Nkain4	Sodium/potassium transporting ATPase interacting 4	ENSRNOG00000031834	296469
44814	29882	24566	3379	2432	1,215	0,281	0,019012968	NM_182668.2	CUST_9750_PI421866198	NM_182668	Nit1	nitrilase 1	ENSRNOG00000003881	289222
3396	3676	3031	404	389	1,215	0,281	0,028566876	BC081997	BC081997_P1	BC081997	NA	lncRNA	NA	NA
41146	231551	188797	36471	9136	1,215	0,28	0,031134272	NM_017236.1	CUST_12056_PI421866198	NM_017236	Pebp1	phosphatidylethanolamine binding protein 1	ENSRNOG00000001136	29542
40225	28843	23752	2715	2109	1,214	0,279	0,010735316	NM_031152.2	CUST_14751_PI421866198	NM_031152	Rab11a	RAB11a, member RAS oncogene family	ENSRNOG00000011302	81830
6644	18	15	3	2	1,212	0,277	0,042577583	XR_006775	XR_006775_P1	XR_006775	NA	lncRNA	NA	NA
42237	3805	3098	689	135	1,212	0,277	0,048909444	NM_001014762.1	CUST_7319_PI421866198	NM_001014762	Pdrg1	p53 and DNA damage regulated 1	ENSRNOG00000008845	296278
45150	27414	22545	3443	1907	1,211	0,277	0,021649496	NM_001004248.1	CUST_3708_PI421866198	NM_001004248	Tmem30a	transmembrane protein 30A	ENSRNOG00000010895	300857
43364	59496	48992	7775	4923	1,211	0,276	0,027699111	NM_001004208.1	CUST_3830_PI421866198	NM_001004208	Cacybp	calcyclin binding protein	ENSRNOG00000002572	289144
43955	51455	42124	7751	823	1,21	0,275	0,025416691	NM_001106647.2	CUST_10053_PI421866198	NM_001106647	Bag1	Bcl2 associated athanogene 1	ENSRNOG00000008277	297994
43279	34776	28743	2763	2159	1,209	0,274	0,007321787	NM_030842.1	CUST_13782_PI421866198	NM_030842	Itga7	integrin subunit alpha 7	ENSRNOG00000007905	81008
40299	6257	5181	315	291	1,208	0,273	0,002339948	NM_001103352.1	CUST_7044_PI421866198	NM_001103352	Kifc3	kinesin family member C3	ENSRNOG00000014087	307644
1883	14726	12234	935	1354	1,208	0,272	0,010992144	BC167761	BC167761_P1	BC167761	NA	lncRNA	NA	NA

10854	268	221	38	14	1,208	0,273	0,027032843	MRAK038640	MRAK038640_P1	MRAK038640	NA	lncRNA	NA	NA
41751	25282	20926	2739	2045	1,207	0,271	0,018497698	NM_001013897.1	CUST_9106_PI421866198	NM_001013897	Hikeshi	Hikeshi, heat shock protein nuclear import factor	ENSRNOG00000017383	293103
41748	13750	11328	1848	831	1,207	0,272	0,025370379	NM_139253.1	CUST_3811_PI421866198	NM_139253	Mcfd2	multiple coagulation factor deficiency 2	ENSRNOG00000015059	246117
19264	19	16	2	2	1,207	0,271	0,025606762	XR_008931	XR_008931_P1	XR_008931	NA	lncRNA	NA	NA
43812	5211	4296	676	207	1,205	0,269	0,02229302	NM_001009689.1	CUST_297_PI421866198	NM_001009689	Cdc42ep2	CDC42 effector protein 2	ENSRNOG00000020904	309175
38865	41250	34437	3607	5222	1,205	0,269	0,035280065	NM_001108492.1	CUST_5324_PI421866198	NM_001108492	NA	NA	NA	NA
41156	11123	9247	1025	1071	1,204	0,268	0,019929577	NM_012932.1	CUST_8588_PI421866198	NM_012932	Crmp1	collapsin response mediator protein 1	ENSRNOG00000004781	25415
42778	10491	8707	949	562	1,203	0,266	0,009690051	uc.45-	uc.45-_P1	uc-	NA	lncRNA	NA	NA
41493	11309	9399	1099	913	1,203	0,267	0,018174647	NM_012948.1	CUST_8549_PI421866198	NM_012948	Emd	emerin	ENSRNOG00000058882	25437
18352	327	272	42	26	1,202	0,265	0,032942078	U03630	U03630_P1	U03630	NA	lncRNA	NA	NA
42163	4232	3523	368	264	1,2	0,263	0,010915488	NM_001106143.1	CUST_13613_PI421866198	NM_001106143	Tcof1	treacle ribosome biogenesis factor 1	ENSRNOG00000026108	291571
40751	18985	15817	1658	950	1,199	0,261	0,007850359	NM_030875.1	CUST_13669_PI421866198	NM_030875	Scn1a	sodium voltage-gated channel alpha subunit 1	ENSRNOG00000053122	81574
45065	14752	12259	1769	879	1,199	0,261	0,021597287	NM_057197.1	CUST_7781_PI421866198	NM_057197	Decr1	2,4-dienoyl-CoA reductase 1	ENSRNOG00000008236	117543
7585	389	325	46	46	1,199	0,262	0,049271701	NM_021853.1	CUST_4067_PI421866198	NM_021853	Kcnt1	potassium sodium-activated channel subfamily T member 1	ENSRNOG00000017283	60444
44818	8250	6887	539	380	1,197	0,26	0,004446373	NM_017201.1	CUST_12186_PI421866198	NM_017201	Ahcy	adenosylhomocysteinase	ENSRNOG00000017777	29443
43867	5081	4234	646	371	1,196	0,258	0,03047568	NM_001014207.1	CUST_9578_PI421866198	NM_001014207	Taf1d	TATA-box binding protein associated factor, RNA polymerase I subunit D	ENSRNOG00000010921	363017
37343	22715	18994	2685	2287	1,196	0,258	0,040903646	NM_001106733.1	CUST_9337_PI421866198	NM_001106733	Trappc6b	trafficking protein particle complex 6B	ENSRNOG00000004103	299075
38596	1197	1002	158	132	1,196	0,258	0,047458784	NM_134401.2	CUST_3627_PI421866198	NM_134401	Crtac1	cartilage acidic protein 1	ENSRNOG00000015220	171438
42758	17918	14933	2049	734	1,194	0,256	0,017910899	NM_001011935.2	CUST_415_PI421866198	NM_001011935	Xpo6	exportin 6	ENSRNOG00000016722	293476
9487	92	77	8	10	1,194	0,256	0,030074109	MRAK132631	MRAK132631_P1	MRAK132631	NA	lncRNA	NA	NA
44300	28684	24086	2792	3005	1,194	0,255	0,033858685	NM_053598.1	CUST_3167_PI421866198	NM_053598	Nudt4	nudix hydrolase 4	ENSRNOG00000009094	94267
2719	917	777	49	134	1,194	0,255	0,045917927	MRAK133434	MRAK133434_P1	MRAK133434	NA	lncRNA	NA	NA
44811	60009	50290	5637	4454	1,193	0,254	0,016753465	NM_080697.2	CUST_952_PI421866198	NM_080697	Dynll2	dynein light chain LC8-type 2	ENSRNOG00000008921	140734
39711	7984	6768	338	1194	1,193	0,255	0,041059292	NM_032072.2	CUST_11531_PI421866198	NM_032072	Nae1	NEDD8 activating enzyme E1 subunit 1	ENSRNOG00000033133	84019
40812	18159	15222	2136	1683	1,192	0,253	0,038090282	NM_001108291.1	CUST_6668_PI421866198	NM_001108291	Luc7l3	LUC7-like 3 pre-mRNA splicing factor	ENSRNOG00000002835	360602
450	13578	11447	1300	1575	1,192	0,253	0,045177907	NM_001031646.1	CUST_15117_PI421866198	NM_001031646	Stoml2	stomatin like 2	ENSRNOG00000009535	298203
31633	181572	152259	22237	14840	1,19	0,25	0,036356264	NM_001033955.1	CUST_6984_PI421866198	NM_001033955	Calca	calcitonin-related polypeptide alpha	ENSRNOG00000011130	24241
6002	131	111	10	17	1,19	0,252	0,044965442	U75923	U75923_P1	U75923	NA	lncRNA	NA	NA
43367	8129	6835	500	409	1,189	0,25	0,005630034	NM_053410.1	CUST_4037_PI421866198	NM_053410	Gnpat	glyceronephosphate O-acyltransferase	ENSRNOG00000019205	84470
5676	8952	7478	1281	416	1,189	0,25	0,033584598	MRAK020287	MRAK020287_P1	MRAK020287	NA	lncRNA	NA	NA
34811	532	449	50	54	1,188	0,249	0,03408618	BC098000	BC098000_P1	BC098000	NA	lncRNA	NA	NA
45148	23226	19545	1383	587	1,187	0,247	0,003668503	NM_053474.1	CUST_3896_PI421866198	NM_053474	Ppp1r9b	protein phosphatase 1, regulatory subunit 9B	ENSRNOG00000052113	84686
40802	13580	11465	586	870	1,186	0,246	0,006044321	NM_001107747.2	CUST_6971_PI421866198	NM_001107747	Arhgap1	Rho GTPase activating protein 1	ENSRNOG00000016610	311193
41425	79478	67354	4563	8510	1,186	0,247	0,025213213	NM_001008309.1	CUST_5733_PI421866198	NM_001008309	Btf3	basic transcription factor 3	ENSRNOG00000016912	294680
10326	35	30	5	2	1,186	0,246	0,038213808	MRAK045275	MRAK045275_P1	MRAK045275	NA	lncRNA	NA	NA
15745	38	32	5	3	1,186	0,247	0,048177568	NM_021678.1	CUST_5244_PI421866198	NM_021678	Camk2n2	calcium/calmodulin-dependent protein kinase II inhibitor 2	ENSRNOG00000038184	59314
42023	56022	47459	1757	4719	1,185	0,244	0,009502005	NM_001008217.1	CUST_6277_PI421866198	NM_001008217	Psma7	proteasome subunit alpha 7	ENSRNOG00000056853	29674
34747	3652	3091	246	297	1,184	0,243	0,016137862	MRuc009aid	MRuc009aid_P1	MRuc009aid	NA	lncRNA	NA	NA
43549	7273	6158	624	710	1,184	0,244	0,031517827	NM_001134789.1	CUST_5195_PI421866198	NM_001134789	Polr2h	RNA polymerase II subunit H	ENSRNOG00000001748	100912534
1537	9278	7853	539	656	1,183	0,242	0,009558902	NM_001106288.1	CUST_12747_PI421866198	NM_001106288	Swap70	SWAP switching B-cell complex 70	ENSRNOG00000009910	293410
44900	9799	8271	950	473	1,181	0,241	0,0167267	NM_133299.1	CUST_7754_PI421866198	NM_133299	Pecr	peroxisomal trans-2-enoyl-CoA reductase	ENSRNOG00000055295	NA
41498	6628	5611	724	599	1,181	0,24	0,037739796	NM_024386.1	CUST_12344_PI421866198	NM_024386	Hmgcl	3-hydroxymethyl-3-methylglutaryl-CoA lyase	ENSRNOG00000009422	79238
42938	9139	7739	656	493	1,18	0,239	0,010034855	NM_130826.2	CUST_14322_PI421866198	NM_130826	Hadha	hydroxyacyl-CoA dehydrogenase/3-ketoacyl-CoA thiolase/enoyl-CoA hydratase (trifunctional protein), alpha subunit	ENSRNOG00000024629	170670
41760	14052	11938	1103	1370	1,18	0,239	0,028088848	NM_013006.1	CUST_10881_PI421866198	NM_013006	Lypla1	lysophospholipase I	ENSRNOG00000008320	25514
9954	15978	13677	617	2125	1,18	0,238	0,044002204	NM_001108194.1	CUST_7207_PI421866198	NM_001108194	Oxsr1	oxidative-stress responsive 1	ENSRNOG00000013136	316064
44041	30908	26212	2234	1366	1,178	0,236	0,009212407	NM_001106993.1	CUST_8318_PI421866198	NM_001106993	Fbxw11	F-box and WD repeat domain containing 11	ENSRNOG00000004395	303024
36394	10626	9049	602	959	1,178	0,236	0,017387808	NM_001011979.2	CUST_329_PI421866198	NM_001011979	Tardbp	TAR DNA binding protein	ENSRNOG00000012455	298648
38288	5719	4843	622	315	1,177	0,235	0,028020674	NM_001108288.1	CUST_6685_PI421866198	NM_001108288	Trim37	tripartite motif-containing 37	ENSRNOG00000006248	360592
44037	14394	12233	719	441	1,176	0,234	0,004018768	NM_001114502.1	CUST_8443_PI421866198	NM_001114502	Sgf29	SAGA complex associated factor 29	ENSRNOG00000019245	293488
41667	6211	5290	249	355	1,176	0,233	0,006369977	NM_001106030.1	CUST_14363_PI421866198	NM_001106030	Parp2	poly (ADP-ribose) polymerase 2	ENSRNOG00000008892	290027
40723	24738	21082	1031	1698	1,176	0,233	0,008764367	NM_001037496.1	CUST_11618_PI421866198	NM_001037496	Otud5	OTU deubiquitinase 5	ENSRNOG00000008764	363452
1572	4182	3547	419	127	1,175	0,233	0,016887975	MRAK032655	MRAK032655_P1	MRAK032655	NA	lncRNA	NA	NA
7216	4237	3610	416	369	1,174	0,231	0,038031645	AY264286	AY264286_P1	AY264286	NA	lncRNA	NA	NA
34693	5590	4751	641	456	1,174	0,231	0,047452228	BC098730	BC098730_P1	BC098730	NA	lncRNA	NA	NA
6872	17507	14929	1458	1233	1,173	0,23	0,019379833	MRAK005120	MRAK005120_P1	MRAK005120	NA	lncRNA	NA	NA
43959	13393	11426	1115	1129	1,173	0,231	0,026793819	NM_001008766.1	CUST_3056_PI421866198	NM_001008766	Ciao1	cytosolic iron-sulfur assembly component 1	ENSRNOG00000012638	29231
39552	6944	5966	337	878	1,173	0,23	0,041982981	NM_053625.1	CUST_2853_PI421866198	NM_053625	Gfm1	G elongation factor, mitochondrial 1	ENSRNOG00000012873	114017
44217	7434	6292	1018	329	1,173	0,231	0,046274648	NM_001108402.1	CUST_5542_PI421866198	NM_001108402	Naxd	NAD(P)HX dehydratase	ENSRNOG00000015021	361185
41572	14044	11960	1369	605	1,171	0,228	0,019548041	NM_001012190.1	CUST_1608_PI421866198	NM_001012190	Ubap1	ubiquitin-associated protein 1	ENSRNOG00000012004	362502
43866	34050	28944	3939	1215	1,171	0,227	0,0281715	NM_001108109.2	CUST_7331_PI421866198	NM_001108109	Asb8	ankyrin repeat and SOCS box-containing 8	ENSRNOG00000010099	315287
44901	9498	8073	1205	560	1,171	0,228	0,043375494	NM_001025054.1	CUST_14628_PI421866198	NM_001025054	LOC500956	hypothetical protein LOC500956	ENSRNOG00000020580	500956
7464	18431	15754	1772	1738	1,171	0,228	0,044877181	NM_022397.3	CUST_3643_PI421866198	NM_022397	Hnrnpf	heterogeneous nuclear ribonucleoprotein F	ENSRNOG00000014562	64200
43952	33299	28388	3478	1843	1,17	0,226	0,02796178	NM_145677.1	CUST_5649_PI421866198	NM_145677	Slc25a25	solute carrier family 25 member 25	ENSRNOG00000014338	246771
42271	46266	39409	5488	1955	1,169	0,225	0,030928836	NM_012518.3	CUST_11110_PI421866198	NM_012518	Calm3	calmodulin 3	ENSRNOG00000004060	24242
6875	1973	1680	228	109	1,169	0,226	0,04085448	NM_031335.3	CUST_13345_PI421866198	NM_031335	Polr2f	RNA polymerase II subunit F	ENSRNOG00000011214	83503
39194	23481	20148	1522	1498	1,166	0,221	0,01523251	NM_001002831.1	CUST_6881_PI421866198	NM_001002831	Rpp21	ribonuclease P/MRP 21 subunit	ENSRNOG00000000786	406230
40400	14440	12414	940	1307	1,166	0,222	0,029307765	NM_001135781.1	CUST_1602_PI421866198	NM_001135781	RGD1305350	similar to RIKEN cDNA 2510039O18	ENSRNOG00000007968	313699
41998	2867	2457	290	206	1,165	0,22	0,035882733	NM_001144851.1	CUST_10595_PI421866198	NM_001144851	B3galnt2	beta-1,3-N-acetylgalactosaminyltransferase 2	ENSRNOG00000016855	291212
40892	24113	20758	1269	1923	1,164	0,219	0,018601655	NM_031139.1	CUST_14794_PI421866198	NM_031139	Usf2	upstream transcription factor 2, c-fos interacting	ENSRNOG00000053725	81817
35706	15005	12945	656	1419	1,164	0,219	0,025204797	NM_001106127.1	CUST_13647_PI421866198	NM_001106127	Prtfdc1	phosphoribosyl transferase domain containing 1	ENSRNOG00000024874	291355
42619	33732	29008	2653	2308	1,163	0,217	0,025217688	NM_001109442.1	CUST_1967_PI421866198	NM_001109442	Erh	enhancer of rudimentary homolog (Drosophila)	ENSRNOG00000004883	681415
35075	26180	22527	1924	1534	1,162	0,216	0,019380376	BC167051	BC167051_P1	BC167051	NA	lncRNA	NA	NA
3652	62304	53637	4824	4386	1,162	0,216	0,025930273	NM_133621.2	CUST_5841_PI421866198	NM_133621	Hopx	HOP homeobox	ENSRNOG00000024689	171160
8416	11	9	1	1	1,162	0,217	0,031031939	NM_001122975.1	CUST_4056_PI421866198	NM_001122975	Lrrc55	leucine rich repeat containing 55	ENSRNOG00000028266	311171
37325	8529	7338	661	409	1,161	0,215	0,018006335	NM_001106092.2	CUST_14177_PI421866198	NM_001106092	Vps36	vacuolar protein sorting 36	ENSRNOG00000012654	290851
41658	76223	65456	6739	1964	1,161	0,215	0,018859646	NM_001012171.1	CUST_1671_PI421866198	NM_001012171	Bscl2	BSCL2, seipin lipid droplet biogenesis associated	ENSRNOG00000052393	NA
44904	13868	11933	1163	785	1,161	0,215	0,023952347	NM_001014147.1	CUST_10228_PI421866198	NM_001014147	Tpgs2	tubulin polyglutamylase complex subunit 2	ENSRNOG00000054118	361301
44214	3834	3304	297	275	1,161	0,215	0,025832998	NM_001024759.1	CUST_13596_PI421866198	NM_001024759	Tmem5	transmembrane protein 5	ENSRNOG00000004421	299841
4222	79518	68559	6915	6547	1,161	0,215	0,038943961	MRAK140299	MRAK140299_P1	MRAK140299	NA	lncRNA	NA	NA
582	8447	7281	507	476	1,16	0,215	0,014299225	BC081716	BC081716_P1	BC081716	NA	lncRNA	NA	NA
41823	27149	23465	1365	2389	1,16	0,215	0,023612287	NM_001106486.2	CUST_11351_PI421866198	NM_001106486	Dnajc10	DnaJ heat shock protein family (Hsp40) member C10	ENSRNOG00000006803	295690
39535	13970	12065	955	1215	1,16	0,215	0,030056336	NM_001079698.1	CUST_14878_PI421866198	NM_001079698	Cxxc1	CXXC finger protein 1	ENSRNOG00000014614	291440
41071	11039	9555	616	1090	1,16	0,214	0,033652177	NM_001025059.1	CUST_14619_PI421866198	NM_001025059	Tbc1d22b	TBC1 domain family, member 22B	ENSRNOG00000059458	NA
42949	42811	36921	2590	2008	1,159	0,213	0,012416844	NM_017348.2	CUST_11314_PI421866198	NM_017348	Slc6a8	solute carrier family 6 member 8	ENSRNOG00000057620	50690
43624	10996	9467	975	445	1,159	0,213	0,021689076	NM_021661.2	CUST_5281_PI421866198	NM_021661	Rgs19	regulator of G-protein signaling 19	ENSRNOG00000016547	59293
36978	3636	3148	236	321	1,158	0,212	0,031149007	NM_001107993.1	CUST_5606_PI421866198	NM_001107993	Ubiad1	UbiA prenyltransferase domain containing 1	ENSRNOG00000009575	313706
11738	718	621	69	63	1,158	0,211	0,049578167	MRAK013469	MRAK013469_P1	MRAK013469	NA	lncRNA	NA	NA
44899	58064	50263	785	3682	1,157	0,211	0,008916396	NM_001033683.1	CUST_8181_PI421866198	NM_001033683	Vat1	vesicle amine transport 1	ENSRNOG00000020684	287721
43365	4426	3815	404	184	1,157	0,211	0,024294064	NM_001047093.1	CUST_5722_PI421866198	NM_001047093	Klhl5	kelch-like family member 5	ENSRNOG00000008421	305351
39283	62998	54548	4120	5039	1,157	0,21	0,027201003	NM_001013244.1	CUST_13264_PI421866198	NM_001013244	Tspan13	tetraspanin 13	ENSRNOG00000005046	366602
41918	987	852	97	54	1,156	0,21	0,031908272	NM_001025726.1	CUST_9791_PI421866198	NM_001025726	Srpk1	SRSF protein kinase 1	ENSRNOG00000000511	361811
37491	12252	10629	651	955	1,155	0,208	0,023020251	NM_001037652.1	CUST_10382_PI421866198	NM_001037652	Zdhhc6	zinc finger, DHHC-type containing 6	ENSRNOG00000036576	361771
34601	78480	68061	5151	5296	1,154	0,206	0,026259086	NM_013214.4	CUST_9374_PI421866198	NM_013214	Acot7	acyl-CoA thioesterase 7	ENSRNOG00000010580	26759
42445	20107	17469	1159	1608	1,153	0,206	0,0272932	NM_001008286.1	CUST_6182_PI421866198	NM_001008286	Mtx2	metaxin 2	ENSRNOG00000001559	288150
40749	2088	1817	131	179	1,152	0,204	0,035518537	NM_001134612.1	CUST_6056_PI421866198	NM_001134612	Slf2	SMC5-SMC6 complex localization factor 2	ENSRNOG00000014482	499360
36815	30692	26691	2099	2396	1,151	0,203	0,031966224	NM_001013935.1	CUST_8777_PI421866198	NM_001013935	Mtfr1l	mitochondrial fission regulator 1-like	ENSRNOG00000016937	298549
42862	33721	29250	3360	1297	1,149	0,2	0,035865316	NM_001111057.1	CUST_6452_PI421866198	NM_001111057	Adarb1	adenosine deaminase, RNA-specific, B1	ENSRNOG00000001227	25367
42286	65824	57267	4468	2610	1,148	0,199	0,017898817	NM_001079936.1	CUST_12954_PI421866198	NM_001079936	Rab5b	RAB5B, member RAS oncogene family	ENSRNOG00000006130	288779
23636	35573	31045	2653	2590	1,147	0,197	0,040328866	MRAK020023	MRAK020023_P1	MRAK020023	NA	lncRNA	NA	NA
41183	4040	3522	300	163	1,146	0,196	0,022512239	NM_001008364.1	CUST_5559_PI421866198	NM_001008364	Snx24	sorting nexin 24	ENSRNOG00000017488	361328
4573	272	237	29	9	1,146	0,197	0,044658749	NM_001106535.1	CUST_10828_PI421866198	NM_001106535	Aar2	AAR2 splicing factor homolog	ENSRNOG00000020086	296312
5548	2	2	0	0	1,145	0,195	0,048686346	NM_175592.2	CUST_9356_PI421866198	NM_175592	Cacna2d2	calcium voltage-gated channel auxiliary subunit alpha2delta 2	ENSRNOG00000015835	300992
36734	25652	22511	774	2410	1,144	0,194	0,032681474	NM_172074.2	CUST_7555_PI421866198	NM_172074	Timm10	translocase of inner mitochondrial membrane 10	ENSRNOG00000007883	64464
44019	16095	14084	1262	1239	1,143	0,193	0,045767293	NM_022252.2	CUST_4451_PI421866198	NM_022252	Slc33a1	solute carrier family 33 member 1	ENSRNOG00000010023	64018
36304	33589	29584	1094	3592	1,141	0,191	0,047241923	NM_057153.2	CUST_7843_PI421866198	NM_057153	Oxr1	oxidation resistance 1	ENSRNOG00000056487	117520
34944	8901	7817	645	763	1,141	0,19	0,049892487	NM_012655.2	CUST_10296_PI421866198	NM_012655	Sp1	Sp1 transcription factor	ENSRNOG00000014084	24790
38773	12420	10885	907	506	1,14	0,189	0,025452326	NM_022404.2	CUST_3212_PI421866198	NM_022404	Mlst8	MTOR associated protein, LST8 homolog	ENSRNOG00000009667	64226
8620	18679	16382	1509	1317	1,14	0,189	0,048786269	BC158739	BC158739_P1	BC158739	NA	lncRNA	NA	NA
16870	9494	8326	543	275	1,139	0,188	0,016522957	BC098753	BC098753_P1	BC098753	NA	lncRNA	NA	NA
44562	62949	55236	3470	2127	1,139	0,188	0,017499893	NM_001100517.1	CUST_78_PI421866198	NM_001100517	Aars	alanyl-tRNA synthetase	ENSRNOG00000018404	292023
41927	5506	4863	164	441	1,135	0,183	0,031752131	NM_001107633.1	CUST_7461_PI421866198	NM_001107633	Gcc2	GRIP and coiled-coil domain containing 2	ENSRNOG00000000823	309798
43699	4632	4081	350	259	1,134	0,182	0,039652974	NM_001029926.1	CUST_11123_PI421866198	NM_001029926	Gli4	GLI family zinc finger 4	ENSRNOG00000021765	500893
43036	33822	29811	2744	1125	1,132	0,179	0,036855694	NM_001111055.1	CUST_6454_PI421866198	NM_001111055	Adarb1	adenosine deaminase, RNA-specific, B1	ENSRNOG00000001227	25367
40465	15007	13268	897	1042	1,132	0,179	0,040325079	NM_001025122.2	CUST_14040_PI421866198	NM_001025122	Cyhr1	cysteine and histidine rich 1	ENSRNOG00000014811	100362155
45001	45142	40007	2704	3280	1,13	0,176	0,047083916	NM_001107817.2	CUST_6421_PI421866198	NM_001107817	Nelfb	negative elongation factor complex member B	ENSRNOG00000009377	311796
44405	6349	5612	495	263	1,129	0,176	0,04222862	NM_022509.1	CUST_2703_PI421866198	NM_022509	Smn1	survival of motor neuron 1, telomeric	ENSRNOG00000018067	64301
42608	30444	26937	2273	1612	1,129	0,175	0,04421109	NM_001108879.1	CUST_2935_PI421866198	NM_001108879	Mrpl36	mitochondrial ribosomal protein L36	ENSRNOG00000023407	364656
41887	4763	4220	329	160	1,127	0,173	0,035260054	NM_133319.1	CUST_7504_PI421866198	NM_133319	Taf2	TATA-box binding protein associated factor 2	ENSRNOG00000034022	170844
44922	15608	13845	1003	703	1,127	0,172	0,035491106	NM_031683.1	CUST_11208_PI421866198	NM_031683	Smc1a	structural maintenance of chromosomes 1A	ENSRNOG00000003139	63996
9186	510	451	40	16	1,127	0,173	0,039076393	AB013454	AB013454_P1	AB013454	NA	lncRNA	NA	NA
39735	18730	16627	916	815	1,126	0,172	0,026212899	NM_183325.2	CUST_8500_PI421866198	NM_183325	Adprh	ADP-ribosylarginine hydrolase	ENSRNOG00000027260	25371
36468	4624	4115	157	346	1,126	0,172	0,038215192	NM_001108789.3	CUST_3406_PI421866198	NM_001108789	Xpo5	exportin 5	ENSRNOG00000019085	363194
5503	32569	28912	2342	1820	1,126	0,171	0,045771139	BC089979	BC089979_P1	BC089979	NA	lncRNA	NA	NA
7259	206504	183925	9249	10918	1,123	0,168	0,03220954	NM_021845.5	CUST_4095_PI421866198	NM_021845	Gnas	GNAS complex locus	ENSRNOG00000047374	24896
38706	14408	12851	615	539	1,121	0,165	0,025208048	NM_013187.1	CUST_9922_PI421866198	NM_013187	Plcg1	phospholipase C, gamma 1	ENSRNOG00000051490	25738
38262	82710	74102	2768	4476	1,117	0,16	0,033616064	NM_017276.2	CUST_11958_PI421866198	NM_017276	Gdi2	GDP dissociation inhibitor 2	ENSRNOG00000018091	29662
12912	66190	59192	4546	1877	1,117	0,159	0,043351221	MRAK050264	MRAK050264_P1	MRAK050264	NA	lncRNA	NA	NA
41838	20667	18504	1348	835	1,116	0,158	0,049955825	NM_001107079.1	CUST_10400_PI421866198	NM_001107079	Klhl22	kelch-like family member 22	ENSRNOG00000001878	303792
39080	86764	77851	5095	2974	1,114	0,155	0,042018734	NM_001013235.1	CUST_13300_PI421866198	NM_001013235	Scoc	short coiled-coil protein	ENSRNOG00000003853	364981
28694	12169	13393	473	418	-1,101	-0,139	0,044846212	NM_001011970.1	CUST_345_PI421866198	NM_001011970	Tm7sf3	transmembrane 7 superfamily member 3	ENSRNOG00000001817	297725
23328	13766	15161	622	394	-1,102	-0,14	0,045539635	NM_001009696.1	CUST_281_PI421866198	NM_001009696	Dcun1d5	defective in cullin neddylation 1 domain containing 5	ENSRNOG00000008390	315405
18030	2	2	0	0	-1,104	-0,143	0,039013204	NM_001000828.1	CUST_13072_PI421866198	NM_001000828	Olr407	olfactory receptor 407	ENSRNOG00000054399	405117
15919	10448	11527	424	354	-1,104	-0,142	0,040807236	NM_001013919.1	CUST_8811_PI421866198	NM_001013919	Galk2	galactokinase 2	ENSRNOG00000009289	296117
14743	25172	27897	1293	1330	-1,108	-0,149	0,04906603	NM_001014232.1	CUST_9505_PI421866198	NM_001014232	Cnrip1	cannabinoid receptor interacting protein 1	ENSRNOG00000005326	364208
22040	4496	4980	242	138	-1,109	-0,149	0,041937027	NM_001004102.1	CUST_4535_PI421866198	NM_001004102	ST7	suppression of tumorigenicity 7	ENSRNOG00000056243	296911
18236	51898	57647	1115	2018	-1,11	-0,151	0,025480312	NM_001024332.1	CUST_541_PI421866198	NM_001024332	Arl8b	ADP-ribosylation factor like GTPase 8B	ENSRNOG00000055860	500282
24834	8411	9368	266	563	-1,113	-0,154	0,039621774	NM_001106050.1	CUST_14299_PI421866198	NM_001106050	Dnajc15	DnaJ heat shock protein family (Hsp40) member C15	ENSRNOG00000009063	290370
26567	8356	9299	483	476	-1,113	-0,155	0,048235886	NM_001109274.1	CUST_3138_PI421866198	NM_001109274	Socs5	suppressor of cytokine signaling 5	ENSRNOG00000028504	500616
26997	55754	62121	1702	2557	-1,114	-0,156	0,027276432	NM_001003709.2	CUST_4165_PI421866198	NM_001003709	Ufc1	ubiquitin-fold modifier conjugating enzyme 1	ENSRNOG00000003706	445268
19330	77651	86622	2869	5765	-1,114	-0,156	0,043454806	NM_134415.2	CUST_3607_PI421866198	NM_134415	Nsa2	NSA2 ribosome biogenesis homolog	ENSRNOG00000016530	171456
15678	1657	1845	99	93	-1,114	-0,156	0,04864789	NM_030858.1	CUST_13746_PI421866198	NM_030858	Smad7	SMAD family member 7	ENSRNOG00000018359	81516
13735	108329	120872	4870	6733	-1,115	-0,158	0,038010484	NM_017152.1	CUST_12683_PI421866198	NM_017152	Rps17	ribosomal protein S17	ENSRNOG00000019106	NA
19164	72391	80869	3105	5030	-1,116	-0,159	0,040941024	NM_057123.1	CUST_7916_PI421866198	NM_057123	Psmc1	proteasome 26S subunit, ATPase 1	ENSRNOG00000003951	117263
21207	7187	8017	481	321	-1,117	-0,159	0,044110292	NM_001100671.1	CUST_14882_PI421866198	NM_001100671	Atl2	atlastin GTPase 2	ENSRNOG00000006523	298757
11268	72171	80653	3511	5619	-1,117	-0,159	0,047478346	NM_031099.1	CUST_15133_PI421866198	NM_031099	Rpl5	ribosomal protein L5	ENSRNOG00000023529	81763
27750	31686	35419	1949	1167	-1,119	-0,162	0,03611266	NM_001106122.1	CUST_13651_PI421866198	NM_001106122	Fam188a	family with sequence similarity 188, member A	ENSRNOG00000016955	291320
18918	59593	66719	2815	3259	-1,12	-0,163	0,032301169	NM_031778.2	CUST_10565_PI421866198	NM_031778	Kcns3	potassium voltage-gated channel, modifier subfamily S, member 3	ENSRNOG00000004899	83588
26213	22412	25084	1425	1474	-1,12	-0,163	0,046926778	NM_001106411.1	CUST_11494_PI421866198	NM_001106411	Gpx8	glutathione peroxidase 8	ENSRNOG00000010461	294744
17794	14154	15852	637	410	-1,121	-0,164	0,022404652	NM_001113357.1	CUST_13145_PI421866198	NM_001113357	Npy1r	neuropeptide Y receptor Y1	ENSRNOG00000014149	29358
25229	27611	31018	838	2041	-1,122	-0,166	0,030832264	NM_001006989.1	CUST_8033_PI421866198	NM_001006989	Shisa5	shisa family member 5	ENSRNOG00000020667	301013
22658	74172	83310	3506	3959	-1,123	-0,168	0,027730715	NM_001105722.1	CUST_13189_PI421866198	NM_001105722	NA	NA	NA	NA
22588	12909	14509	409	490	-1,124	-0,168	0,017124714	NM_001108639.1	CUST_4255_PI421866198	NM_001108639	Sumf1	sulfatase modifying factor 1	ENSRNOG00000006813	362409
3364	14171	15918	933	860	-1,124	-0,169	0,039474454	NM_199410.1	CUST_10130_PI421866198	NM_199410	Wdr12	WD repeat domain 12	ENSRNOG00000017340	363237
28429	10084	11343	578	869	-1,124	-0,169	0,048403515	NM_001014260.2	CUST_9433_PI421866198	NM_001014260	Tceanc2	transcription elongation factor A N-terminal and central domain containing 2	ENSRNOG00000009398	366431
23114	34267	38517	2301	2228	-1,125	-0,169	0,04279352	NM_212542.3	CUST_12124_PI421866198	NM_212542	Ppp1r11	protein phosphatase 1, regulatory (inhibitor) subunit 11	ENSRNOG00000000780	294207
25969	2882	3235	232	128	-1,125	-0,169	0,046358854	NM_001008373.1	CUST_5550_PI421866198	NM_001008373	Srek1ip1	NA	NA	NA
20095	23632	26608	1509	1581	-1,126	-0,172	0,037541769	NM_001025063.1	CUST_14597_PI421866198	NM_001025063	Scrn1	secernin 1	ENSRNOG00000009636	502776
13281	14269	16058	983	932	-1,126	-0,171	0,042087119	NM_001130495.1	CUST_4779_PI421866198	NM_001130495	Ift27	intraflagellar transport 27	ENSRNOG00000006440	300062
26294	13651	15394	352	614	-1,127	-0,173	0,015073131	NM_001130999.1	CUST_1486_PI421866198	NM_001130999	Ap4s1	adaptor-related protein complex 4, sigma 1 subunit	ENSRNOG00000005765	366618
25628	8065	9080	504	433	-1,127	-0,172	0,031808711	NM_001008317.1	CUST_5693_PI421866198	NM_001008317	Erp44	endoplasmic reticulum protein 44	ENSRNOG00000005841	298066
19021	43963	49576	3069	3547	-1,128	-0,173	0,049813918	NM_001037217.1	CUST_13216_PI421866198	NM_001037217	Mmd2	monocyte to macrophage differentiation-associated 2	ENSRNOG00000001113	304301
13142	18484	20877	889	1222	-1,129	-0,175	0,027412286	NM_001013079.1	CUST_14655_PI421866198	NM_001013079	Osbpl2	oxysterol binding protein-like 2	ENSRNOG00000057756	296461
2114	35459	39946	2739	1208	-1,129	-0,175	0,036051898	XR_008011	XR_008011_P1	XR_008011	NA	lncRNA	NA	NA
22157	8000	9046	507	677	-1,13	-0,177	0,040328854	NM_053477.1	CUST_3894_PI421866198	NM_053477	Mlycd	malonyl-CoA decarboxylase	ENSRNOG00000014522	85239
25804	169613	191849	11115	15434	-1,13	-0,176	0,049485469	NM_022864.2	CUST_566_PI421866198	NM_022864	Cplx1	complexin 1	ENSRNOG00000000060	64832
16289	12603	14263	643	972	-1,131	-0,177	0,032767543	NM_001134548.1	CUST_6634_PI421866198	NM_001134548	RGD1305938	similar to expressed sequence AW549877	ENSRNOG00000052775	310362
22237	15035	17029	790	1315	-1,131	-0,178	0,038914593	NM_001105835.1	CUST_12531_PI421866198	NM_001105835	Cisd3	CDGSH iron sulfur domain 3	ENSRNOG00000036894	287661
21567	28965	32771	2005	2282	-1,131	-0,178	0,042137611	NM_019222.1	CUST_6043_PI421866198	NM_019222	Coro1b	coronin 1B	ENSRNOG00000021828	29474
27253	42739	48408	1837	1929	-1,133	-0,18	0,016054687	NM_001009703.1	CUST_15195_PI421866198	NM_001009703	Tmed9	transmembrane p24 trafficking protein 9	ENSRNOG00000021882	361207
17817	117107	132870	4383	9702	-1,133	-0,18	0,026339464	NM_053330.1	CUST_4727_PI421866198	NM_053330	Rpl21	ribosomal protein L21	ENSRNOG00000029410	108349002
28792	24743	28035	1444	1552	-1,133	-0,18	0,027648824	NM_001108318.2	CUST_6157_PI421866198	NM_001108318	Atp6v1a	ATPase H+ transporting V1 subunit A	ENSRNOG00000001992	685232
13812	17761	20162	961	1781	-1,133	-0,18	0,044796894	NM_019385.2	CUST_5210_PI421866198	NM_019385	Gorasp1	golgi reassembly stacking protein 1	ENSRNOG00000018047	56082
24817	1637	1861	77	185	-1,133	-0,18	0,047200144	NM_057185.2	CUST_7805_PI421866198	NM_057185	Folh1	folate hydrolase 1	ENSRNOG00000013770	85309
18997	55378	62769	3075	3264	-1,134	-0,181	0,024027015	NM_001106781.2	CUST_9285_PI421866198	NM_001106781	Ndufa12	NADH:ubiquinone oxidoreductase subunit A12	ENSRNOG00000007407	299739
12909	6622	7512	346	444	-1,134	-0,182	0,025479353	MRuc007hgv	MRuc007hgv_P1	MRuc007hgv	NA	lncRNA	NA	NA
17630	27171	30804	1824	792	-1,135	-0,183	0,020699673	NM_001105941.1	CUST_11702_PI421866198	NM_001105941	Bloc1s1	biogenesis of lysosomal organelles complex-1, subunit 1	ENSRNOG00000007784	288785
14465	26404	29854	2597	700	-1,135	-0,182	0,043407175	NM_001108330.1	CUST_6129_PI421866198	NM_001108330	Lamtor4	late endosomal/lysosomal adaptor, MAPK and MTOR activator 4	ENSRNOG00000027028	360776
9380	2909	3299	253	211	-1,136	-0,184	0,04716002	NM_001108265.1	CUST_6750_PI421866198	NM_001108265	Nagpa	N-acetylglucosamine-1-phosphodiester alpha-N-acetylglucosaminidase	ENSRNOG00000002895	360476
26148	50266	57286	2542	5799	-1,136	-0,184	0,049555539	NM_001004083.1	CUST_4993_PI421866198	NM_001004083	Egln2	egl-9 family hypoxia-inducible factor 2	ENSRNOG00000020947	308457
23031	190447	217052	4198	16593	-1,137	-0,186	0,020305074	NM_017101.1	CUST_12868_PI421866198	NM_017101	Ppia	peptidylprolyl isomerase A	ENSRNOG00000027864	25518
18325	8697	9869	761	582	-1,137	-0,185	0,044998145	NM_001108812.1	CUST_3035_PI421866198	NM_001108812	Ubxn6	UBX domain protein 6	ENSRNOG00000048509	363332
16960	7130	8100	606	577	-1,137	-0,185	0,046518548	NM_001034994.1	CUST_3426_PI421866198	NM_001034994	Rwdd4	RWD domain containing 4	ENSRNOG00000022500	NA
29915	55132	62728	2117	2776	-1,138	-0,186	0,013425734	NM_001106704.1	CUST_9406_PI421866198	NM_001106704	Cox7a2l	cytochrome c oxidase subunit 7A2 like	ENSRNOG00000004526	298762
24454	48225	54994	1229	4307	-1,138	-0,186	0,022620261	NM_001100942.1	CUST_12878_PI421866198	NM_001100942	Pomp	proteasome maturation protein	ENSRNOG00000048470	288455
29957	15618	17774	1198	1246	-1,138	-0,187	0,037404651	NM_022545.3	CUST_2501_PI421866198	NM_022545	Prpsap1	phosphoribosyl pyrophosphate synthetase-associated protein 1	ENSRNOG00000010083	64390
28000	48206	54819	4061	3778	-1,138	-0,187	0,042070448	NM_001009692.1	CUST_287_PI421866198	NM_001009692	Sh3glb2	SH3 domain-containing GRB2-like endophilin B2	ENSRNOG00000017295	311848
30051	3544	4028	298	259	-1,138	-0,186	0,043647907	NM_001134500.1	CUST_6764_PI421866198	NM_001134500	LOC288978	hypothetical LOC288978	ENSRNOG00000045570	288978
13558	10790	12283	510	391	-1,139	-0,188	0,012904917	NM_019213.2	CUST_6069_PI421866198	NM_019213	Jtb	jumping translocation breakpoint	ENSRNOG00000016379	29439
14514	18059	20605	856	1753	-1,139	-0,187	0,032409166	NM_001025408.1	CUST_11944_PI421866198	NM_001025408	Mrps25	mitochondrial ribosomal protein S25	ENSRNOG00000010912	297459
34140	19122	21811	1256	2029	-1,139	-0,187	0,046627096	NM_001012113.1	CUST_1888_PI421866198	NM_001012113	Ptdss1	phosphatidylserine synthase 1	ENSRNOG00000052289	314553
21216	15744	17962	1083	1698	-1,139	-0,188	0,04942162	NM_001100519.1	CUST_76_PI421866198	NM_001100519	Gga2	golgi associated, gamma adaptin ear containing, ARF binding protein 2	ENSRNOG00000018599	293455
21465	26311	30023	763	1538	-1,14	-0,189	0,012044347	NM_053411.1	CUST_4036_PI421866198	NM_053411	Snx1	sorting nexin 1	ENSRNOG00000017029	84471
17378	1647	1872	154	74	-1,14	-0,189	0,039114268	NM_001014093.1	CUST_10899_PI421866198	NM_001014093	Parp16	poly (ADP-ribose) polymerase family, member 16	ENSRNOG00000029127	NA
11355	31755	36331	1450	3867	-1,14	-0,189	0,040620743	NM_031618.1	CUST_11335_PI421866198	NM_031618	Nckap1	NCK-associated protein 1	ENSRNOG00000007518	58823
24966	9008	10297	524	1064	-1,14	-0,189	0,046631277	NM_001033707.1	CUST_7922_PI421866198	NM_001033707	Arfgap2	ADP-ribosylation factor GTPase activating protein 2	ENSRNOG00000014429	362162
19197	15310	17465	870	746	-1,141	-0,191	0,016388458	BC090315	BC090315_P1	BC090315	NA	lncRNA	NA	NA
20111	12721	14504	784	496	-1,141	-0,191	0,016701369	NM_001014242.1	CUST_9483_PI421866198	NM_001014242	Isoc1	isochorismatase domain containing 1	ENSRNOG00000019711	364879
16192	57325	65527	1966	5215	-1,141	-0,19	0,023611646	NM_001108906.1	CUST_2258_PI421866198	NM_001108906	Chmp2a	charged multivesicular body protein 2A	ENSRNOG00000043328	365191
781	5296	6032	442	371	-1,141	-0,19	0,036665762	MRAK164296	MRAK164296_P1	MRAK164296	NA	lncRNA	NA	NA
21986	40970	46841	2531	4668	-1,141	-0,19	0,049263014	NM_001100515.1	CUST_81_PI421866198	NM_001100515	Slc25a46	solute carrier family 25, member 46	ENSRNOG00000017091	291709
17969	3045	3480	222	330	-1,141	-0,191	0,049942134	NM_001106643.2	CUST_10057_PI421866198	NM_001106643	Rars2	arginyl-tRNA synthetase 2, mitochondrial	ENSRNOG00000008643	297969
13409	8444	9650	493	784	-1,142	-0,191	0,031947102	BC086544	BC086544_P1	BC086544	NA	lncRNA	NA	NA
20114	34636	39669	1937	4183	-1,142	-0,192	0,042074408	NM_001106828.1	CUST_8910_PI421866198	NM_001106828	Parp6	poly (ADP-ribose) polymerase family, member 6	ENSRNOG00000011199	300759
22268	16503	18874	1131	1788	-1,142	-0,191	0,042988031	NM_001100782.1	CUST_14190_PI421866198	NM_001100782	Nudt16l1	nudix hydrolase 16 like 1	ENSRNOG00000003224	497867
28851	2	2	0	0	-1,143	-0,193	0,017837733	NM_001099471.1	CUST_13468_PI421866198	NM_001099471	Vom2r26	NA	NA	NA
26662	80	92	7	6	-1,143	-0,193	0,039014786	NM_001108242.1	CUST_6783_PI421866198	NM_001108242	Slc9a7	solute carrier family 9 member A7	ENSRNOG00000004150	317170
12664	4114	4685	406	190	-1,143	-0,193	0,042210617	NM_001105786.1	CUST_13067_PI421866198	NM_001105786	B9d1	B9 domain containing 1	ENSRNOG00000047790	108348055
23515	64049	72994	6335	4004	-1,143	-0,193	0,04464598	NM_001031642.1	CUST_15121_PI421866198	NM_001031642	Serpinb1a	serpin family B member 1A	ENSRNOG00000016581	291091
23346	131537	150983	6158	17368	-1,143	-0,192	0,049796052	NM_012554.2	CUST_11040_PI421866198	NM_012554	Eno1	enolase 1	ENSRNOG00000017895	24333
17374	36945	42212	3015	1928	-1,145	-0,195	0,025861021	NM_019153.3	CUST_6630_PI421866198	NM_019153	Fbln5	fibulin 5	ENSRNOG00000050539	29158
28347	6328	7241	458	441	-1,145	-0,195	0,026414524	NM_001024313.1	CUST_623_PI421866198	NM_001024313	Zfp133	zinc finger protein 133	ENSRNOG00000027955	499900
18919	40539	46502	1944	3966	-1,145	-0,195	0,028377417	NM_053428.1	CUST_4010_PI421866198	NM_053428	Fgf13	fibroblast growth factor 13	ENSRNOG00000042753	84488
6183	5947	6781	569	223	-1,145	-0,195	0,039161602	NM_017317.2	CUST_11401_PI421866198	NM_017317	Rab27a	RAB27A, member RAS oncogene family	ENSRNOG00000052499	50645
1953	26095	29875	1607	1432	-1,146	-0,196	0,017668534	BC158812	BC158812_P1	BC158812	NA	lncRNA	NA	NA
24828	4274	4884	379	149	-1,146	-0,197	0,027893313	NM_024381.2	CUST_12350_PI421866198	NM_024381	Gk	glycerol kinase	ENSRNOG00000034116	79223
19441	25712	29405	2220	1449	-1,146	-0,197	0,031196373	NM_134349.3	CUST_4291_PI421866198	NM_134349	Mgst1	microsomal glutathione S-transferase 1	ENSRNOG00000007743	171341

19929	13242	15178	1020	1138	-1,146	-0,197	0,033987245	NM_031359.1	CUST_13280_PI421866198	NM_031359	Mkln1	muskelin 1	ENSRNOG00000054514	83536
27755	7582	8689	614	752	-1,146	-0,196	0,042314886	NM_001007666.1	CUST_7100_PI421866198	NM_001007666	Bcs1l	BCS1 homolog, ubiquinol-cytochrome c reductase complex chaperone	ENSRNOG00000016754	301514
21110	34307	39421	1725	3198	-1,147	-0,198	0,024413981	NM_022599.2	CUST_2328_PI421866198	NM_022599	Synj2bp	synaptojanin 2 binding protein	ENSRNOG00000006399	64531
13984	30050	34446	2550	1883	-1,148	-0,199	0,028920129	MRAK013392	MRAK013392_P1	MRAK013392	NA	lncRNA	NA	NA
22920	8144	9355	690	746	-1,149	-0,201	0,040693065	NM_001014131.1	CUST_10258_PI421866198	NM_001014131	Ccdc181	coiled-coil domain containing 181	ENSRNOG00000002860	360867
17215	4424	5088	354	492	-1,149	-0,2	0,046675096	NM_138866.2	CUST_3416_PI421866198	NM_138866	Eif2b5	eukaryotic translation initiation factor 2B subunit 5 epsilon	ENSRNOG00000038160	192234
4619	9706	11129	1026	871	-1,149	-0,2	0,048256288	NM_001135846.1	CUST_1132_PI421866198	NM_001135846	Rrn3	RRN3 homolog, RNA polymerase I transcription factor	ENSRNOG00000003326	304714
39415	15	17	1	1	-1,15	-0,202	0,023776213	MRAK019653	MRAK019653_P1	MRAK019653	NA	lncRNA	NA	NA
22148	3970	4568	318	404	-1,15	-0,202	0,040106784	NM_001108332.1	CUST_6127_PI421866198	NM_001108332	Rcc1l	RCC1 like	ENSRNOG00000001483	360796
18557	2	2	0	0	-1,151	-0,203	0,036719875	XR_006814	XR_006814_P1	XR_006814	NA	NA	NA	NA
18989	29674	34167	1660	1386	-1,152	-0,204	0,011338319	NM_001007150.1	CUST_9250_PI421866198	NM_001007150	Stau2	staufen double-stranded RNA binding protein 2	ENSRNOG00000042458	NA
7421	14760	17074	429	1859	-1,152	-0,204	0,028569266	BC091295	BC091295_P1	BC091295	NA	lncRNA	NA	NA
27674	1878	2163	151	172	-1,152	-0,204	0,034106593	NM_001108089.1	CUST_7615_PI421866198	NM_001108089	Mettl25	methyltransferase like 25	ENSRNOG00000027451	314798
12111	1567	1802	153	118	-1,152	-0,204	0,035367332	NM_001108181.1	CUST_7230_PI421866198	NM_001108181	Acad11	acyl-CoA dehydrogenase family, member 11	ENSRNOG00000010940	315973
20572	11172	12820	1202	610	-1,152	-0,204	0,036630376	NM_001025678.1	CUST_10429_PI421866198	NM_001025678	Fkrp	fukutin related protein	ENSRNOG00000016055	308390
19767	26799	30871	2328	2593	-1,152	-0,204	0,037098244	NM_053926.2	CUST_903_PI421866198	NM_053926	Pip4k2a	phosphatidylinositol-5-phosphate 4-kinase type 2 alpha	ENSRNOG00000016670	NA
14450	21056	24168	2249	1139	-1,152	-0,204	0,03820489	NM_001008721.1	CUST_3093_PI421866198	NM_001008721	Ebna1bp2	EBNA1 binding protein 2	ENSRNOG00000045760	114021
21461	40698	47012	1316	3763	-1,153	-0,205	0,015973894	NM_053862.2	CUST_1368_PI421866198	NM_053862	Lgals8	galectin 8	ENSRNOG00000018046	116641
11849	34262	39626	1246	3767	-1,153	-0,205	0,023644855	NM_053999.2	CUST_719_PI421866198	NM_053999	Ppp2r2a	protein phosphatase 2, regulatory subunit B, alpha	ENSRNOG00000011158	117104
20036	960	1104	89	56	-1,153	-0,206	0,029174563	BC105763	BC105763_P1	BC105763	NA	lncRNA	NA	NA
27343	7201	8308	506	737	-1,153	-0,205	0,031495399	NM_001106131.1	CUST_13640_PI421866198	NM_001106131	Ctdp1	CTD phosphatase subunit 1	ENSRNOG00000061474	291414
23059	1216	1400	114	88	-1,153	-0,205	0,032386775	NM_030849.1	CUST_13771_PI421866198	NM_030849	Bmpr1a	bone morphogenetic protein receptor type 1A	ENSRNOG00000052469	81507
12195	31005	35720	2744	2821	-1,153	-0,205	0,035081401	MRuc007hyr	MRuc007hyr_P1	MRuc007hyr	NA	lncRNA	NA	NA
18895	2406	2764	257	152	-1,153	-0,205	0,042483703	NM_001009649.1	CUST_379_PI421866198	NM_001009649	Lpxn	leupaxin	ENSRNOG00000012611	293783
27072	2443	2811	258	228	-1,153	-0,206	0,047874856	NM_001012141.1	CUST_1766_PI421866198	NM_001012141	Tada2a	transcriptional adaptor 2A	ENSRNOG00000002757	360581
16171	2313	2669	174	195	-1,154	-0,207	0,025684773	BC097338	BC097338_P1	BC097338	NA	lncRNA	NA	NA
14588	12931	14980	711	1761	-1,154	-0,206	0,041541269	NM_001107318.2	CUST_8730_PI421866198	NM_001107318	Gtf2e2	general transcription factor IIE subunit 2	ENSRNOG00000014422	306516
8566	30805	35642	2289	3911	-1,154	-0,207	0,045015238	MRAK017353	MRAK017353_P1	MRAK017353	NA	lncRNA	NA	NA
18711	10564	12163	1083	719	-1,155	-0,207	0,035311936	NM_013222.1	CUST_9345_PI421866198	NM_013222	Gfer	growth factor, augmenter of liver regeneration	ENSRNOG00000013370	27100
24415	3124	3596	345	217	-1,155	-0,208	0,037205056	NM_001100510.1	CUST_85_PI421866198	NM_001100510	Osgep	O-sialoglycoprotein endopeptidase	ENSRNOG00000009333	290028
9309	45	52	3	5	-1,156	-0,209	0,0310157	MRAK140379	MRAK140379_P1	MRAK140379	NA	lncRNA	NA	NA
24351	14805	17143	1148	1727	-1,156	-0,209	0,035033233	NM_001024975.1	CUST_12130_PI421866198	NM_001024975	Morn4	MORN repeat containing 4	ENSRNOG00000027156	293950
23571	1636	1886	166	84	-1,157	-0,211	0,031607243	NM_001024771.1	CUST_13540_PI421866198	NM_001024771	Tnip2	TNFAIP3 interacting protein 2	ENSRNOG00000013805	305451
33787	6638	7655	746	483	-1,157	-0,211	0,042641073	NM_181381.2	CUST_13_PI421866198	NM_181381	Abcg2	ATP-binding cassette, subfamily G (WHITE), member 2	ENSRNOG00000007041	312382
12023	6055	7001	481	372	-1,158	-0,212	0,019966976	NM_001005552.1	CUST_14023_PI421866198	NM_001005552	MGC95208	similar to 4930453N24Rik protein	ENSRNOG00000000720	304176
8358	20344	23505	2019	1625	-1,158	-0,212	0,035550122	BC078936	BC078936_P1	BC078936	NA	lncRNA	NA	NA
26661	4752	5497	445	454	-1,158	-0,212	0,041565504	NM_134460.1	CUST_3510_PI421866198	NM_134460	Ap1g1	NA	NA	NA
18443	6967	8101	424	971	-1,158	-0,211	0,0427676	NM_001110491.1	CUST_7218_PI421866198	NM_001110491	Fbxo27	NA	NA	NA
29891	11039	12813	686	1116	-1,159	-0,213	0,022667297	NM_001106300.1	CUST_12314_PI421866198	NM_001106300	Ate1	arginyltransferase 1	ENSRNOG00000024414	293526
7110	59508	68989	4268	5735	-1,159	-0,213	0,024046075	XR_007663	XR_007663_P1	XR_007663	NA	lncRNA	NA	NA
16150	13562	15747	913	1555	-1,159	-0,213	0,029893356	NM_001106798.2	CUST_9266_PI421866198	NM_001106798	Aasdhppt	aminoadipate-semialdehyde dehydrogenase-phosphopantetheinyl transferase	ENSRNOG00000005795	300328
27216	7978	9210	910	596	-1,159	-0,213	0,044722148	NM_001106013.1	CUST_14400_PI421866198	NM_001106013	Nop14	NOP14 nucleolar protein	ENSRNOG00000012147	289724
18067	826	958	52	63	-1,16	-0,214	0,015874337	NM_001030026.1	CUST_6861_PI421866198	NM_001030026	Ifi30	IFI30, lysosomal thiol reductase	ENSRNOG00000019387	290644
25398	1549	1791	171	97	-1,16	-0,214	0,035093585	NM_001135612.1	CUST_2586_PI421866198	NM_001135612	Hps5	NA	NA	NA
29703	700	809	77	50	-1,16	-0,214	0,041050808	NM_182736.1	CUST_9248_PI421866198	NM_182736	Ccdc50	coiled-coil domain containing 50	ENSRNOG00000001930	288022
10264	21	25	2	3	-1,16	-0,214	0,041895878	MRuc007wzq	MRuc007wzq_P1	MRuc007wzq	NA	lncRNA	NA	NA
23177	21824	25315	1939	1861	-1,161	-0,216	0,029417742	NM_001109118.1	CUST_3964_PI421866198	NM_001109118	Elovl2	ELOVL fatty acid elongase 2	ENSRNOG00000014702	498728
12995	1557	1809	136	176	-1,161	-0,215	0,042750262	NM_001009678.1	CUST_318_PI421866198	NM_001009678	Nepro	nucleolus and neural progenitor protein	ENSRNOG00000013721	303948
22063	6398	7456	457	973	-1,161	-0,215	0,045764372	NM_001105993.1	CUST_11561_PI421866198	NM_001105993	Glmn	glomulin, FKBP associated protein	ENSRNOG00000002054	289437
14512	23744	27605	2176	2945	-1,161	-0,216	0,046451015	NM_001007265.1	CUST_8791_PI421866198	NM_001007265	Os9	OS9, endoplasmic reticulum lectin	ENSRNOG00000025570	362891
2633	10	12	1	0	-1,162	-0,216	0,029279609	MRAK053735	MRAK053735_P1	MRAK053735	NA	lncRNA	NA	NA
26650	131030	152183	13647	14996	-1,162	-0,216	0,047303719	NM_138549.1	CUST_5401_PI421866198	NM_138549	Tecr	trans-2,3-enoyl-CoA reductase	ENSRNOG00000021808	191576
19760	1164	1352	102	91	-1,163	-0,218	0,024930947	NM_198754.2	CUST_11677_PI421866198	NM_198754	Cmtm8	CKLF-like MARVEL transmembrane domain containing 8	ENSRNOG00000011201	301045
34133	29340	33985	3435	2288	-1,163	-0,217	0,04151314	NM_019383.2	CUST_5212_PI421866198	NM_019383	Atp5h	ATP synthase, H+ transporting, mitochondrial Fo complex, subunit d	ENSRNOG00000003626	641434
23009	1984	2307	163	129	-1,164	-0,22	0,017093701	NM_001009424.1	CUST_1510_PI421866198	NM_001009424	Eps15	epidermal growth factor receptor pathway substrate 15	ENSRNOG00000010299	NA
19092	142	165	12	13	-1,164	-0,219	0,025852044	NM_131912.1	CUST_9749_PI421866198	NM_131912	Kcnh7	potassium voltage-gated channel subfamily H member 7	ENSRNOG00000007528	170739
26490	3109	3621	311	379	-1,164	-0,22	0,047578743	NM_001107694.1	CUST_7357_PI421866198	NM_001107694	Rfx5	regulatory factor X5	ENSRNOG00000021012	310659
27786	63	74	2	12	-1,164	-0,219	0,047848658	XR_007118	XR_007118_P1	XR_007118	NA	lncRNA	NA	NA
20360	2877	3342	277	205	-1,165	-0,22	0,028868698	NM_001106426.1	CUST_11479_PI421866198	NM_001106426	Ndufb5	NADH:ubiquinone oxidoreductase subunit B5	ENSRNOG00000011949	294964
19869	2428	2832	209	294	-1,165	-0,22	0,034526805	NM_001107348.1	CUST_8675_PI421866198	NM_001107348	Rreb1	ras responsive element binding protein 1	ENSRNOG00000015701	306873
12174	58	68	6	7	-1,165	-0,22	0,043154425	MRAK037136	MRAK037136_P1	MRAK037136	NA	lncRNA	NA	NA
20992	19645	22954	1626	2822	-1,165	-0,22	0,048852806	NM_001126275.1	CUST_9899_PI421866198	NM_001126275	Ccm2	CCM2 scaffolding protein	ENSRNOG00000060825	305505
23925	15780	18406	1024	1300	-1,166	-0,222	0,014343886	NM_053438.2	CUST_3996_PI421866198	NM_053438	Rnf103	ring finger protein 103	ENSRNOG00000007272	84508
24384	9813	11465	631	1118	-1,166	-0,221	0,024471508	NM_019367.2	CUST_5253_PI421866198	NM_019367	Ppt2	palmitoyl-protein thioesterase 2	ENSRNOG00000000435	54398
24094	28605	33233	3106	1867	-1,166	-0,222	0,030209306	NM_001013115.2	CUST_14144_PI421866198	NM_001013115	Guk1	guanylate kinase 1	ENSRNOG00000002928	303179
14160	10192	11912	778	1296	-1,166	-0,222	0,036719005	NM_001013105.2	CUST_14164_PI421866198	NM_001013105	Fkbp11	FK506 binding protein 11	ENSRNOG00000054775	140940
17644	4516	5256	484	448	-1,166	-0,221	0,043513504	NM_001012043.1	CUST_2482_PI421866198	NM_001012043	Tdrd3	tudor domain containing 3	ENSRNOG00000009034	306066
27420	12092	14134	633	1104	-1,167	-0,223	0,01262235	NM_001108422.2	CUST_5507_PI421866198	NM_001108422	Plxdc2	plexin domain containing 2	ENSRNOG00000000142	361282
21080	1494	1745	107	153	-1,167	-0,223	0,023037832	NM_001106528.1	CUST_10844_PI421866198	NM_001106528	Plagl2	PLAG1 like zinc finger 2	ENSRNOG00000010086	296281
20209	10796	12633	716	1333	-1,167	-0,223	0,028653147	NM_001127450.1	CUST_6266_PI421866198	NM_001127450	Alkbh6	alkB homolog 6	ENSRNOG00000025216	108348106
23082	13514	15852	807	2128	-1,167	-0,222	0,038754786	NM_001013240.1	CUST_13271_PI421866198	NM_001013240	Cdc26	cell division cycle 26	ENSRNOG00000029785	366381
31160	5364	6240	643	517	-1,167	-0,223	0,044580604	NM_001108517.2	CUST_4936_PI421866198	NM_001108517	Rps6ka4	ribosomal protein S6 kinase A4	ENSRNOG00000021117	361715
18822	5166	6005	610	274	-1,168	-0,225	0,036721975	MRAK013679	MRAK013679_P1	MRAK013679	NA	lncRNA	NA	NA
28627	2459	2879	221	341	-1,168	-0,224	0,049383783	NM_001107983.1	CUST_5648_PI421866198	NM_001107983	Zmym1	zinc finger MYM-type containing 1	ENSRNOG00000013855	313604
22098	74088	86306	7137	3170	-1,169	-0,225	0,016714157	NM_031149.1	CUST_14773_PI421866198	NM_031149	Psmc5	proteasome 26S subunit, ATPase 5	ENSRNOG00000010038	81827
24137	36	42	3	5	-1,169	-0,225	0,035845019	NM_001000739.1	CUST_13831_PI421866198	NM_001000739	Olr150	olfactory receptor 150	ENSRNOG00000017143	405001
14955	2733	3188	317	292	-1,169	-0,225	0,046402569	NM_001105742.1	CUST_13151_PI421866198	NM_001105742	Klf3	Kruppel like factor 3	ENSRNOG00000002163	114845
24527	91	107	9	13	-1,169	-0,226	0,047894079	NM_001001509.1	CUST_11418_PI421866198	NM_001001509	Slc25a47	solute carrier family 25, member 47	ENSRNOG00000003020	299316
24622	19328	22664	1356	2391	-1,17	-0,226	0,028024577	NM_130746.1	CUST_14916_PI421866198	NM_130746	Slc5a6	solute carrier family 5 member 6	ENSRNOG00000057628	170551
26486	12994	15237	1000	1378	-1,171	-0,228	0,023114341	NM_177419.1	CUST_4383_PI421866198	NM_177419	Xrcc5	X-ray repair cross complementing 5	ENSRNOG00000016105	363247
15145	12635	14749	1306	564	-1,172	-0,229	0,019181577	NM_139113.1	CUST_4626_PI421866198	NM_139113	Nr2f6	nuclear receptor subfamily 2, group F, member 6	ENSRNOG00000016892	245980
25483	829	972	65	81	-1,172	-0,229	0,020578971	NM_001107022.1	CUST_10558_PI421866198	NM_001107022	Scarf1	scavenger receptor class F, member 1	ENSRNOG00000059618	NA
13760	13134	15376	1430	1508	-1,172	-0,229	0,041859733	NM_133421.1	CUST_7079_PI421866198	NM_133421	Marf1	meiosis arrest female 1	ENSRNOG00000056248	170946
20034	7090	8335	332	685	-1,173	-0,231	0,011185999	NM_001031650.1	CUST_15106_PI421866198	NM_001031650	Mrpl44	mitochondrial ribosomal protein L44	ENSRNOG00000015231	301552
40457	21	24	2	1	-1,173	-0,231	0,025616806	uc.131+	uc.131+_P1	uc+	NA	lncRNA	NA	NA
24869	11562	13656	493	1895	-1,173	-0,231	0,030686567	NM_001105846.1	CUST_12510_PI421866198	NM_001105846	Fmnl1	formin-like 1	ENSRNOG00000003207	287746
26322	25546	29998	2440	3299	-1,173	-0,23	0,041022502	NM_001107908.1	CUST_5827_PI421866198	NM_001107908	Wdtc1	WD and tetratricopeptide repeats 1	ENSRNOG00000008377	313020
28302	3041	3546	436	220	-1,173	-0,231	0,044777727	NM_001009539.1	CUST_932_PI421866198	NM_001009539	B4galt3	beta-1,4-galactosyltransferase 3	ENSRNOG00000003551	494342
15532	2496	2906	355	157	-1,173	-0,23	0,04990749	NM_053729.1	CUST_2091_PI421866198	NM_053729	Tdg	thymine-DNA glycosylase	ENSRNOG00000027124	NA
24553	10901	12849	475	1410	-1,174	-0,232	0,018108776	NM_001106380.1	CUST_12078_PI421866198	NM_001106380	Ube2g2	ubiquitin-conjugating enzyme E2G 2	ENSRNOG00000001222	294331
18093	5216	6120	521	565	-1,174	-0,232	0,031339399	NM_001039197.1	CUST_7382_PI421866198	NM_001039197	Tor1b	torsin family 1, member B	ENSRNOG00000006435	311854
14867	8739	10228	1029	785	-1,174	-0,232	0,039105328	NM_001130559.1	CUST_4138_PI421866198	NM_001130559	Nipal2	NIPA-like domain containing 2	ENSRNOG00000005190	362899
21470	3085	3617	264	140	-1,175	-0,233	0,011284108	NM_001108457.1	CUST_5431_PI421866198	NM_001108457	Ppil4	peptidylprolyl isomerase like 4	ENSRNOG00000015552	361449
7434	2701	3179	188	311	-1,175	-0,232	0,020163519	MRAK050127	MRAK050127_P1	MRAK050127	NA	lncRNA	NA	NA
23783	6038	7078	668	644	-1,175	-0,232	0,040975243	NM_001107111.1	CUST_9806_PI421866198	NM_001107111	Il10rb	interleukin 10 receptor subunit beta	ENSRNOG00000028638	304091
20459	19	22	2	3	-1,175	-0,233	0,048009758	XR_006642	XR_006642_P1	XR_006642	NA	lncRNA	NA	NA
20964	2998	3519	257	179	-1,176	-0,234	0,013072022	NM_001107682.1	CUST_7369_PI421866198	NM_001107682	Nmd3	NMD3 ribosome export adaptor	ENSRNOG00000009310	310512
21796	1899	2223	215	121	-1,176	-0,233	0,027250516	NM_001107228.2	CUST_9163_PI421866198	NM_001107228	Fam53a	family with sequence similarity 53, member A	ENSRNOG00000017548	305461
20841	2	2	0	0	-1,176	-0,234	0,02982906	MRAK034244	MRAK034244_P1	MRAK034244	NA	NA	NA	NA
27684	10175	12008	899	1543	-1,176	-0,233	0,048146106	NM_001107959.1	CUST_5756_PI421866198	NM_001107959	Atpaf1	ATP synthase mitochondrial F1 complex assembly factor 1	ENSRNOG00000010169	313510
24222	29479	34910	829	4395	-1,177	-0,235	0,022898012	NM_032058.2	CUST_11581_PI421866198	NM_032058	Eif2b2	eukaryotic translation initiation factor 2B subunit beta	ENSRNOG00000006467	84005
32118	6340	7470	534	708	-1,177	-0,235	0,023940376	NM_001106731.1	CUST_9339_PI421866198	NM_001106731	Brms1l	breast cancer metastasis-suppressor 1-like	ENSRNOG00000051077	299053
25631	11243	13239	1013	1280	-1,177	-0,236	0,02840841	NM_031093.2	CUST_15139_PI421866198	NM_031093	Rala	RAS like proto-oncogene A	ENSRNOG00000013454	81757
638	25	29	3	1	-1,177	-0,235	0,03882996	MRAK052087	MRAK052087_P1	MRAK052087	NA	lncRNA	NA	NA
29123	11617	13688	885	995	-1,178	-0,237	0,013397418	NM_001106613.2	CUST_10120_PI421866198	NM_001106613	Ppp4r2	protein phosphatase 4, regulatory subunit 2	ENSRNOG00000024647	297486
4404	898	1064	73	157	-1,178	-0,237	0,047591752	NM_053770.1	CUST_1983_PI421866198	NM_053770	Sorbs2	sorbin and SH3 domain containing 2	ENSRNOG00000013391	114901
15468	10550	12438	906	1031	-1,179	-0,238	0,018574761	NM_001007753.1	CUST_6482_PI421866198	NM_001007753	Cyb561d2	cytochrome b561 family, member D2	ENSRNOG00000021887	363137
18568	38227	44849	4510	2260	-1,179	-0,237	0,027058782	NM_012880.1	CUST_8970_PI421866198	NM_012880	Sod3	superoxide dismutase 3, extracellular	ENSRNOG00000003869	25352
5594	30	35	3	4	-1,179	-0,237	0,033142959	MRAK034038	MRAK034038_P1	MRAK034038	NA	lncRNA	NA	NA
18215	4151	4871	552	421	-1,179	-0,237	0,044916238	XR_007127	XR_007127_P1	XR_007127	NA	lncRNA	NA	NA
24534	27761	32554	3463	2464	-1,179	-0,237	0,047127146	NM_053934.2	CUST_897_PI421866198	NM_053934	Pcdha13	NA	NA	NA
26320	5364	6325	573	707	-1,179	-0,238	0,04971288	NM_024137.1	CUST_13861_PI421866198	NM_024137	Hivep2	human immunodeficiency virus type I enhancer binding protein 2	ENSRNOG00000011015	29721
18830	2	2	0	0	-1,18	-0,239	0,026672358	MRAK042350	MRAK042350_P1	MRAK042350	NA	NA	NA	NA
3536	14	16	1	2	-1,18	-0,239	0,034393205	MRAK052029	MRAK052029_P1	MRAK052029	NA	lncRNA	NA	NA
27569	1181	1387	149	116	-1,18	-0,238	0,040678539	NM_022632.2	CUST_1816_PI421866198	NM_022632	Slit2	slit guidance ligand 2	ENSRNOG00000003840	360272
11510	1894	2232	221	231	-1,18	-0,239	0,047153255	NM_001129777.1	CUST_12980_PI421866198	NM_001129777	Rad51c	RAD51 paralog C	ENSRNOG00000006661	497976
16541	4017	4744	274	303	-1,181	-0,24	0,009143127	NM_001009541.1	CUST_930_PI421866198	NM_001009541	Ier2	immediate early response 2	ENSRNOG00000054344	NA
26588	29011	34325	1873	3135	-1,181	-0,24	0,013590049	NM_001106227.1	CUST_12925_PI421866198	NM_001106227	Tmem160	transmembrane protein 160	ENSRNOG00000015304	292654
22662	7116	8432	440	951	-1,181	-0,24	0,020621618	NM_001105790.1	CUST_13056_PI421866198	NM_001105790	Rangrf	RAN guanine nucleotide release factor	ENSRNOG00000004980	287419
20966	4087	4823	378	422	-1,181	-0,24	0,024468196	NM_001024292.1	CUST_992_PI421866198	NM_001024292	Nmrk1	nicotinamide riboside kinase 1	ENSRNOG00000012665	499330
21656	4137	4898	336	536	-1,181	-0,241	0,026914271	NM_031052.1	CUST_15220_PI421866198	NM_031052	Mipep	mitochondrial intermediate peptidase	ENSRNOG00000013876	NA
27573	2017	2369	241	133	-1,181	-0,24	0,03629945	NM_001127541.1	CUST_5710_PI421866198	NM_001127541	Cracr2b	calcium release activated channel regulator 2B	ENSRNOG00000019199	309112
17999	2	3	0	0	-1,181	-0,24	0,041206407	XR_005718	XR_005718_P1	XR_005718	NA	NA	NA	NA
2749	48	57	4	8	-1,181	-0,241	0,045693151	MRAK156405	MRAK156405_P1	MRAK156405	NA	lncRNA	NA	NA
15331	4074	4796	434	191	-1,182	-0,241	0,015664781	NM_001007754.1	CUST_6481_PI421866198	NM_001007754	Rassf1	Ras association domain family member 1	ENSRNOG00000021548	363140
27846	1330	1577	82	172	-1,182	-0,241	0,017071382	NM_001108434.1	CUST_5477_PI421866198	NM_001108434	Fech	ferrochelatase	ENSRNOG00000018053	361338
29238	6271	7410	562	664	-1,182	-0,241	0,021484794	NM_001004272.1	CUST_3659_PI421866198	NM_001004272	Phf23	PHD finger protein 23	ENSRNOG00000017657	360550
26094	6416	7547	842	324	-1,183	-0,243	0,025792482	NM_001108576.1	CUST_4861_PI421866198	NM_001108576	Mrps2	mitochondrial ribosomal protein S2	ENSRNOG00000010164	362094
16538	5216	6169	558	580	-1,183	-0,243	0,02790531	NM_001108647.1	CUST_4235_PI421866198	NM_001108647	Iffo1	intermediate filament family orphan 1	ENSRNOG00000018533	362437
29580	2092	2481	173	296	-1,183	-0,242	0,031463476	NM_001107555.1	CUST_7797_PI421866198	NM_001107555	Ikzf5	IKAROS family zinc finger 5	ENSRNOG00000025651	309031
38394	17	20	2	2	-1,183	-0,243	0,032625711	uc.453-	uc.453-_P1	uc-	NA	lncRNA	NA	NA
22304	6164	7233	907	291	-1,183	-0,242	0,03988939	NM_001039549.1	CUST_5126_PI421866198	NM_001039549	Ugt1a5	UDP glucuronosyltransferase family 1 member A5	ENSRNOG00000018740	24861
18662	892	1049	124	79	-1,183	-0,243	0,046271519	NM_023981.4	CUST_11843_PI421866198	NM_023981	Csf1	colony stimulating factor 1	ENSRNOG00000018659	78965
29064	47291	56123	2957	5373	-1,184	-0,244	0,01334469	NM_001115013.1	CUST_8301_PI421866198	NM_001115013	Selenom	selenoprotein M	ENSRNOG00000061231	498398
12240	3537	4196	67	214	-1,185	-0,245	0,002179147	NM_001024368.1	CUST_501_PI421866198	NM_001024368	Slc10a3	solute carrier family 10, member 3	ENSRNOG00000060580	501665
14507	4488	5295	524	299	-1,185	-0,245	0,024512706	NM_198130.1	CUST_341_PI421866198	NM_198130	Myd88	myeloid differentiation primary response 88	ENSRNOG00000013634	301059
15058	6866	8131	736	763	-1,186	-0,246	0,026654657	MRAK052198	MRAK052198_P1	MRAK052198	NA	lncRNA	NA	NA
20027	36	43	3	6	-1,186	-0,246	0,038309369	NM_021856.1	CUST_4064_PI421866198	NM_021856	Clock	clock circadian regulator	ENSRNOG00000002175	60447
20306	2990	3526	425	269	-1,186	-0,246	0,043952772	NM_001008508.1	CUST_4435_PI421866198	NM_001008508	Tpst2	tyrosylprotein sulfotransferase 2	ENSRNOG00000000664	288719
24606	2480	2934	310	319	-1,186	-0,246	0,046999263	NM_001017472.1	CUST_14574_PI421866198	NM_001017472	LOC497899	similar to hypothetical protein 4930503F14	ENSRNOG00000022555	497899
13955	17682	20985	1188	1278	-1,187	-0,248	0,007010427	NM_019187.1	CUST_6528_PI421866198	NM_019187	Coq3	coenzyme Q3 methyltransferase	ENSRNOG00000009974	29309
18654	8584	10175	720	690	-1,187	-0,247	0,012352707	NM_001013123.1	CUST_14123_PI421866198	NM_001013123	Zdhhc4	zinc finger, DHHC-type containing 4	ENSRNOG00000051699	304291
11241	20	23	2	2	-1,187	-0,247	0,016438623	XR_005735	XR_005735_P1	XR_005735	NA	lncRNA	NA	NA
28360	9958	11888	505	1624	-1,187	-0,247	0,023905145	NM_012792.1	CUST_9447_PI421866198	NM_012792	Fmo1	flavin containing monooxygenase 1	ENSRNOG00000034191	25256
12068	3095	3659	381	245	-1,187	-0,248	0,029243582	NM_001025650.1	CUST_10496_PI421866198	NM_001025650	Dusp11	dual specificity phosphatase 11	ENSRNOG00000022082	103690006
14882	6032	7093	953	262	-1,187	-0,248	0,045073379	NM_001024989.1	CUST_12091_PI421866198	NM_001024989	Trex1	three prime repair exonuclease 1	ENSRNOG00000022540	100049583
14681	8099	9646	752	1215	-1,188	-0,248	0,036882796	NM_031824.1	CUST_9890_PI421866198	NM_031824	Ctcf	CCCTC-binding factor	ENSRNOG00000017674	83726
23348	16729	19979	1367	2733	-1,188	-0,249	0,038367415	NM_001024908.1	CUST_12333_PI421866198	NM_001024908	Mfsd3	major facilitator superfamily domain containing 3	ENSRNOG00000015685	500899
23841	2322	2758	143	94	-1,189	-0,25	0,003479821	NM_001098727.1	CUST_15111_PI421866198	NM_001098727	Rprd1b	regulation of nuclear pre-mRNA domain containing 1B	ENSRNOG00000012923	311591
27861	9840	11728	782	1296	-1,189	-0,25	0,022384869	NM_001012014.1	CUST_2641_PI421866198	NM_001012014	Ftsj3	FtsJ homolog 3	ENSRNOG00000009857	303608
25034	7828	9252	1046	472	-1,189	-0,25	0,026355585	NM_001108377.2	CUST_6037_PI421866198	NM_001108377	Pck2	phosphoenolpyruvate carboxykinase 2 (mitochondrial)	ENSRNOG00000018536	NA
6384	18	21	2	3	-1,189	-0,249	0,037771305	BC091253	BC091253_P1	BC091253	NA	lncRNA	NA	NA
20723	25	29	4	2	-1,189	-0,25	0,048881033	MRAK083598	MRAK083598_P1	MRAK083598	NA	lncRNA	NA	NA
16909	39	47	3	6	-1,19	-0,251	0,031954796	BC091252	BC091252_P1	BC091252	NA	lncRNA	NA	NA
16995	43	51	3	6	-1,191	-0,252	0,017357554	MRAK050852	MRAK050852_P1	MRAK050852	NA	lncRNA	NA	NA
22991	25	30	2	4	-1,191	-0,252	0,026420507	MRAK164782	MRAK164782_P1	MRAK164782	NA	lncRNA	NA	NA
3641	1537	1827	182	177	-1,191	-0,253	0,032867719	NM_133577.1	CUST_6321_PI421866198	NM_133577	P2ry14	purinergic receptor P2Y14	ENSRNOG00000013872	171108
17073	4904	5834	545	601	-1,191	-0,252	0,034083964	NM_001127293.1	CUST_7347_PI421866198	NM_001127293	Zc3hc1	zinc finger, C3HC-type containing 1	ENSRNOG00000010154	296957
35821	137	163	16	21	-1,191	-0,252	0,045156001	NM_001134508.1	CUST_6751_PI421866198	NM_001134508	RGD1307603	similar to hypothetical protein MGC37914	ENSRNOG00000018287	293656
20740	77132	92413	7021	14193	-1,191	-0,252	0,045369057	NM_001142949.1	CUST_1503_PI421866198	NM_001142949	Nars	asparaginyl-tRNA synthetase	ENSRNOG00000017852	291556
13651	2957	3528	179	283	-1,192	-0,253	0,007939242	NM_001105842.1	CUST_12517_PI421866198	NM_001105842	Rdm1	RAD52 motif containing 1	ENSRNOG00000020751	287726
18507	53	64	5	7	-1,192	-0,254	0,025162202	MRAK042170	MRAK042170_P1	MRAK042170	NA	lncRNA	NA	NA
11552	1679	1997	192	171	-1,192	-0,253	0,026657849	NM_001007622.1	CUST_7171_PI421866198	NM_001007622	Pdlim2	PDZ and LIM domain 2	ENSRNOG00000008543	290354
23263	13159	15652	1704	1692	-1,192	-0,253	0,041628473	NM_001100698.1	CUST_14829_PI421866198	NM_001100698	Zfp131	zinc finger protein 131	ENSRNOG00000015925	310375
4052	26	31	1	2	-1,193	-0,255	0,006933735	EF088428	EF088428_P1	EF088428	NA	lncRNA	NA	NA
19718	43	51	4	4	-1,193	-0,255	0,014502669	NM_001001065.1	CUST_14771_PI421866198	NM_001001065	Olr707	olfactory receptor 707	ENSRNOG00000059153	NA
22865	82530	98433	7627	8564	-1,193	-0,255	0,018059714	NM_001017374.2	CUST_15112_PI421866198	NM_001017374	Eif4g2	eukaryotic translation initiation factor 4, gamma 2	ENSRNOG00000017158	361628
27334	4260	5093	350	553	-1,193	-0,255	0,018276994	NM_001107343.1	CUST_8682_PI421866198	NM_001107343	Kdm1b	lysine demethylase 1B	ENSRNOG00000016519	306819
45111	1739	2072	207	246	-1,193	-0,254	0,047584313	NM_001025749.1	CUST_9729_PI421866198	NM_001025749	Grap	GRB2-related adaptor protein	ENSRNOG00000002599	363616
24433	1143	1368	102	155	-1,194	-0,256	0,023451993	NM_001109054.1	CUST_4556_PI421866198	NM_001109054	Tctex1d2	Tctex1 domain containing 2	ENSRNOG00000001758	498095
25312	8644	10393	470	1494	-1,194	-0,256	0,024955092	NM_031695.2	CUST_11187_PI421866198	NM_031695	St3gal2	ST3 beta-galactoside alpha-2,3-sialyltransferase 2	ENSRNOG00000017932	64442
27452	6567	7864	718	1106	-1,194	-0,256	0,047892035	NM_001108064.1	CUST_7645_PI421866198	NM_001108064	Wiz	widely-interspaced zinc finger motifs	ENSRNOG00000006217	314598
25377	7836	9376	272	576	-1,195	-0,257	0,00272219	NM_001013866.1	CUST_9173_PI421866198	NM_001013866	Cmss1	cms1 ribosomal small subunit homolog (yeast)	ENSRNOG00000027888	288176
16082	30	36	2	4	-1,195	-0,257	0,021824909	BC100145	BC100145_P1	BC100145	NA	lncRNA	NA	NA
22585	5279	6323	446	733	-1,195	-0,257	0,023118616	NM_001014001.1	CUST_11071_PI421866198	NM_001014001	Cfap97	cilia and flagella associated protein 97	ENSRNOG00000032192	306469
19620	4405	5241	600	477	-1,195	-0,257	0,03646684	MRAK135279	MRAK135279_P1	MRAK135279	NA	lncRNA	NA	NA
26251	3905	4671	408	572	-1,195	-0,256	0,03732436	NM_001033063.1	CUST_11816_PI421866198	NM_001033063	Rpp38	ribonuclease P/MRP 38 subunit	ENSRNOG00000038025	291317
18017	10723	12789	1342	1378	-1,195	-0,257	0,038950832	NM_001105906.1	CUST_11793_PI421866198	NM_001105906	Uspl1	ubiquitin specific peptidase like 1	ENSRNOG00000000909	288447
12728	31	37	3	3	-1,196	-0,258	0,012343292	NM_001107888.2	CUST_6253_PI421866198	NM_001107888	Emg1	EMG1 N1-specific pseudouridine methyltransferase	ENSRNOG00000012828	312706
21900	6150	7358	609	752	-1,196	-0,258	0,023607443	NM_001024302.1	CUST_656_PI421866198	NM_001024302	Lysmd1	LysM domain containing 1	ENSRNOG00000021099	499671
19656	2869	3411	404	201	-1,196	-0,258	0,025494128	NM_001013951.1	CUST_8748_PI421866198	NM_001013951	Alg13	ALG13, UDP-N-acetylglucosaminyltransferase subunit	ENSRNOG00000005753	300284
19949	5777	6875	756	457	-1,196	-0,259	0,026050904	NM_201424.2	CUST_9188_PI421866198	NM_201424	Ugt1a3	NA	NA	NA
11785	2577	3088	254	384	-1,196	-0,258	0,032703545	MRAK034224	MRAK034224_P1	MRAK034224	NA	lncRNA	NA	NA
16971	3761	4477	508	426	-1,196	-0,258	0,049487004	NM_212507.2	CUST_12232_PI421866198	NM_212507	Ltb	lymphotoxin beta	ENSRNOG00000000836	103694381
18150	5557	6617	786	594	-1,197	-0,26	0,045880852	NM_001106378.1	CUST_12102_PI421866198	NM_001106378	Agpat3	1-acylglycerol-3-phosphate O-acyltransferase 3	ENSRNOG00000001205	294324
20533	4967	5957	421	601	-1,198	-0,261	0,015064974	NM_058209.2	CUST_4357_PI421866198	NM_058209	Zfp37	zinc finger protein 37	ENSRNOG00000051078	NA
21037	3905	4682	395	530	-1,198	-0,26	0,031132504	NM_001037776.1	CUST_9563_PI421866198	NM_001037776	Tmem177	transmembrane protein 177	ENSRNOG00000025484	304735
24296	185	221	24	22	-1,198	-0,261	0,032288709	NM_057200.1	CUST_7550_PI421866198	NM_057200	Kif1b	kinesin family member 1B	ENSRNOG00000057626	117548
24777	18	22	2	3	-1,198	-0,26	0,035217351	MRAK087717	MRAK087717_P1	MRAK087717	NA	lncRNA	NA	NA
20651	2923	3489	371	260	-1,199	-0,262	0,024294007	NM_001006994.1	CUST_8027_PI421866198	NM_001006994	Gpr180	G protein-coupled receptor 180	ENSRNOG00000009766	306165
24367	31372	37789	2247	4944	-1,199	-0,261	0,024655381	NM_001013151.1	CUST_14061_PI421866198	NM_001013151	Gna14	G protein subunit alpha 14	ENSRNOG00000014840	309242
22549	2955	3541	361	432	-1,199	-0,262	0,045184683	NM_001107674.1	CUST_7389_PI421866198	NM_001107674	Naa15	N(alpha)-acetyltransferase 15, NatA auxiliary subunit	ENSRNOG00000012385	310399
16872	649	782	69	113	-1,199	-0,262	0,046532463	NM_053385.1	CUST_4568_PI421866198	NM_053385	Prelp	proline and arginine rich end leucine rich repeat protein	ENSRNOG00000003120	84400
22226	1975	2374	213	298	-1,2	-0,263	0,032523518	NM_001024793.1	CUST_13443_PI421866198	NM_001024793	Cdc27	cell division cycle 27	ENSRNOG00000005904	360643
15553	10336	12353	1176	464	-1,201	-0,265	0,014671451	BC088294	BC088294_P1	BC088294	NA	lncRNA	NA	NA
15128	1352	1620	141	114	-1,201	-0,264	0,016157134	NM_001014023.1	CUST_11028_PI421866198	NM_001014023	Trim5	NA	NA	NA
23269	5824	7016	575	906	-1,201	-0,265	0,029815652	NM_001109293.1	CUST_3115_PI421866198	NM_001109293	RGD1563941	similar to hypothetical protein FLJ20010	ENSRNOG00000005918	500993
22579	815	977	93	82	-1,202	-0,265	0,019442021	NM_001079892.1	CUST_13485_PI421866198	NM_001079892	Apex2	apurinic/apyrimidinic endodeoxyribonuclease 2	ENSRNOG00000000166	317628
29093	1277	1527	160	120	-1,202	-0,265	0,028501119	NM_001109531.1	CUST_1358_PI421866198	NM_001109531	Mis18bp1	MIS18 binding protein 1	ENSRNOG00000023093	689296
26909	2093	2520	214	306	-1,202	-0,266	0,028832537	NM_001126299.1	CUST_9848_PI421866198	NM_001126299	Esco1	establishment of sister chromatid cohesion N-acetyltransferase 1	ENSRNOG00000014031	680014
18506	2372	2846	288	316	-1,202	-0,265	0,039504965	NM_001160263.1	CUST_9242_PI421866198	NM_001160263	Abo	ABO, alpha 1-3-N-acetylgalactosaminyltransferase and alpha 1-3-galactosyltransferase	ENSRNOG00000046958	100301568
1262	37	44	3	4	-1,203	-0,267	0,018202235	NM_001000002.1	CUST_2796_PI421866198	NM_001000002	Olr1398	olfactory receptor 1398	ENSRNOG00000045940	287245
9068	11	14	1	1	-1,203	-0,266	0,02441428	AJ286282	AJ286282_P1	AJ286282	NA	lncRNA	NA	NA
36823	86	103	9	14	-1,203	-0,267	0,037851042	MRAK037153	MRAK037153_P1	MRAK037153	NA	lncRNA	NA	NA
28213	551	660	76	69	-1,203	-0,267	0,03897312	NM_001013992.1	CUST_8670_PI421866198	NM_001013992	Zfp385d	zinc finger protein 385D	ENSRNOG00000014661	305691
21096	576	695	65	87	-1,204	-0,268	0,031667271	NM_001109478.1	CUST_1820_PI421866198	NM_001109478	Nme4	NA	NA	NA
18844	243	294	26	43	-1,204	-0,268	0,038135239	NM_001108556.1	CUST_4888_PI421866198	NM_001108556	Tsacc	TSSK6 activating co-chaperone	ENSRNOG00000037552	361979
2582	37	44	5	6	-1,204	-0,268	0,046598971	MRAK153687	MRAK153687_P1	MRAK153687	NA	lncRNA	NA	NA
18471	8735	10465	1289	854	-1,205	-0,27	0,03476174	NM_001024754.1	CUST_13601_PI421866198	NM_001024754	Gmps	guanine monophosphate synthase	ENSRNOG00000051151	295088
39485	121	146	13	11	-1,206	-0,27	0,013105043	NM_012628.1	CUST_10385_PI421866198	NM_012628	Prkcg	protein kinase C, gamma	ENSRNOG00000054371	24681
17844	786	946	96	92	-1,206	-0,271	0,024577852	NM_001109585.1	CUST_1174_PI421866198	NM_001109585	Trim47	tripartite motif-containing 47	ENSRNOG00000008215	690374
25893	6246	7520	817	878	-1,206	-0,27	0,033757334	NM_001107262.1	CUST_9085_PI421866198	NM_001107262	Sall2	spalt-like transcription factor 2	ENSRNOG00000013287	305854
9721	60	72	8	9	-1,206	-0,27	0,03856765	XR_008277	XR_008277_P1	XR_008277	NA	lncRNA	NA	NA
17247	150	181	17	26	-1,206	-0,271	0,042254552	MRAK147872	MRAK147872_P1	MRAK147872	NA	lncRNA	NA	NA
15067	210	253	25	37	-1,206	-0,27	0,047759329	NM_212518.1	CUST_12210_PI421866198	NM_212518	Abcb7	ATP binding cassette subfamily B member 7	ENSRNOG00000002790	NA
18873	2477	2962	406	82	-1,208	-0,273	0,030849762	NM_001163492.1	CUST_14219_PI421866198	NM_001163492	Zswim7	zinc finger, SWIM-type containing 7	ENSRNOG00000002970	287388
23004	51	62	5	10	-1,208	-0,273	0,038469437	NM_214456.1	CUST_6707_PI421866198	NM_214456	Olr1714	olfactory receptor 1714	ENSRNOG00000061444	NA
5276	28	34	3	6	-1,208	-0,272	0,042834333	MRAK037591	MRAK037591_P1	MRAK037591	NA	lncRNA	NA	NA

27362	1511	1821	224	230	-1,208	-0,272	0,04308056	NM_019123.1	CUST_6727_PI421866198	NM_019123	St6galnac3	ST6 N-acetylgalactosaminide alpha-2,6-sialyltransferase 3	ENSRNOG00000056894	29758
25395	1756	2142	148	374	-1,208	-0,273	0,045479726	NM_001107800.1	CUST_6450_PI421866198	NM_001107800	Stk4	serine/threonine kinase 4	ENSRNOG00000013529	311622
10879	18	22	3	3	-1,208	-0,272	0,049186216	MRAK084293	MRAK084293_P1	MRAK084293	NA	lncRNA	NA	NA
3341	17	21	1	2	-1,209	-0,274	0,007826847	MRAK029846	MRAK029846_P1	MRAK029846	NA	lncRNA	NA	NA
22085	2854	3453	231	300	-1,209	-0,274	0,009131428	NM_001009640.1	CUST_391_PI421866198	NM_001009640	Utp4	UTP4 small subunit processome component	ENSRNOG00000020333	291987
19329	2347	2817	343	124	-1,209	-0,274	0,021331008	NM_001017503.1	CUST_14080_PI421866198	NM_001017503	Purb	purine rich element binding protein B	ENSRNOG00000056150	498407
637	1090	1314	148	142	-1,209	-0,274	0,039045949	NM_001108119.1	CUST_7321_PI421866198	NM_001108119	Nckap1l	NCK associated protein 1 like	ENSRNOG00000036829	315348
4192	27	32	5	1	-1,209	-0,274	0,040761631	MRFT38186	MRFT38186_P1	MRFT38186	NA	lncRNA	NA	NA
8330	16	20	1	3	-1,21	-0,275	0,027993539	MRAK133424	MRAK133424_P1	MRAK133424	NA	lncRNA	NA	NA
17308	18	22	2	3	-1,21	-0,275	0,029622386	AB094071	AB094071_P1	AB094071	NA	lncRNA	NA	NA
23685	1411	1713	87	179	-1,211	-0,276	0,00793567	NM_001135016.1	CUST_6371_PI421866198	NM_001135016	Tmem86a	transmembrane protein 86A	ENSRNOG00000013663	308602
2249	103	125	7	13	-1,211	-0,277	0,010088011	MRAK137174	MRAK137174_P1	MRAK137174	NA	lncRNA	NA	NA
17528	1840	2208	275	128	-1,211	-0,276	0,032924648	NM_021583.2	CUST_5904_PI421866198	NM_021583	Ptges	prostaglandin E synthase	ENSRNOG00000006320	59103
34079	30	36	5	3	-1,211	-0,277	0,03563479	NM_001166678.1	CUST_3179_PI421866198	NM_001166678	Tas2r136	taste receptor, type 2, member 136	ENSRNOG00000029561	100310876
21242	2571	3118	314	417	-1,212	-0,277	0,042110063	NM_001109207.2	CUST_3309_PI421866198	NM_001109207	Gins1	GINS complex subunit 1	ENSRNOG00000008091	499914
21375	4703	5683	737	726	-1,212	-0,278	0,049514065	NM_001107506.1	CUST_7879_PI421866198	NM_001107506	Tshz3	teashirt zinc finger homeobox 3	ENSRNOG00000013938	308523
12207	16	19	1	3	-1,213	-0,279	0,02102132	MRAK020998	MRAK020998_P1	MRAK020998	NA	lncRNA	NA	NA
21591	14280	17144	2482	732	-1,213	-0,278	0,028172153	NM_001014226.2	CUST_9525_PI421866198	NM_001014226	Naa60	N(alpha)-acetyltransferase 60, NatF catalytic subunit	ENSRNOG00000007280	363545
19719	9928	11963	1518	1096	-1,213	-0,278	0,035483584	BC127549	BC127549_P1	BC127549	NA	lncRNA	NA	NA
21331	22062	26858	2527	3899	-1,213	-0,279	0,037249963	NM_199081.1	CUST_12774_PI421866198	NM_199081	Slc35b1	solute carrier family 35, member B1	ENSRNOG00000004510	NA
1143	79	96	9	15	-1,213	-0,278	0,038690882	MRAK006253	MRAK006253_P1	MRAK006253	NA	lncRNA	NA	NA
12140	38	47	4	8	-1,213	-0,279	0,042177832	NM_001107791.1	CUST_6876_PI421866198	NM_001107791	Bpifb6	BPI fold containing family B, member 6	ENSRNOG00000013371	311558
21380	51	62	4	7	-1,214	-0,279	0,013349136	NM_017186.1	CUST_12610_PI421866198	NM_017186	Gcm1	glial cells missing homolog 1	ENSRNOG00000007932	29394
15980	3816	4628	377	423	-1,214	-0,279	0,014219751	NM_019147.1	CUST_6649_PI421866198	NM_019147	Jag1	jagged 1	ENSRNOG00000007443	29146
28245	2	3	0	0	-1,214	-0,28	0,01957685	NM_001100981.1	CUST_12749_PI421866198	NM_001100981	NA	NA	NA	NA
21070	54	66	6	8	-1,214	-0,28	0,026160451	MRuc008ich	MRuc008ich_P1	MRuc008ich	NA	lncRNA	NA	NA
25894	1125	1377	78	204	-1,214	-0,279	0,028404054	NM_001077677.1	CUST_6160_PI421866198	NM_001077677	Pacrg	NA	NA	NA
21650	120	146	13	21	-1,214	-0,28	0,033540489	BC097997	BC097997_P1	BC097997	NA	lncRNA	NA	NA
10829	9	11	1	2	-1,214	-0,28	0,044968994	L11031	L11031_P1	L11031	NA	lncRNA	NA	NA
19947	37835	45991	2472	3452	-1,215	-0,281	0,00416919	NM_019209.1	CUST_6093_PI421866198	NM_019209	Urod	uroporphyrinogen decarboxylase	ENSRNOG00000018211	29421
29017	3146	3806	376	267	-1,215	-0,281	0,015948786	NM_001106164.1	CUST_13548_PI421866198	NM_001106164	Cmtm3	CKLF-like MARVEL transmembrane domain containing 3	ENSRNOG00000010691	291813
14519	31	37	5	3	-1,215	-0,281	0,03485809	MRuc007zhj	MRuc007zhj_P1	MRuc007zhj	NA	lncRNA	NA	NA
10998	56	68	7	11	-1,215	-0,281	0,047797451	MRAK028642	MRAK028642_P1	MRAK028642	NA	lncRNA	NA	NA
36458	31	38	3	5	-1,216	-0,282	0,021466691	NM_053447.1	CUST_3974_PI421866198	NM_053447	Hdac2	histone deacetylase 2	ENSRNOG00000000604	84577
16747	1230	1491	165	152	-1,216	-0,282	0,029421209	NM_001108022.1	CUST_7696_PI421866198	NM_001108022	RGD1304624	similar to RIKEN cDNA 2700097O09	ENSRNOG00000006986	314128
29448	2266	2739	332	259	-1,216	-0,282	0,036690445	NM_134369.1	CUST_4244_PI421866198	NM_134369	Cyp2t1	cytochrome P450, family 2, subfamily t, polypeptide 1	ENSRNOG00000028891	171380
103	2	3	0	0	-1,216	-0,282	0,038018641	AY310151	AY310151_P1	AY310151	NA	NA	NA	NA
3130	27	33	3	5	-1,216	-0,282	0,041594881	XR_007091	XR_007091_P1	XR_007091	NA	lncRNA	NA	NA
17223	3186	3861	399	252	-1,218	-0,285	0,016340521	NM_001024868.1	CUST_12849_PI421866198	NM_001024868	Pih1d1	PIH1 domain containing 1	ENSRNOG00000020634	292898
7325	1651	1995	263	160	-1,218	-0,284	0,037277862	NM_001107650.1	CUST_7437_PI421866198	NM_001107650	Ercc8	ERCC excision repair 8, CSA ubiquitin ligase complex subunit	ENSRNOG00000009968	310071
6910	1982	2387	372	162	-1,218	-0,284	0,040044947	NM_001039006.1	CUST_7933_PI421866198	NM_001039006	Klhl25	kelch-like family member 25	ENSRNOG00000010959	293023
27011	192	234	28	33	-1,218	-0,284	0,044255971	NM_013051.1	CUST_10783_PI421866198	NM_013051	Scgb1a1	secretoglobin family 1A member 1	ENSRNOG00000020196	25575
28844	462	558	72	41	-1,219	-0,285	0,039287229	NM_001006958.1	CUST_8085_PI421866198	NM_001006958	Sdad1	SDA1 domain containing 1	ENSRNOG00000022229	289504
12064	51	63	5	7	-1,22	-0,287	0,013024535	NM_001047881.1	CUST_606_PI421866198	NM_001047881	Arsi	arylsulfatase family, member I	ENSRNOG00000026060	307404
21583	2145	2591	395	215	-1,22	-0,287	0,043212824	NM_001105774.1	CUST_13092_PI421866198	NM_001105774	Rpusd1	RNA pseudouridylate synthase domain containing 1	ENSRNOG00000023639	287148
23330	110	134	17	21	-1,22	-0,286	0,047950952	uc.83+	uc.83+_P1	uc+	NA	lncRNA	NA	NA
31283	566	686	87	61	-1,221	-0,288	0,037970895	NM_001012085.1	CUST_2351_PI421866198	NM_001012085	Stam2	signal transducing adaptor molecule 2	ENSRNOG00000027447	311030
18548	2	3	0	0	-1,221	-0,288	0,038559974	MRAK164412	MRAK164412_P1	MRAK164412	NA	NA	NA	NA
26376	2811	3427	429	504	-1,221	-0,288	0,048336576	NM_033359.2	CUST_7159_PI421866198	NM_033359	Ngb	neuroglobin	ENSRNOG00000011719	NA
22828	2551	3119	292	405	-1,222	-0,289	0,026939673	MRAK088511	MRAK088511_P1	MRAK088511	NA	lncRNA	NA	NA
5847	807	976	130	37	-1,222	-0,29	0,029759034	MRAK148546	MRAK148546_P1	MRAK148546	NA	lncRNA	NA	NA
3697	2166	2635	299	260	-1,222	-0,29	0,031881375	MRAK162901	MRAK162901_P1	MRAK162901	NA	lncRNA	NA	NA
17992	1090	1337	140	222	-1,222	-0,29	0,047237256	NM_001008557.1	CUST_4349_PI421866198	NM_001008557	Zfp667	zinc finger protein 667	ENSRNOG00000033906	308326
12155	4332	5279	508	387	-1,223	-0,291	0,011977581	NM_001105737.1	CUST_13160_PI421866198	NM_001105737	Tek	TEK receptor tyrosine kinase	ENSRNOG00000008587	89804
19363	141037	171080	23667	16393	-1,223	-0,29	0,040519995	NM_022500.3	CUST_2715_PI421866198	NM_022500	Ftl1	ferritin light chain 1	ENSRNOG00000031506	100360087
28007	1412	1736	176	284	-1,223	-0,291	0,044008161	NM_001024979.1	CUST_12126_PI421866198	NM_001024979	Gpatch4	G patch domain containing 4	ENSRNOG00000018969	295228
15816	38	47	6	3	-1,224	-0,292	0,018556333	MRAK018088	MRAK018088_P1	MRAK018088	NA	lncRNA	NA	NA
16210	1442	1777	119	270	-1,224	-0,292	0,021742823	NM_001012061.1	CUST_2407_PI421866198	NM_001012061	Cnksr3	Cnksr family member 3	ENSRNOG00000018052	308113
23309	29	36	2	7	-1,224	-0,291	0,024575136	MRAK048830	MRAK048830_P1	MRAK048830	NA	lncRNA	NA	NA
17243	285	351	32	61	-1,224	-0,292	0,038919955	NM_001100975.2	CUST_12785_PI421866198	NM_001100975	Vps13a	vacuolar protein sorting 13A	ENSRNOG00000025539	NA
6242	38	47	5	8	-1,224	-0,291	0,04835681	MRuc008ymy	MRuc008ymy_P1	MRuc008ymy	NA	lncRNA	NA	NA
22679	7987	9839	682	1424	-1,225	-0,293	0,020581878	NM_053416.2	CUST_4027_PI421866198	NM_053416	Strbp	spermatid perinuclear RNA binding protein	ENSRNOG00000010150	84476
4676	637	771	100	13	-1,225	-0,293	0,03024031	AF204873	AF204873_P1	AF204873	NA	lncRNA	NA	NA
21333	141	171	24	13	-1,225	-0,292	0,032762969	XR_009487	XR_009487_P1	XR_009487	NA	lncRNA	NA	NA
21907	97	119	11	21	-1,225	-0,293	0,039573292	NM_019173.1	CUST_6565_PI421866198	NM_019173	Galr3	galanin receptor 3	ENSRNOG00000027060	29235
23185	4509	5530	327	458	-1,226	-0,294	0,004789383	NM_001109581.2	CUST_1179_PI421866198	NM_001109581	RGD1597339	uncharacterized protein LOC690349	ENSRNOG00000019701	690349
25235	18	22	3	3	-1,226	-0,294	0,034827934	MRAK052766	MRAK052766_P1	MRAK052766	NA	lncRNA	NA	NA
19875	2	3	0	0	-1,226	-0,293	0,046470699	XR_007120	XR_007120_P1	XR_007120	NA	NA	NA	NA
22099	71	86	10	6	-1,227	-0,296	0,015713833	NM_001000397.1	CUST_550_PI421866198	NM_001000397	Olr434	olfactory receptor 434	ENSRNOG00000008003	296694
18422	2014	2467	233	247	-1,227	-0,295	0,018085493	NM_001107258.1	CUST_9099_PI421866198	NM_001107258	Atg14	autophagy related 14	ENSRNOG00000011873	305831
9472	17	21	2	2	-1,227	-0,295	0,025311596	XR_006061	XR_006061_P1	XR_006061	NA	lncRNA	NA	NA
23002	35	44	3	8	-1,227	-0,295	0,032628632	NM_001014064.1	CUST_10941_PI421866198	NM_001014064	Pars2	prolyl-tRNA synthetase 2, mitochondrial (putative)	ENSRNOG00000007327	313429
42894	79	98	9	19	-1,227	-0,296	0,045335496	NM_175590.2	CUST_9359_PI421866198	NM_175590	Taar7e	trace-amine-associated receptor 7e	ENSRNOG00000046903	294133
22499	22	28	2	6	-1,227	-0,295	0,047867187	NM_001109237.1	CUST_3244_PI421866198	NM_001109237	Neurod6	neuronal differentiation 6	ENSRNOG00000026055	500137
37934	18	22	3	2	-1,229	-0,298	0,021945448	NM_001001506.1	CUST_11425_PI421866198	NM_001001506	Mrgprd	MAS related GPR family member D	ENSRNOG00000013448	293648
3709	6818	8316	1108	680	-1,229	-0,298	0,027251038	MRAK164927	MRAK164927_P1	MRAK164927	NA	lncRNA	NA	NA
29532	2348	2873	359	299	-1,229	-0,298	0,030975786	NM_001109286.1	CUST_3124_PI421866198	NM_001109286	Bbs10	Bardet-Biedl syndrome 10	ENSRNOG00000026913	500832
37173	478	589	60	92	-1,229	-0,297	0,033177823	NM_012545.3	CUST_11057_PI421866198	NM_012545	Ddc	dopa decarboxylase	ENSRNOG00000004327	24311
15703	5301	6523	471	623	-1,23	-0,298	0,007312455	NM_053857.1	CUST_1393_PI421866198	NM_053857	Eif4ebp1	eukaryotic translation initiation factor 4E binding protein 1	ENSRNOG00000012582	116636
25527	386	475	35	54	-1,23	-0,299	0,010212484	NM_001108131.1	CUST_7303_PI421866198	NM_001108131	Snx19	sorting nexin 19	ENSRNOG00000014719	315478
35019	50	61	7	8	-1,23	-0,299	0,033039444	NM_001000709.1	CUST_13902_PI421866198	NM_001000709	Olr1084	olfactory receptor 1084	ENSRNOG00000051708	404958
11557	27	34	4	5	-1,23	-0,299	0,039574855	MRAK046308	MRAK046308_P1	MRAK046308	NA	lncRNA	NA	NA
11636	24	30	4	4	-1,231	-0,3	0,035077635	NM_001108313.1	CUST_6167_PI421866198	NM_001108313	Ifngr2	interferon gamma receptor 2	ENSRNOG00000002032	360697
24960	31	38	3	7	-1,231	-0,299	0,036012939	NM_001109670.1	CUST_692_PI421866198	NM_001109670	Slc52a2	solute carrier family 52 member 2	ENSRNOG00000032561	362942
661	6	7	1	1	-1,231	-0,3	0,043123379	MRAK088349	MRAK088349_P1	MRAK088349	NA	NA	NA	NA
25130	285	350	45	44	-1,231	-0,3	0,048982591	NM_031787.1	CUST_10534_PI421866198	NM_031787	Hipk3	homeodomain interacting protein kinase 3	ENSRNOG00000011358	83617
40329	44	55	3	11	-1,232	-0,301	0,032970787	NM_019622.1	CUST_3433_PI421866198	NM_019622	Espn	espin	ENSRNOG00000010270	56227
32372	1485	1818	261	223	-1,232	-0,301	0,039938829	NM_001134704.1	CUST_5412_PI421866198	NM_001134704	Ormdl1	ORMDL sphingolipid biosynthesis regulator 1	ENSRNOG00000042961	100188936
23039	468	574	73	71	-1,233	-0,302	0,044052944	MRAK142189	MRAK142189_P1	MRAK142189	NA	lncRNA	NA	NA
41442	33	41	4	8	-1,234	-0,303	0,04063678	NM_001135090.1	CUST_6234_PI421866198	NM_001135090	Adam34	NA	NA	NA
25549	1823	2261	237	392	-1,234	-0,304	0,046029906	NM_001013969.1	CUST_8715_PI421866198	NM_001013969	Slc46a1	solute carrier family 46 member 1	ENSRNOG00000010291	303333
22068	8531	10564	869	1419	-1,235	-0,304	0,018810374	NM_001024360.2	CUST_508_PI421866198	NM_001024360	Lrrc73	leucine rich repeat containing 73	ENSRNOG00000039856	501101
4745	2	3	0	1	-1,235	-0,305	0,032877741	XR_008085	XR_008085_P1	XR_008085	NA	NA	NA	NA
20627	4942	6095	602	636	-1,236	-0,305	0,015085646	NM_001130566.1	CUST_4113_PI421866198	NM_001130566	Ppp1r16a	protein phosphatase 1, regulatory subunit 16A	ENSRNOG00000015450	362944
13430	16	20	2	2	-1,236	-0,306	0,020047381	S83271	S83271_P1	S83271	NA	lncRNA	NA	NA
24446	770	954	103	146	-1,236	-0,306	0,035905884	NM_001109321.1	CUST_2779_PI421866198	NM_001109321	Mum1l1	MUM1 like 1	ENSRNOG00000030475	501630
22870	17	22	2	4	-1,236	-0,306	0,037077362	MRAK132953	MRAK132953_P1	MRAK132953	NA	lncRNA	NA	NA
9376	1357	1678	189	245	-1,236	-0,306	0,037902348	NM_022676.3	CUST_1675_PI421866198	NM_022676	Ppp1r1a	protein phosphatase 1, regulatory (inhibitor) subunit 1A	ENSRNOG00000036827	58977
14929	24	30	5	4	-1,236	-0,306	0,046926822	MRAK032599	MRAK032599_P1	MRAK032599	NA	lncRNA	NA	NA
25327	5086	6259	866	839	-1,236	-0,306	0,049495447	NM_001107691.1	CUST_7360_PI421866198	NM_001107691	Trim46	tripartite motif-containing 46	ENSRNOG00000055433	310641
24010	4866	5996	528	378	-1,237	-0,307	0,007632456	NM_001108175.1	CUST_7241_PI421866198	NM_001108175	Tbc1d2b	TBC1 domain family, member 2B	ENSRNOG00000014543	315880
24274	2	2	0	0	-1,237	-0,307	0,023522495	NM_031029.1	CUST_15243_PI421866198	NM_031029	Gabrp	gamma-aminobutyric acid type A receptor pi subunit	ENSRNOG00000032417	81658
298	152	190	18	32	-1,237	-0,307	0,036438891	BC167034	BC167034_P1	BC167034	NA	lncRNA	NA	NA
22386	1431	1758	254	197	-1,237	-0,307	0,037747909	NM_001109111.1	CUST_3971_PI421866198	NM_001109111	Wwc2	WW and C2 domain containing 2	ENSRNOG00000013248	498630
37252	170	210	26	30	-1,237	-0,307	0,037777107	NM_012719.1	CUST_9640_PI421866198	NM_012719	Sstr1	somatostatin receptor 1	ENSRNOG00000048145	25033
4306	2	3	0	1	-1,237	-0,307	0,038301719	AF036298	AF036298_P1	AF036298	NA	NA	NA	NA
26735	17048	21135	1832	2810	-1,238	-0,308	0,014678333	NM_031675.2	CUST_11214_PI421866198	NM_031675	Actn4	actinin alpha 4	ENSRNOG00000020433	63836
14323	395	487	62	66	-1,238	-0,308	0,043842132	BC085710	BC085710_P1	BC085710	NA	lncRNA	NA	NA
4396	26	33	4	6	-1,238	-0,308	0,045298464	XR_007678	XR_007678_P1	XR_007678	NA	lncRNA	NA	NA
26643	13	17	2	1	-1,239	-0,309	0,008460816	uc.304-	uc.304-_P1	uc-	NA	lncRNA	NA	NA
13915	39	48	7	4	-1,239	-0,31	0,022984112	EU348628	EU348628_P1	EU348628	NA	lncRNA	NA	NA
36069	89	108	20	6	-1,239	-0,309	0,046124013	NM_001127488.1	CUST_6228_PI421866198	NM_001127488	Snx33	sorting nexin 33	ENSRNOG00000017382	315696
4391	16	20	1	1	-1,24	-0,311	0,001476338	AM293840	AM293840_P1	AM293840	NA	lncRNA	NA	NA
23689	31	38	4	6	-1,24	-0,311	0,022311765	NM_053831.1	CUST_1462_PI421866198	NM_053831	Gucy2f	guanylate cyclase 2F	ENSRNOG00000019086	116556
17660	85	105	12	16	-1,24	-0,31	0,034167879	NM_138861.1	CUST_3422_PI421866198	NM_138861	Prl2b1	Prolactin family 2, subfamily b, member 1	ENSRNOG00000017007	192224
22581	17	21	2	3	-1,241	-0,312	0,015472727	NM_207606.1	CUST_4591_PI421866198	NM_207606	Kirrel1	NA	NA	NA
33785	14	18	1	2	-1,242	-0,312	0,006735177	uc.203-	uc.203-_P1	uc-	NA	lncRNA	NA	NA
30925	57	71	8	3	-1,242	-0,313	0,01053841	NM_031016.1	CUST_15255_PI421866198	NM_031016	Chrm2	cholinergic receptor, muscarinic 2	ENSRNOG00000046972	81645
9771	36	44	5	3	-1,242	-0,313	0,013829649	XR_007976	XR_007976_P1	XR_007976	NA	lncRNA	NA	NA
29887	119	147	17	19	-1,243	-0,314	0,02752614	NM_012765.3	CUST_9518_PI421866198	NM_012765	Htr2c	5-hydroxytryptamine receptor 2C	ENSRNOG00000030877	25187
12014	543	668	94	54	-1,243	-0,314	0,027574503	NM_053806.2	CUST_1494_PI421866198	NM_053806	Kcnk6	potassium two pore domain channel subfamily K member 6	ENSRNOG00000020598	100909725
19538	77	95	14	10	-1,243	-0,313	0,044822318	NM_001000906.1	CUST_12485_PI421866198	NM_001000906	Olr1104	olfactory receptor 1104	ENSRNOG00000031284	405216
15638	59	75	6	14	-1,244	-0,315	0,032635055	NM_001135715.1	CUST_1834_PI421866198	NM_001135715	Trim67	tripartite motif-containing 67	ENSRNOG00000019024	307938
14600	68	86	6	20	-1,244	-0,315	0,049060934	XR_006144	XR_006144_P1	XR_006144	NA	lncRNA	NA	NA
16642	49	61	4	8	-1,245	-0,317	0,014192549	NM_001109078.1	CUST_4500_PI421866198	NM_001109078	Slamf1	signaling lymphocytic activation molecule family member 1	ENSRNOG00000059073	498286
26302	233	291	27	45	-1,245	-0,316	0,027712653	NM_019142.1	CUST_6658_PI421866198	NM_019142	Prkaa1	protein kinase AMP-activated catalytic subunit alpha 1	ENSRNOG00000012799	65248
18852	7425	9276	1023	1545	-1,245	-0,316	0,036718672	NM_001109645.2	CUST_775_PI421866198	NM_001109645	Katnbl1	NA	NA	NA
21509	542	667	98	55	-1,245	-0,316	0,037705045	NM_001017456.1	CUST_14593_PI421866198	NM_001017456	Klhdc10	kelch domain containing 10	ENSRNOG00000010267	312199
1952	183	225	40	26	-1,245	-0,316	0,047180049	MRAK164565	MRAK164565_P1	MRAK164565	NA	lncRNA	NA	NA
28560	2046	2538	314	284	-1,246	-0,317	0,022607488	NM_133323.1	CUST_7499_PI421866198	NM_133323	Zfp111	zinc finger protein 111	ENSRNOG00000024376	170849
24476	30	37	5	4	-1,246	-0,317	0,025535692	NM_001000006.1	CUST_2792_PI421866198	NM_001000006	Olr1423	olfactory receptor 1423	ENSRNOG00000049845	287309
25660	26	32	5	2	-1,246	-0,317	0,034227371	NM_001000923.1	CUST_12450_PI421866198	NM_001000923	Olr714	olfactory receptor 714	ENSRNOG00000049728	405239
10225	12	15	1	3	-1,246	-0,317	0,037016503	AB073720	AB073720_P1	AB073720	NA	lncRNA	NA	NA
7512	2	3	0	0	-1,247	-0,318	0,022352963	MRAK051886	MRAK051886_P1	MRAK051886	NA	NA	NA	NA
10897	16	20	2	3	-1,247	-0,318	0,022832174	AM293802	AM293802_P1	AM293802	NA	lncRNA	NA	NA
17923	16	20	2	2	-1,248	-0,32	0,006497544	NM_001106220.1	CUST_12932_PI421866198	NM_001106220	Nlrp4a	NLR family, pyrin domain containing 4A	ENSRNOG00000042030	292545
26920	2604	3250	276	371	-1,248	-0,319	0,012506714	NM_001024256.1	CUST_1049_PI421866198	NM_001024256	Med4	mediator complex subunit 4	ENSRNOG00000017170	306030
12217	628	771	130	52	-1,248	-0,319	0,044730959	NM_001014039.1	CUST_10998_PI421866198	NM_001014039	Tnfaip8l2	TNF alpha induced protein 8 like 2	ENSRNOG00000021100	310663
22938	43	55	3	8	-1,249	-0,321	0,009109551	NM_001000454.1	CUST_176_PI421866198	NM_001000454	Olr1252	olfactory receptor 1252	ENSRNOG00000046241	100912507
12223	78	97	8	12	-1,249	-0,32	0,011730773	NM_001166681.1	CUST_3154_PI421866198	NM_001166681	Tas2r104	taste receptor, type 2, member 104	ENSRNOG00000032448	100310879
9279	9	11	1	2	-1,249	-0,321	0,018394609	MRAK035806	MRAK035806_P1	MRAK035806	NA	lncRNA	NA	NA
27759	38	47	6	6	-1,249	-0,321	0,032823936	NM_001079838.1	CUST_13670_PI421866198	NM_001079838	Slc15a1	solute carrier family 15 member 1	ENSRNOG00000011598	117261
35736	934	1150	175	54	-1,25	-0,322	0,034852567	NM_001105868.1	CUST_12475_PI421866198	NM_001105868	Ephb3	Eph receptor B3	ENSRNOG00000031801	287989
28630	13	17	2	3	-1,25	-0,321	0,039570531	NM_001134536.1	CUST_6665_PI421866198	NM_001134536	Tmem132b	transmembrane protein 132B	ENSRNOG00000038406	NA
22252	8134	10172	542	649	-1,251	-0,323	0,001319447	NM_001106673.1	CUST_9984_PI421866198	NM_001106673	Gpx7	glutathione peroxidase 7	ENSRNOG00000009751	298376
12142	48	60	5	8	-1,251	-0,323	0,012743884	NM_001008969.1	CUST_1554_PI421866198	NM_001008969	NA	NA	NA	NA
16911	16	20	2	3	-1,251	-0,324	0,036103484	MRAK039199	MRAK039199_P1	MRAK039199	NA	lncRNA	NA	NA
30608	13	16	1	1	-1,252	-0,324	0,006912052	XR_005697	XR_005697_P1	XR_005697	NA	lncRNA	NA	NA
33512	20	25	3	3	-1,252	-0,324	0,029645207	AY539906	AY539906_P1	AY539906	NA	lncRNA	NA	NA
17407	46	58	7	10	-1,252	-0,324	0,030897602	L03556	L03556_P1	L03556	NA	lncRNA	NA	NA
9902	48	60	6	10	-1,252	-0,325	0,036887219	BC092653	BC092653_P1	BC092653	NA	lncRNA	NA	NA
36248	15	18	3	2	-1,252	-0,325	0,039751955	NM_001108474.1	CUST_5376_PI421866198	NM_001108474	Igfl3	NA	NA	NA
18957	680	839	130	57	-1,252	-0,324	0,042731234	NM_017117.1	CUST_12827_PI421866198	NM_017117	Capn3	calpain 3	ENSRNOG00000008609	29155
20482	2	3	0	0	-1,253	-0,325	0,003587879	MRAK015134	MRAK015134_P1	MRAK015134	NA	NA	NA	NA
38488	32	41	4	8	-1,253	-0,325	0,035047059	MRAK089898	MRAK089898_P1	MRAK089898	NA	lncRNA	NA	NA
13263	11	13	2	2	-1,253	-0,326	0,036967237	MRAK152883	MRAK152883_P1	MRAK152883	NA	lncRNA	NA	NA
19360	63	81	5	20	-1,253	-0,325	0,04461691	MRAK143555	MRAK143555_P1	MRAK143555	NA	lncRNA	NA	NA
26402	4177	5205	642	396	-1,254	-0,327	0,012310562	NM_001108623.1	CUST_4284_PI421866198	NM_001108623	NA	NA	NA	NA
14531	30	37	3	5	-1,254	-0,326	0,013710853	NM_001106354.1	CUST_12184_PI421866198	NM_001106354	NA	NA	NA	NA
18360	1864	2327	298	304	-1,254	-0,326	0,031578437	NM_133310.2	CUST_7513_PI421866198	NM_133310	Icmt	isoprenylcysteine carboxyl methyltransferase	ENSRNOG00000010953	170818
21298	1143	1434	167	228	-1,254	-0,327	0,040273015	NM_024161.2	CUST_13807_PI421866198	NM_024161	Dnajc5	DnaJ heat shock protein family (Hsp40) member C5	ENSRNOG00000015202	79130
6437	15	19	3	2	-1,254	-0,327	0,042219184	AY539921	AY539921_P1	AY539921	NA	lncRNA	NA	NA
24525	4023	5051	499	635	-1,255	-0,328	0,013661735	NM_001126281.1	CUST_9875_PI421866198	NM_001126281	Zfp955a	zinc finger protein 955A	ENSRNOG00000048577	314600
20800	11	14	2	2	-1,255	-0,328	0,02092999	L11022	L11022_P1	L11022	NA	lncRNA	NA	NA
19745	115	146	14	26	-1,255	-0,327	0,032447601	BC161910	BC161910_P1	BC161910	NA	lncRNA	NA	NA
4063	35	44	3	2	-1,256	-0,329	0,002590424	MRuc008ktl	MRuc008ktl_P1	MRuc008ktl	NA	lncRNA	NA	NA
2776	20	25	2	2	-1,256	-0,329	0,008267024	MRAK037832	MRAK037832_P1	MRAK037832	NA	lncRNA	NA	NA
22762	19	24	2	4	-1,256	-0,329	0,030195148	NM_001106475.1	CUST_11379_PI421866198	NM_001106475	Adh6a	alcohol dehydrogenase 6A (class V)	ENSRNOG00000012281	295498
23245	5588	6958	994	749	-1,256	-0,329	0,034280619	NM_001025762.1	CUST_9685_PI421866198	NM_001025762	Smyd3	NA	NA	NA
21383	105	131	18	18	-1,256	-0,329	0,041598877	NM_001134739.1	CUST_5316_PI421866198	NM_001134739	NA	NA	NA	NA
25140	399	508	40	107	-1,256	-0,329	0,042454706	NM_001035251.1	CUST_4612_PI421866198	NM_001035251	Slc38a9	solute carrier family 38, member 9	ENSRNOG00000009851	310091
23173	20	25	2	3	-1,257	-0,33	0,006590925	AF112446	AF112446_P1	AF112446	NA	lncRNA	NA	NA
19861	3163	3987	363	581	-1,257	-0,33	0,01714821	NM_001013216.2	CUST_13356_PI421866198	NM_001013216	Zbtb48	zinc finger and BTB domain containing 48	ENSRNOG00000009595	362668
19084	14	17	2	2	-1,257	-0,33	0,018012953	MRAK050685	MRAK050685_P1	MRAK050685	NA	lncRNA	NA	NA
18762	1834	2289	312	296	-1,257	-0,33	0,038431491	NM_133380.2	CUST_7424_PI421866198	NM_133380	Il4r	interleukin 4 receptor	ENSRNOG00000015441	25084
13188	50	63	9	10	-1,257	-0,329	0,042405158	BC168184	BC168184_P1	BC168184	NA	lncRNA	NA	NA
22824	5298	6637	596	284	-1,258	-0,332	0,003651431	NM_001108679.2	CUST_4126_PI421866198	NM_001108679	Eva1b	eva-1 homolog B	ENSRNOG00000024818	362597
38627	103	130	10	13	-1,258	-0,331	0,005261337	NM_001142944.1	CUST_1505_PI421866198	NM_001142944	Trim43a	tripartite motif-containing 43A	ENSRNOG00000010699	300884
17252	62	78	8	7	-1,258	-0,331	0,007648758	MRAK080604	MRAK080604_P1	MRAK080604	NA	lncRNA	NA	NA
22631	8248	10363	1195	1452	-1,258	-0,331	0,021125756	NM_031147.2	CUST_14777_PI421866198	NM_031147	Cirbp	cold inducible RNA binding protein	ENSRNOG00000015999	81825
17676	59	74	8	12	-1,258	-0,331	0,027541512	uc.289-	uc.289-_P1	uc-	NA	lncRNA	NA	NA
26502	196	246	29	36	-1,258	-0,331	0,030366823	NM_001012359.1	CUST_580_PI421866198	NM_001012359	Ang2	angiogenin, ribonuclease A family, member 2	ENSRNOG00000025562	497229
30721	773	953	176	31	-1,258	-0,331	0,038570527	NM_017103.2	CUST_12864_PI421866198	NM_017103	Eloa	elongin A	ENSRNOG00000010902	25562
25902	21	26	3	5	-1,258	-0,332	0,039251898	NM_001017514.1	CUST_14037_PI421866198	NM_001017514	Izumo1	izumo sperm-egg fusion 1	ENSRNOG00000024678	499152
18465	482	613	69	133	-1,258	-0,331	0,046650988	EF673688	EF673688_P1	EF673688	NA	lncRNA	NA	NA
2631	888	1110	147	121	-1,259	-0,333	0,028605846	AF473845	AF473845_P1	AF473845	NA	lncRNA	NA	NA
27769	241	300	43	26	-1,259	-0,332	0,034020003	NM_001108095.1	CUST_7606_PI421866198	NM_001108095	NA	NA	NA	NA
40624	69	86	13	11	-1,259	-0,333	0,037293005	NM_001008957.1	CUST_1567_PI421866198	NM_001008957	NA	NA	NA	NA
19490	5215	6568	372	433	-1,26	-0,333	0,001232609	NM_199097.1	CUST_12707_PI421866198	NM_199097	Adi1	acireductone dioxygenase 1	ENSRNOG00000008950	298934
11623	12	15	1	2	-1,26	-0,334	0,007223262	XR_009509	XR_009509_P1	XR_009509	NA	lncRNA	NA	NA
2318	2	3	0	1	-1,26	-0,333	0,027445914	MRAK171098	MRAK171098_P1	MRAK171098	NA	NA	NA	NA
25302	6383	8112	798	1516	-1,26	-0,334	0,03544878	NM_001108615.1	CUST_4311_PI421866198	NM_001108615	Myt1	myelin transcription factor 1	ENSRNOG00000017346	362291
42953	177	221	31	27	-1,26	-0,333	0,035945442	NM_053970.2	CUST_792_PI421866198	NM_053970	Nln	neurolysin	ENSRNOG00000011561	117041
18940	120	151	21	21	-1,26	-0,333	0,040431723	MRAK142313	MRAK142313_P1	MRAK142313	NA	lncRNA	NA	NA
24710	1570	1980	250	317	-1,261	-0,334	0,041008836	NM_001044224.1	CUST_14342_PI421866198	NM_001044224	Spag5	sperm associated antigen 5	ENSRNOG00000011777	252918
24217	69	88	10	14	-1,262	-0,335	0,026115281	BC158633	BC158633_P1	BC158633	NA	lncRNA	NA	NA
14535	46	59	6	11	-1,262	-0,336	0,029209956	NM_001107686.1	CUST_7365_PI421866198	NM_001107686	Sprr3	small proline-rich protein 3	ENSRNOG00000024046	310575
5836	29	36	3	3	-1,263	-0,337	0,006318831	MRAK090160	MRAK090160_P1	MRAK090160	NA	lncRNA	NA	NA
19093	90	114	11	19	-1,263	-0,337	0,02155349	NM_001014152.1	CUST_10217_PI421866198	NM_001014152	Phkb	phosphorylase kinase regulatory subunit beta	ENSRNOG00000024101	361377
20431	112	142	17	24	-1,263	-0,337	0,036224091	NM_001037194.1	CUST_13873_PI421866198	NM_001037194	Det1	de-etiolated homolog 1 (Arabidopsis)	ENSRNOG00000018515	308775
11732	1417	1791	159	211	-1,264	-0,338	0,009432034	BC169051	BC169051_P1	BC169051	NA	lncRNA	NA	NA
12948	373	471	59	76	-1,264	-0,338	0,044050807	NM_001029918.1	CUST_11146_PI421866198	NM_001029918	Ttc30a1	tetratricopeptide repeat domain 30A1	ENSRNOG00000052519	311123
20591	418	523	80	68	-1,264	-0,338	0,048677942	MRAK012957	MRAK012957_P1	MRAK012957	NA	lncRNA	NA	NA
15622	9	11	1	2	-1,265	-0,339	0,023608267	MRAK075685	MRAK075685_P1	MRAK075685	NA	lncRNA	NA	NA
41551	119	150	22	22	-1,265	-0,34	0,036546764	NM_001139494.1	CUST_6147_PI421866198	NM_001139494	Lrtomt	leucine rich transmembrane and O-methyltransferase domain containing	ENSRNOG00000023434	308868
25790	2	3	0	1	-1,265	-0,34	0,046571796	NM_001000120.1	CUST_1990_PI421866198	NM_001000120	Olr30	olfactory receptor 30	ENSRNOG00000029233	293091
23115	25	31	5	4	-1,266	-0,341	0,035626148	uc.20-	uc.20-_P1	uc-	NA	lncRNA	NA	NA
1171	18	23	2	6	-1,266	-0,34	0,03748362	L21004	L21004_P1	L21004	NA	lncRNA	NA	NA
23943	654	815	146	80	-1,266	-0,34	0,042210368	NM_001024328.2	CUST_567_PI421866198	NM_001024328	Gimap7	GTPase, IMAP family member 7	ENSRNOG00000038740	500113
23169	944	1190	127	102	-1,267	-0,342	0,011541062	NM_001107217.1	CUST_9204_PI421866198	NM_001107217	Med28	mediator complex subunit 28	ENSRNOG00000003592	305391
25442	2	3	0	0	-1,268	-0,342	0,00804465	DQ294718	DQ294718_P1	DQ294718	NA	NA	NA	NA
3617	25	31	3	2	-1,268	-0,343	0,009471342	MRAK138791	MRAK138791_P1	MRAK138791	NA	lncRNA	NA	NA
6303	35	44	6	5	-1,268	-0,342	0,019859647	MRAK079288	MRAK079288_P1	MRAK079288	NA	lncRNA	NA	NA
19615	186	235	31	37	-1,268	-0,343	0,026939981	NM_001107816.1	CUST_6422_PI421866198	NM_001107816	Tor4a	torsin family 4, member A	ENSRNOG00000009369	311795
6363	12	15	2	3	-1,268	-0,343	0,047321284	MRAK163087	MRAK163087_P1	MRAK163087	NA	lncRNA	NA	NA
9113	21	27	4	3	-1,269	-0,344	0,02545207	MRAK033668	MRAK033668_P1	MRAK033668	NA	lncRNA	NA	NA
33901	143	179	31	16	-1,269	-0,343	0,029868203	NM_001017507.1	CUST_14072_PI421866198	NM_001017507	Mef2b	myocyte enhancer factor 2B	ENSRNOG00000020400	498607
21820	1289	1631	139	76	-1,27	-0,345	0,002412678	NM_001012133.1	CUST_1796_PI421866198	NM_001012133	Sp140	SP140 nuclear body protein	ENSRNOG00000022800	NA

11748	659	830	122	112	-1,27	-0,345	0,043221082	NM_001103361.1	CUST_7012_PI421866198	NM_001103361	Adat3	NA	NA	NA
1494	79	100	15	14	-1,27	-0,345	0,043853778	NM_138857.1	CUST_3440_PI421866198	NM_138857	Prom2	prominin 2	ENSRNOG00000014710	192211
9268	29	36	3	3	-1,271	-0,346	0,004643957	M63991	M63991_P1	M63991	NA	lncRNA	NA	NA
2293	2431	3084	335	353	-1,271	-0,346	0,012052233	MRAK164892	MRAK164892_P1	MRAK164892	NA	lncRNA	NA	NA
3940	2	3	0	1	-1,271	-0,346	0,015836867	MRAK043422	MRAK043422_P1	MRAK043422	NA	NA	NA	NA
20125	1809	2272	342	241	-1,271	-0,346	0,035829676	NM_001004242.1	CUST_3719_PI421866198	NM_001004242	Arhgap8	Rho GTPase activating protein 8	ENSRNOG00000033570	300115
5913	35	44	5	4	-1,272	-0,347	0,009204009	M77360	M77360_P1	M77360	NA	lncRNA	NA	NA
15065	42	52	10	4	-1,272	-0,347	0,03887894	NM_001108671.1	CUST_4134_PI421866198	NM_001108671	Ror1	receptor tyrosine kinase-like orphan receptor 1	ENSRNOG00000029449	362550
11196	17	21	3	4	-1,272	-0,347	0,049702214	XR_005773	XR_005773_P1	XR_005773	NA	lncRNA	NA	NA
27594	2377	3048	173	470	-1,273	-0,348	0,009301115	NM_053656.2	CUST_2782_PI421866198	NM_053656	P2rx2	purinergic receptor P2X 2	ENSRNOG00000037456	114115
37554	26	32	5	2	-1,273	-0,348	0,024136602	NM_001109015.1	CUST_4682_PI421866198	NM_001109015	Tceanc	transcription elongation factor A N-terminal and central domain containing	ENSRNOG00000004531	367782
28553	7024	8924	1118	1328	-1,273	-0,348	0,025242892	NM_001110137.1	CUST_8454_PI421866198	NM_001110137	Prom1	prominin 1	ENSRNOG00000003098	60357
17232	144	183	27	31	-1,273	-0,349	0,035931745	NM_001001427.1	CUST_12075_PI421866198	NM_001001427	Olr1746	olfactory receptor 1746	ENSRNOG00000050443	405199
10836	486	615	94	91	-1,273	-0,348	0,042542824	NM_001105766.1	CUST_13113_PI421866198	NM_001105766	Pkmyt1	protein kinase, membrane associated tyrosine/threonine 1	ENSRNOG00000003657	287101
16679	50	64	9	14	-1,273	-0,348	0,045040869	MRAK040552	MRAK040552_P1	MRAK040552	NA	lncRNA	NA	NA
21726	41	52	7	9	-1,274	-0,349	0,039212614	NM_001005382.1	CUST_15130_PI421866198	NM_001005382	Klk1c12	NA	NA	NA
6256	14	18	3	3	-1,274	-0,35	0,043178202	MRuc009akg	MRuc009akg_P1	MRuc009akg	NA	lncRNA	NA	NA
23754	10411	13306	829	1538	-1,275	-0,35	0,003323823	NM_001108870.1	CUST_2946_PI421866198	NM_001108870	Scara3	scavenger receptor class A, member 3	ENSRNOG00000016177	364405
3288	30	39	4	5	-1,275	-0,35	0,010898799	Z93360	Z93360_P1	Z93360	NA	lncRNA	NA	NA
26765	86	109	15	15	-1,275	-0,35	0,022187807	NM_175586.2	CUST_9378_PI421866198	NM_175586	Taar7b	trace amine-associated receptor 7b	ENSRNOG00000046302	294126
1063	1042	1311	203	127	-1,275	-0,35	0,031733183	MRAK033995	MRAK033995_P1	MRAK033995	NA	lncRNA	NA	NA
24008	326	417	48	72	-1,275	-0,351	0,033823213	NM_001108427.1	CUST_5500_PI421866198	NM_001108427	Pfdn1	prefoldin subunit 1	ENSRNOG00000018653	361310
26625	2	3	0	0	-1,276	-0,352	0,002703241	NM_053364.1	CUST_4631_PI421866198	NM_053364	Prl7d1	prolactin family 7, subfamily d, member 1	ENSRNOG00000016510	84377
19347	10	12	1	1	-1,276	-0,351	0,011308458	MRAK135567	MRAK135567_P1	MRAK135567	NA	lncRNA	NA	NA
14188	38	49	6	8	-1,276	-0,352	0,029540924	NM_001106036.1	CUST_14354_PI421866198	NM_001106036	Nrl	neural retina leucine zipper	ENSRNOG00000018528	290221
29830	53	67	9	9	-1,276	-0,352	0,035747444	MRAK051931	MRAK051931_P1	MRAK051931	NA	lncRNA	NA	NA
22332	397	512	54	103	-1,276	-0,352	0,0368925	AF124821	AF124821_P1	AF124821	NA	lncRNA	NA	NA
25913	10	13	1	3	-1,276	-0,352	0,037857253	NM_001009531.1	CUST_940_PI421866198	NM_001009531	NA	NA	NA	NA
42623	48	62	8	13	-1,276	-0,352	0,044705579	NM_001109458.1	CUST_1914_PI421866198	NM_001109458	Fam163b	family with sequence similarity 163, member B	ENSRNOG00000006591	685169
244	5929	7526	975	984	-1,277	-0,353	0,028195404	EF217429	EF217429_P1	EF217429	NA	lncRNA	NA	NA
6534	338	434	45	83	-1,278	-0,354	0,024133655	MRuc008jab	MRuc008jab_P1	MRuc008jab	NA	lncRNA	NA	NA
16182	30	38	7	6	-1,278	-0,354	0,049778938	BC059164	BC059164_P1	BC059164	NA	lncRNA	NA	NA
39825	24	31	2	4	-1,279	-0,355	0,006347291	NM_001108286.1	CUST_6689_PI421866198	NM_001108286	Ypel2	yippee-like 2	ENSRNOG00000005447	360590
26007	4171	5311	711	683	-1,279	-0,355	0,02076121	NM_001004206.1	CUST_3835_PI421866198	NM_001004206	Pa2g4	proliferation-associated 2G4	ENSRNOG00000004904	288778
1130	16	21	3	3	-1,279	-0,355	0,040213366	MRuc008dmy	MRuc008dmy_P1	MRuc008dmy	NA	lncRNA	NA	NA
4924	15	19	3	3	-1,279	-0,355	0,045987705	MRAK079345	MRAK079345_P1	MRAK079345	NA	lncRNA	NA	NA
41617	49	63	4	11	-1,28	-0,356	0,008674125	NM_001106560.1	CUST_10779_PI421866198	NM_001106560	Lcn9	lipocalin 9	ENSRNOG00000027821	296578
23598	118	150	19	13	-1,28	-0,356	0,009071802	NM_001000315.1	CUST_821_PI421866198	NM_001000315	Olr515	olfactory receptor 515	ENSRNOG00000032722	295768
20961	1558	1995	225	286	-1,28	-0,357	0,01918784	NM_001013860.1	CUST_9181_PI421866198	NM_001013860	Ptrh2	peptidyl-tRNA hydrolase 2	ENSRNOG00000004288	287593
14231	402	513	62	75	-1,28	-0,356	0,028416438	BC079432	BC079432_P1	BC079432	NA	lncRNA	NA	NA
19840	870	1118	126	195	-1,28	-0,356	0,031887784	NM_017304.2	CUST_11438_PI421866198	NM_017304	Kcnab2	potassium voltage-gated channel subfamily A regulatory beta subunit 2	ENSRNOG00000011550	29738
20106	1938	2454	401	306	-1,28	-0,356	0,033648264	NM_001106827.1	CUST_8911_PI421866198	NM_001106827	Celf6	CUGBP, Elav-like family member 6	ENSRNOG00000052224	300758
21856	2	3	0	0	-1,281	-0,357	0,015060719	NM_001000792.1	CUST_13663_PI421866198	NM_001000792	Olr1321	olfactory receptor 1321	ENSRNOG00000055420	405076
22653	27	35	4	6	-1,281	-0,357	0,025431021	NM_001000044.1	CUST_2654_PI421866198	NM_001000044	Olr1566	olfactory receptor 1566	ENSRNOG00000049950	NA
25809	2205	2840	328	559	-1,281	-0,357	0,036807364	NM_001037646.1	CUST_10417_PI421866198	NM_001037646	Fuz	fuzzy planar cell polarity protein	ENSRNOG00000053084	308577
14868	2723	3425	582	286	-1,281	-0,357	0,040429579	NM_001012091.1	CUST_2326_PI421866198	NM_001012091	Foxs1	forkhead box S1	ENSRNOG00000008529	311547
24360	162	205	35	33	-1,281	-0,357	0,048711523	XR_008816	XR_008816_P1	XR_008816	NA	lncRNA	NA	NA
6297	22	28	3	3	-1,282	-0,359	0,007905684	MRAK089184	MRAK089184_P1	MRAK089184	NA	lncRNA	NA	NA
15626	26	33	3	4	-1,282	-0,358	0,010751339	NM_001014099.1	CUST_10893_PI421866198	NM_001014099	Paqr8	progestin and adipoQ receptor family member 8	ENSRNOG00000012830	316275
18955	834	1066	135	143	-1,282	-0,358	0,021746906	NM_001108672.1	CUST_4133_PI421866198	NM_001108672	Bend5	BEN domain containing 5	ENSRNOG00000008025	362564
23023	34	44	4	8	-1,282	-0,358	0,023867185	MRAK053043	MRAK053043_P1	MRAK053043	NA	lncRNA	NA	NA
7080	17	22	3	4	-1,282	-0,358	0,03174375	BC158780	BC158780_P1	BC158780	NA	lncRNA	NA	NA
12813	23	29	4	5	-1,282	-0,359	0,031971933	NM_001000642.1	CUST_14484_PI421866198	NM_001000642	Olr646	olfactory receptor 646	ENSRNOG00000049365	404835
19468	1380	1728	345	95	-1,282	-0,358	0,041620045	NM_001107454.1	CUST_8210_PI421866198	NM_001107454	Nkd2	naked cuticle homolog 2	ENSRNOG00000016103	308068
44497	42	54	3	7	-1,283	-0,36	0,003943844	NM_001000361.1	CUST_679_PI421866198	NM_001000361	Olr718	olfactory receptor 718	ENSRNOG00000060711	295910
2604	30	39	1	8	-1,283	-0,359	0,008999024	MRAK144508	MRAK144508_P1	MRAK144508	NA	lncRNA	NA	NA
17497	43	56	6	7	-1,283	-0,359	0,01421674	uc.252+	uc.252+_P1	uc+	NA	lncRNA	NA	NA
18523	2258	2888	341	373	-1,283	-0,359	0,01595058	BC079173	BC079173_P1	BC079173	NA	lncRNA	NA	NA
15715	33	43	6	7	-1,283	-0,36	0,037250571	M15529	M15529_P1	M15529	NA	lncRNA	NA	NA
37953	13	17	2	4	-1,283	-0,359	0,04166981	uc.429+	uc.429+_P1	uc+	NA	lncRNA	NA	NA
35323	82	108	6	30	-1,283	-0,36	0,046640884	NM_001109413.1	CUST_2056_PI421866198	NM_001109413	Lrrc19	leucine rich repeat containing 19	ENSRNOG00000022225	680478
1111	131	172	17	47	-1,283	-0,36	0,047005902	XR_005724	XR_005724_P1	XR_005724	NA	lncRNA	NA	NA
19954	1788	2294	190	219	-1,284	-0,361	0,00372212	NM_001037210.1	CUST_13222_PI421866198	NM_001037210	Gipc2	GIPC PDZ domain containing family, member 2	ENSRNOG00000042152	365960
19586	500	638	75	41	-1,284	-0,361	0,008404519	NM_001034930.1	CUST_3569_PI421866198	NM_001034930	NA	NA	NA	NA
22833	17	22	2	3	-1,284	-0,361	0,012451461	NM_001001274.1	CUST_13279_PI421866198	NM_001001274	Olr141	olfactory receptor 141	ENSRNOG00000016946	293280
25525	30	39	2	10	-1,284	-0,36	0,028435396	XR_007703	XR_007703_P1	XR_007703	NA	lncRNA	NA	NA
1892	25	32	5	4	-1,284	-0,361	0,035674862	BC086367	BC086367_P1	BC086367	NA	lncRNA	NA	NA
2287	44	56	8	7	-1,284	-0,361	0,036421525	NM_001012168.1	CUST_1692_PI421866198	NM_001012168	Tulp2	tubby-like protein 2	ENSRNOG00000020927	361576
41414	298	382	58	66	-1,284	-0,36	0,038942659	NM_001025692.1	CUST_10361_PI421866198	NM_001025692	Sdcbp2	syndecan binding protein 2	ENSRNOG00000009528	311532
5439	12	15	2	2	-1,285	-0,362	0,011823339	L20989	L20989_P1	L20989	NA	lncRNA	NA	NA
26229	17	22	3	2	-1,285	-0,362	0,01470245	NM_001109221.1	CUST_3275_PI421866198	NM_001109221	Bmt2	base methyltransferase of 25S rRNA 2 homolog	ENSRNOG00000007224	NA
12745	37	49	3	12	-1,285	-0,362	0,029358873	NM_001166267.1	CUST_5835_PI421866198	NM_001166267	RGD1562885	NA	NA	NA
10644	119	156	10	38	-1,285	-0,361	0,029452411	MRAK090207	MRAK090207_P1	MRAK090207	NA	lncRNA	NA	NA
23952	100	128	17	21	-1,285	-0,362	0,02996759	NM_001106651.1	CUST_10044_PI421866198	NM_001106651	Snx30	sorting nexin family member 30	ENSRNOG00000017132	298033
24827	183	233	35	30	-1,285	-0,361	0,032262694	MRAK036857	MRAK036857_P1	MRAK036857	NA	lncRNA	NA	NA
36610	30	40	3	11	-1,285	-0,362	0,040365156	NM_001134620.1	CUST_6029_PI421866198	NM_001134620	Fndc11	fibronectin type III domain containing 11	ENSRNOG00000013243	NA
1159	662	836	159	94	-1,285	-0,362	0,045709483	MRAK037342	MRAK037342_P1	MRAK037342	NA	lncRNA	NA	NA
27441	954	1231	149	233	-1,286	-0,363	0,038607533	NM_022282.1	CUST_4380_PI421866198	NM_022282	Dlg2	discs large MAGUK scaffold protein 2	ENSRNOG00000022635	64053
33644	48	62	3	11	-1,287	-0,364	0,008447599	MRuc007uxo	MRuc007uxo_P1	MRuc007uxo	NA	lncRNA	NA	NA
572	14	17	3	1	-1,287	-0,364	0,022917309	XR_006919	XR_006919_P1	XR_006919	NA	lncRNA	NA	NA
24140	454	586	37	63	-1,288	-0,365	0,002022411	XR_008738	XR_008738_P1	XR_008738	NA	lncRNA	NA	NA
23332	50	66	5	19	-1,288	-0,365	0,046623415	BC085355	BC085355_P1	BC085355	NA	lncRNA	NA	NA
7060	61	79	9	5	-1,289	-0,366	0,005881318	MRAK142450	MRAK142450_P1	MRAK142450	NA	lncRNA	NA	NA
21477	167	216	24	35	-1,289	-0,366	0,014917203	MRAK081771	MRAK081771_P1	MRAK081771	NA	lncRNA	NA	NA
6714	26	33	4	4	-1,289	-0,367	0,018961195	NM_001109125.1	CUST_3936_PI421866198	NM_001109125	Znf474	NA	NA	NA
15441	8	10	1	1	-1,289	-0,366	0,022596305	NM_001099507.1	CUST_12968_PI421866198	NM_001099507	Vom2r50	vomeronasal 2 receptor, 50	ENSRNOG00000049200	500312
24967	16	20	3	4	-1,289	-0,366	0,03863228	NM_001108828.1	CUST_3009_PI421866198	NM_001108828	Rnf222	ring finger protein 222	ENSRNOG00000022964	363627
28194	1961	2519	436	489	-1,289	-0,366	0,0464207	NM_001012097.1	CUST_2320_PI421866198	NM_001012097	Atg7	autophagy related 7	ENSRNOG00000007486	312647
14914	33	43	4	7	-1,29	-0,367	0,010361975	U95155	U95155_P1	U95155	NA	lncRNA	NA	NA
28956	366	473	72	98	-1,29	-0,367	0,04139402	NM_001126085.1	CUST_11280_PI421866198	NM_001126085	Lsm6	LSM6 homolog, U6 small nuclear RNA and mRNA degradation associated	ENSRNOG00000030374	100360750
16338	115	147	29	22	-1,29	-0,367	0,044173377	MRAK080041	MRAK080041_P1	MRAK080041	NA	lncRNA	NA	NA
2875	58	74	10	3	-1,291	-0,369	0,006540275	MRAK132131	MRAK132131_P1	MRAK132131	NA	lncRNA	NA	NA
35548	445	578	48	94	-1,291	-0,369	0,010305239	NM_053897.2	CUST_1249_PI421866198	NM_053897	F2rl1	F2R like trypsin receptor 1	ENSRNOG00000018003	116677
27166	51	65	10	4	-1,291	-0,369	0,013336943	NM_012565.1	CUST_11016_PI421866198	NM_012565	Gck	glucokinase	ENSRNOG00000061527	24385
65	93	121	11	24	-1,291	-0,368	0,018279993	MRBC048639	MRBC048639_P1	MRBC048639	NA	lncRNA	NA	NA
29193	2	3	0	1	-1,291	-0,368	0,019347415	NM_001008916.1	CUST_1683_PI421866198	NM_001008916	NA	NA	NA	NA
34826	9	11	2	2	-1,291	-0,368	0,038241256	NM_001000821.1	CUST_13083_PI421866198	NM_001000821	Olr868	olfactory receptor 868	ENSRNOG00000047897	405110
25146	28	37	2	9	-1,292	-0,369	0,027632055	NM_017069.1	CUST_13325_PI421866198	NM_017069	Gabra3	gamma-aminobutyric acid type A receptor alpha3 subunit	ENSRNOG00000056558	24947
4545	499	634	110	70	-1,292	-0,37	0,041509085	NM_001106641.2	CUST_10061_PI421866198	NM_001106641	Rragd	Ras-related GTP binding D	ENSRNOG00000007331	297960
15386	57	74	6	9	-1,293	-0,371	0,005754405	MRAK042298	MRAK042298_P1	MRAK042298	NA	lncRNA	NA	NA
5774	46	59	7	6	-1,293	-0,371	0,009117604	NM_001008912.1	CUST_1687_PI421866198	NM_001008912	NA	NA	NA	NA
44108	76	99	10	15	-1,293	-0,371	0,012139702	NM_053726.2	CUST_2093_PI421866198	NM_053726	Slc7a10	solute carrier family 7 member 10	ENSRNOG00000010938	114518
22968	1789	2303	321	325	-1,293	-0,371	0,025676622	NM_001007725.1	CUST_6560_PI421866198	NM_001007725	Icam2	intercellular adhesion molecule 2	ENSRNOG00000025143	360647
25966	1000	1292	144	172	-1,294	-0,372	0,01483788	NM_053653.1	CUST_2785_PI421866198	NM_053653	Vegfc	vascular endothelial growth factor C	ENSRNOG00000011416	114111
22918	17	21	3	2	-1,295	-0,373	0,011329304	DQ203295	DQ203295_P1	DQ203295	NA	lncRNA	NA	NA
14680	66	86	9	11	-1,295	-0,373	0,01159159	NM_057155.3	CUST_7842_PI421866198	NM_057155	Xpnpep2	X-prolyl aminopeptidase 2	ENSRNOG00000004009	117522
15707	850	1091	155	141	-1,295	-0,373	0,026633656	NM_031634.1	CUST_11270_PI421866198	NM_031634	Mefv	MEFV, pyrin innate immunity regulator	ENSRNOG00000008134	58923
24111	3	3	1	0	-1,295	-0,373	0,046887907	MRAK138885	MRAK138885_P1	MRAK138885	NA	NA	NA	NA
2803	11	14	1	3	-1,296	-0,374	0,017027268	MRAK166328	MRAK166328_P1	MRAK166328	NA	lncRNA	NA	NA
6963	22	28	4	3	-1,296	-0,374	0,018002132	XR_008269	XR_008269_P1	XR_008269	NA	lncRNA	NA	NA
9439	125	163	21	30	-1,296	-0,374	0,026558702	MRAK049430	MRAK049430_P1	MRAK049430	NA	lncRNA	NA	NA
23997	777	1007	141	179	-1,296	-0,374	0,03153825	NM_001106555.1	CUST_10789_PI421866198	NM_001106555	C8g	complement C8 gamma chain	ENSRNOG00000028701	296545
7831	16	21	4	3	-1,296	-0,374	0,041527951	XR_008811	XR_008811_P1	XR_008811	NA	lncRNA	NA	NA
29230	9	12	1	3	-1,298	-0,377	0,03967033	NM_001000483.1	CUST_93_PI421866198	NM_001000483	Olr1344	olfactory receptor 1344	ENSRNOG00000033704	301610
15455	2	3	0	1	-1,298	-0,376	0,048659603	MRAK135461	MRAK135461_P1	MRAK135461	NA	NA	NA	NA
12021	1391	1802	151	143	-1,299	-0,377	0,002475964	NM_133304.1	CUST_7525_PI421866198	NM_133304	Heph	hephaestin	ENSRNOG00000012294	117240
40583	72	94	6	14	-1,299	-0,377	0,004753103	NM_001009503.1	CUST_978_PI421866198	NM_001009503	Pfn4	profilin family, member 4	ENSRNOG00000050386	494222
13592	44	57	6	7	-1,299	-0,377	0,008054022	NM_022216.1	CUST_4545_PI421866198	NM_022216	Gpr20	NA	NA	NA
20705	76	98	12	10	-1,299	-0,378	0,009064701	NM_053785.1	CUST_1939_PI421866198	NM_053785	Tm4sf4	transmembrane 4 L six family member 4	ENSRNOG00000016437	116467
22949	21	27	3	3	-1,299	-0,377	0,011487479	MRAK089395	MRAK089395_P1	MRAK089395	NA	lncRNA	NA	NA
8326	16	20	2	4	-1,299	-0,378	0,015789467	NM_001000733.1	CUST_13837_PI421866198	NM_001000733	Olr268	olfactory receptor 268	ENSRNOG00000053711	404989
15319	70	91	11	20	-1,299	-0,377	0,028693852	AJ286307	AJ286307_P1	AJ286307	NA	lncRNA	NA	NA
30230	103	134	19	24	-1,299	-0,377	0,028964166	NM_012564.2	CUST_11018_PI421866198	NM_012564	Gc	group specific component	ENSRNOG00000003119	24384
23450	396	516	65	99	-1,299	-0,378	0,030331144	NM_001139491.1	CUST_6150_PI421866198	NM_001139491	LOC680200	similar to zinc finger protein 455	ENSRNOG00000042308	680200
25463	1540	1982	304	270	-1,299	-0,377	0,030965445	NM_001105844.1	CUST_12514_PI421866198	NM_001105844	Higd1b	HIG1 hypoxia inducible domain family, member 1B	ENSRNOG00000002814	287738
2035	13	16	3	1	-1,299	-0,377	0,040250386	MRAK029887	MRAK029887_P1	MRAK029887	NA	lncRNA	NA	NA
9271	26	34	4	4	-1,3	-0,378	0,015221997	MRAK157480	MRAK157480_P1	MRAK157480	NA	lncRNA	NA	NA
7094	31	39	8	4	-1,3	-0,379	0,033532472	MRAK048943	MRAK048943_P1	MRAK048943	NA	lncRNA	NA	NA
17595	1231	1595	150	112	-1,301	-0,38	0,002439515	NM_001108509.2	CUST_4945_PI421866198	NM_001108509	Pnpla2	patatin-like phospholipase domain containing 2	ENSRNOG00000018736	361676
4510	102	131	15	13	-1,301	-0,38	0,008957468	L11024	L11024_P1	L11024	NA	lncRNA	NA	NA
20817	1590	2064	234	282	-1,301	-0,38	0,015420032	NM_172055.2	CUST_7566_PI421866198	NM_172055	Cited1	Cbp/p300-interacting transactivator with Glu/Asp-rich carboxy-terminal domain 1	ENSRNOG00000003189	64466
16098	3	3	0	0	-1,301	-0,38	0,016217863	XR_005710	XR_005710_P1	XR_005710	NA	NA	NA	NA
26151	16	21	3	3	-1,301	-0,38	0,032123464	uc.96+	uc.96+_P1	uc+	NA	lncRNA	NA	NA
36032	93	119	24	18	-1,301	-0,38	0,045695356	uc.212+	uc.212+_P1	uc+	NA	lncRNA	NA	NA
19971	2	3	0	1	-1,302	-0,381	0,035464442	NM_139088.1	CUST_5100_PI421866198	NM_139088	Impg2	interphotoreceptor matrix proteoglycan 2	ENSRNOG00000001622	245919
30551	69	91	8	25	-1,303	-0,382	0,039711776	NM_001001016.1	CUST_14898_PI421866198	NM_001001016	Olr1174	olfactory receptor 1174	ENSRNOG00000049712	405390
35580	1368	1766	240	156	-1,304	-0,383	0,013185266	NM_053399.1	CUST_4529_PI421866198	NM_053399	Nrtn	neurturin	ENSRNOG00000048651	84423
11958	6	7	1	1	-1,304	-0,383	0,032265516	MRAK140701	MRAK140701_P1	MRAK140701	NA	lncRNA	NA	NA
24447	559	728	78	98	-1,305	-0,384	0,010470885	NM_138882.1	CUST_3382_PI421866198	NM_138882	Pla1a	phospholipase A1 member A	ENSRNOG00000057153	85311
3144	13	17	2	1	-1,305	-0,384	0,014474094	AJ286152	AJ286152_P1	AJ286152	NA	lncRNA	NA	NA
22830	38	50	5	8	-1,305	-0,384	0,018364788	NM_001105751.1	CUST_13142_PI421866198	NM_001105751	Hivep1	human immunodeficiency virus type I enhancer binding protein 1	ENSRNOG00000014460	NA
24560	16	21	3	4	-1,305	-0,384	0,028558167	XR_007957	XR_007957_P1	XR_007957	NA	lncRNA	NA	NA
20641	10	13	1	3	-1,305	-0,384	0,041080968	NM_001108571.1	CUST_4866_PI421866198	NM_001108571	Il1f10	interleukin 1 family member 10	ENSRNOG00000005800	362077
21808	53	70	7	15	-1,306	-0,385	0,03098528	MRuc007tcl	MRuc007tcl_P1	MRuc007tcl	NA	lncRNA	NA	NA
13792	125	164	20	33	-1,306	-0,385	0,036170831	BC166992	BC166992_P1	BC166992	NA	lncRNA	NA	NA
2131	34	45	4	12	-1,306	-0,385	0,038702846	XR_007267	XR_007267_P1	XR_007267	NA	lncRNA	NA	NA
24180	60	79	9	13	-1,307	-0,386	0,01476887	XR_008987	XR_008987_P1	XR_008987	NA	lncRNA	NA	NA
4638	61	81	6	19	-1,307	-0,386	0,02139917	BC160868	BC160868_P1	BC160868	NA	lncRNA	NA	NA
43618	52	67	9	11	-1,307	-0,386	0,023968142	uc.113+	uc.113+_P1	uc+	NA	lncRNA	NA	NA
13974	5	7	1	1	-1,307	-0,387	0,030045944	MRAK006276	MRAK006276_P1	MRAK006276	NA	NA	NA	NA
16914	833	1087	115	139	-1,308	-0,388	0,007362428	BC091404	BC091404_P1	BC091404	NA	lncRNA	NA	NA
22410	101	131	20	24	-1,308	-0,387	0,036050039	L11029	L11029_P1	L11029	NA	lncRNA	NA	NA
25624	22	29	4	7	-1,308	-0,387	0,043354914	NM_001144850.1	CUST_10596_PI421866198	NM_001144850	Psapl1	prosaposin-like 1	ENSRNOG00000006845	289720
11305	222	289	33	27	-1,31	-0,39	0,006604503	NM_001100993.1	CUST_12693_PI421866198	NM_001100993	Stpg2	sperm-tail PG-rich repeat containing 2	ENSRNOG00000023457	499719
23301	2	3	0	1	-1,31	-0,39	0,028695006	NM_001000970.1	CUST_12330_PI421866198	NM_001000970	Olr1635	olfactory receptor 1635	ENSRNOG00000031108	405124
12609	22	29	2	5	-1,311	-0,391	0,005769507	MRAK006042	MRAK006042_P1	MRAK006042	NA	lncRNA	NA	NA
38573	59	77	13	12	-1,311	-0,391	0,028308818	NM_001106394.1	CUST_12033_PI421866198	NM_001106394	Nodal	nodal growth differentiation factor	ENSRNOG00000000556	294503
8400	100	133	15	37	-1,311	-0,391	0,039341445	BC111073	BC111073_P1	BC111073	NA	lncRNA	NA	NA
20324	2	3	0	0	-1,312	-0,392	0,001398939	AF227190	AF227190_P1	AF227190	NA	NA	NA	NA
31742	2	3	0	1	-1,312	-0,392	0,01274852	NM_001109127.1	CUST_3934_PI421866198	NM_001109127	Cbln1	cerebellin 1 precursor	ENSRNOG00000000010	498922
5004	13	16	2	2	-1,312	-0,392	0,0149773	XR_007150	XR_007150_P1	XR_007150	NA	lncRNA	NA	NA
19735	44	58	7	8	-1,312	-0,392	0,018344601	XR_006411	XR_006411_P1	XR_006411	NA	lncRNA	NA	NA
27108	10	13	2	3	-1,312	-0,391	0,047578688	NM_001000531.1	CUST_15052_PI421866198	NM_001000531	Olr1542	olfactory receptor 1542	ENSRNOG00000046780	363763
25180	3	4	0	0	-1,313	-0,393	0,010048473	MRAK086417	MRAK086417_P1	MRAK086417	NA	NA	NA	NA
38636	48	63	8	11	-1,313	-0,393	0,018853807	NM_001000034.1	CUST_2701_PI421866198	NM_001000034	Olr1511	olfactory receptor 1511	ENSRNOG00000050608	287509
17170	88	118	9	30	-1,313	-0,392	0,024045249	MRAK137618	MRAK137618_P1	MRAK137618	NA	lncRNA	NA	NA
20905	1169	1528	213	247	-1,313	-0,393	0,025496862	NM_001033913.1	CUST_7089_PI421866198	NM_001033913	Gimap5	GTPase, IMAP family member 5	ENSRNOG00000008416	246774
25379	10	13	2	2	-1,313	-0,392	0,03308707	MRAK144469	MRAK144469_P1	MRAK144469	NA	lncRNA	NA	NA
22433	18	24	3	3	-1,314	-0,394	0,010622104	MRuc008wog	MRuc008wog_P1	MRuc008wog	NA	lncRNA	NA	NA
21917	55	73	5	15	-1,315	-0,395	0,011332496	MRAK034248	MRAK034248_P1	MRAK034248	NA	lncRNA	NA	NA
1551	160	211	32	52	-1,315	-0,395	0,04336516	BC088288	BC088288_P1	BC088288	NA	lncRNA	NA	NA
15292	10	13	1	2	-1,316	-0,396	0,004269888	NM_017170.2	CUST_12639_PI421866198	NM_017170	Apcs	amyloid P component, serum	ENSRNOG00000009086	NA
18262	26	34	3	6	-1,316	-0,396	0,009965616	AF087697	AF087697_P1	AF087697	NA	lncRNA	NA	NA
20565	11	15	1	3	-1,316	-0,396	0,020145584	MRBC094251	MRBC094251_P1	MRBC094251	NA	lncRNA	NA	NA
41351	30	40	5	9	-1,316	-0,396	0,028099026	NM_001008935.1	CUST_1624_PI421866198	NM_001008935	NA	NA	NA	NA
3383	48	62	10	10	-1,316	-0,396	0,039763384	AY383702	AY383702_P1	AY383702	NA	lncRNA	NA	NA
38724	75	98	9	9	-1,317	-0,398	0,00258337	NM_001108823.1	CUST_3015_PI421866198	NM_001108823	Zg16b	NA	NA	NA
28300	16	21	3	5	-1,317	-0,398	0,038264153	NM_001108890.1	CUST_2917_PI421866198	NM_001108890	Ticam2	toll-like receptor adaptor molecule 2	ENSRNOG00000042070	364867
12946	79	102	21	15	-1,317	-0,397	0,043121399	MRAK031562	MRAK031562_P1	MRAK031562	NA	lncRNA	NA	NA
38707	47	63	5	9	-1,318	-0,398	0,003855777	NM_001108760.1	CUST_3461_PI421866198	NM_001108760	Mpzl3	myelin protein zero-like 3	ENSRNOG00000026753	363054
18263	23	30	3	4	-1,318	-0,398	0,007920767	NM_001000996.1	CUST_12255_PI421866198	NM_001000996	Olr766	olfactory receptor 766	ENSRNOG00000050138	405356
14004	35	47	7	10	-1,318	-0,399	0,043032002	NM_012874.1	CUST_8977_PI421866198	NM_012874	Ros1	ROS proto-oncogene 1 , receptor tyrosine kinase	ENSRNOG00000000406	25346
3295	16	21	2	3	-1,319	-0,399	0,005307524	MRAK036231	MRAK036231_P1	MRAK036231	NA	lncRNA	NA	NA
15619	105	137	19	12	-1,319	-0,4	0,007343191	NM_001001422.1	CUST_12084_PI421866198	NM_001001422	Olr1737	olfactory receptor 1737	ENSRNOG00000048048	405349
15711	33	44	6	5	-1,319	-0,399	0,019518893	XR_006559	XR_006559_P1	XR_006559	NA	lncRNA	NA	NA
23695	572	756	100	152	-1,319	-0,399	0,040585895	NM_001014230.1	CUST_9507_PI421866198	NM_001014230	Iqcg	IQ motif containing G	ENSRNOG00000026420	363796
4342	67	88	16	17	-1,319	-0,399	0,041889524	AY845221	AY845221_P1	AY845221	NA	lncRNA	NA	NA
29280	73	97	15	21	-1,319	-0,399	0,048938677	NM_001014271.1	CUST_9422_PI421866198	NM_001014271	LOC367515	NA	NA	NA
20930	94	126	15	37	-1,319	-0,399	0,049136156	MRuc008bfj	MRuc008bfj_P1	MRuc008bfj	NA	lncRNA	NA	NA
38458	23	31	2	3	-1,32	-0,401	0,000691952	NM_001077681.1	CUST_6141_PI421866198	NM_001077681	Tex47	NA	NA	NA
24156	2	3	0	1	-1,32	-0,401	0,022957033	NM_031650.1	CUST_11236_PI421866198	NM_031650	Slco1b2	solute carrier organic anion transporter family, member 1B2	ENSRNOG00000030538	58978
4604	151	199	36	41	-1,32	-0,401	0,042400261	BC098708	BC098708_P1	BC098708	NA	lncRNA	NA	NA
14721	17	23	2	4	-1,321	-0,402	0,010676019	U61747	U61747_P1	U61747	NA	lncRNA	NA	NA
10719	48	63	8	10	-1,321	-0,401	0,014420274	XR_006458	XR_006458_P1	XR_006458	NA	lncRNA	NA	NA
42141	29	38	7	6	-1,321	-0,402	0,031477286	NM_001008554.1	CUST_4353_PI421866198	NM_001008554	Tmprss11g	transmembrane protease, serine 11G	ENSRNOG00000029964	289546
6327	7	10	1	1	-1,322	-0,403	0,003649585	MRAK080830	MRAK080830_P1	MRAK080830	NA	lncRNA	NA	NA
17850	30	39	4	5	-1,322	-0,403	0,005297522	NM_019273.1	CUST_5914_PI421866198	NM_019273	Kcnmb1	potassium calcium-activated channel subfamily M regulatory beta subunit 1	ENSRNOG00000005465	29747
12557	310	409	52	57	-1,322	-0,403	0,013936383	NM_001106957.1	CUST_8354_PI421866198	NM_001106957	Glod5	glyoxalase domain containing 5	ENSRNOG00000039571	302554
23502	1052	1376	224	173	-1,323	-0,404	0,020109181	NM_001012214.1	CUST_1248_PI421866198	NM_001012214	Serpinb6b	serine (or cysteine) peptidase inhibitor, clade B, member 6b	ENSRNOG00000016420	364705
11739	33	45	4	11	-1,323	-0,404	0,027343974	MRAK006976	MRAK006976_P1	MRAK006976	NA	lncRNA	NA	NA
25516	19	25	5	4	-1,323	-0,403	0,038493499	MRAK142869	MRAK142869_P1	MRAK142869	NA	lncRNA	NA	NA
6290	18	23	2	4	-1,324	-0,404	0,012156939	MRAK034750	MRAK034750_P1	MRAK034750	NA	lncRNA	NA	NA
18188	22	30	2	6	-1,324	-0,405	0,014061907	NM_001000551.1	CUST_15030_PI421866198	NM_001000551	Olr218	olfactory receptor 218	ENSRNOG00000048022	365340
9435	49	64	11	9	-1,325	-0,406	0,027724649	MRAK158907	MRAK158907_P1	MRAK158907	NA	lncRNA	NA	NA
12829	953	1265	163	221	-1,326	-0,408	0,021531807	NM_012613.1	CUST_10434_PI421866198	NM_012613	Npr1	natriuretic peptide receptor 1	ENSRNOG00000014684	24603
33780	26	34	3	3	-1,327	-0,408	0,001478506	MRAK041069	MRAK041069_P1	MRAK041069	NA	lncRNA	NA	NA
12968	36	48	5	5	-1,327	-0,408	0,004899677	MRAK089484	MRAK089484_P1	MRAK089484	NA	lncRNA	NA	NA
23773	52	70	5	17	-1,327	-0,408	0,018585467	NM_053333.1	CUST_4723_PI421866198	NM_053333	Retnla	resistin like alpha	ENSRNOG00000001955	81712
41275	20	27	1	4	-1,328	-0,41	0,002067386	NM_001000847.1	CUST_13036_PI421866198	NM_001000847	Olr812	olfactory receptor 812	ENSRNOG00000005407	405137
25627	1582	2087	271	237	-1,328	-0,409	0,007317245	NM_001008354.1	CUST_5575_PI421866198	NM_001008354	Mrrf	mitochondrial ribosome recycling factor	ENSRNOG00000025997	311903
23455	104	139	12	23	-1,329	-0,41	0,007410435	MRAK077312	MRAK077312_P1	MRAK077312	NA	lncRNA	NA	NA
23853	56	73	15	8	-1,329	-0,41	0,031589866	MRuc009jeh	MRuc009jeh_P1	MRuc009jeh	NA	lncRNA	NA	NA
22400	251	331	56	61	-1,329	-0,41	0,044936958	NM_020305.1	CUST_9701_PI421866198	NM_020305	Adam4	a disintegrin and metalloprotease domain 4	ENSRNOG00000038275	57022
27247	31	40	8	7	-1,329	-0,41	0,046465766	MRAK158075	MRAK158075_P1	MRAK158075	NA	lncRNA	NA	NA
29965	3964	5271	352	463	-1,33	-0,412	0,000821406	NM_001009709.1	CUST_15187_PI421866198	NM_001009709	Tmem140	transmembrane protein 140	ENSRNOG00000026965	362334
18100	7	10	1	2	-1,33	-0,412	0,012866541	MRuc009dte	MRuc009dte_P1	MRuc009dte	NA	lncRNA	NA	NA
19519	79	105	14	18	-1,33	-0,412	0,017367781	MRAK046975	MRAK046975_P1	MRAK046975	NA	lncRNA	NA	NA
23933	789	1045	155	168	-1,33	-0,411	0,020339198	NM_139102.2	CUST_4661_PI421866198	NM_139102	Dmgdh	dimethylglycine dehydrogenase	ENSRNOG00000023588	245961

23201	357	468	90	72	-1,33	-0,412	0,035578476	MRAK028522	MRAK028522_P1	MRAK028522	NA	lncRNA	NA	NA
27511	139	191	14	62	-1,33	-0,411	0,046539216	BC097389	BC097389_P1	BC097389	NA	lncRNA	NA	NA
23029	59	79	7	6	-1,331	-0,412	0,001844851	NM_057108.2	CUST_7946_PI421866198	NM_057108	Serpinb5	serpin family B member 5	ENSRNOG00000002640	116589
16811	46	61	5	9	-1,331	-0,412	0,005045305	NM_175591.2	CUST_9357_PI421866198	NM_175591	Taar7d	trace-amine-associated receptor 7d	ENSRNOG00000045914	294134
15121	688	931	63	221	-1,331	-0,413	0,01252794	NM_133591.2	CUST_6287_PI421866198	NM_133591	Rph3al	rabphilin 3A-like (without C2 domains)	ENSRNOG00000061429	171123
34376	20	27	4	5	-1,331	-0,413	0,0205916	NM_001106721.1	CUST_9370_PI421866198	NM_001106721	NA	NA	NA	NA
22784	2	3	0	1	-1,331	-0,413	0,03382637	NM_001000274.1	CUST_1165_PI421866198	NM_001000274	Olr1690	olfactory receptor 1690	ENSRNOG00000046933	294162
43931	48	62	14	9	-1,331	-0,413	0,039462624	NM_001113753.1	CUST_10340_PI421866198	NM_001113753	NA	NA	NA	NA
15216	21	27	5	5	-1,331	-0,412	0,042502814	MRAK044571	MRAK044571_P1	MRAK044571	NA	lncRNA	NA	NA
2640	31	41	6	4	-1,332	-0,413	0,012108513	MRAK015925	MRAK015925_P1	MRAK015925	NA	lncRNA	NA	NA
35824	51	68	6	9	-1,333	-0,414	0,004427446	uc.452+	uc.452+_P1	uc+	NA	lncRNA	NA	NA
27561	35	49	3	16	-1,333	-0,415	0,04140897	XR_006066	XR_006066_P1	XR_006066	NA	lncRNA	NA	NA
14448	2	3	0	1	-1,333	-0,415	0,043737908	NM_001109466.1	CUST_1871_PI421866198	NM_001109466	Fmo9	flavin containing monooxygenase 9	ENSRNOG00000025119	685365
32728	72	97	4	19	-1,334	-0,416	0,004978869	MRuc008emz	MRuc008emz_P1	MRuc008emz	NA	lncRNA	NA	NA
1470	2	3	0	1	-1,334	-0,416	0,01052401	CUST_4_PI415351601	CUST_4_PI415351601	CUST_4_PI415351601	NA	NA	NA	NA
32940	134	186	9	61	-1,334	-0,416	0,04133326	NM_001017988.1	CUST_11077_PI421866198	NM_001017988	LOC303566	E2F1-inducible gene	ENSRNOG00000020908	303566
10756	10	13	1	3	-1,335	-0,416	0,029536215	L11033	L11033_P1	L11033	NA	lncRNA	NA	NA
10180	2	3	0	1	-1,335	-0,417	0,038449552	MRAK049786	MRAK049786_P1	MRAK049786	NA	NA	NA	NA
8949	16	21	3	1	-1,336	-0,418	0,00638036	MRAK141453	MRAK141453_P1	MRAK141453	NA	lncRNA	NA	NA
37838	74	99	15	21	-1,336	-0,418	0,033513531	NM_001106807.1	CUST_8931_PI421866198	NM_001106807	Zfp653	zinc finger protein 653	ENSRNOG00000014065	300446
27281	31	41	4	2	-1,337	-0,419	0,001957937	MRuc007ldt	MRuc007ldt_P1	MRuc007ldt	NA	lncRNA	NA	NA
26835	40	53	7	7	-1,337	-0,42	0,008674075	NM_001107679.1	CUST_7380_PI421866198	NM_001107679	NA	NA	NA	NA
42338	13	17	2	4	-1,337	-0,419	0,023353983	MRAK132214	MRAK132214_P1	MRAK132214	NA	lncRNA	NA	NA
20020	7	10	1	3	-1,337	-0,419	0,038415409	XR_007038	XR_007038_P1	XR_007038	NA	lncRNA	NA	NA
28438	15	20	2	2	-1,338	-0,42	0,001661978	MRAK034786	MRAK034786_P1	MRAK034786	NA	lncRNA	NA	NA
19943	35	47	4	8	-1,338	-0,42	0,00669087	NM_001109201.1	CUST_3315_PI421866198	NM_001109201	RGD1563263	similar to RIKEN cDNA 1700029I15	ENSRNOG00000006884	499836
9680	36	48	7	5	-1,338	-0,42	0,014084561	NM_001000118.1	CUST_2008_PI421866198	NM_001000118	Olr20	olfactory receptor 20	ENSRNOG00000045648	293083
17082	92	123	21	26	-1,338	-0,42	0,041514524	NM_001122658.1	CUST_6030_PI421866198	NM_001122658	Fam178b	family with sequence similarity 178, member B	ENSRNOG00000023941	501125
6371	63	83	16	17	-1,338	-0,421	0,045189919	MRAK141533	MRAK141533_P1	MRAK141533	NA	lncRNA	NA	NA
33789	30	42	2	13	-1,338	-0,42	0,045485556	NM_001107572.1	CUST_7751_PI421866198	NM_001107572	Ovol1	ovo like transcriptional repressor 1	ENSRNOG00000020669	309164
11800	28	37	5	3	-1,339	-0,421	0,004857419	X05701	X05701_P1	X05701	NA	lncRNA	NA	NA
18184	191	257	39	69	-1,339	-0,421	0,045234146	NM_001106582.1	CUST_10713_PI421866198	NM_001106582	Gpr165	G protein-coupled receptor 165	ENSRNOG00000012995	296866
15107	141	186	32	19	-1,34	-0,422	0,012781543	NM_001108912.1	CUST_2244_PI421866198	NM_001108912	Ffar3	free fatty acid receptor 3	ENSRNOG00000037467	365228
30054	919	1217	196	169	-1,34	-0,422	0,019224945	NM_031547.1	CUST_11971_PI421866198	NM_031547	Chrm4	cholinergic receptor, muscarinic 4	ENSRNOG00000017556	25111
20879	195	261	39	48	-1,34	-0,423	0,033702871	NM_001106906.1	CUST_8425_PI421866198	NM_001106906	Gpr45	G protein-coupled receptor 45	ENSRNOG00000016446	301372
26436	789	1033	243	145	-1,34	-0,422	0,039474948	NM_001130013.1	CUST_7308_PI421866198	NM_001130013	Cdc42bpg	CDC42 binding protein kinase gamma	ENSRNOG00000027456	293693
33996	67	88	18	12	-1,34	-0,422	0,046186327	uc.300-	uc.300-_P1	uc-	NA	lncRNA	NA	NA
22027	1609	2160	231	357	-1,341	-0,424	0,008177307	NM_001107942.2	CUST_5785_PI421866198	NM_001107942	Lad1	ladinin 1	ENSRNOG00000009144	313325
35348	53	71	9	11	-1,341	-0,423	0,011455326	MRAK050022	MRAK050022_P1	MRAK050022	NA	lncRNA	NA	NA
14876	329	434	78	64	-1,341	-0,423	0,038769429	NM_001013044.1	CUST_14735_PI421866198	NM_001013044	Cryzl1	crystallin zeta like 1	ENSRNOG00000028014	288256
41041	122	162	35	29	-1,341	-0,423	0,041363147	NM_001009175.1	CUST_3331_PI421866198	NM_001009175	Trim40	tripartite motif-containing 40	ENSRNOG00000000783	365528
23320	546	719	118	42	-1,342	-0,425	0,014962564	NM_001108400.2	CUST_5544_PI421866198	NM_001108400	Ankrd37	ankyrin repeat domain 37	ENSRNOG00000031335	361149
14292	13	17	3	3	-1,343	-0,426	0,043986406	MRAK162039	MRAK162039_P1	MRAK162039	NA	lncRNA	NA	NA
1867	25	33	4	4	-1,344	-0,427	0,00583181	NM_133516.1	CUST_6468_PI421866198	NM_133516	Svs5	seminal vesicle secretory protein 5	ENSRNOG00000055535	NA
23594	424	568	70	83	-1,344	-0,426	0,01361653	NM_001009497.1	CUST_1411_PI421866198	NM_001009497	Ly49si1	immunoreceptor Ly49si1	ENSRNOG00000061895	494206
34217	29	39	7	7	-1,344	-0,427	0,027916105	NM_023973.1	CUST_11858_PI421866198	NM_023973	Ido1	indoleamine 2,3-dioxygenase 1	ENSRNOG00000031189	66029
38070	16	21	4	4	-1,344	-0,427	0,029532938	NM_001003691.1	CUST_4670_PI421866198	NM_001003691	Ly6g6f	lymphocyte antigen 6 complex, locus G6F	ENSRNOG00000027225	309609
13959	53	73	9	17	-1,344	-0,427	0,031293398	J01878	J01878_P1	J01878	NA	lncRNA	NA	NA
19782	15	21	3	4	-1,344	-0,427	0,03433187	MRAK033161	MRAK033161_P1	MRAK033161	NA	lncRNA	NA	NA
32343	75	104	8	35	-1,344	-0,427	0,043366121	NM_001134765.1	CUST_5235_PI421866198	NM_001134765	Oc90	otoconin 90	ENSRNOG00000025516	690625
17270	35	47	6	7	-1,345	-0,427	0,010237134	AM293751	AM293751_P1	AM293751	NA	lncRNA	NA	NA
21378	638	854	109	125	-1,345	-0,427	0,018563762	NM_001106952.1	CUST_8360_PI421866198	NM_001106952	Efhc2	EF-hand domain containing 2	ENSRNOG00000002986	302507
24334	108	144	30	28	-1,345	-0,427	0,039165701	BC160863	BC160863_P1	BC160863	NA	lncRNA	NA	NA
17188	63	82	20	11	-1,345	-0,427	0,040471426	MRAK038400	MRAK038400_P1	MRAK038400	NA	lncRNA	NA	NA
21995	2479	3358	266	597	-1,346	-0,429	0,005946165	NM_001108098.1	CUST_7603_PI421866198	NM_001108098	Cpm	carboxypeptidase M	ENSRNOG00000034134	314855
38633	35	47	5	10	-1,346	-0,429	0,015495189	NM_001130575.1	CUST_4086_PI421866198	NM_001130575	Rspo2	R-spondin 2	ENSRNOG00000037687	500863
22597	60	81	7	16	-1,347	-0,43	0,009858543	NM_001001083.1	CUST_14716_PI421866198	NM_001001083	Olr1225	olfactory receptor 1225	ENSRNOG00000059901	405974
11919	8	11	1	2	-1,347	-0,43	0,018188278	MRuc009iks	MRuc009iks_P1	MRuc009iks	NA	lncRNA	NA	NA
31773	128	173	21	39	-1,347	-0,429	0,021190969	NM_001144860.1	CUST_10563_PI421866198	NM_001144860	Tmem242	transmembrane protein 242	ENSRNOG00000016973	292228
9860	31	42	3	9	-1,348	-0,431	0,01292598	MRAK158670	MRAK158670_P1	MRAK158670	NA	lncRNA	NA	NA
22024	2	3	0	1	-1,348	-0,431	0,015125625	uc.446-	uc.446-_P1	uc-	NA	NA	NA	NA
19147	23	31	4	5	-1,348	-0,431	0,016720302	MRuc009nlv	MRuc009nlv_P1	MRuc009nlv	NA	lncRNA	NA	NA
23941	6	7	1	1	-1,348	-0,431	0,030408172	AY387089	AY387089_P1	AY387089	NA	lncRNA	NA	NA
22875	2	3	0	1	-1,348	-0,431	0,041988043	NM_001134622.1	CUST_6027_PI421866198	NM_001134622	Ccser1	coiled-coil serine-rich protein 1	ENSRNOG00000029321	500153
25475	18	25	1	5	-1,349	-0,432	0,007527901	NM_001009174.1	CUST_3332_PI421866198	NM_001009174	Prss58	protease, serine, 58	ENSRNOG00000013132	408204
1768	8	10	2	2	-1,349	-0,432	0,024026097	MRAK036094	MRAK036094_P1	MRAK036094	NA	lncRNA	NA	NA
20453	21	29	3	6	-1,35	-0,433	0,026902551	NM_198972.1	CUST_10272_PI421866198	NM_198972	Gkn1	gastrokine 1	ENSRNOG00000008838	297418
33400	266	350	74	49	-1,35	-0,433	0,043732822	NM_019230.1	CUST_6017_PI421866198	NM_019230	Slc22a3	solute carrier family 22 member 3	ENSRNOG00000022946	29504
7910	48	65	9	12	-1,351	-0,434	0,017844703	XR_008234	XR_008234_P1	XR_008234	NA	lncRNA	NA	NA
23480	356	476	59	34	-1,352	-0,435	0,006103199	NM_031741.1	CUST_10631_PI421866198	NM_031741	Slc2a5	solute carrier family 2 member 5	ENSRNOG00000017693	65197
2687	13	17	2	4	-1,352	-0,436	0,023037515	AF083329	AF083329_P1	AF083329	NA	lncRNA	NA	NA
20311	11	15	2	3	-1,352	-0,435	0,025899289	MRuc007lta	MRuc007lta_P1	MRuc007lta	NA	lncRNA	NA	NA
20703	68	93	11	22	-1,352	-0,435	0,037057408	NM_001012019.1	CUST_2627_PI421866198	NM_001012019	Tssk2	testis-specific serine kinase 2	ENSRNOG00000037997	NA
29633	229	306	49	33	-1,353	-0,436	0,013484278	NM_012550.2	CUST_11048_PI421866198	NM_012550	Ednra	endothelin receptor type A	ENSRNOG00000012721	24326
17330	469	639	72	123	-1,353	-0,437	0,013914454	NM_001037773.3	CUST_9566_PI421866198	NM_001037773	Ccnjl	cyclin J-like	ENSRNOG00000003893	303059
25120	41	55	9	10	-1,353	-0,436	0,026928179	XR_006699	XR_006699_P1	XR_006699	NA	lncRNA	NA	NA
14338	16	21	3	5	-1,353	-0,437	0,044045109	AJ010024	AJ010024_P1	AJ010024	NA	lncRNA	NA	NA
15976	35	47	4	5	-1,354	-0,437	0,000879453	NM_001000726.1	CUST_13852_PI421866198	NM_001000726	Olr1555	olfactory receptor 1555	ENSRNOG00000058137	404980
9555	20	26	4	3	-1,354	-0,438	0,021680252	MRAK140086	MRAK140086_P1	MRAK140086	NA	lncRNA	NA	NA
22100	13	18	2	5	-1,354	-0,437	0,031576176	NM_001105922.1	CUST_11748_PI421866198	NM_001105922	Fkbp6	FK506 binding protein 6	ENSRNOG00000001451	288597
36712	52	69	16	15	-1,354	-0,437	0,048537138	NM_012854.2	CUST_9002_PI421866198	NM_012854	Il10	interleukin 10	ENSRNOG00000004647	25325
36596	47	63	6	11	-1,355	-0,438	0,005988971	NM_001000881.1	CUST_12984_PI421866198	NM_001000881	Olr1125	olfactory receptor 1125	ENSRNOG00000026489	405180
15030	23	30	5	3	-1,355	-0,438	0,013694613	NM_001105753.1	CUST_13140_PI421866198	NM_001105753	Rasgrf1	RAS protein-specific guanine nucleotide-releasing factor 1	ENSRNOG00000014025	192213
4141	91	123	22	26	-1,355	-0,438	0,036452115	XR_009503	XR_009503_P1	XR_009503	NA	lncRNA	NA	NA
19371	13	17	2	4	-1,355	-0,438	0,038370199	NM_001106105.1	CUST_13713_PI421866198	NM_001106105	Gcm2	glial cells missing homolog 2	ENSRNOG00000015008	291047
15918	9	11	1	1	-1,356	-0,439	0,006140029	MRBC039874	MRBC039874_P1	MRBC039874	NA	lncRNA	NA	NA
15993	11	14	3	2	-1,356	-0,439	0,043128377	L07396	L07396_P1	L07396	NA	lncRNA	NA	NA
12095	16	21	3	3	-1,357	-0,441	0,016654019	MRAK163699	MRAK163699_P1	MRAK163699	NA	lncRNA	NA	NA
35674	94	124	31	19	-1,357	-0,441	0,043809481	NM_001109490.1	CUST_1761_PI421866198	NM_001109490	LOC686031	hypothetical protein LOC686031	ENSRNOG00000047036	686031
37520	18	24	3	3	-1,358	-0,442	0,002816167	NM_001108917.1	CUST_2235_PI421866198	NM_001108917	Lipt2	lipoyl(octanoyl) transferase 2 (putative)	ENSRNOG00000016906	365314
24342	291	391	53	53	-1,358	-0,441	0,014875709	NM_024000.1	CUST_14555_PI421866198	NM_024000	Camkv	CaM kinase-like vesicle-associated	ENSRNOG00000058938	79011
35349	16	21	3	5	-1,358	-0,441	0,0241498	BC129124	BC129124_P1	BC129124	NA	lncRNA	NA	NA
13170	2	3	0	1	-1,358	-0,442	0,034633691	NM_001000229.1	CUST_1339_PI421866198	NM_001000229	Olr270	olfactory receptor 270	ENSRNOG00000060915	293437
24866	14	20	2	6	-1,359	-0,443	0,038997245	MRAK053163	MRAK053163_P1	MRAK053163	NA	lncRNA	NA	NA
17967	24	32	5	7	-1,36	-0,444	0,027093033	MRAK043342	MRAK043342_P1	MRAK043342	NA	lncRNA	NA	NA
33488	3563	4875	506	917	-1,361	-0,445	0,010047996	NM_153734.1	CUST_2554_PI421866198	NM_153734	Cst12	cystatin 12	ENSRNOG00000004946	266776
12325	4	6	1	1	-1,361	-0,445	0,029372405	XR_008406	XR_008406_P1	XR_008406	NA	NA	NA	NA
18006	149	206	24	59	-1,361	-0,444	0,032185395	MRuc009thv	MRuc009thv_P1	MRuc009thv	NA	lncRNA	NA	NA
24361	40	54	9	5	-1,363	-0,446	0,012635947	uc.457+	uc.457+_P1	uc+	NA	lncRNA	NA	NA
41276	25	34	3	7	-1,364	-0,448	0,006338078	NM_001008898.1	CUST_2229_PI421866198	NM_001008898	NA	NA	NA	NA
1688	8	11	1	1	-1,365	-0,449	0,000964877	MRBC038268	MRBC038268_P1	MRBC038268	NA	lncRNA	NA	NA
22984	183	247	37	33	-1,365	-0,449	0,016198512	NM_001109648.1	CUST_772_PI421866198	NM_001109648	Tmem107	transmembrane protein 107	ENSRNOG00000006519	691750
3141	6	8	2	1	-1,365	-0,449	0,034337505	XR_006262	XR_006262_P1	XR_006262	NA	lncRNA	NA	NA
36848	22	31	4	9	-1,365	-0,449	0,039224074	NM_001029928.1	CUST_11121_PI421866198	NM_001029928	Lair1	leukocyte-associated immunoglobulin-like receptor 1	ENSRNOG00000027733	574531
1800	161	222	34	53	-1,366	-0,45	0,031274688	MRAK143591	MRAK143591_P1	MRAK143591	NA	lncRNA	NA	NA
20333	275	372	56	49	-1,367	-0,451	0,013424192	NM_001024333.1	CUST_540_PI421866198	NM_001024333	Cidec	cell death-inducing DFFA-like effector c	ENSRNOG00000009153	500292
1495	54	73	13	18	-1,367	-0,451	0,037379649	XR_006497	XR_006497_P1	XR_006497	NA	lncRNA	NA	NA
9265	46	64	6	13	-1,368	-0,452	0,006314923	NM_001000299.1	CUST_1121_PI421866198	NM_001000299	Olr469	olfactory receptor 469	ENSRNOG00000031426	295739
19730	817	1104	176	160	-1,368	-0,452	0,019506276	NM_001012056.1	CUST_2433_PI421866198	NM_001012056	Ces5a	carboxylesterase 5A	ENSRNOG00000025710	307660
41636	64	87	17	19	-1,368	-0,453	0,03720648	uc.299-	uc.299-_P1	uc-	NA	lncRNA	NA	NA
26563	12	16	3	4	-1,368	-0,453	0,04150962	NM_001109038.1	CUST_4613_PI421866198	NM_001109038	Fam222b	family with sequence similarity 222, member B	ENSRNOG00000029570	497960
37634	30	40	8	8	-1,369	-0,453	0,0343814	NM_001107263.1	CUST_9084_PI421866198	NM_001107263	Ngdn	neuroguidin	ENSRNOG00000018069	305887
16486	36	51	2	15	-1,37	-0,455	0,012129583	MRAK155851	MRAK155851_P1	MRAK155851	NA	lncRNA	NA	NA
28736	292	396	79	84	-1,37	-0,454	0,04543171	NM_001014182.1	CUST_10171_PI421866198	NM_001014182	Mall	mal, T-cell differentiation protein-like	ENSRNOG00000015599	362211
22577	42	57	4	6	-1,371	-0,455	0,000871134	NM_001000840.1	CUST_13043_PI421866198	NM_001000840	Olr1616	olfactory receptor 1616	ENSRNOG00000053022	405129
43670	54	73	13	11	-1,371	-0,456	0,018306469	NM_001009964.1	CUST_13931_PI421866198	NM_001009964	Tbx5	T-box 5	ENSRNOG00000001399	304514
40672	51	72	7	21	-1,372	-0,456	0,025008184	NM_001013230.1	CUST_13309_PI421866198	NM_001013230	Selplg	selectin P ligand	ENSRNOG00000000699	363930
22407	25	35	4	5	-1,373	-0,457	0,008009007	MRAK041418	MRAK041418_P1	MRAK041418	NA	lncRNA	NA	NA
34216	36	50	3	15	-1,373	-0,457	0,016514202	NM_001047901.1	CUST_279_PI421866198	NM_001047901	Ankle2	ankyrin repeat and LEM domain containing 2	ENSRNOG00000060144	360829
14099	3	4	1	1	-1,373	-0,457	0,029164478	MRAK040226	MRAK040226_P1	MRAK040226	NA	NA	NA	NA
44220	45	62	7	13	-1,374	-0,459	0,013316099	NM_001047092.1	CUST_5724_PI421866198	NM_001047092	Amz1	archaelysin family metallopeptidase 1	ENSRNOG00000024264	304317
5788	19	27	3	5	-1,374	-0,459	0,01369042	MRAK041468	MRAK041468_P1	MRAK041468	NA	lncRNA	NA	NA
25859	2	3	0	1	-1,374	-0,458	0,021328515	NM_173315.1	CUST_2530_PI421866198	NM_173315	Vom2r32	vomeronasal 2 receptor, 32	ENSRNOG00000002628	286981
20055	15	21	3	4	-1,374	-0,458	0,025436185	NM_153736.1	CUST_2549_PI421866198	NM_153736	Prl3a1	Prolactin family 3, subfamily a, member 1	ENSRNOG00000017103	NA
40241	52	73	10	20	-1,374	-0,458	0,034819786	NM_001109366.1	CUST_2625_PI421866198	NM_001109366	Cldn34a	claudin 34A	ENSRNOG00000024350	503435
20825	23	31	5	3	-1,375	-0,46	0,009553215	uc.323+	uc.323+_P1	uc+	NA	lncRNA	NA	NA
26268	2	3	1	1	-1,379	-0,464	0,014006413	uc.22+	uc.22+_P1	uc+	NA	NA	NA	NA
35911	31	43	3	5	-1,38	-0,465	0,000718364	NM_172093.1	CUST_7514_PI421866198	NM_172093	Hbg1	hemoglobin subunit gamma 1	ENSRNOG00000030879	94164
33292	319	447	56	127	-1,38	-0,465	0,021783616	NM_001004087.1	CUST_4989_PI421866198	NM_001004087	Pcdh7	protocadherin 7	ENSRNOG00000012367	360942
8101	14	20	3	5	-1,38	-0,465	0,035196491	XR_007567	XR_007567_P1	XR_007567	NA	lncRNA	NA	NA
38232	23	31	5	3	-1,381	-0,466	0,0097037	NM_001106682.1	CUST_9955_PI421866198	NM_001106682	Lao1	L-amino acid oxidase 1	ENSRNOG00000007232	298483
25870	92	129	14	31	-1,382	-0,466	0,015141427	MRAK021405	MRAK021405_P1	MRAK021405	NA	lncRNA	NA	NA
5925	10	13	2	3	-1,382	-0,467	0,023167193	Y09181	Y09181_P1	Y09181	NA	lncRNA	NA	NA
44425	3	5	1	1	-1,383	-0,467	0,020156569	NM_001009644.1	CUST_387_PI421866198	NM_001009644	Dbx1	developing brain homeobox 1	ENSRNOG00000014706	292934
39791	17	23	4	4	-1,384	-0,469	0,014719567	NM_138918.1	CUST_2996_PI421866198	NM_138918	Ss18l1	SS18L1, nBAF chromatin remodeling complex subunit	ENSRNOG00000060010	192352
10404	13	18	3	3	-1,384	-0,469	0,021088524	MRAK044925	MRAK044925_P1	MRAK044925	NA	lncRNA	NA	NA
35785	259	356	70	80	-1,386	-0,471	0,033660801	NM_001135173.1	CUST_5634_PI421866198	NM_001135173	Klk1c10	kallikrein 1-related peptidase C10	ENSRNOG00000046297	292858
9398	492	670	127	117	-1,387	-0,472	0,042800821	NM_012786.2	CUST_9469_PI421866198	NM_012786	Cox8b	cytochrome c oxidase, subunit VIIIb	ENSRNOG00000014656	25250
26601	490	681	124	171	-1,388	-0,473	0,040758964	NM_001141935.1	CUST_5041_PI421866198	NM_001141935	Atp10a	ATPase phospholipid transporting 10A (putative)	ENSRNOG00000056228	365266
13062	4742	6614	693	1150	-1,389	-0,474	0,005740023	NM_173142.1	CUST_3704_PI421866198	NM_173142	Stk16	serine/threonine kinase 16	ENSRNOG00000019294	286927
40586	140	202	12	71	-1,389	-0,474	0,025188712	NM_001106252.1	CUST_12871_PI421866198	NM_001106252	Klk11	kallikrein related-peptidase 11	ENSRNOG00000018742	292849
24865	10	14	2	2	-1,389	-0,474	0,028415633	NM_001102403.1	CUST_8962_PI421866198	NM_001102403	Svs6	seminal vesicle secretory protein 6	ENSRNOG00000026346	362267
16159	133	185	33	47	-1,389	-0,474	0,029053274	XR_009115	XR_009115_P1	XR_009115	NA	lncRNA	NA	NA
35445	223	313	47	87	-1,389	-0,474	0,029835353	NM_001000963.1	CUST_12358_PI421866198	NM_001000963	Olr1237	olfactory receptor 1237	ENSRNOG00000047869	100911939
21024	21	28	3	2	-1,39	-0,475	0,003230304	MRAK136747	MRAK136747_P1	MRAK136747	NA	lncRNA	NA	NA
9629	12	16	3	3	-1,39	-0,475	0,018385713	MRAK033665	MRAK033665_P1	MRAK033665	NA	lncRNA	NA	NA
14561	7	10	2	2	-1,39	-0,475	0,029490541	XR_006171	XR_006171_P1	XR_006171	NA	lncRNA	NA	NA
4735	3	4	1	1	-1,39	-0,476	0,044411866	MRAK082632	MRAK082632_P1	MRAK082632	NA	NA	NA	NA
41449	14	20	2	4	-1,391	-0,476	0,007459593	NM_001008909.1	CUST_1718_PI421866198	NM_001008909	NA	NA	NA	NA
32347	299	432	32	157	-1,391	-0,476	0,027426392	MRuc007otd	MRuc007otd_P1	MRuc007otd	NA	lncRNA	NA	NA
23815	42	61	3	23	-1,391	-0,476	0,032500166	MRAK002809	MRAK002809_P1	MRAK002809	NA	lncRNA	NA	NA
3765	384	530	113	130	-1,391	-0,476	0,038413776	XR_009359	XR_009359_P1	XR_009359	NA	lncRNA	NA	NA
1841	14	20	1	4	-1,392	-0,477	0,003823525	XR_006413	XR_006413_P1	XR_006413	NA	lncRNA	NA	NA
2802	57	78	14	13	-1,393	-0,479	0,035113976	XR_005565	XR_005565_P1	XR_005565	NA	lncRNA	NA	NA
13083	51	72	3	10	-1,394	-0,479	0,000509651	NM_001014089.1	CUST_10917_PI421866198	NM_001014089	Tbcel	tubulin folding cofactor E-like	ENSRNOG00000032364	315591
15963	9	13	2	2	-1,394	-0,479	0,009188874	MRAK089534	MRAK089534_P1	MRAK089534	NA	lncRNA	NA	NA
4350	47	66	8	12	-1,397	-0,482	0,006505434	NR_026689	NR_026689_P1	NR_026689	Abhd11os	lncRNA	NA	NA
35867	16	22	5	3	-1,398	-0,483	0,024655049	NM_001009532.1	CUST_939_PI421866198	NM_001009532	Taar3	trace amine-associated receptor 3	ENSRNOG00000025982	494319
34116	403	577	59	166	-1,398	-0,483	0,024947469	NM_001037556.2	CUST_11085_PI421866198	NM_001037556	Nmnat1	nicotinamide nucleotide adenylyltransferase 1	ENSRNOG00000015962	298653
11812	30	42	5	10	-1,399	-0,485	0,015885942	NM_130748.1	CUST_14913_PI421866198	NM_130748	Slc38a4	solute carrier family 38, member 4	ENSRNOG00000006653	170573
37030	90	130	16	46	-1,399	-0,484	0,036069224	AF267752	AF267752_P1	AF267752	NA	lncRNA	NA	NA
14985	46	66	11	20	-1,399	-0,484	0,039601976	MRAK144416	MRAK144416_P1	MRAK144416	NA	lncRNA	NA	NA
29964	23	32	4	11	-1,399	-0,485	0,039812711	NM_138825.2	CUST_3502_PI421866198	NM_138825	Serpina12	serpin family A member 12	ENSRNOG00000042634	191570
39839	10	13	3	2	-1,399	-0,484	0,044246223	NM_001106099.1	CUST_14165_PI421866198	NM_001106099	Cts7	cathepsin 7	ENSRNOG00000033540	290970
28763	22	31	5	5	-1,4	-0,485	0,021242803	MRAK043920	MRAK043920_P1	MRAK043920	NA	lncRNA	NA	NA
16672	7	10	2	3	-1,401	-0,487	0,045619536	XR_009279	XR_009279_P1	XR_009279	NA	lncRNA	NA	NA
14709	53	75	9	16	-1,402	-0,488	0,01102269	MRAK083456	MRAK083456_P1	MRAK083456	NA	lncRNA	NA	NA
39030	59	82	12	14	-1,402	-0,488	0,013470237	NM_001107531.1	CUST_7828_PI421866198	NM_001107531	Mesp1	mesoderm posterior bHLH transcription factor 1	ENSRNOG00000014951	308766
27131	2	3	0	0	-1,403	-0,489	0,001655803	NM_001107229.1	CUST_9162_PI421866198	NM_001107229	Depdc5	DEP domain containing 5	ENSRNOG00000018144	305464
10392	18	25	4	4	-1,403	-0,489	0,012811367	MRAK048827	MRAK048827_P1	MRAK048827	NA	lncRNA	NA	NA
26901	2	3	0	1	-1,403	-0,489	0,013152043	NM_012662.2	CUST_10280_PI421866198	NM_012662	Svs4	seminal vesicle secretory protein 4	ENSRNOG00000058182	103691965
38971	200	282	43	70	-1,403	-0,488	0,024911265	NM_024365.1	CUST_12387_PI421866198	NM_024365	Htr6	5-hydroxytryptamine receptor 6	ENSRNOG00000049761	64354
33353	20	27	6	2	-1,403	-0,488	0,035504002	X55180	X55180_P1	X55180	NA	lncRNA	NA	NA
15201	2	3	0	1	-1,405	-0,49	0,002283235	MRAK009488	MRAK009488_P1	MRAK009488	NA	NA	NA	NA
12370	18	25	6	5	-1,405	-0,49	0,047027305	AM293769	AM293769_P1	AM293769	NA	lncRNA	NA	NA
11028	2	3	0	1	-1,406	-0,492	0,007502768	MRuc009nia	MRuc009nia_P1	MRuc009nia	NA	NA	NA	NA
17562	276	374	87	46	-1,407	-0,493	0,039966913	uc.428+	uc.428+_P1	uc+	NA	lncRNA	NA	NA
39484	68	98	7	23	-1,408	-0,494	0,00782554	NM_001025276.1	CUST_13195_PI421866198	NM_001025276	RGD1305807	hypothetical LOC298077	ENSRNOG00000010497	298077
22425	68	95	15	20	-1,408	-0,494	0,013557196	MRAK015962	MRAK015962_P1	MRAK015962	NA	lncRNA	NA	NA
21645	65	93	9	29	-1,408	-0,493	0,018658483	MRAK037031	MRAK037031_P1	MRAK037031	NA	lncRNA	NA	NA
42997	40	55	13	12	-1,408	-0,494	0,036536955	NM_001108061.1	CUST_7648_PI421866198	NM_001108061	Amn	amnion associated transmembrane protein	ENSRNOG00000009050	314459
19526	10	15	2	4	-1,41	-0,496	0,031174771	NM_001000244.1	CUST_1267_PI421866198	NM_001000244	Olr321	olfactory receptor 321	ENSRNOG00000021065	293759
31691	18	25	6	5	-1,41	-0,496	0,047143886	NM_022935.2	CUST_160_PI421866198	NM_022935	Aoc1	amine oxidase, copper containing 1	ENSRNOG00000008575	65029
39883	75	104	21	14	-1,412	-0,498	0,019295328	NM_001107510.1	CUST_7862_PI421866198	NM_001107510	Acp4	NA	NA	NA
1175	8	11	1	1	-1,413	-0,499	0,000823976	MRuc007slx	MRuc007slx_P1	MRuc007slx	NA	lncRNA	NA	NA
18813	9	12	1	2	-1,413	-0,498	0,003128334	AY387050	AY387050_P1	AY387050	NA	lncRNA	NA	NA
44470	16	23	4	4	-1,414	-0,499	0,039193763	NM_017023.1	CUST_13499_PI421866198	NM_017023	Kcnj1	potassium voltage-gated channel subfamily J member 1	ENSRNOG00000059005	24521
41340	44	62	13	13	-1,414	-0,5	0,049509594	NM_001108277.1	CUST_6724_PI421866198	NM_001108277	Tom1l2	target of myb1 like 2 membrane trafficking protein	ENSRNOG00000003590	360537
11579	18	25	3	8	-1,415	-0,501	0,021851753	XR_006859	XR_006859_P1	XR_006859	NA	lncRNA	NA	NA
15977	2	3	0	1	-1,415	-0,501	0,046210192	BC166491	BC166491_P1	BC166491	NA	NA	NA	NA
9193	72	105	8	31	-1,416	-0,501	0,015483146	MRAK135767	MRAK135767_P1	MRAK135767	NA	lncRNA	NA	NA
36757	37	54	7	14	-1,417	-0,503	0,032044171	NM_001017483.1	CUST_14563_PI421866198	NM_001017483	LOC498063	similar to RIKEN cDNA 4930563D23	ENSRNOG00000001986	498063
33694	28	40	4	7	-1,419	-0,504	0,003670513	NM_017072.1	CUST_13308_PI421866198	NM_017072	Cps1	carbamoyl-phosphate synthase 1	ENSRNOG00000013704	497840
16970	2	3	0	1	-1,419	-0,505	0,012262569	XR_006233	XR_006233_P1	XR_006233	NA	NA	NA	NA
40648	233	330	47	69	-1,419	-0,505	0,017282969	NM_001106847.1	CUST_8891_PI421866198	NM_001106847	Tfdp2	transcription factor Dp-2	ENSRNOG00000011241	300947
35426	16	22	1	3	-1,42	-0,506	0,000551825	MRAK044945	MRAK044945_P1	MRAK044945	NA	lncRNA	NA	NA
22436	41	59	6	16	-1,42	-0,506	0,010886759	MRAK045928	MRAK045928_P1	MRAK045928	NA	lncRNA	NA	NA
12766	55	82	4	35	-1,42	-0,506	0,035117204	AF370447	AF370447_P1	AF370447	NA	lncRNA	NA	NA
23022	78	108	25	21	-1,42	-0,506	0,042711906	NM_001105962.2	CUST_11657_PI421866198	NM_001105962	Prg4	proteoglycan 4	ENSRNOG00000002385	289104
39742	11	15	3	1	-1,421	-0,507	0,014355201	NM_001106774.1	CUST_9293_PI421866198	NM_001106774	Nuak1	NUAK family kinase 1	ENSRNOG00000008061	299694
12303	70	98	21	28	-1,421	-0,507	0,042801536	MRAK014848	MRAK014848_P1	MRAK014848	NA	lncRNA	NA	NA
35569	51	72	6	13	-1,422	-0,508	0,002162185	uc.131-	uc.131-_P1	uc-	NA	lncRNA	NA	NA
13936	33	47	6	7	-1,423	-0,509	0,005308457	XR_005749	XR_005749_P1	XR_005749	NA	lncRNA	NA	NA
3298	12	16	2	3	-1,423	-0,509	0,00921567	MRAK087243	MRAK087243_P1	MRAK087243	NA	lncRNA	NA	NA
27757	2	3	0	1	-1,423	-0,509	0,017030766	NM_001001102.1	CUST_14273_PI421866198	NM_001001102	Olr1531	olfactory receptor 1531	ENSRNOG00000046676	NA
22471	13	19	2	3	-1,424	-0,51	0,003966387	MRAK133687	MRAK133687_P1	MRAK133687	NA	lncRNA	NA	NA
42466	4	6	1	2	-1,424	-0,51	0,047293373	NM_001000489.1	CUST_87_PI421866198	NM_001000489	Olr1765	olfactory receptor 1765	ENSRNOG00000030722	302821
35946	19	26	4	3	-1,425	-0,51	0,006361487	NM_001000539.1	CUST_15044_PI421866198	NM_001000539	Olr6	olfactory receptor 6	ENSRNOG00000045783	365175
39593	188	267	58	79	-1,425	-0,511	0,043348509	NM_001134547.1	CUST_6637_PI421866198	NM_001134547	RGD1304770	similar to Na+ dependent glucose transporter 1	ENSRNOG00000042918	309810
34486	84	119	15	23	-1,426	-0,512	0,006142227	MRuc007zkv	MRuc007zkv_P1	MRuc007zkv	NA	lncRNA	NA	NA
33737	141	198	40	40	-1,427	-0,513	0,019798945	MRAK041297	MRAK041297_P1	MRAK041297	NA	lncRNA	NA	NA
6035	78	112	18	27	-1,427	-0,513	0,021755293	XR_006126	XR_006126_P1	XR_006126	NA	lncRNA	NA	NA
26178	176	245	54	43	-1,427	-0,513	0,038099221	NM_013034.3	CUST_10819_PI421866198	NM_013034	Slc6a4	solute carrier family 6 member 4	ENSRNOG00000003476	25553
12575	13	18	2	4	-1,428	-0,514	0,008248585	AM293823	AM293823_P1	AM293823	NA	lncRNA	NA	NA
38073	29	41	8	2	-1,428	-0,514	0,010736257	NM_214831.1	CUST_4178_PI421866198	NM_214831	Olr1382	olfactory receptor 1382	ENSRNOG00000050905	287093
23640	217	324	31	124	-1,428	-0,514	0,033973357	MRAK143819	MRAK143819_P1	MRAK143819	NA	lncRNA	NA	NA
42536	74	109	10	34	-1,429	-0,515	0,012287221	NM_001009510.1	CUST_969_PI421866198	NM_001009510	NA	NA	NA	NA
7289	25	35	3	3	-1,43	-0,516	0,000488369	MRAK158353	MRAK158353_P1	MRAK158353	NA	lncRNA	NA	NA

24557	20	28	3	6	-1,43	-0,516	0,00715394	uc.324+	uc.324+_P1	uc+	NA	lncRNA	NA	NA
35334	13	19	2	3	-1,432	-0,518	0,004299163	NM_001008940.1	CUST_1591_PI421866198	NM_001008940	NA	NA	NA	NA
21129	17	25	4	5	-1,432	-0,518	0,010638675	NM_001000864.1	CUST_13019_PI421866198	NM_001000864	Olr1179	olfactory receptor 1179	ENSRNOG00000033150	405158
15512	131	191	22	55	-1,432	-0,518	0,017565922	MRAK076662	MRAK076662_P1	MRAK076662	NA	lncRNA	NA	NA
26260	12	17	2	3	-1,434	-0,52	0,005560949	NM_001000521.1	CUST_15063_PI421866198	NM_001000521	Olr1619	olfactory receptor 1619	ENSRNOG00000048856	361033
32077	18	27	2	8	-1,434	-0,521	0,018782188	NM_001014238.1	CUST_9498_PI421866198	NM_001014238	Poteg	POTE ankyrin domain family, member G	ENSRNOG00000012218	364620
37444	23	32	8	5	-1,434	-0,52	0,028055447	NM_001106956.1	CUST_8355_PI421866198	NM_001106956	Suv39h1	NA	NA	NA
38806	16	23	3	3	-1,435	-0,521	0,004518232	NM_001106892.1	CUST_8838_PI421866198	NM_001106892	Enpp4	ectonucleotide pyrophosphatase/phosphodiesterase 4	ENSRNOG00000010174	301261
9783	8	11	2	3	-1,435	-0,521	0,014379826	MRuc009hel	MRuc009hel_P1	MRuc009hel	NA	lncRNA	NA	NA
24727	20	29	3	6	-1,437	-0,523	0,003921695	MRAK019764	MRAK019764_P1	MRAK019764	NA	lncRNA	NA	NA
39337	11	16	3	2	-1,437	-0,523	0,015210747	NM_022291.1	CUST_4367_PI421866198	NM_022291	Npffr1	neuropeptide FF receptor 1	ENSRNOG00000000559	64107
39083	16	23	3	5	-1,437	-0,523	0,017972566	NM_080894.1	CUST_15148_PI421866198	NM_080894	Pde7b	phosphodiesterase 7B	ENSRNOG00000013436	140929
30332	9	13	2	2	-1,437	-0,523	0,021475696	MRAK037283	MRAK037283_P1	MRAK037283	NA	lncRNA	NA	NA
23005	41	61	8	18	-1,438	-0,524	0,01756919	MRuc009tdb	MRuc009tdb_P1	MRuc009tdb	NA	lncRNA	NA	NA
40331	14	20	3	5	-1,438	-0,524	0,029710057	NM_001107636.1	CUST_7458_PI421866198	NM_001107636	Adamts14	ADAM metallopeptidase with thrombospondin type 1 motif, 14	ENSRNOG00000000563	309837
20452	13	19	2	4	-1,439	-0,525	0,006453884	AY325213	AY325213_P1	AY325213	NA	lncRNA	NA	NA
41510	268	383	65	90	-1,439	-0,525	0,022382624	NM_001107763.2	CUST_6937_PI421866198	NM_001107763	Ltk	leukocyte receptor tyrosine kinase	ENSRNOG00000025130	311337
30244	154	221	40	54	-1,439	-0,525	0,027343163	NM_001106634.1	CUST_10076_PI421866198	NM_001106634	Sdr16c5	short chain dehydrogenase/reductase family 16C, member 5	ENSRNOG00000008871	297805
9032	12	18	3	2	-1,44	-0,526	0,0056192	XR_008107	XR_008107_P1	XR_008107	NA	lncRNA	NA	NA
34782	33	47	8	7	-1,444	-0,53	0,027915907	MRAK142801	MRAK142801_P1	MRAK142801	NA	lncRNA	NA	NA
8944	267	370	90	52	-1,444	-0,53	0,039507116	BC084694	BC084694_P1	BC084694	NA	lncRNA	NA	NA
29456	46	68	8	20	-1,445	-0,531	0,021767026	NM_001107653.1	CUST_7434_PI421866198	NM_001107653	Slc45a2	solute carrier family 45, member 2	ENSRNOG00000018759	310152
10386	6	9	2	2	-1,445	-0,531	0,043534044	MRLIT3360	MRLIT3360_P1	MRLIT3360	NA	lncRNA	NA	NA
32678	27	38	4	3	-1,446	-0,532	0,001087845	NM_001106803.1	CUST_8935_PI421866198	NM_001106803	Mbd3l1	methyl-CpG binding domain protein 3-like 1	ENSRNOG00000006363	300389
24475	33	48	6	12	-1,446	-0,532	0,014221998	XR_008678	XR_008678_P1	XR_008678	NA	lncRNA	NA	NA
34864	3	5	1	1	-1,446	-0,532	0,020295047	AY177417	AY177417_P1	AY177417	NA	NA	NA	NA
38542	132	198	21	76	-1,446	-0,532	0,026858394	NM_001099499.1	CUST_13370_PI421866198	NM_001099499	Vom2r63	NA	NA	NA
38552	71	107	9	35	-1,447	-0,533	0,024136388	NM_031600.1	CUST_11368_PI421866198	NM_031600	Ptprn2	protein tyrosine phosphatase, receptor type N2	ENSRNOG00000005003	29714
34537	11	16	2	4	-1,449	-0,535	0,019615672	NM_001108500.1	CUST_4956_PI421866198	NM_001108500	Pik3c2a	phosphatidylinositol-4-phosphate 3-kinase, catalytic subunit type 2 alpha	ENSRNOG00000020479	361632
37111	12	17	2	2	-1,45	-0,536	0,0017861	NM_001001285.1	CUST_13248_PI421866198	NM_001001285	Olr131	olfactory receptor 131	ENSRNOG00000048465	405071
20479	35	49	8	3	-1,45	-0,536	0,003809303	MRAK051970	MRAK051970_P1	MRAK051970	NA	lncRNA	NA	NA
36367	72	101	24	21	-1,45	-0,536	0,031228241	NM_057109.1	CUST_7945_PI421866198	NM_057109	Barhl1	BarH-like homeobox 1	ENSRNOG00000013209	117232
24965	23	35	3	9	-1,451	-0,537	0,01053582	NM_001109389.1	CUST_2508_PI421866198	NM_001109389	LOC680045	hypothetical protein LOC680045	ENSRNOG00000047468	680045
33545	68	99	16	28	-1,451	-0,537	0,022775388	MRAK082368	MRAK082368_P1	MRAK082368	NA	lncRNA	NA	NA
36271	15	22	3	6	-1,453	-0,539	0,021938865	NM_001107105.1	CUST_9823_PI421866198	NM_001107105	Zbtb21	NA	NA	NA
18185	51	72	16	10	-1,456	-0,542	0,013887749	NR_003527	NR_003527_P1	NR_003527	Dzip1-ps1	lncRNA	ENSRNOG00000046611	NA
23898	2	3	0	1	-1,456	-0,542	0,015060507	MRAK041702	MRAK041702_P1	MRAK041702	NA	NA	NA	NA
35144	26	39	4	15	-1,456	-0,542	0,021672804	XR_006950	XR_006950_P1	XR_006950	NA	lncRNA	NA	NA
24478	216	305	67	50	-1,456	-0,542	0,026514755	NM_019280.1	CUST_5899_PI421866198	NM_019280	Gja5	gap junction protein, alpha 5	ENSRNOG00000017484	50563
19100	19	28	3	7	-1,457	-0,543	0,008078061	MRAK008425	MRAK008425_P1	MRAK008425	NA	lncRNA	NA	NA
40601	7	9	2	1	-1,457	-0,543	0,029909161	NM_001000654.1	CUST_14471_PI421866198	NM_001000654	Olr613	olfactory receptor 613	ENSRNOG00000045685	404852
41345	67	100	5	27	-1,458	-0,544	0,00547944	NM_022263.2	CUST_4432_PI421866198	NM_022263	Cabs1	calcium binding protein, spermatid associated 1	ENSRNOG00000001950	64029
11890	105	155	28	47	-1,458	-0,544	0,042700541	MRAK044010	MRAK044010_P1	MRAK044010	NA	lncRNA	NA	NA
12270	19	28	3	3	-1,459	-0,545	0,00224691	MRuc007srl	MRuc007srl_P1	MRuc007srl	NA	lncRNA	NA	NA
38027	119	174	20	36	-1,459	-0,545	0,004562233	NM_001011698.1	CUST_2065_PI421866198	NM_001011698	Spetex-2C	NA	NA	NA
1353	23	34	8	8	-1,459	-0,545	0,033098442	AF514285	AF514285_P1	AF514285	NA	lncRNA	NA	NA
15520	41	60	6	13	-1,46	-0,546	0,00365177	XR_008909	XR_008909_P1	XR_008909	NA	lncRNA	NA	NA
31769	19	29	4	7	-1,46	-0,546	0,014064699	BC160898	BC160898_P1	BC160898	NA	lncRNA	NA	NA
41296	73	104	22	19	-1,46	-0,546	0,016040995	NM_001029911.1	CUST_11157_PI421866198	NM_001029911	Cit	citron rho-interacting serine/threonine kinase	ENSRNOG00000001143	83620
37192	15	22	4	3	-1,46	-0,546	0,016236746	uc.250-	uc.250-_P1	uc-	NA	lncRNA	NA	NA
14686	4	6	1	2	-1,46	-0,546	0,022424321	XR_006711	XR_006711_P1	XR_006711	NA	NA	NA	NA
2396	5	6	2	1	-1,461	-0,547	0,029522914	MRAK041283	MRAK041283_P1	MRAK041283	NA	NA	NA	NA
14987	5	7	1	2	-1,462	-0,548	0,019868522	MRAK040911	MRAK040911_P1	MRAK040911	NA	NA	NA	NA
3327	9	13	3	3	-1,463	-0,549	0,04780131	MRAK140517	MRAK140517_P1	MRAK140517	NA	lncRNA	NA	NA
31435	7	10	1	1	-1,464	-0,55	0,002504886	Z93369	Z93369_P1	Z93369	NA	lncRNA	NA	NA
11291	11	15	3	2	-1,464	-0,55	0,017198131	NM_001004271.1	CUST_3660_PI421866198	NM_001004271	Ugt2b35	UDP glucuronosyltransferase 2 family, polypeptide B35	ENSRNOG00000001980	83808
23965	18	26	3	3	-1,465	-0,551	0,001648516	uc.67-	uc.67-_P1	uc-	NA	lncRNA	NA	NA
7049	18	27	3	4	-1,465	-0,551	0,002328548	MRAK046551	MRAK046551_P1	MRAK046551	NA	lncRNA	NA	NA
37241	84	123	16	21	-1,465	-0,551	0,008082331	NM_017329.1	CUST_11369_PI421866198	NM_017329	Sftpa1	surfactant protein A1	ENSRNOG00000011438	24773
13089	254	361	77	75	-1,465	-0,551	0,041026868	NM_001106573.1	CUST_10747_PI421866198	NM_001106573	Crygn	crystallin, gamma N	ENSRNOG00000009226	296730
37552	74	106	23	14	-1,466	-0,552	0,012580902	NM_001012117.1	CUST_1879_PI421866198	NM_001012117	Fbxo43	F-box protein 43	ENSRNOG00000010459	315034
10622	4	6	1	1	-1,466	-0,552	0,01890789	MRAK030009	MRAK030009_P1	MRAK030009	NA	NA	NA	NA
25928	3	5	1	1	-1,467	-0,553	0,016561995	M34730	M34730_P1	M34730	NA	NA	NA	NA
25092	18	26	4	3	-1,468	-0,554	0,003828592	MRuc009kuc	MRuc009kuc_P1	MRuc009kuc	NA	lncRNA	NA	NA
41079	82	122	22	43	-1,47	-0,556	0,036486595	DQ223059	DQ223059_P1	DQ223059	NA	lncRNA	NA	NA
31234	19	27	6	5	-1,472	-0,558	0,037974468	NM_001000405.1	CUST_269_PI421866198	NM_001000405	Olr841	olfactory receptor 841	ENSRNOG00000049788	298049
9250	106	162	12	57	-1,473	-0,559	0,018107814	XR_006791	XR_006791_P1	XR_006791	NA	lncRNA	NA	NA
1306	1047	1544	166	235	-1,474	-0,56	0,002074263	NM_001107074.1	CUST_10409_PI421866198	NM_001107074	Cd7	Cd7 molecule	ENSRNOG00000036674	303747
18605	3	5	1	1	-1,477	-0,563	0,015125459	MRuc008ysg	MRuc008ysg_P1	MRuc008ysg	NA	NA	NA	NA
25973	10	14	3	2	-1,477	-0,563	0,025352264	NM_001008868.1	CUST_2281_PI421866198	NM_001008868	Wfdc9	WAP four-disulfide core domain 9	ENSRNOG00000031380	408228
37390	14	21	2	3	-1,478	-0,564	0,002908053	uc.155+	uc.155+_P1	uc+	NA	lncRNA	NA	NA
25784	28	40	7	7	-1,478	-0,564	0,00878346	MRAK043901	MRAK043901_P1	MRAK043901	NA	lncRNA	NA	NA
18008	459	656	155	136	-1,478	-0,564	0,042973609	NM_001105967.2	CUST_11648_PI421866198	NM_001105967	Lmx1a	LIM homeobox transcription factor 1 alpha	ENSRNOG00000004642	289201
37029	42	63	5	17	-1,479	-0,565	0,007038782	NM_001109316.1	CUST_2803_PI421866198	NM_001109316	Rai2	retinoic acid induced 2	ENSRNOG00000006670	501555
14434	8	13	2	4	-1,479	-0,565	0,02969359	Y09183	Y09183_P1	Y09183	NA	lncRNA	NA	NA
11690	2	3	0	1	-1,479	-0,565	0,031418765	XR_008558	XR_008558_P1	XR_008558	NA	NA	NA	NA
17619	9	13	3	3	-1,48	-0,566	0,034077169	AF050660	AF050660_P1	AF050660	NA	lncRNA	NA	NA
25177	33	49	4	10	-1,482	-0,567	0,002095727	MRAK031283	MRAK031283_P1	MRAK031283	NA	lncRNA	NA	NA
41125	14	20	5	3	-1,482	-0,568	0,017823999	NM_013182.2	CUST_9932_PI421866198	NM_013182	Mc5r	melanocortin 5 receptor	ENSRNOG00000016685	25726
39553	37	53	13	9	-1,482	-0,567	0,032422027	uc.359-	uc.359-_P1	uc-	NA	lncRNA	NA	NA
22269	27	41	3	13	-1,483	-0,568	0,007737245	NM_001008866.1	CUST_2285_PI421866198	NM_001008866	Wfdc12	WAP four-disulfide core domain 12	ENSRNOG00000032412	408227
34371	3	4	1	2	-1,483	-0,568	0,044269237	uc.389-	uc.389-_P1	uc-	NA	NA	NA	NA
19854	2	3	0	1	-1,484	-0,569	0,013922498	uc.148+	uc.148+_P1	uc+	NA	NA	NA	NA
18104	47	66	17	10	-1,484	-0,569	0,029227803	AY169318	AY169318_P1	AY169318	NA	lncRNA	NA	NA
20463	28	42	5	10	-1,485	-0,571	0,010922721	NM_001109158.1	CUST_3859_PI421866198	NM_001109158	Fam24a	family with sequence similarity 24, member A	ENSRNOG00000026035	499278
13201	2	3	0	2	-1,488	-0,573	0,038631903	NM_001009663.1	CUST_349_PI421866198	NM_001009663	Serpina6	serpin family A member 6	ENSRNOG00000009438	299270
30456	67	95	20	13	-1,489	-0,574	0,036669541	AF329638	AF329638_P1	AF329638	NA	lncRNA	NA	NA
37646	6	10	1	2	-1,492	-0,577	0,009427033	NM_001099462.1	CUST_13516_PI421866198	NM_001099462	Vom2r5	vomeronasal 2 receptor, 5	ENSRNOG00000032365	679691
29442	666	1007	172	297	-1,493	-0,579	0,022823628	NM_212466.3	CUST_12718_PI421866198	NM_212466	Cfb	complement factor B	ENSRNOG00000000419	294257
36722	169	269	27	118	-1,494	-0,579	0,040990699	NM_001082410.1	CUST_14783_PI421866198	NM_001082410	Rnf144a	ring finger protein 144A	ENSRNOG00000007370	500636
40172	36	55	5	10	-1,495	-0,58	0,001844394	NM_001008932.1	CUST_1628_PI421866198	NM_001008932	NA	NA	NA	NA
32500	580	881	155	335	-1,495	-0,58	0,041874527	NM_001107193.1	CUST_9596_PI421866198	NM_001107193	Tbx19	T-box 19	ENSRNOG00000002979	304935
43019	115	172	22	33	-1,496	-0,581	0,005967783	NM_001025038.1	CUST_14665_PI421866198	NM_001025038	Mettl24	methyltransferase like 24	ENSRNOG00000024221	499465
13882	16	23	4	2	-1,497	-0,582	0,002754349	MRAK141018	MRAK141018_P1	MRAK141018	NA	lncRNA	NA	NA
24275	11	16	4	3	-1,498	-0,583	0,019088053	NM_001008878.1	CUST_2269_PI421866198	NM_001008878	LOC362921	Ac2-224	ENSRNOG00000033584	362921
7783	105	153	35	27	-1,499	-0,584	0,015457439	BC166515	BC166515_P1	BC166515	NA	lncRNA	NA	NA
24469	73	106	27	26	-1,499	-0,584	0,038307146	U03628	U03628_P1	U03628	NA	lncRNA	NA	NA
3406	4	6	2	1	-1,5	-0,585	0,038818281	S83270	S83270_P1	S83270	NA	NA	NA	NA
40894	6	9	2	2	-1,5	-0,585	0,042196121	NM_001000018.1	CUST_2761_PI421866198	NM_001000018	Olr1443	olfactory receptor 1443	ENSRNOG00000046777	287331
19792	48	72	12	20	-1,501	-0,586	0,019842822	MRAK082556	MRAK082556_P1	MRAK082556	NA	lncRNA	NA	NA
40250	16	25	2	8	-1,502	-0,587	0,023174105	NM_001008958.1	CUST_1566_PI421866198	NM_001008958	NA	NA	NA	NA
45155	4	6	1	2	-1,502	-0,587	0,039353875	uc.1-	uc.1-_P1	uc-	NA	NA	NA	NA
19291	2	3	0	1	-1,503	-0,588	0,011643567	MRuc008yjh	MRuc008yjh_P1	MRuc008yjh	NA	NA	NA	NA
5930	158	234	46	61	-1,503	-0,588	0,032571151	XR_006482	XR_006482_P1	XR_006482	NA	lncRNA	NA	NA
6975	229	337	90	103	-1,503	-0,588	0,049436509	XR_009017	XR_009017_P1	XR_009017	NA	lncRNA	NA	NA
24507	2	3	0	1	-1,504	-0,589	0,003020648	NM_001000893.1	CUST_12946_PI421866198	NM_001000893	Olr1678	NA	NA	NA
23670	10	14	3	2	-1,504	-0,589	0,012450637	MRAK047008	MRAK047008_P1	MRAK047008	NA	lncRNA	NA	NA
41560	163	235	65	45	-1,504	-0,589	0,035062829	uc.47+	uc.47+_P1	uc+	NA	lncRNA	NA	NA
15652	55	82	9	14	-1,506	-0,591	0,002084881	X80519	X80519_P1	X80519	NA	lncRNA	NA	NA
5042	7	11	2	4	-1,507	-0,591	0,029630059	MRAK155091	MRAK155091_P1	MRAK155091	NA	lncRNA	NA	NA
4355	199	293	82	85	-1,507	-0,592	0,044607112	MRAK047367	MRAK047367_P1	MRAK047367	NA	lncRNA	NA	NA
35290	4	6	1	2	-1,508	-0,593	0,018531127	MRAK020435	MRAK020435_P1	MRAK020435	NA	NA	NA	NA
38679	11	17	3	6	-1,508	-0,593	0,043671161	NM_001007670.1	CUST_7087_PI421866198	NM_001007670	Odf4	outer dense fiber of sperm tails 4	ENSRNOG00000004225	303236
13493	10	16	2	2	-1,509	-0,594	0,001363754	MRAK042490	MRAK042490_P1	MRAK042490	NA	lncRNA	NA	NA
12940	2	4	0	1	-1,509	-0,593	0,007839084	MRAK163405	MRAK163405_P1	MRAK163405	NA	NA	NA	NA
27676	9	13	1	4	-1,509	-0,594	0,01001782	NM_001037530.1	CUST_11133_PI421866198	NM_001037530	Defb18	defensin beta 18	ENSRNOG00000039650	641655
21641	8	11	2	1	-1,509	-0,594	0,026041289	MRAK053676	MRAK053676_P1	MRAK053676	NA	lncRNA	NA	NA
32022	21	33	2	10	-1,511	-0,595	0,019773336	MRAK148575	MRAK148575_P1	MRAK148575	NA	lncRNA	NA	NA
10810	2	4	1	1	-1,511	-0,595	0,042301729	XR_005500	XR_005500_P1	XR_005500	NA	NA	NA	NA
44911	7	10	1	3	-1,513	-0,597	0,005293659	NM_001001114.1	CUST_14237_PI421866198	NM_001001114	Olr1701	NA	NA	NA
4076	4	5	1	1	-1,513	-0,598	0,015824191	MRAK079765	MRAK079765_P1	MRAK079765	NA	NA	NA	NA
11211	2	4	0	1	-1,514	-0,598	0,001734411	MRAK015431	MRAK015431_P1	MRAK015431	NA	NA	NA	NA
7500	78	120	13	29	-1,514	-0,598	0,007417585	MRAK157089	MRAK157089_P1	MRAK157089	NA	lncRNA	NA	NA
23971	2	4	1	1	-1,514	-0,598	0,014161143	uc.289+	uc.289+_P1	uc+	NA	NA	NA	NA
38745	12	18	2	3	-1,515	-0,599	0,001250959	uc.134-	uc.134-_P1	uc-	NA	lncRNA	NA	NA
40087	14	21	2	5	-1,516	-0,6	0,003918881	NM_001113788.1	CUST_10261_PI421866198	NM_001113788	RGD1562024	RGD1562024	ENSRNOG00000042749	498699
15208	143	220	35	75	-1,516	-0,6	0,02593059	MRAK028093	MRAK028093_P1	MRAK028093	NA	lncRNA	NA	NA
17393	3	4	1	1	-1,516	-0,6	0,029844692	Z93363	Z93363_P1	Z93363	NA	NA	NA	NA
36537	15	21	5	4	-1,516	-0,601	0,038121362	NM_022288.1	CUST_4375_PI421866198	NM_022288	Csnk1g1	casein kinase 1, gamma 1	ENSRNOG00000016620	64086
13169	76	115	31	41	-1,516	-0,6	0,046461373	MRuc008uyy	MRuc008uyy_P1	MRuc008uyy	NA	lncRNA	NA	NA
16834	2	3	0	1	-1,517	-0,602	0,013126204	uc.124+	uc.124+_P1	uc+	NA	NA	NA	NA
35298	64	100	14	35	-1,517	-0,601	0,024759605	AJ286232	AJ286232_P1	AJ286232	NA	lncRNA	NA	NA
843	3	4	1	1	-1,521	-0,605	0,004498275	MRAK081022	MRAK081022_P1	MRAK081022	NA	NA	NA	NA
33663	83	131	16	53	-1,521	-0,605	0,024904755	MRAK040720	MRAK040720_P1	MRAK040720	NA	lncRNA	NA	NA
45036	83	122	31	17	-1,522	-0,606	0,017828552	NM_201561.1	CUST_8645_PI421866198	NM_201561	Scgn	secretagogin, EF-hand calcium binding protein	ENSRNOG00000016628	306942
3042	4	6	1	1	-1,525	-0,609	0,048012199	MRAK149866	MRAK149866_P1	MRAK149866	NA	NA	NA	NA
3255	19	30	3	11	-1,526	-0,61	0,028174759	MRAK015708	MRAK015708_P1	MRAK015708	NA	lncRNA	NA	NA
37632	57	86	23	25	-1,528	-0,612	0,034928157	NM_001008962.1	CUST_1561_PI421866198	NM_001008962	NA	NA	NA	NA
19373	7	11	2	3	-1,528	-0,612	0,036523396	XR_005523	XR_005523_P1	XR_005523	NA	lncRNA	NA	NA
39072	30	46	7	10	-1,529	-0,613	0,006084263	uc.430-	uc.430-_P1	uc-	NA	lncRNA	NA	NA
11035	9	13	2	3	-1,53	-0,613	0,002379372	NM_001105956.1	CUST_11669_PI421866198	NM_001105956	F13b	coagulation factor XIII B chain	ENSRNOG00000012613	NA
11994	13	19	3	3	-1,53	-0,614	0,004637571	U06749	U06749_P1	U06749	NA	lncRNA	NA	NA
39567	32	47	9	8	-1,531	-0,615	0,010336202	NM_017013.4	CUST_13546_PI421866198	NM_017013	Gsta2	glutathione S-transferase alpha 2	ENSRNOG00000029861	NA
13253	4	6	1	2	-1,534	-0,617	0,035621519	NM_080908.1	CUST_14887_PI421866198	NM_080908	Slc28a3	solute carrier family 28 member 3	ENSRNOG00000018853	140944
5625	65	97	21	21	-1,535	-0,618	0,015810245	X80526	X80526_P1	X80526	NA	lncRNA	NA	NA
33595	434	663	148	213	-1,535	-0,618	0,041247638	MRAK043711	MRAK043711_P1	MRAK043711	NA	lncRNA	NA	NA
23843	14	20	4	3	-1,536	-0,619	0,003082577	NM_001008908.1	CUST_1719_PI421866198	NM_001008908	NA	NA	NA	NA
30881	2	4	0	2	-1,537	-0,621	0,016247461	NM_001008926.1	CUST_1653_PI421866198	NM_001008926	NA	NA	NA	NA
24984	18	29	3	11	-1,537	-0,62	0,017280794	NM_022257.1	CUST_4448_PI421866198	NM_022257	Masp1	mannan-binding lectin serine peptidase 1	ENSRNOG00000001827	64023
18011	4	7	1	1	-1,538	-0,621	0,007698906	MRAK157048	MRAK157048_P1	MRAK157048	NA	NA	NA	NA
42624	207	331	34	110	-1,538	-0,621	0,015001011	NM_001014251.1	CUST_9454_PI421866198	NM_001014251	LOC365778	similar to RIKEN cDNA 1700034I23	ENSRNOG00000061390	365778
38555	198	320	25	120	-1,538	-0,621	0,016114699	NM_001106405.1	CUST_11500_PI421866198	NM_001106405	Cd180	CD180 molecule	ENSRNOG00000010266	294706
20208	9	15	3	7	-1,542	-0,625	0,04876756	XR_006220	XR_006220_P1	XR_006220	NA	lncRNA	NA	NA
14452	4	6	1	2	-1,543	-0,626	0,040310814	MRuc008jtw	MRuc008jtw_P1	MRuc008jtw	NA	NA	NA	NA
41935	25	38	7	7	-1,547	-0,63	0,023562782	NM_001000751.1	CUST_13796_PI421866198	NM_001000751	Olr1351	olfactory receptor 1351	ENSRNOG00000032545	405021
24311	7	11	3	1	-1,547	-0,629	0,040515704	NM_001106302.1	CUST_12309_PI421866198	NM_001106302	Hmx3	H6 family homeobox 3	ENSRNOG00000020637	293537
24136	115	188	5	73	-1,548	-0,631	0,012642262	MRAK047415	MRAK047415_P1	MRAK047415	NA	lncRNA	NA	NA
40079	7	11	2	4	-1,548	-0,631	0,03575429	NM_001000330.1	CUST_769_PI421866198	NM_001000330	Olr592	olfactory receptor 592	ENSRNOG00000049572	295824
30623	3	5	1	1	-1,549	-0,631	0,011892107	uc.321-	uc.321-_P1	uc-	NA	NA	NA	NA
1074	65	109	5	52	-1,549	-0,631	0,028651984	AM981047	AM981047_P1	AM981047	NA	lncRNA	NA	NA
44083	91	153	15	77	-1,549	-0,631	0,038437717	NM_001024986.1	CUST_12094_PI421866198	NM_001024986	RGD1306186	similar to RIKEN cDNA 4930569K13	ENSRNOG00000037039	NA
25050	312	500	82	223	-1,549	-0,632	0,042131606	MRAK163632	MRAK163632_P1	MRAK163632	NA	lncRNA	NA	NA
5078	2	4	0	2	-1,549	-0,631	0,043267249	uc.277-	uc.277-_P1	uc-	NA	NA	NA	NA
13147	48	73	23	26	-1,55	-0,632	0,04938364	MRAK048166	MRAK048166_P1	MRAK048166	NA	lncRNA	NA	NA
36966	29	44	12	13	-1,551	-0,633	0,032517427	NM_001008945.1	CUST_1585_PI421866198	NM_001008945	NA	NA	NA	NA
3722	10	16	2	3	-1,552	-0,635	0,004895748	MRAK036070	MRAK036070_P1	MRAK036070	NA	lncRNA	NA	NA
10377	13	22	2	8	-1,552	-0,634	0,02168227	XR_006202	XR_006202_P1	XR_006202	NA	lncRNA	NA	NA
13346	15	23	4	2	-1,553	-0,635	0,00310572	AB005146	AB005146_P1	AB005146	NA	lncRNA	NA	NA
34877	3	5	1	1	-1,553	-0,635	0,013742858	NM_001109652.1	CUST_750_PI421866198	NM_001109652	Terb2	telomere repeat binding bouquet formation protein 2	ENSRNOG00000037178	691952
36724	10	14	3	2	-1,553	-0,635	0,045328776	NM_001126082.1	CUST_11283_PI421866198	NM_001126082	Anapc11	anaphase promoting complex subunit 11	ENSRNOG00000036686	498030
14130	5	8	2	1	-1,554	-0,636	0,009404594	MRAK078828	MRAK078828_P1	MRAK078828	NA	NA	NA	NA
26607	3	6	1	2	-1,554	-0,636	0,022067438	NM_032070.1	CUST_11533_PI421866198	NM_032070	Hmga2	high mobility group AT-hook 2	ENSRNOG00000042460	84017
21050	7	12	1	3	-1,555	-0,637	0,002844999	XR_007830	XR_007830_P1	XR_007830	NA	lncRNA	NA	NA
17412	29	46	8	14	-1,555	-0,637	0,012830005	AY539950	AY539950_P1	AY539950	NA	lncRNA	NA	NA
9115	14	21	4	6	-1,556	-0,638	0,038444803	MRAK016141	MRAK016141_P1	MRAK016141	NA	lncRNA	NA	NA
7374	2	4	1	2	-1,556	-0,638	0,044528825	MRAK163282	MRAK163282_P1	MRAK163282	NA	NA	NA	NA
10808	17	27	4	5	-1,557	-0,639	0,003834891	MRAK047202	MRAK047202_P1	MRAK047202	NA	lncRNA	NA	NA
24990	15	23	2	3	-1,558	-0,64	0,000503229	NM_001137562.1	CUST_11515_PI421866198	NM_001137562	Urm1	ubiquitin related modifier 1	ENSRNOG00000026636	311840
39100	163	261	29	90	-1,558	-0,64	0,008179939	NM_001007638.1	CUST_7150_PI421866198	NM_001007638	RGD1359334	similar to hypothetical protein FLJ20519	ENSRNOG00000021656	295265
40119	10	14	3	1	-1,558	-0,639	0,012132071	NM_001001040.1	CUST_14818_PI421866198	NM_001001040	Olr313	olfactory receptor 313	ENSRNOG00000029226	405926
11694	7	11	1	3	-1,559	-0,64	0,000952656	XR_008333	XR_008333_P1	XR_008333	NA	lncRNA	NA	NA
23374	23	37	2	10	-1,559	-0,64	0,002167648	XR_006304	XR_006304_P1	XR_006304	NA	lncRNA	NA	NA
29126	42	69	5	23	-1,559	-0,641	0,012384156	XR_009350	XR_009350_P1	XR_009350	NA	lncRNA	NA	NA
5833	3	4	1	1	-1,559	-0,641	0,038065802	XR_006298	XR_006298_P1	XR_006298	NA	NA	NA	NA
36964	17	27	1	6	-1,561	-0,643	0,000298735	uc.135+	uc.135+_P1	uc+	NA	lncRNA	NA	NA
6789	118	186	18	36	-1,561	-0,643	0,001074146	MRAK048306	MRAK048306_P1	MRAK048306	NA	lncRNA	NA	NA
13675	3	4	1	1	-1,561	-0,643	0,009436639	MRAK037363	MRAK037363_P1	MRAK037363	NA	NA	NA	NA
28472	67	111	9	40	-1,561	-0,643	0,022228198	U89745	U89745_P1	U89745	NA	lncRNA	NA	NA
14990	139	217	54	89	-1,561	-0,643	0,037911809	MRAK084573	MRAK084573_P1	MRAK084573	NA	lncRNA	NA	NA
10413	8	12	4	2	-1,562	-0,644	0,030443047	MRAK143708	MRAK143708_P1	MRAK143708	NA	lncRNA	NA	NA
9748	3	4	1	1	-1,563	-0,644	0,006087262	MRAK033137	MRAK033137_P1	MRAK033137	NA	NA	NA	NA
12991	14	22	4	4	-1,564	-0,645	0,002443845	uc.286+	uc.286+_P1	uc+	NA	lncRNA	NA	NA
2986	21	32	5	6	-1,564	-0,645	0,004408478	MRAK149038	MRAK149038_P1	MRAK149038	NA	lncRNA	NA	NA
16604	2	4	0	2	-1,565	-0,646	0,031883516	uc.149+	uc.149+_P1	uc+	NA	NA	NA	NA
42581	141	215	63	67	-1,566	-0,647	0,037491303	uc.293+	uc.293+_P1	uc+	NA	lncRNA	NA	NA
35055	39	63	7	17	-1,567	-0,648	0,007586038	NM_001108370.1	CUST_6054_PI421866198	NM_001108370	NA	NA	NA	NA
32632	6	9	1	3	-1,567	-0,648	0,03652509	BC082047	BC082047_P1	BC082047	NA	lncRNA	NA	NA
9165	3	4	1	1	-1,568	-0,649	0,007284112	NM_001146004.1	CUST_8888_PI421866198	NM_001146004	Txndc2	thioredoxin domain containing 2	ENSRNOG00000027916	316777
15040	7	12	2	2	-1,569	-0,65	0,004595354	MRAK078262	MRAK078262_P1	MRAK078262	NA	NA	NA	NA
41051	20	29	10	4	-1,571	-0,652	0,033459532	NM_001011960.1	CUST_364_PI421866198	NM_001011960	Zswim2	zinc finger, SWIM-type containing 2	ENSRNOG00000032579	296455
20069	3	4	0	2	-1,572	-0,652	0,042224262	AB097854	AB097854_P1	AB097854	NA	NA	NA	NA
451	2	4	0	2	-1,573	-0,653	0,02943495	CUST_31_PI415351601	CUST_31_PI415351601	CUST_31_PI415351601	NA	NA	NA	NA
14417	3	5	1	2	-1,574	-0,655	0,014603895	XR_009149	XR_009149_P1	XR_009149	NA	NA	NA	NA
36524	224	358	25	84	-1,575	-0,655	0,001134812	NM_001108115.1	CUST_7325_PI421866198	NM_001108115	Atf7	activating transcription factor 7	ENSRNOG00000015269	315333
42006	99	159	27	58	-1,575	-0,655	0,021220476	NM_001108973.1	CUST_2157_PI421866198	NM_001108973	Slc1a7	solute carrier family 1 member 7	ENSRNOG00000011644	366432
37938	24	37	8	10	-1,576	-0,656	0,020415428	NM_013196.1	CUST_9880_PI421866198	NM_013196	Ppara	peroxisome proliferator activated receptor alpha	ENSRNOG00000021463	25747
31317	40	67	9	30	-1,576	-0,656	0,033887575	NM_001000527.1	CUST_15057_PI421866198	NM_001000527	Olr1481	olfactory receptor 1481	ENSRNOG00000061453	404974
21550	6	9	1	1	-1,577	-0,657	0,001131206	NM_001024323.1	CUST_576_PI421866198	NM_001024323	Fam71f2	family with sequence similarity 71, member F2	ENSRNOG00000039383	500060
27175	19	29	5	6	-1,578	-0,658	0,00877189	MRBC030003	MRBC030003_P1	MRBC030003	NA	lncRNA	NA	NA
37130	49	75	20	18	-1,578	-0,658	0,020671463	NM_181080.1	CUST_1611_PI421866198	NM_181080	Trim50	tripartite motif-containing 50	ENSRNOG00000022483	288596
37263	18	27	7	3	-1,58	-0,66	0,042402694	NM_001007695.1	CUST_7020_PI421866198	NM_001007695	Tcp11	t-complex 11	ENSRNOG00000000499	309641
35885	51	78	23	22	-1,58	-0,66	0,049798318	NM_053721.1	CUST_2097_PI421866198	NM_053721	Rasgrf2	NA	NA	NA
26181	28	44	6	6	-1,581	-0,661	0,001395049	MRAK150131	MRAK150131_P1	MRAK150131	NA	lncRNA	NA	NA
26672	13	20	3	6	-1,581	-0,661	0,035437088	BC168236	BC168236_P1	BC168236	NA	lncRNA	NA	NA
15304	16	26	4	6	-1,585	-0,665	0,008276219	AY325171	AY325171_P1	AY325171	NA	lncRNA	NA	NA
39877	78	121	24	31	-1,585	-0,664	0,019164925	NM_031653.1	CUST_11233_PI421866198	NM_031653	Ncan	neurocan	ENSRNOG00000048036	58982
24650	9	13	3	2	-1,585	-0,665	0,022796376	uc.396+	uc.396+_P1	uc+	NA	lncRNA	NA	NA
17422	3	6	1	3	-1,586	-0,665	0,040384056	XR_005547	XR_005547_P1	XR_005547	NA	NA	NA	NA

3360	81	126	29	29	-1,587	-0,667	0,011349586	AY169317	AY169317_P1	AY169317	NA	lncRNA	NA	NA
38326	12	19	2	4	-1,589	-0,669	0,001521939	NM_001123469.1	CUST_3747_PI421866198	NM_001123469	Hist2h4	histone cluster 2, H4	ENSRNOG00000048735	364723
39365	72	115	20	40	-1,589	-0,668	0,024045614	NM_001009478.1	CUST_1464_PI421866198	NM_001009478	Saa4	serum amyloid A4	ENSRNOG00000055531	308598
37274	138	236	25	121	-1,59	-0,669	0,029358849	NM_012626.2	CUST_10390_PI421866198	NM_012626	Ppy	pancreatic polypeptide	ENSRNOG00000020874	24677
26893	2	4	0	2	-1,591	-0,67	0,029155076	NM_001024316.1	CUST_619_PI421866198	NM_001024316	Gata5	GATA binding protein 5	ENSRNOG00000058983	499951
37122	46	69	23	16	-1,591	-0,67	0,030090936	uc.343-	uc.343-_P1	uc-	NA	lncRNA	NA	NA
19340	6	10	1	2	-1,592	-0,671	0,004642487	AF390079	AF390079_P1	AF390079	NA	NA	NA	NA
25885	5	8	1	2	-1,592	-0,671	0,010634103	NM_001000532.1	CUST_15050_PI421866198	NM_001000532	Olr1568	NA	NA	NA
31985	3	5	0	2	-1,592	-0,67	0,026358605	NM_001109153.1	CUST_3864_PI421866198	NM_001109153	RGD1559600	RGD1559600	ENSRNOG00000042232	499256
22182	17	27	4	6	-1,594	-0,673	0,003632483	NM_001107251.1	CUST_9109_PI421866198	NM_001107251	Fezf2	Fez family zinc finger 2	ENSRNOG00000009206	305719
8155	5	8	2	1	-1,594	-0,673	0,028230654	MRAK015138	MRAK015138_P1	MRAK015138	NA	lncRNA	NA	NA
20476	5	7	2	1	-1,596	-0,675	0,029216258	NM_001166677.1	CUST_3180_PI421866198	NM_001166677	Tas2r110	taste receptor, type 2, member 110	ENSRNOG00000050637	100310875
35225	129	210	34	74	-1,597	-0,675	0,016490914	NM_001166689.1	CUST_3147_PI421866198	NM_001166689	Tas2r113	taste receptor, type 2, member 113	ENSRNOG00000030752	100310885
43925	585	913	272	280	-1,598	-0,677	0,040890072	NM_001013247.1	CUST_13258_PI421866198	NM_001013247	Sln	sarcolipin	ENSRNOG00000009047	367086
24766	99	165	20	71	-1,599	-0,677	0,016166445	MRAK140270	MRAK140270_P1	MRAK140270	NA	lncRNA	NA	NA
16933	2	4	0	1	-1,601	-0,679	0,006612283	XR_006123	XR_006123_P1	XR_006123	NA	NA	NA	NA
39418	28	47	3	19	-1,601	-0,679	0,009818765	MRAK020698	MRAK020698_P1	MRAK020698	NA	lncRNA	NA	NA
29784	3	4	1	1	-1,602	-0,68	0,002151153	AF196252	AF196252_P1	AF196252	NA	NA	NA	NA
14111	13	20	4	3	-1,602	-0,68	0,006228534	MRAK051163	MRAK051163_P1	MRAK051163	NA	lncRNA	NA	NA
19469	2	4	1	1	-1,602	-0,68	0,008947607	NM_001106422.1	CUST_11483_PI421866198	NM_001106422	Tnik	TRAF2 and NCK interacting kinase	ENSRNOG00000012422	294917
33267	144	252	21	119	-1,603	-0,68	0,040142508	NM_001108763.1	CUST_3455_PI421866198	NM_001108763	Ppcdc	phosphopantothenoylcysteine decarboxylase	ENSRNOG00000018711	103689921
35776	95	152	27	29	-1,606	-0,683	0,003810403	NM_001108348.1	CUST_6111_PI421866198	NM_001108348	Lhx4	LIM homeobox 4	ENSRNOG00000003595	NA
38492	3	4	1	2	-1,607	-0,684	0,024306261	NM_001000073.1	CUST_2496_PI421866198	NM_001000073	Olr951	NA	NA	NA
8052	5	7	3	1	-1,611	-0,688	0,036924041	XR_006116	XR_006116_P1	XR_006116	NA	NA	NA	NA
23489	3	5	1	2	-1,612	-0,689	0,02574735	MRAK040618	MRAK040618_P1	MRAK040618	NA	NA	NA	NA
39749	125	210	10	70	-1,613	-0,689	0,003100009	NM_031517.1	CUST_12040_PI421866198	NM_031517	Met	MET proto-oncogene, receptor tyrosine kinase	ENSRNOG00000052745	24553
40363	102	162	43	55	-1,613	-0,69	0,025550408	NM_212493.1	CUST_12649_PI421866198	NM_212493	Olr1750	olfactory receptor 1750	ENSRNOG00000043481	294204
14159	4	6	1	1	-1,613	-0,69	0,026988993	MRAK148389	MRAK148389_P1	MRAK148389	NA	lncRNA	NA	NA
37907	60	95	24	25	-1,614	-0,69	0,017933465	uc.161+	uc.161+_P1	uc+	NA	lncRNA	NA	NA
7052	5	8	2	2	-1,614	-0,69	0,029315611	MRAK008823	MRAK008823_P1	MRAK008823	NA	NA	NA	NA
15585	24	38	7	4	-1,615	-0,691	0,003697669	XR_007883	XR_007883_P1	XR_007883	NA	lncRNA	NA	NA
10045	3	4	1	1	-1,616	-0,693	0,002782874	MRAK043707	MRAK043707_P1	MRAK043707	NA	NA	NA	NA
37977	15	26	2	9	-1,621	-0,697	0,004805092	uc.254+	uc.254+_P1	uc+	NA	lncRNA	NA	NA
40055	36	57	12	12	-1,623	-0,698	0,00988101	NM_001000027.1	CUST_2735_PI421866198	NM_001000027	Olr1475	olfactory receptor 1475	ENSRNOG00000053411	287487
36668	28	43	10	6	-1,626	-0,701	0,020246566	NM_133543.3	CUST_6404_PI421866198	NM_133543	Rdh7	NA	NA	NA
36904	21	34	7	8	-1,629	-0,704	0,016756254	NM_012900.1	CUST_8653_PI421866198	NM_012900	Ambn	ameloblastin	ENSRNOG00000003718	25376
42447	13	21	4	5	-1,629	-0,704	0,016953422	NM_013042.1	CUST_10801_PI421866198	NM_013042	Tff3	trefoil factor 3	ENSRNOG00000001159	25563
12846	8	12	3	3	-1,629	-0,704	0,031666973	MRuc007aqp	MRuc007aqp_P1	MRuc007aqp	NA	lncRNA	NA	NA
9625	19	30	4	5	-1,631	-0,706	0,001035188	XR_007951	XR_007951_P1	XR_007951	NA	lncRNA	NA	NA
36026	25	41	6	11	-1,633	-0,707	0,003901641	NM_001000680.1	CUST_14412_PI421866198	NM_001000680	Olr500	olfactory receptor 500	ENSRNOG00000056110	404884
10106	32	50	14	12	-1,633	-0,707	0,036465009	MRAK046710	MRAK046710_P1	MRAK046710	NA	lncRNA	NA	NA
23310	2	4	0	1	-1,635	-0,709	0,00887384	MRuc009moc	MRuc009moc_P1	MRuc009moc	NA	NA	NA	NA
27521	2	4	0	2	-1,636	-0,71	0,02400055	NM_001000241.1	CUST_1271_PI421866198	NM_001000241	Olr307	olfactory receptor 307	ENSRNOG00000061350	293605
16032	3	5	1	2	-1,636	-0,71	0,036768667	BC166736	BC166736_P1	BC166736	NA	NA	NA	NA
11466	6	9	1	1	-1,637	-0,711	0,004657572	MRuc009hcy	MRuc009hcy_P1	MRuc009hcy	NA	NA	NA	NA
44979	5	7	2	2	-1,637	-0,711	0,019624676	NM_001009530.1	CUST_941_PI421866198	NM_001009530	NA	NA	NA	NA
40868	7	10	3	3	-1,638	-0,712	0,032994507	NM_001000753.1	CUST_13792_PI421866198	NM_001000753	Olr1345	olfactory receptor 1345	ENSRNOG00000047633	405023
27665	159	276	40	137	-1,64	-0,714	0,023871461	MRuc009aud	MRuc009aud_P1	MRuc009aud	NA	lncRNA	NA	NA
33937	15	26	3	10	-1,641	-0,715	0,018440566	AY155460	AY155460_P1	AY155460	NA	lncRNA	NA	NA
41754	46	73	15	16	-1,642	-0,715	0,011349857	uc.244-	uc.244-_P1	uc-	NA	lncRNA	NA	NA
40092	8	12	3	4	-1,643	-0,717	0,029154342	NM_001109479.1	CUST_1819_PI421866198	NM_001109479	Znrd1as	NA	NA	NA
21902	15	27	3	13	-1,643	-0,716	0,032129938	NM_001082477.2	CUST_14618_PI421866198	NM_001082477	Igf1	insulin-like growth factor 1	ENSRNOG00000004517	24482
36323	14	24	2	4	-1,644	-0,717	0,000235055	MRuc008hcg	MRuc008hcg_P1	MRuc008hcg	NA	lncRNA	NA	NA
36913	10	16	3	3	-1,644	-0,717	0,003606083	NM_001105772.1	CUST_13097_PI421866198	NM_001105772	Prss34	protease, serine, 34	ENSRNOG00000018466	287140
33589	61	103	16	36	-1,645	-0,718	0,013492291	XR_007123	XR_007123_P1	XR_007123	NA	lncRNA	NA	NA
520	12	20	4	5	-1,646	-0,719	0,016733278	AJ286183	AJ286183_P1	AJ286183	NA	lncRNA	NA	NA
42848	4	6	2	2	-1,646	-0,719	0,020132571	uc.323-	uc.323-_P1	uc-	NA	NA	NA	NA
39132	32	48	15	11	-1,646	-0,719	0,040207901	NM_001025280.1	CUST_13178_PI421866198	NM_001025280	Slc10a5	solute carrier family 10, member 5	ENSRNOG00000032426	310230
12608	9	14	2	3	-1,649	-0,722	0,002590086	AY621359	AY621359_P1	AY621359	NA	lncRNA	NA	NA
36703	11	17	4	6	-1,651	-0,723	0,039438398	NM_053354.3	CUST_4664_PI421866198	NM_053354	Dnmt1	DNA methyltransferase 1	ENSRNOG00000039859	84350
13013	13	21	3	1	-1,652	-0,725	0,000203559	MRAK158384	MRAK158384_P1	MRAK158384	NA	lncRNA	NA	NA
27713	9	14	3	2	-1,652	-0,724	0,007692983	XR_005680	XR_005680_P1	XR_005680	NA	lncRNA	NA	NA
15848	3	5	1	2	-1,652	-0,724	0,043195339	NM_001000165.1	CUST_1804_PI421866198	NM_001000165	Olr148	olfactory receptor 148	ENSRNOG00000016993	293285
39288	21	34	9	10	-1,654	-0,726	0,028191349	NM_001110295.1	CUST_8015_PI421866198	NM_001110295	Lypd4	Ly6/Plaur domain containing 4	ENSRNOG00000020098	681194
38579	228	355	119	115	-1,656	-0,728	0,045294642	MRuc008msz	MRuc008msz_P1	MRuc008msz	NA	lncRNA	NA	NA
34928	2	4	0	2	-1,656	-0,728	0,045346721	uc.319-	uc.319-_P1	uc-	NA	NA	NA	NA
17079	3	5	1	2	-1,656	-0,727	0,046428477	MRAK051640	MRAK051640_P1	MRAK051640	NA	NA	NA	NA
19210	148	260	52	153	-1,659	-0,73	0,049830348	NM_001109155.1	CUST_3862_PI421866198	NM_001109155	RGD1563217	similar to RIKEN cDNA 4930451I11	ENSRNOG00000036713	499265
25065	2	4	0	2	-1,662	-0,733	0,045030544	uc.333+	uc.333+_P1	uc+	NA	NA	NA	NA
40068	5	9	2	3	-1,663	-0,733	0,024916019	uc.28-	uc.28-_P1	uc-	NA	NA	NA	NA
41583	4	6	1	1	-1,667	-0,737	0,011768261	uc.71+	uc.71+_P1	uc+	NA	NA	NA	NA
6188	12	21	4	8	-1,667	-0,737	0,019702922	NM_031732.2	CUST_10640_PI421866198	NM_031732	Sult1c3	sulfotransferase family 1C member 3	ENSRNOG00000011269	65185
12883	5	8	2	3	-1,668	-0,738	0,045323078	MRAK016066	MRAK016066_P1	MRAK016066	NA	NA	NA	NA
24317	2	4	0	2	-1,673	-0,743	0,007365843	uc.368-	uc.368-_P1	uc-	NA	NA	NA	NA
4775	4	6	1	1	-1,673	-0,743	0,009523141	BC105757	BC105757_P1	BC105757	NA	NA	NA	NA
32979	10	17	4	5	-1,674	-0,743	0,031294425	XR_007334	XR_007334_P1	XR_007334	NA	lncRNA	NA	NA
6647	11	17	3	2	-1,675	-0,744	0,004949712	XR_007095	XR_007095_P1	XR_007095	NA	lncRNA	NA	NA
14180	3	5	1	1	-1,675	-0,745	0,008262178	XR_007857	XR_007857_P1	XR_007857	NA	NA	NA	NA
2028	10	16	5	2	-1,678	-0,747	0,04811428	MRAK052539	MRAK052539_P1	MRAK052539	NA	lncRNA	NA	NA
39335	52	83	28	25	-1,68	-0,749	0,035802708	NM_001000604.1	CUST_14532_PI421866198	NM_001000604	Olr784	olfactory receptor 784	ENSRNOG00000050402	404793
26272	2	4	0	2	-1,681	-0,749	0,024504918	NM_001109020.1	CUST_4654_PI421866198	NM_001109020	Upk3bl1	NA	NA	NA
1099	269	443	126	155	-1,681	-0,749	0,03016824	MRAK079352	MRAK079352_P1	MRAK079352	NA	lncRNA	NA	NA
27794	6	10	2	4	-1,681	-0,75	0,034685094	uc.1+	uc.1+_P1	uc+	NA	NA	NA	NA
35713	25	41	6	8	-1,682	-0,75	0,002696442	uc.327+	uc.327+_P1	uc+	NA	lncRNA	NA	NA
27547	3	5	1	1	-1,683	-0,751	0,003829486	NM_001000803.1	CUST_13129_PI421866198	NM_001000803	Olr1262	olfactory receptor 1262	ENSRNOG00000045981	405089
33946	10	18	3	8	-1,685	-0,752	0,034243493	NM_199490.1	CUST_9927_PI421866198	NM_199490	Dsg4	desmoglein 4	ENSRNOG00000022364	291754
35734	26	43	7	3	-1,686	-0,754	0,002057612	NM_001106984.1	CUST_8328_PI421866198	NM_001106984	Prss22	protease, serine, 22	ENSRNOG00000039698	302971
12954	2	4	0	1	-1,69	-0,757	0,001252969	XR_005963	XR_005963_P1	XR_005963	NA	NA	NA	NA
12839	15	26	1	7	-1,69	-0,757	0,001311053	MRAK042984	MRAK042984_P1	MRAK042984	NA	lncRNA	NA	NA
12305	67	109	34	38	-1,692	-0,759	0,02759853	MRAK087326	MRAK087326_P1	MRAK087326	NA	lncRNA	NA	NA
33260	16	27	7	8	-1,692	-0,759	0,032710322	NM_001107473.1	CUST_8171_PI421866198	NM_001107473	Zim1	zinc finger, imprinted 1	ENSRNOG00000015071	308322
14739	2	4	0	2	-1,694	-0,76	0,018532045	MRAK034779	MRAK034779_P1	MRAK034779	NA	NA	NA	NA
25083	3	5	1	3	-1,696	-0,762	0,021842672	NM_177482.1	CUST_4303_PI421866198	NM_177482	Scgb1d2	secretoglobin, family 1D, member 2	ENSRNOG00000020294	309203
44563	4	7	1	3	-1,697	-0,763	0,020591719	NM_001108183.1	CUST_7227_PI421866198	NM_001108183	Manf	mesencephalic astrocyte-derived neurotrophic factor	ENSRNOG00000014201	315989
39648	9	14	3	2	-1,698	-0,764	0,007457365	NM_001000036.1	CUST_2700_PI421866198	NM_001000036	Olr1514	olfactory receptor 1514	ENSRNOG00000033267	287514
37497	250	450	35	198	-1,699	-0,765	0,007912229	NM_017275.1	CUST_11960_PI421866198	NM_017275	Pnck	pregnancy up-regulated nonubiquitous CaM kinase	ENSRNOG00000058317	29660
20541	7	12	2	3	-1,701	-0,767	0,004013178	NM_175601.1	CUST_8996_PI421866198	NM_175601	Taar8b	NA	NA	NA
44824	69	116	16	19	-1,702	-0,767	0,002122146	NM_019157.2	CUST_6622_PI421866198	NM_019157	Aqp7	aquaporin 7	ENSRNOG00000009686	29171
40496	12	21	2	5	-1,704	-0,769	0,000829792	NM_001108659.1	CUST_4196_PI421866198	NM_001108659	Dctn3	dynactin subunit 3	ENSRNOG00000014208	362504
19448	7	12	2	3	-1,705	-0,77	0,003169543	uc.326+	uc.326+_P1	uc+	NA	NA	NA	NA
32393	30	48	11	12	-1,705	-0,769	0,026070803	AF098320	AF098320_P1	AF098320	NA	lncRNA	NA	NA
24735	10	16	3	4	-1,706	-0,77	0,015894987	uc.35+	uc.35+_P1	uc+	NA	lncRNA	NA	NA
23704	4	6	1	2	-1,707	-0,771	0,027905099	AM981048	AM981048_P1	AM981048	NA	NA	NA	NA
18519	324	522	194	182	-1,707	-0,772	0,038035556	MRuc009kqo	MRuc009kqo_P1	MRuc009kqo	NA	lncRNA	NA	NA
39798	23	38	6	7	-1,71	-0,774	0,001589447	NM_001000764.1	CUST_13770_PI421866198	NM_001000764	Olr312	olfactory receptor 312	ENSRNOG00000050629	405038
7412	6	9	2	1	-1,71	-0,774	0,01805304	AY539914	AY539914_P1	AY539914	NA	lncRNA	NA	NA
37594	27	44	11	3	-1,711	-0,775	0,004109391	uc.300+	uc.300+_P1	uc+	NA	lncRNA	NA	NA
29275	4	6	2	2	-1,711	-0,775	0,02750537	NM_001000870.1	CUST_13009_PI421866198	NM_001000870	Olr1162	olfactory receptor 1162	ENSRNOG00000047533	405167
13007	5	9	2	3	-1,713	-0,776	0,04661172	MRAK028483	MRAK028483_P1	MRAK028483	NA	NA	NA	NA
883	4	8	1	4	-1,714	-0,778	0,020825409	XR_007833	XR_007833_P1	XR_007833	NA	NA	NA	NA
32442	50	79	20	16	-1,714	-0,778	0,02791996	AJ286211	AJ286211_P1	AJ286211	NA	lncRNA	NA	NA
7403	18	31	5	7	-1,717	-0,78	0,003620874	MRuc008ihi	MRuc008ihi_P1	MRuc008ihi	NA	lncRNA	NA	NA
12894	2	4	0	2	-1,722	-0,784	0,010983371	MRAK021372	MRAK021372_P1	MRAK021372	NA	NA	NA	NA
13379	4	7	1	1	-1,723	-0,785	0,000948009	MRAK090235	MRAK090235_P1	MRAK090235	NA	NA	NA	NA
16034	17	28	7	6	-1,723	-0,785	0,040334518	MRAK040445	MRAK040445_P1	MRAK040445	NA	lncRNA	NA	NA
6577	2	4	0	2	-1,725	-0,786	0,012219206	MRAK148525	MRAK148525_P1	MRAK148525	NA	NA	NA	NA
417	38	60	18	7	-1,727	-0,788	0,0206103	AF127390	AF127390_P1	AF127390	NA	lncRNA	NA	NA
35059	35	62	7	23	-1,728	-0,789	0,005586388	MRAK078313	MRAK078313_P1	MRAK078313	NA	lncRNA	NA	NA
30565	40	64	21	12	-1,729	-0,79	0,013394154	MRAK141332	MRAK141332_P1	MRAK141332	NA	lncRNA	NA	NA
4417	6	10	2	2	-1,73	-0,79	0,008840791	MRAK049176	MRAK049176_P1	MRAK049176	NA	NA	NA	NA
16000	4	7	2	2	-1,73	-0,791	0,033253253	MRAK051354	MRAK051354_P1	MRAK051354	NA	NA	NA	NA
37167	9	14	4	3	-1,731	-0,791	0,018777438	NM_001037350.1	CUST_12537_PI421866198	NM_001037350	LOC296235	similar to Cystatin S precursor (LM protein)	ENSRNOG00000036952	296235
16070	1093	1996	367	1014	-1,732	-0,793	0,027604608	NM_001077642.1	CUST_6225_PI421866198	NM_001077642	Cfd	complement factor D	ENSRNOG00000033564	54249
8334	3	5	1	1	-1,733	-0,793	0,008593192	XR_006072	XR_006072_P1	XR_006072	NA	NA	NA	NA
41378	172	276	108	94	-1,734	-0,794	0,048306564	uc.325-	uc.325-_P1	uc-	NA	lncRNA	NA	NA
11041	5	8	1	1	-1,736	-0,796	0,011273992	MRAK047431	MRAK047431_P1	MRAK047431	NA	NA	NA	NA
37592	52	91	16	33	-1,737	-0,797	0,012845756	NM_012871.2	CUST_8980_PI421866198	NM_012871	Oxtr	oxytocin receptor	ENSRNOG00000005806	25342
41427	8	13	2	3	-1,742	-0,8	0,001219443	NM_181692.1	CUST_13560_PI421866198	NM_181692	Kiss1	KiSS-1 metastasis-suppressor	ENSRNOG00000047481	289023
29356	25	43	10	16	-1,744	-0,802	0,048766706	NM_012624.3	CUST_10394_PI421866198	NM_012624	Pklr	pyruvate kinase, liver and RBC	ENSRNOG00000020420	24651
15035	3	5	0	3	-1,748	-0,805	0,016931255	XR_005726	XR_005726_P1	XR_005726	NA	NA	NA	NA
44231	428	794	95	401	-1,749	-0,806	0,010922505	NM_001000247.1	CUST_1263_PI421866198	NM_001000247	Olr325	olfactory receptor 325	ENSRNOG00000021071	293763
34279	21	36	8	12	-1,752	-0,809	0,021337647	uc.160-	uc.160-_P1	uc-	NA	lncRNA	NA	NA
24907	7	11	3	3	-1,753	-0,81	0,039760699	MRAK165050	MRAK165050_P1	MRAK165050	NA	lncRNA	NA	NA
33698	3	6	1	2	-1,755	-0,812	0,00746293	NM_001000820.1	CUST_13084_PI421866198	NM_001000820	Olr1194	olfactory receptor 1194	ENSRNOG00000045972	405108
25249	10	18	3	6	-1,755	-0,812	0,010621305	XR_008714	XR_008714_P1	XR_008714	NA	lncRNA	NA	NA
16972	1295	2372	310	911	-1,755	-0,812	0,012560055	NM_001134614.1	CUST_6053_PI421866198	NM_001134614	Fabp12	fatty acid binding protein 12	ENSRNOG00000038825	499570
23010	6	10	2	3	-1,757	-0,813	0,031550611	NM_001099460.1	CUST_13518_PI421866198	NM_001099460	Vom2r3	vomeronasal 2 receptor, 3	ENSRNOG00000046189	NA
37601	8	13	1	2	-1,758	-0,814	0,000250801	MRAK017983	MRAK017983_P1	MRAK017983	NA	lncRNA	NA	NA
35099	4	6	2	3	-1,759	-0,815	0,046051855	uc.327-	uc.327-_P1	uc-	NA	NA	NA	NA
16650	7	14	2	5	-1,761	-0,816	0,01827472	NM_001008911.1	CUST_1689_PI421866198	NM_001008911	NA	NA	NA	NA
27117	14	24	3	4	-1,764	-0,819	0,000194601	MRAK032649	MRAK032649_P1	MRAK032649	NA	lncRNA	NA	NA
37939	103	183	34	63	-1,766	-0,82	0,009730069	NM_001109103.1	CUST_3991_PI421866198	NM_001109103	RGD1565212	similar to 4930578I06Rik protein	ENSRNOG00000012402	498534
4469	60	114	6	50	-1,769	-0,823	0,005188992	MRAK050890	MRAK050890_P1	MRAK050890	NA	lncRNA	NA	NA
8583	6	11	3	3	-1,769	-0,823	0,013797648	NM_001000218.1	CUST_1369_PI421866198	NM_001000218	Olr251	olfactory receptor 251	ENSRNOG00000050593	293394
40174	11	20	1	4	-1,773	-0,826	7,00E-05	NM_053408.1	CUST_4047_PI421866198	NM_053408	Chst3	carbohydrate sulfotransferase 3	ENSRNOG00000000572	84468
26343	9	16	3	5	-1,774	-0,827	0,020171029	NM_001000677.1	CUST_14435_PI421866198	NM_001000677	Olr508	olfactory receptor 508	ENSRNOG00000053924	404880
9653	4	8	1	2	-1,775	-0,828	0,008260846	AM293796	AM293796_P1	AM293796	NA	NA	NA	NA
35791	4	6	1	1	-1,777	-0,829	0,004131204	NM_001014270.1	CUST_9423_PI421866198	NM_001014270	Slco6d1	solute carrier organic anion transporter family, member 6d1	ENSRNOG00000047903	367321
25582	6	10	2	3	-1,777	-0,83	0,023116683	NM_130813.1	CUST_14358_PI421866198	NM_130813	Kcnk15	potassium two pore domain channel subfamily K member 15	ENSRNOG00000010816	156873
9887	3	5	1	1	-1,778	-0,83	0,001812462	MRuc009bks	MRuc009bks_P1	MRuc009bks	NA	NA	NA	NA
37391	69	119	34	41	-1,778	-0,83	0,017509361	NM_001106954.1	CUST_8358_PI421866198	NM_001106954	RGD1561661	similar to Ferritin light chain (Ferritin L subunit)	ENSRNOG00000023094	302536
40061	6	10	3	3	-1,779	-0,831	0,018519248	uc.416+	uc.416+_P1	uc+	NA	NA	NA	NA
18387	11	19	4	1	-1,78	-0,832	0,013130477	XR_008168	XR_008168_P1	XR_008168	NA	lncRNA	NA	NA
26438	4	7	1	2	-1,782	-0,833	0,002084767	MRAK005704	MRAK005704_P1	MRAK005704	NA	NA	NA	NA
42355	43	75	13	22	-1,782	-0,833	0,005796084	NM_001038588.1	CUST_7976_PI421866198	NM_001038588	Prodh2	proline dehydrogenase 2	ENSRNOG00000057578	361538
28759	13	24	5	9	-1,783	-0,834	0,015261957	MRAK084110	MRAK084110_P1	MRAK084110	NA	lncRNA	NA	NA
30017	59	97	28	22	-1,784	-0,835	0,021284051	XR_005786	XR_005786_P1	XR_005786	NA	lncRNA	NA	NA
19536	35	61	17	23	-1,785	-0,836	0,021427327	XR_006153	XR_006153_P1	XR_006153	NA	lncRNA	NA	NA
41680	9	15	4	5	-1,786	-0,837	0,025413769	uc.292-	uc.292-_P1	uc-	NA	NA	NA	NA
38788	42	79	14	45	-1,786	-0,837	0,028465394	NM_001108881.1	CUST_2930_PI421866198	NM_001108881	Rnf144b	ring finger protein 144B	ENSRNOG00000016123	364681
1525	3	6	1	4	-1,788	-0,839	0,033224881	NM_017164.2	CUST_12661_PI421866198	NM_017164	Capza3	capping actin protein of muscle Z-line alpha subunit 3	ENSRNOG00000008727	29324
35339	11	17	7	3	-1,788	-0,838	0,035332981	NM_001108058.1	CUST_7653_PI421866198	NM_001108058	Spaca5	sperm acrosome associated 5	ENSRNOG00000045622	314431
42784	19	34	4	11	-1,789	-0,839	0,0030475	NM_001109217.1	CUST_3288_PI421866198	NM_001109217	Nupl2	nucleoporin like 2	ENSRNOG00000010435	499974
12281	2	4	0	2	-1,795	-0,844	0,006945004	MRAK132138	MRAK132138_P1	MRAK132138	NA	NA	NA	NA
9805	5	9	3	3	-1,795	-0,844	0,042750027	XR_006957	XR_006957_P1	XR_006957	NA	NA	NA	NA
39494	17	30	7	10	-1,797	-0,845	0,013287068	NM_001135777.1	CUST_1632_PI421866198	NM_001135777	Gngt1	G protein subunit gamma transducin 1	ENSRNOG00000010546	680149
43631	23	43	4	16	-1,799	-0,847	0,002959188	NM_024357.2	CUST_12404_PI421866198	NM_024357	Htt	huntingtin	ENSRNOG00000011073	29424
20267	7	13	2	3	-1,804	-0,851	0,001243664	MRAK144466	MRAK144466_P1	MRAK144466	NA	lncRNA	NA	NA
12226	40	69	21	19	-1,806	-0,853	0,013012593	MRAK158936	MRAK158936_P1	MRAK158936	NA	lncRNA	NA	NA
4158	43	79	7	19	-1,807	-0,853	0,000467931	XR_005898	XR_005898_P1	XR_005898	NA	lncRNA	NA	NA
26310	4	8	1	2	-1,807	-0,853	0,004487594	XR_005432	XR_005432_P1	XR_005432	NA	NA	NA	NA
37763	86	152	33	45	-1,807	-0,853	0,008502478	NM_001108616.1	CUST_4310_PI421866198	NM_001108616	Agap3	ArfGAP with GTPase domain, ankyrin repeat and PH domain 3	ENSRNOG00000012764	362300
24394	6	12	1	3	-1,81	-0,856	0,000951638	NM_133291.1	CUST_7762_PI421866198	NM_133291	Sval2	seminal vesicle antigen-like 2	ENSRNOG00000025541	84605
35280	22	36	11	8	-1,81	-0,856	0,033459751	XR_007033	XR_007033_P1	XR_007033	NA	lncRNA	NA	NA
37417	63	115	15	30	-1,812	-0,858	0,001596502	NM_138872.1	CUST_3402_PI421866198	NM_138872	Smad9	SMAD family member 9	ENSRNOG00000000091	85435
8485	4	7	2	1	-1,812	-0,858	0,010078669	MRuc008dyv	MRuc008dyv_P1	MRuc008dyv	NA	NA	NA	NA
38382	6	11	2	4	-1,813	-0,858	0,011368934	uc.4-	uc.4-_P1	uc-	NA	NA	NA	NA
42085	140	252	45	95	-1,813	-0,858	0,019510764	NM_001004097.1	CUST_4965_PI421866198	NM_001004097	Try10	trypsin 10	ENSRNOG00000050119	408247
39885	12	22	5	7	-1,813	-0,858	0,026230925	NM_054005.1	CUST_3103_PI421866198	NM_054005	Cuzd1	CUB and zona pellucida-like domains 1	ENSRNOG00000029945	117179
25164	9	16	3	4	-1,814	-0,859	0,013167807	MRAK045020	MRAK045020_P1	MRAK045020	NA	lncRNA	NA	NA
35442	9	15	4	6	-1,814	-0,859	0,02276326	NM_001000869.1	CUST_13015_PI421866198	NM_001000869	Olr1163	olfactory receptor 1163	ENSRNOG00000030569	405166
31820	4	7	1	2	-1,815	-0,86	0,003133896	U65217	U65217_P1	U65217	NA	NA	NA	NA
34748	175	329	65	160	-1,815	-0,86	0,021201026	MRAK085890	MRAK085890_P1	MRAK085890	NA	lncRNA	NA	NA
13081	39	67	20	24	-1,816	-0,861	0,037534448	MRAK043449	MRAK043449_P1	MRAK043449	NA	lncRNA	NA	NA
18543	8	15	3	4	-1,818	-0,862	0,011840879	AY383712	AY383712_P1	AY383712	NA	lncRNA	NA	NA
13696	2	4	0	3	-1,819	-0,863	0,02658921	NM_001000285.1	CUST_1139_PI421866198	NM_001000285	Olr443	olfactory receptor 443	ENSRNOG00000050082	295715
10794	4	8	2	2	-1,82	-0,864	0,013534148	MRAK155338	MRAK155338_P1	MRAK155338	NA	NA	NA	NA
19926	5	9	2	5	-1,82	-0,864	0,046362936	MRuc009dng	MRuc009dng_P1	MRuc009dng	NA	NA	NA	NA
39143	8	14	4	7	-1,822	-0,866	0,02894222	NM_001008821.1	CUST_2374_PI421866198	NM_001008821	Krt40	keratin 40	ENSRNOG00000013896	287679
36996	102	185	29	51	-1,824	-0,867	0,004072502	NM_020090.1	CUST_11578_PI421866198	NM_020090	Slc24a1	solute carrier family 24 member 1	ENSRNOG00000052051	56814
36865	11	22	1	9	-1,835	-0,876	0,003621127	MRAK040122	MRAK040122_P1	MRAK040122	NA	lncRNA	NA	NA
12849	21	35	9	5	-1,835	-0,876	0,028264105	U26310	U26310_P1	U26310	NA	lncRNA	NA	NA
9862	8	15	4	5	-1,836	-0,877	0,019887391	BC158653	BC158653_P1	BC158653	NA	lncRNA	NA	NA
39088	7	11	2	2	-1,842	-0,881	0,005974214	NM_001001357.1	CUST_12669_PI421866198	NM_001001357	Olr907	olfactory receptor 907	ENSRNOG00000053774	NA
41295	59	101	33	27	-1,843	-0,882	0,01404279	NM_001013102.1	CUST_14170_PI421866198	NM_001013102	Theg	theg spermatid protein	ENSRNOG00000007960	299599
36355	69	119	41	35	-1,844	-0,883	0,018225163	NM_001000195.1	CUST_1707_PI421866198	NM_001000195	Olr210	olfactory receptor 210	ENSRNOG00000029365	293357
44551	18	32	8	4	-1,846	-0,884	0,005511272	uc.202-	uc.202-_P1	uc-	NA	lncRNA	NA	NA
39861	7	12	3	2	-1,847	-0,885	0,005807995	NM_001000925.1	CUST_12449_PI421866198	NM_001000925	Olr661	olfactory receptor 661	ENSRNOG00000046340	405243
35028	4	8	1	5	-1,849	-0,887	0,035468121	uc.244+	uc.244+_P1	uc+	NA	NA	NA	NA
8457	5	8	2	1	-1,852	-0,889	0,00498915	MRAK089638	MRAK089638_P1	MRAK089638	NA	NA	NA	NA
11827	6	11	3	3	-1,852	-0,889	0,010137446	AB288378	AB288378_P1	AB288378	NA	lncRNA	NA	NA
44142	13	23	6	8	-1,853	-0,89	0,043613311	uc.314-	uc.314-_P1	uc-	NA	lncRNA	NA	NA
18133	10	16	5	2	-1,855	-0,891	0,02342251	MRBC048721	MRBC048721_P1	MRBC048721	NA	lncRNA	NA	NA
36107	72	130	32	53	-1,855	-0,891	0,026901363	MRAK087001	MRAK087001_P1	MRAK087001	NA	lncRNA	NA	NA
38490	30	56	12	15	-1,861	-0,896	0,003360449	NM_001000434.1	CUST_221_PI421866198	NM_001000434	Olr1169	olfactory receptor 1169	ENSRNOG00000047916	300425
13483	4	8	1	3	-1,863	-0,898	0,002337079	MRAK014875	MRAK014875_P1	MRAK014875	NA	NA	NA	NA
33127	57	96	40	31	-1,868	-0,901	0,026393001	NM_001107094.1	CUST_10334_PI421866198	NM_001107094	Hoxd10	homeo box D10	ENSRNOG00000001581	NA
41249	148	276	81	134	-1,868	-0,901	0,033508726	NM_198784.1	CUST_11602_PI421866198	NM_198784	Mup4	major urinary protein 4	ENSRNOG00000033130	259247
34793	37	68	14	19	-1,869	-0,902	0,009586684	NM_133554.2	CUST_6367_PI421866198	NM_133554	Slc17a1	solute carrier family 17 member 1	ENSRNOG00000042692	NA
25666	10	19	3	4	-1,87	-0,903	0,002168282	XR_009421	XR_009421_P1	XR_009421	NA	lncRNA	NA	NA
45158	6	11	3	4	-1,87	-0,903	0,011769515	NM_001000012.1	CUST_2768_PI421866198	NM_001000012	Olr1432	olfactory receptor 1432	ENSRNOG00000002858	287321
33519	37	70	7	19	-1,871	-0,904	0,001256839	NM_001047942.1	CUST_203_PI421866198	NM_001047942	LOC499541	LRRGT00045	ENSRNOG00000030171	499541
34141	8	15	3	4	-1,871	-0,904	0,004339279	MRAK037793	MRAK037793_P1	MRAK037793	NA	lncRNA	NA	NA
8653	2	5	0	3	-1,872	-0,904	0,030630333	NM_001115029.2	CUST_8276_PI421866198	NM_001115029	Lekr1	leucine, glutamate and lysine rich 1	ENSRNOG00000031247	361953
14449	3	6	1	4	-1,873	-0,906	0,045078834	NM_001000815.1	CUST_13101_PI421866198	NM_001000815	Olr1213	olfactory receptor 1213	ENSRNOG00000051323	405101
44657	10	18	3	5	-1,874	-0,906	0,00741853	NM_001000854.1	CUST_13028_PI421866198	NM_001000854	Olr799	olfactory receptor 799	ENSRNOG00000012214	405144
34028	39	73	10	17	-1,877	-0,909	0,001164018	NM_023985.1	CUST_11837_PI421866198	NM_023985	Trib1	tribbles pseudokinase 1	ENSRNOG00000004100	78969
44473	4	8	2	3	-1,881	-0,912	0,017046736	uc.35-	uc.35-_P1	uc-	NA	NA	NA	NA

39129	107	206	44	98	-1,888	-0,917	0,018041924	NM_001145538.1	CUST_8975_PI421866198	NM_001145538	LOC687707	hypothetical protein LOC687707	ENSRNOG00000042847	687707
41419	7	14	3	6	-1,889	-0,918	0,029797212	NM_001037507.1	CUST_11199_PI421866198	NM_001037507	Defb2	defensin beta 2	ENSRNOG00000038147	641631
21135	4	7	3	2	-1,889	-0,918	0,04300451	NM_001000448.1	CUST_194_PI421866198	NM_001000448	Olr1240	olfactory receptor 1240	ENSRNOG00000049725	103692019
34449	56	107	19	37	-1,891	-0,919	0,005820472	uc.155-	uc.155-_P1	uc-	NA	lncRNA	NA	NA
16498	5	9	2	1	-1,893	-0,921	0,012027864	MRAK155635	MRAK155635_P1	MRAK155635	NA	NA	NA	NA
11726	253	514	89	327	-1,893	-0,92	0,026946799	NM_012632.1	CUST_10355_PI421866198	NM_012632	Prp15	proline-rich protein 15	ENSRNOG00000028983	24687
38998	6	12	2	4	-1,9	-0,926	0,001573101	NM_001000257.1	CUST_1220_PI421866198	NM_001000257	Olr357	olfactory receptor 357	ENSRNOG00000053559	293794
34437	4	7	1	2	-1,901	-0,927	0,009757496	NM_001107014.1	CUST_10589_PI421866198	NM_001107014	Alox12e	arachidonate 12-lipoxygenase, epidermal	ENSRNOG00000019074	303252
31825	18	38	5	21	-1,901	-0,927	0,035264456	XR_008349	XR_008349_P1	XR_008349	NA	lncRNA	NA	NA
41463	6	12	2	3	-1,902	-0,927	0,002330276	NM_001080938.1	CUST_2902_PI421866198	NM_001080938	Tas2r124	taste receptor, type 2, member 124	ENSRNOG00000021430	690472
42180	22	39	10	4	-1,902	-0,927	0,003182448	NM_001108462.1	CUST_5407_PI421866198	NM_001108462	Mthfd1l	methylenetetrahydrofolate dehydrogenase (NADP+ dependent) 1-like	ENSRNOG00000019582	361472
41937	135	237	52	56	-1,902	-0,928	0,024473039	NM_001000192.1	CUST_1711_PI421866198	NM_001000192	Olr204	olfactory receptor 204	ENSRNOG00000048643	293351
44550	23	42	10	10	-1,906	-0,931	0,005624359	NM_001007635.2	CUST_7153_PI421866198	NM_001007635	tGap1	GTPase activating protein testicular GAP1	ENSRNOG00000022511	294892
39758	3	6	1	4	-1,906	-0,93	0,029528602	uc.273+	uc.273+_P1	uc+	NA	NA	NA	NA
25239	3	6	1	3	-1,909	-0,933	0,008854106	MRAK038803	MRAK038803_P1	MRAK038803	NA	NA	NA	NA
37768	16	29	9	12	-1,913	-0,936	0,037525167	NM_001000791.1	CUST_13665_PI421866198	NM_001000791	Olr1329	olfactory receptor 1329	ENSRNOG00000060221	405075
44566	122	231	28	40	-1,917	-0,939	0,000171472	NM_012770.1	CUST_9503_PI421866198	NM_012770	Gucy1b2	guanylate cyclase 1 soluble subunit beta 2	ENSRNOG00000009440	25206
42685	5	9	2	2	-1,917	-0,939	0,006536085	NM_001145072.1	CUST_12362_PI421866198	NM_001145072	Iqcf5	IQ motif containing F5	ENSRNOG00000013164	503228
40206	34	63	15	18	-1,92	-0,941	0,013200711	NM_001000533.1	CUST_15049_PI421866198	NM_001000533	Olr1581	olfactory receptor 1581	ENSRNOG00000046418	364054
11214	3	6	1	1	-1,922	-0,942	0,00032376	NM_001000660.1	CUST_14455_PI421866198	NM_001000660	Olr586	olfactory receptor 586	ENSRNOG00000030946	404860
44397	36	76	6	40	-1,923	-0,943	0,008160787	uc.305+	uc.305+_P1	uc+	NA	lncRNA	NA	NA
34115	3	6	1	1	-1,93	-0,949	0,000218192	NM_001107757.1	CUST_6955_PI421866198	NM_001107757	Aven	apoptosis and caspase activation inhibitor	ENSRNOG00000006419	311299
16048	2	5	0	3	-1,94	-0,956	0,019049328	NM_001000859.1	CUST_13023_PI421866198	NM_001000859	Olr1192	olfactory receptor 1192	ENSRNOG00000033025	405152
10432	8	16	2	8	-1,943	-0,958	0,0046727	MRAK042044	MRAK042044_P1	MRAK042044	NA	lncRNA	NA	NA
15108	3	6	1	3	-1,944	-0,959	0,044537122	NM_001001081.1	CUST_14718_PI421866198	NM_001001081	Olr1195	olfactory receptor 1195	ENSRNOG00000047801	405971
8233	31	57	16	16	-1,945	-0,96	0,016260209	AJ535460	AJ535460_P1	AJ535460	NA	lncRNA	NA	NA
14254	3	7	2	5	-1,947	-0,961	0,048811992	NM_001106315.1	CUST_12269_PI421866198	NM_001106315	Pkp3	plakophilin 3	ENSRNOG00000015152	293619
43764	6	11	3	4	-1,955	-0,967	0,009077349	NM_001000850.1	CUST_13032_PI421866198	NM_001000850	Olr808	olfactory receptor 808	ENSRNOG00000029299	405140
34447	77	157	22	74	-1,957	-0,968	0,005338059	MRAK052925	MRAK052925_P1	MRAK052925	NA	lncRNA	NA	NA
38407	4	8	1	5	-1,96	-0,971	0,024946063	NM_001047925.1	CUST_233_PI421866198	NM_001047925	LOC498231	LRRGT00144	ENSRNOG00000029418	498231
36582	9	18	2	8	-1,962	-0,972	0,005494022	NM_019145.1	CUST_6652_PI421866198	NM_019145	Chrng	NA	NA	NA
42266	21	40	8	13	-1,962	-0,972	0,008050242	NM_001108851.1	CUST_2971_PI421866198	NM_001108851	Zscan25	NA	NA	NA
38615	14	22	12	4	-1,968	-0,977	0,046569892	uc.342+	uc.342+_P1	uc+	NA	lncRNA	NA	NA
36779	10	19	3	5	-1,969	-0,977	0,004338157	MRAK079392	MRAK079392_P1	MRAK079392	NA	lncRNA	NA	NA
35692	3	6	1	4	-1,969	-0,978	0,024418231	NM_022957.2	CUST_98_PI421866198	NM_022957	Serpina5	serpin family A member 5	ENSRNOG00000009855	65051
40057	9	15	5	2	-1,97	-0,978	0,009971937	NM_199266.2	CUST_11416_PI421866198	NM_199266	P22k15	cystatin related protein 2	ENSRNOG00000030184	296229
38011	12	22	9	9	-1,972	-0,98	0,031765515	NM_001002834.1	CUST_6879_PI421866198	NM_001002834	Senp18	Sumo1/sentrin/SMT3 specific peptidase 18	ENSRNOG00000050153	408222
24819	3	6	1	2	-1,974	-0,981	0,002444441	MRAK045143	MRAK045143_P1	MRAK045143	NA	NA	NA	NA
35731	203	357	152	117	-1,975	-0,982	0,021974329	NM_001008904.1	CUST_1723_PI421866198	NM_001008904	NA	NA	NA	NA
5154	281	556	105	208	-1,978	-0,984	0,002982947	MRuc009gcf	MRuc009gcf_P1	MRuc009gcf	NA	lncRNA	NA	NA
42434	14	27	6	12	-1,98	-0,985	0,012367584	NM_053303.1	CUST_4800_PI421866198	NM_053303	Cxcr5	C-X-C motif chemokine receptor 5	ENSRNOG00000012430	29363
33849	4	8	1	2	-1,983	-0,987	0,002319642	MRAK080776	MRAK080776_P1	MRAK080776	NA	NA	NA	NA
38864	83	163	34	63	-1,987	-0,991	0,007065229	NM_013133.1	CUST_10067_PI421866198	NM_013133	Glra1	glycine receptor, alpha 1	ENSRNOG00000013588	25674
40320	40	76	17	17	-1,988	-0,992	0,004496763	NM_031605.2	CUST_11363_PI421866198	NM_031605	Cyp4a8	cytochrome P450, family 4, subfamily a, polypeptide 8	ENSRNOG00000008842	266674
42195	65	137	25	82	-1,99	-0,993	0,01308386	MRNR_024051	MRNR_024051_P1	MRNR_024051	NA	lncRNA	NA	NA
10010	572	1114	366	497	-1,991	-0,993	0,027654461	Y13972	Y13972_P1	Y13972	NA	lncRNA	NA	NA
35805	7	16	3	10	-1,995	-0,996	0,039831059	NM_001000845.1	CUST_13038_PI421866198	NM_001000845	Olr816	olfactory receptor 816	ENSRNOG00000005431	405134
42676	14	26	8	8	-1,996	-0,997	0,019611868	NM_001012130.1	CUST_1800_PI421866198	NM_001012130	Pgk2	phosphoglycerate kinase 2	ENSRNOG00000013600	316265
39291	13	24	6	4	-2	-1	0,005646287	NM_001033710.1	CUST_7904_PI421866198	NM_001033710	Prss16	protease, serine 16	ENSRNOG00000057865	364719
34111	173	360	31	125	-2,006	-1,004	0,000944347	NM_001013904.1	CUST_8827_PI421866198	NM_001013904	Cyp2c6v1	cytochrome P450, family 2, subfamily C, polypeptide 6, variant 1	ENSRNOG00000024016	293989
33680	3	7	1	1	-2,015	-1,011	0,00177802	uc.31-	uc.31-_P1	uc-	NA	NA	NA	NA
35535	14	28	5	11	-2,016	-1,011	0,008348964	AJ271303	AJ271303_P1	AJ271303	NA	lncRNA	NA	NA
10850	177	322	116	108	-2,016	-1,011	0,020365997	BC166600	BC166600_P1	BC166600	NA	lncRNA	NA	NA
22654	5	9	2	3	-2,016	-1,012	0,021045712	NM_001107851.1	CUST_6318_PI421866198	NM_001107851	Tsga13	testis specific, 13	ENSRNOG00000011108	688848
39332	659	1506	133	929	-2,018	-1,013	0,013706927	NM_001102364.1	CUST_9375_PI421866198	NM_001102364	Cldn6	claudin 6	ENSRNOG00000003648	287098
40815	7	14	2	1	-2,02	-1,014	0,000109355	NM_001013219.1	CUST_13351_PI421866198	NM_001013219	St3gal1	ST3 beta-galactoside alpha-2,3-sialyltransferase 1	ENSRNOG00000008209	362924
34441	6	13	1	5	-2,02	-1,015	0,014862749	NM_001037526.1	CUST_11149_PI421866198	NM_001037526	Defb41	defensin beta 41	ENSRNOG00000038760	641650
34951	5	11	1	5	-2,025	-1,018	0,007480571	uc.226-	uc.226-_P1	uc-	NA	NA	NA	NA
3854	402	845	114	383	-2,026	-1,019	0,003283361	MRAK079124	MRAK079124_P1	MRAK079124	NA	lncRNA	NA	NA
12925	9	16	4	3	-2,026	-1,019	0,00853839	MRuc009umr	MRuc009umr_P1	MRuc009umr	NA	lncRNA	NA	NA
17257	3	7	1	4	-2,028	-1,02	0,014475932	uc.243+	uc.243+_P1	uc+	NA	NA	NA	NA
35536	8	18	3	16	-2,03	-1,021	0,03508868	NM_001107274.1	CUST_9063_PI421866198	NM_001107274	NA	NA	NA	NA
14521	5	11	2	4	-2,031	-1,022	0,007909156	NM_001099515.1	CUST_12939_PI421866198	NM_001099515	Vom2r49	vomeronasal 2 receptor, 49	ENSRNOG00000050003	688392
33995	2	5	0	2	-2,034	-1,024	0,002009259	NM_001000432.1	CUST_223_PI421866198	NM_001000432	Olr1151	olfactory receptor 1151	ENSRNOG00000055639	300411
34199	38	76	13	21	-2,035	-1,025	0,001878776	Z93356	Z93356_P1	Z93356	NA	lncRNA	NA	NA
38075	14	27	7	3	-2,037	-1,026	0,004396488	NM_001105788.1	CUST_13065_PI421866198	NM_001105788	NA	NA	NA	NA
7507	4	7	1	1	-2,039	-1,028	0,002228764	MRAK045737	MRAK045737_P1	MRAK045737	NA	lncRNA	NA	NA
40899	4	8	1	1	-2,04	-1,028	0,000243737	NM_001000043.1	CUST_2655_PI421866198	NM_001000043	Olr1567	olfactory receptor 1567	ENSRNOG00000046736	287970
37469	6	12	4	4	-2,042	-1,03	0,007338854	NM_001000319.1	CUST_814_PI421866198	NM_001000319	Olr528	olfactory receptor 528	ENSRNOG00000061596	295776
39200	5	10	2	4	-2,045	-1,032	0,006891605	NM_001130582.1	CUST_4058_PI421866198	NM_001130582	Xpnpep3	X-prolyl aminopeptidase 3	ENSRNOG00000019196	685823
8725	118	230	53	69	-2,049	-1,035	0,005507336	AY562215	AY562215_P1	AY562215	NA	lncRNA	NA	NA
40401	7	13	4	3	-2,05	-1,036	0,009671759	NM_001006597.1	CUST_10110_PI421866198	NM_001006597	Olr990	olfactory receptor 990	ENSRNOG00000049555	288801
12302	5	11	2	4	-2,052	-1,037	0,00586046	XR_007620	XR_007620_P1	XR_007620	NA	lncRNA	NA	NA
45181	9	19	3	9	-2,052	-1,037	0,006887374	NM_170666.1	CUST_9832_PI421866198	NM_170666	Rims4	regulating synaptic membrane exocytosis 4	ENSRNOG00000010868	266976
22071	3	6	2	1	-2,058	-1,041	0,010024115	uc.190-	uc.190-_P1	uc-	NA	NA	NA	NA
37756	135	286	46	123	-2,059	-1,042	0,003670306	NM_131909.1	CUST_9764_PI421866198	NM_131909	Hrh4	histamine receptor H4	ENSRNOG00000016887	170704
30626	44	84	18	13	-2,069	-1,049	0,005567018	MRAK147844	MRAK147844_P1	MRAK147844	NA	lncRNA	NA	NA
15033	3	5	1	2	-2,076	-1,054	0,000546706	NM_001000809.1	CUST_13117_PI421866198	NM_001000809	Olr1245	olfactory receptor 1245	ENSRNOG00000052884	405095
29105	7	14	2	4	-2,085	-1,06	0,001745329	MRuc009ngj	MRuc009ngj_P1	MRuc009ngj	NA	lncRNA	NA	NA
2475	3	7	1	2	-2,087	-1,061	0,00116633	MRAK083678	MRAK083678_P1	MRAK083678	NA	NA	NA	NA
43258	512	938	455	424	-2,091	-1,064	0,036469122	NM_001109493.1	CUST_1755_PI421866198	NM_001109493	Lce1l	late cornified envelope 1L	ENSRNOG00000042105	686125
9396	10	21	2	5	-2,093	-1,066	0,000210529	AY564232	AY564232_P1	AY564232	NA	lncRNA	NA	NA
28816	11	23	4	9	-2,094	-1,066	0,006516742	MRAK020046	MRAK020046_P1	MRAK020046	NA	lncRNA	NA	NA
38916	4	9	2	2	-2,098	-1,069	0,003166258	NM_001127344.1	CUST_6922_PI421866198	NM_001127344	Tp63	tumor protein p63	ENSRNOG00000001924	246334
41400	57	100	43	34	-2,099	-1,07	0,045177025	uc.463+	uc.463+_P1	uc+	NA	lncRNA	NA	NA
34723	638	1404	359	993	-2,1	-1,07	0,046072455	NM_001001096.1	CUST_14684_PI421866198	NM_001001096	Olr1400	NA	NA	NA
41069	28	54	14	8	-2,105	-1,074	0,003411882	NM_001004278.2	CUST_3653_PI421866198	NM_001004278	Tsga10ip	testis specific 10 interacting protein	ENSRNOG00000027564	361707
17165	5	10	2	1	-2,117	-1,082	0,005724221	NM_023963.1	CUST_11877_PI421866198	NM_023963	Cdx2	caudal type homeo box 2	ENSRNOG00000032759	66019
13097	94	171	66	48	-2,12	-1,084	0,025411779	XR_008328	XR_008328_P1	XR_008328	NA	lncRNA	NA	NA
42847	14	31	4	11	-2,129	-1,09	0,001667642	NM_001025278.1	CUST_13193_PI421866198	NM_001025278	Pus10	pseudouridylate synthase 10	ENSRNOG00000054151	305583
38911	12	25	6	10	-2,133	-1,093	0,005519561	uc.295+	uc.295+_P1	uc+	NA	lncRNA	NA	NA
33096	37	82	10	29	-2,142	-1,099	0,00215898	NM_001037852.1	CUST_9047_PI421866198	NM_001037852	Spag11b	NA	NA	NA
28079	2	6	0	3	-2,143	-1,1	0,007841874	NM_001080756.1	CUST_4023_PI421866198	NM_001080756	Car12	carbonic anhydrase 12	ENSRNOG00000017766	363085
42868	72	149	28	41	-2,144	-1,1	0,001775975	NM_001079707.1	CUST_14458_PI421866198	NM_001079707	Gtsf1	gametocyte specific factor 1	ENSRNOG00000036831	315347
35648	34	66	16	15	-2,146	-1,102	0,016604111	uc.441-	uc.441-_P1	uc-	NA	lncRNA	NA	NA
26870	4	11	2	10	-2,151	-1,105	0,044789712	XR_006836	XR_006836_P1	XR_006836	NA	lncRNA	NA	NA
10965	10	25	2	15	-2,153	-1,107	0,004004928	XR_007026	XR_007026_P1	XR_007026	NA	lncRNA	NA	NA
13163	5	11	3	4	-2,154	-1,107	0,023066042	MRAK051145	MRAK051145_P1	MRAK051145	NA	lncRNA	NA	NA
15460	6	12	2	3	-2,158	-1,11	0,00600134	NM_001108690.1	CUST_4080_PI421866198	NM_001108690	Emc1	ER membrane protein complex subunit 1	ENSRNOG00000018097	362643
34358	5	12	2	5	-2,158	-1,11	0,007212312	NM_001001372.1	CUST_12624_PI421866198	NM_001001372	Olr219	olfactory receptor 219	ENSRNOG00000047623	293365
38012	26	44	26	13	-2,158	-1,11	0,038993858	NM_001000674.1	CUST_14438_PI421866198	NM_001000674	Olr529	olfactory receptor 529	ENSRNOG00000054730	404877
3335	3	7	1	3	-2,165	-1,114	0,001227741	MRAK138769	MRAK138769_P1	MRAK138769	NA	NA	NA	NA
23449	10	19	5	9	-2,168	-1,117	0,046904347	MRAK052988	MRAK052988_P1	MRAK052988	NA	lncRNA	NA	NA
6889	5	11	1	4	-2,175	-1,121	0,000341412	MRAK040850	MRAK040850_P1	MRAK040850	NA	NA	NA	NA
43803	27	48	15	8	-2,177	-1,122	0,044758497	NM_001000352.1	CUST_707_PI421866198	NM_001000352	Olr674	NA	NA	NA
40148	5	9	3	4	-2,178	-1,123	0,018167368	NM_001109168.1	CUST_3824_PI421866198	NM_001109168	Calhm1	calcium homeostasis modulator 1	ENSRNOG00000030682	499367
37234	77	168	41	94	-2,186	-1,128	0,025919024	uc.456-	uc.456-_P1	uc-	NA	lncRNA	NA	NA
14290	6	14	2	3	-2,188	-1,13	0,000630854	XR_006929	XR_006929_P1	XR_006929	NA	lncRNA	NA	NA
13723	144	314	87	166	-2,192	-1,132	0,021797244	XR_006644	XR_006644_P1	XR_006644	NA	lncRNA	NA	NA
18586	3	6	1	4	-2,198	-1,136	0,026141548	NM_001000586.1	CUST_14975_PI421866198	NM_001000586	Olr879	olfactory receptor 879	ENSRNOG00000043438	366793
24106	5	10	2	1	-2,208	-1,142	0,003582548	NM_001000375.1	CUST_639_PI421866198	NM_001000375	Olr781	olfactory receptor 781	ENSRNOG00000048229	296035
42358	8	18	4	9	-2,209	-1,144	0,010242952	NM_001000207.1	CUST_1402_PI421866198	NM_001000207	Olr233	olfactory receptor 233	ENSRNOG00000046869	293377
42360	125	250	64	82	-2,215	-1,147	0,01506169	NM_001000958.1	CUST_12374_PI421866198	NM_001000958	Olr1445	olfactory receptor 1445	ENSRNOG00000046040	405307
39126	25	45	19	4	-2,215	-1,147	0,026034907	NM_001008959.1	CUST_1565_PI421866198	NM_001008959	NA	NA	NA	NA
40476	19	37	10	8	-2,225	-1,154	0,012883621	NM_022611.1	CUST_1912_PI421866198	NM_022611	Il12b	interleukin 12B	ENSRNOG00000004380	64546
39807	34	67	28	29	-2,225	-1,154	0,02492807	NM_001106725.1	CUST_9363_PI421866198	NM_001106725	Agr2	anterior gradient 2, protein disulphide isomerase family member	ENSRNOG00000005023	298961
4543	17	38	7	15	-2,226	-1,154	0,012067579	MRAK080245	MRAK080245_P1	MRAK080245	NA	lncRNA	NA	NA
38174	291	691	120	435	-2,235	-1,16	0,016732871	NM_017108.1	CUST_12854_PI421866198	NM_017108	Kcnh3	potassium voltage-gated channel subfamily H member 3	ENSRNOG00000057315	27150
11979	7	14	4	6	-2,244	-1,166	0,031392261	NM_012669.1	CUST_10267_PI421866198	NM_012669	Hnf1a	HNF1 homeobox A	ENSRNOG00000001183	24817
38777	74	146	45	12	-2,246	-1,167	0,003939255	NM_001134799.1	CUST_5182_PI421866198	NM_001134799	Tmem95	transmembrane protein 95	ENSRNOG00000043507	691982
14860	6	12	4	3	-2,246	-1,167	0,013352789	uc.290+	uc.290+_P1	uc+	NA	lncRNA	NA	NA
43530	24	51	13	23	-2,246	-1,168	0,028425316	uc.398-	uc.398-_P1	uc-	NA	lncRNA	NA	NA
43118	48	98	28	17	-2,252	-1,171	0,005226955	NM_001000897.1	CUST_12938_PI421866198	NM_001000897	Olr837	olfactory receptor 837	ENSRNOG00000033505	405207
40719	69	143	43	73	-2,258	-1,175	0,029394995	NM_133296.1	CUST_7757_PI421866198	NM_133296	Slc6a20	NA	NA	NA
38460	46	104	20	47	-2,264	-1,179	0,006776484	NM_001013993.1	CUST_8669_PI421866198	NM_001013993	NA	NA	NA	NA
42166	5	10	2	4	-2,268	-1,181	0,007997343	uc.381-	uc.381-_P1	uc-	NA	NA	NA	NA
33889	10	21	5	7	-2,271	-1,183	0,007555061	MRAK150047	MRAK150047_P1	MRAK150047	NA	lncRNA	NA	NA
42444	23	61	9	45	-2,283	-1,191	0,017859135	NM_001001115.1	CUST_14236_PI421866198	NM_001001115	Olr1707	NA	NA	NA
36027	6	11	3	4	-2,284	-1,192	0,019754704	NM_001109371.1	CUST_2579_PI421866198	NM_001109371	Awat1	acyl-CoA wax alcohol acyltransferase 1	ENSRNOG00000026231	679520
38325	5	10	3	1	-2,292	-1,197	0,001013543	MRAK144529	MRAK144529_P1	MRAK144529	NA	NA	NA	NA
43887	40	84	19	28	-2,297	-1,2	0,008070204	uc.57+	uc.57+_P1	uc+	NA	lncRNA	NA	NA
38087	12	25	7	9	-2,3	-1,202	0,011904479	BC100252	BC100252_P1	BC100252	NA	lncRNA	NA	NA
37915	10	18	8	3	-2,3	-1,201	0,026134527	NM_001037549.1	CUST_11102_PI421866198	NM_001037549	Defb5	defensin beta 5	ENSRNOG00000038126	641623
37412	31	72	15	33	-2,302	-1,203	0,010739752	NM_001106987.1	CUST_8325_PI421866198	NM_001106987	Rnf151	ring finger protein 151	ENSRNOG00000014127	302977
34791	101	244	27	107	-2,304	-1,204	0,002113102	NM_001000003.1	CUST_2795_PI421866198	NM_001000003	Olr1401	olfactory receptor 1401	ENSRNOG00000033524	287247
37841	34	70	21	14	-2,304	-1,204	0,007043359	NM_001106866.1	CUST_8871_PI421866198	NM_001106866	Exog	exo/endonuclease G	ENSRNOG00000014801	301062
36312	5	12	1	5	-2,31	-1,208	0,000488222	NM_012493.1	CUST_11540_PI421866198	NM_012493	Afp	alpha-fetoprotein	ENSRNOG00000002889	24177
36139	7	17	3	4	-2,321	-1,215	0,00073506	uc.78+	uc.78+_P1	uc+	NA	lncRNA	NA	NA
37460	5	11	2	4	-2,334	-1,223	0,002049799	NM_182667.1	CUST_9753_PI421866198	NM_182667	Myocd	myocardin	ENSRNOG00000003669	246297
18574	26	56	13	15	-2,375	-1,248	0,006396185	MRAK007090	MRAK007090_P1	MRAK007090	NA	lncRNA	NA	NA
39016	5	12	2	6	-2,376	-1,249	0,014181422	NM_001000466.1	CUST_145_PI421866198	NM_001000466	Olr1309	olfactory receptor 1309	ENSRNOG00000050335	300610
34264	151	427	50	311	-2,385	-1,254	0,017862928	NM_001047953.1	CUST_177_PI421866198	NM_001047953	LOC499843	LRRGT00091	ENSRNOG00000032177	499843
42437	10	21	5	4	-2,389	-1,257	0,005675919	NM_001107719.1	CUST_7026_PI421866198	NM_001107719	Sass6	SAS-6 centriolar assembly protein	ENSRNOG00000015211	310807
41157	33	79	15	34	-2,391	-1,257	0,001407152	uc.246+	uc.246+_P1	uc+	NA	lncRNA	NA	NA
17409	4	10	2	5	-2,397	-1,261	0,013321008	NM_001137670.1	CUST_10924_PI421866198	NM_001137670	Sep-14	NA	NA	NA
44407	139	374	28	234	-2,402	-1,264	0,002313679	NM_001013243.1	CUST_13265_PI421866198	NM_001013243	Slc30a3	solute carrier family 30 member 3	ENSRNOG00000006204	366568
35688	4	11	3	11	-2,414	-1,271	0,04518691	NM_001106017.1	CUST_14395_PI421866198	NM_001106017	Myl7	myosin light chain 7	ENSRNOG00000014409	289759
40813	16	33	9	5	-2,432	-1,282	0,010893602	NM_001109587.1	CUST_1172_PI421866198	NM_001109587	Pla2g2f	phospholipase A2, group IIF	ENSRNOG00000016798	690388
44391	120	288	61	149	-2,438	-1,286	0,008100199	NM_020087.2	CUST_11603_PI421866198	NM_020087	Notch3	notch 3	ENSRNOG00000004346	56761
38188	17	33	13	8	-2,44	-1,287	0,026220269	uc.322+	uc.322+_P1	uc+	NA	lncRNA	NA	NA
40046	74	150	49	37	-2,446	-1,29	0,02212556	NM_001001116.1	CUST_14234_PI421866198	NM_001001116	Olr1720	NA	NA	NA
40140	10	23	5	8	-2,46	-1,299	0,00377402	NM_001000296.1	CUST_1124_PI421866198	NM_001000296	Olr464	olfactory receptor 464	ENSRNOG00000051431	295734
41237	5	11	2	4	-2,475	-1,307	0,002351374	NM_001008954.1	CUST_1570_PI421866198	NM_001008954	NA	NA	NA	NA
29154	6	20	1	21	-2,478	-1,309	0,019200979	NM_212459.1	CUST_12761_PI421866198	NM_212459	Arl9	ADP-ribosylation factor like GTPase 9	ENSRNOG00000029628	289565
40633	23	53	12	14	-2,484	-1,313	0,003677754	NM_022667.1	CUST_1714_PI421866198	NM_022667	Slco2a1	solute carrier organic anion transporter family, member 2a1	ENSRNOG00000009005	24546
37347	3	9	1	6	-2,485	-1,313	0,015333771	NM_001000801.1	CUST_13131_PI421866198	NM_001000801	Olr1274	olfactory receptor 1274	ENSRNOG00000060027	100910402
39638	7	15	6	8	-2,499	-1,322	0,025926396	NM_001000196.1	CUST_1706_PI421866198	NM_001000196	Olr211	olfactory receptor 211	ENSRNOG00000048223	293358
26334	3	8	2	4	-2,503	-1,324	0,016530465	NM_001106516.1	CUST_10867_PI421866198	NM_001106516	Ankef1	ankyrin repeat and EF-hand domain containing 1	ENSRNOG00000005792	296184
43540	29	81	11	46	-2,538	-1,344	0,012556017	NM_001011948.1	CUST_393_PI421866198	NM_001011948	Slc7a12	solute carrier family 7 (cationic amino acid transporter, y+ system), member 12	ENSRNOG00000033830	294881
41412	9	21	4	4	-2,544	-1,347	0,001528913	NM_001030035.1	CUST_6849_PI421866198	NM_001030035	NA	NA	NA	NA
11076	31	69	21	27	-2,551	-1,351	0,010105087	MRAK044797	MRAK044797_P1	MRAK044797	NA	lncRNA	NA	NA
24820	5	11	2	4	-2,555	-1,353	0,008330871	NM_001099457.1	CUST_13561_PI421866198	NM_001099457	Vom2r2	NA	NA	NA
37662	50	122	24	45	-2,559	-1,356	0,003585188	NM_001000788.1	CUST_13689_PI421866198	NM_001000788	Olr1388	olfactory receptor 1388	ENSRNOG00000046425	405066
43286	45	124	21	83	-2,56	-1,356	0,011362764	NM_001000167.1	CUST_1802_PI421866198	NM_001000167	Olr153	olfactory receptor 153	ENSRNOG00000017214	293301
43853	298	645	262	240	-2,568	-1,36	0,0089218	NM_001013232.1	CUST_13306_PI421866198	NM_001013232	Rnase1l1	ribonuclease, RNase A family, 1-like 1 (pancreatic)	ENSRNOG00000025615	364303
43953	4	12	2	6	-2,57	-1,362	0,009733514	NM_001001090.1	CUST_14690_PI421866198	NM_001001090	Olr1392	olfactory receptor 1392	ENSRNOG00000046072	405984
35899	15	33	11	8	-2,573	-1,363	0,01619926	NM_001047948.1	CUST_193_PI421866198	NM_001047948	LOC499584	LRRGT00202	ENSRNOG00000033028	499584
39029	4	14	2	12	-2,575	-1,365	0,035083717	NM_001000512.1	CUST_15075_PI421866198	NM_001000512	Olr601	olfactory receptor 601	ENSRNOG00000005863	311175
39022	4	11	2	5	-2,578	-1,366	0,006995425	NM_001106595.1	CUST_10667_PI421866198	NM_001106595	Smyd1	SET and MYND domain containing 1	ENSRNOG00000006776	297333
44059	33	72	19	22	-2,578	-1,366	0,022553213	NM_001000682.1	CUST_14410_PI421866198	NM_001000682	Olr486	olfactory receptor 486	ENSRNOG00000029884	404887
23459	4	9	2	2	-2,609	-1,384	0,000702862	uc.24-	uc.24-_P1	uc-	NA	NA	NA	NA
37464	2	8	0	6	-2,619	-1,389	0,008430256	NM_134380.1	CUST_4195_PI421866198	NM_134380	Ust5r	integral membrane transport protein UST5r	ENSRNOG00000061890	171398
36986	11	24	9	5	-2,623	-1,391	0,00961598	NM_031133.1	CUST_14803_PI421866198	NM_031133	Thpo	thrombopoietin	ENSRNOG00000050172	81811
34695	36	98	13	45	-2,627	-1,393	0,002433419	NM_023992.1	CUST_11811_PI421866198	NM_023992	Kiss1r	KISS1 receptor	ENSRNOG00000011954	78976
23236	43	142	11	105	-2,639	-1,4	0,013202688	XR_006594	XR_006594_P1	XR_006594	NA	lncRNA	NA	NA
9507	4	10	2	3	-2,647	-1,405	0,000315041	MRAK040942	MRAK040942_P1	MRAK040942	NA	NA	NA	NA
39380	24	48	22	6	-2,663	-1,413	0,011382497	NM_001025150.1	CUST_13961_PI421866198	NM_001025150	Tas2r144	taste receptor, type 2, member 144	ENSRNOG00000028130	500101
45077	8	22	4	10	-2,665	-1,414	0,004064999	uc.450-	uc.450-_P1	uc-	NA	lncRNA	NA	NA
36137	19	50	8	16	-2,686	-1,425	0,001887319	NM_001001350.1	CUST_12676_PI421866198	NM_001001350	Olr1512	olfactory receptor 1512	ENSRNOG00000061506	287511
36918	18	37	18	7	-2,71	-1,438	0,013546535	uc.208+	uc.208+_P1	uc+	NA	lncRNA	NA	NA
23977	30	89	4	41	-2,716	-1,441	0,001076955	XR_008140	XR_008140_P1	XR_008140	NA	lncRNA	NA	NA
36305	16	40	9	14	-2,722	-1,445	0,003742921	uc.312-	uc.312-_P1	uc-	NA	lncRNA	NA	NA
23313	3	8	0	5	-2,724	-1,446	0,000628586	XR_006363	XR_006363_P1	XR_006363	NA	lncRNA	NA	NA
35556	111	254	94	83	-2,75	-1,46	0,008775605	NM_001000585.1	CUST_14976_PI421866198	NM_001000585	Olr859	olfactory receptor 859	ENSRNOG00000047595	366458
39956	56	126	40	56	-2,752	-1,46	0,025857404	NM_147165.1	CUST_1837_PI421866198	NM_147165	Gpx6	glutathione peroxidase 6	ENSRNOG00000060749	NA
35541	7	17	7	4	-2,782	-1,476	0,005993912	uc.476+	uc.476+_P1	uc+	NA	NA	NA	NA
43622	7	16	5	3	-2,791	-1,481	0,00368943	NM_013076.2	CUST_10720_PI421866198	NM_013076	Lep	leptin	ENSRNOG00000045797	25608
8259	130	298	129	107	-2,798	-1,484	0,008403087	XR_008529	XR_008529_P1	XR_008529	NA	lncRNA	NA	NA
40976	8	16	7	5	-2,814	-1,493	0,015731815	uc.363+	uc.363+_P1	uc+	NA	NA	NA	NA
41578	13	32	6	6	-2,827	-1,499	0,008001536	NM_001109600.1	CUST_892_PI421866198	NM_001109600	Adam30	ADAM metallopeptidase domain 30	ENSRNOG00000019044	690547
44646	51	132	29	23	-2,867	-1,519	0,000824857	NM_001109439.1	CUST_1991_PI421866198	NM_001109439	LOC681325	hypothetical protein LOC681325	ENSRNOG00000023005	681325
37137	5	13	3	2	-2,879	-1,525	0,001519649	uc.125-	uc.125-_P1	uc-	NA	NA	NA	NA
41577	11	23	9	3	-2,892	-1,532	0,017339069	uc.199+	uc.199+_P1	uc+	NA	lncRNA	NA	NA
43698	18	47	12	9	-2,928	-1,55	0,000824963	NM_001107528.1	CUST_7831_PI421866198	NM_001107528	Hddc3	HD domain containing 3	ENSRNOG00000012236	308758
18097	3	14	1	19	-2,931	-1,551	0,042858615	NM_001013903.1	CUST_8829_PI421866198	NM_001013903	Tmem171	transmembrane protein 171	ENSRNOG00000015449	293634
39957	14	35	10	11	-2,982	-1,576	0,007398643	NM_022945.1	CUST_134_PI421866198	NM_022945	Lcn1	lipocalin 1	ENSRNOG00000033761	65039
44471	9	26	5	13	-2,984	-1,577	0,005227254	uc.166-	uc.166-_P1	uc-	NA	NA	NA	NA
41410	19	49	13	1	-2,991	-1,58	0,001014099	NM_001000225.1	CUST_1345_PI421866198	NM_001000225	Olr281	olfactory receptor 281	ENSRNOG00000049832	293425
44548	122	223	186	139	-3,008	-1,589	0,049304286	NM_001001014.1	CUST_14902_PI421866198	NM_001001014	Olr402	olfactory receptor 402	ENSRNOG00000046228	405388
34887	43	120	26	67	-3,02	-1,595	0,012637595	MRuc008bws	MRuc008bws_P1	MRuc008bws	NA	lncRNA	NA	NA
36811	8	21	9	10	-3,048	-1,608	0,023608548	uc.104-	uc.104-_P1	uc-	NA	lncRNA	NA	NA
40729	8	30	4	34	-3,074	-1,62	0,013943443	NM_001025149.1	CUST_13972_PI421866198	NM_001025149	Tas2r137	taste receptor, type 2, member 137	ENSRNOG00000030413	500089
39228	294	1094	111	1058	-3,084	-1,625	0,005775137	NM_017330.2	CUST_11356_PI421866198	NM_017330	Prf1	perforin 1	ENSRNOG00000000562	NA
35221	27	61	23	11	-3,114	-1,639	0,016498818	NM_001000912.1	CUST_12470_PI421866198	NM_001000912	Olr1591	olfactory receptor 1591	ENSRNOG00000032352	NA
25072	3	15	1	19	-3,125	-1,644	0,027865018	NM_001001027.1	CUST_14858_PI421866198	NM_001001027	Olr129	olfactory receptor 129	ENSRNOG00000025163	405912
39443	8	25	4	6	-3,13	-1,646	0,000222828	uc.98-	uc.98-_P1	uc-	NA	NA	NA	NA
7292	24	53	30	24	-3,15	-1,655	0,018662376	XR_009545	XR_009545_P1	XR_009545	NA	lncRNA	NA	NA
3998	16	38	19	20	-3,202	-1,679	0,011548447	XR_005967	XR_005967_P1	XR_005967	NA	lncRNA	NA	NA
34696	15	47	9	26	-3,203	-1,679	0,0064361	NM_001000148.1	CUST_1896_PI421866198	NM_001000148	Olr107	olfactory receptor 107	ENSRNOG00000015901	293246
40728	11	27	11	4	-3,205	-1,681	0,0075263	NM_001106959.1	CUST_8352_PI421866198	NM_001106959	NA	NA	NA	NA
38006	17	47	11	13	-3,21	-1,682	0,002098556	NM_001013915.1	CUST_8815_PI421866198	NM_001013915	Tas2r134	taste receptor, type 2, member 134	ENSRNOG00000027510	295589
38683	7	19	6	6	-3,224	-1,689	0,004272655	NM_176079.1	CUST_9229_PI421866198	NM_176079	Myod1	myogenic differentiation 1	ENSRNOG00000011306	337868
41312	9	23	6	9	-3,241	-1,696	0,008976581	NM_001000346.1	CUST_737_PI421866198	NM_001000346	Olr663	olfactory receptor 663	ENSRNOG00000047879	295869
43022	9	24	7	4	-3,297	-1,721	0,002526279	NM_012672.2	CUST_10250_PI421866198	NM_012672	Thrb	thyroid hormone receptor beta	ENSRNOG00000006649	24831
44640	5	15	3	3	-3,455	-1,789	0,000482837	uc.392-	uc.392-_P1	uc-	NA	NA	NA	NA
41495	5	17	3	6	-3,494	-1,805	0,000384089	NM_001008929.1	CUST_1649_PI421866198	NM_001008929	NA	NA	NA	NA
39867	8	26	5	6	-3,502	-1,808	0,00072623	NM_001047931.1	CUST_224_PI421866198	NM_001047931	LOC498460	LRRGT00055	ENSRNOG00000028821	498460
41314	25	66	22	40	-3,618	-1,855	0,031313433	NM_001080939.1	CUST_2901_PI421866198	NM_001080939	Tas2r109	taste receptor, type 2, member 109	ENSRNOG00000032724	690572

38176	6	19	3	7	-3,633	-1,861	0,000472271	NM_001164826.1	CUST_8296_PI421866198	NM_001164826	RT1-Db2	RT1 class II, locus Db2	ENSRNOG00000030431	24981
44381	9	25	7	3	-3,641	-1,864	0,004881053	NM_001130497.1	CUST_4777_PI421866198	NM_001130497	Pnpla5	patatin-like phospholipase domain containing 5	ENSRNOG00000022296	300108
35607	5	20	3	13	-3,665	-1,874	0,001626491	MRAK078136	MRAK078136_P1	MRAK078136	NA	lncRNA	NA	NA
16902	3	17	1	21	-3,73	-1,899	0,018555276	uc.163+	uc.163+_P1	uc+	NA	NA	NA	NA
41319	14	37	13	10	-3,76	-1,911	0,0102028	NM_001012084.1	CUST_2352_PI421866198	NM_001012084	Adh6	NA	NA	NA
37492	19	60	14	19	-3,778	-1,918	0,002483192	NM_001003979.1	CUST_2839_PI421866198	NM_001003979	Tmprss11c	transmembrane protease, serine 11C	ENSRNOG00000033910	408213
36560	10	30	9	5	-3,867	-1,951	0,002329635	NM_001000338.1	CUST_759_PI421866198	NM_001000338	Olr619	olfactory receptor 619	ENSRNOG00000021473	295843
43356	12	40	10	7	-4,153	-2,054	0,000942008	NM_022673.2	CUST_1677_PI421866198	NM_022673	Mecp2	methyl CpG binding protein 2	ENSRNOG00000056659	29386
42598	6	24	3	7	-4,166	-2,059	0,00012381	NM_001017480.1	CUST_14566_PI421866198	NM_001017480	Hoxb7	homeo box B7	ENSRNOG00000007611	497985
38934	27	96	24	39	-4,623	-2,209	0,003554041	NM_052808.1	CUST_4978_PI421866198	NM_052808	Bpifa2	BPI fold containing family A, member 2	ENSRNOG00000013540	50585
38765	127	470	119	306	-4,638	-2,213	0,005567588	NM_001000575.1	CUST_14996_PI421866198	NM_001000575	Olr741	olfactory receptor 741	ENSRNOG00000053815	366120
37233	8	30	7	16	-4,685	-2,228	0,005334988	NM_012689.1	CUST_10220_PI421866198	NM_012689	Esr1	estrogen receptor 1	ENSRNOG00000019358	24890
43878	12	44	11	13	-4,714	-2,237	0,001673099	NM_001000307.1	CUST_844_PI421866198	NM_001000307	Olr485	olfactory receptor 485	ENSRNOG00000009747	295751
38741	3	21	1	21	-4,797	-2,262	0,004343828	NM_001001355.1	CUST_12671_PI421866198	NM_001001355	Olr905	olfactory receptor 905	ENSRNOG00000057325	288875
44809	8	30	6	8	-4,858	-2,28	0,000573719	NM_001109459.1	CUST_1913_PI421866198	NM_001109459	LOC685171	similar to protein disulfide isomerase-associated 6	ENSRNOG00000058543	685171
40038	8	28	7	8	-4,88	-2,287	0,001235846	uc.400-	uc.400-_P1	uc-	NA	lncRNA	NA	NA
43786	68	248	60	63	-5,144	-2,363	0,002342316	NM_001109285.1	CUST_3125_PI421866198	NM_001109285	C2cd4c	C2 calcium-dependent domain containing 4C	ENSRNOG00000008026	500798
43442	7	28	7	8	-5,396	-2,432	0,000394893	uc.225+	uc.225+_P1	uc+	NA	lncRNA	NA	NA
40720	31	111	33	41	-5,65	-2,498	0,004147011	NM_001000692.1	CUST_14388_PI421866198	NM_001000692	Olr25	olfactory receptor 25	ENSRNOG00000046609	404897
41996	14	54	15	11	-6,14	-2,618	0,001417661	uc.47-	uc.47-_P1	uc-	NA	lncRNA	NA	NA
43609	14	49	20	6	-6,599	-2,722	0,00182715	NM_001000394.1	CUST_552_PI421866198	NM_001000394	Olr428	olfactory receptor 428	ENSRNOG00000030460	296689
36293	27	111	36	19	-8,031	-3,006	0,002058348	XR_008902	XR_008902_P1	XR_008902	NA	lncRNA	NA	NA
44472	15	72	20	9	-9,201	-3,202	0,001238324	NM_019326.1	CUST_5367_PI421866198	NM_019326	Neurod2	neuronal differentiation 2	ENSRNOG00000028417	NA
