# Supplementary material for: Transcriptional Alterations in the Trigeminal Ganglia, Nucleus and Peripheral Blood Mononuclear Cells in a Rat Orofacial Pain Model
Source: Front Mol Neurosci. 2018 Jun 26;11:219. doi: 10.3389/fnmol.2018.00219 (PMC6028693; doi:10.3389/fnmol.2018.00219)
Supplement: Supplementary file 3 [file Image_1.PDF]

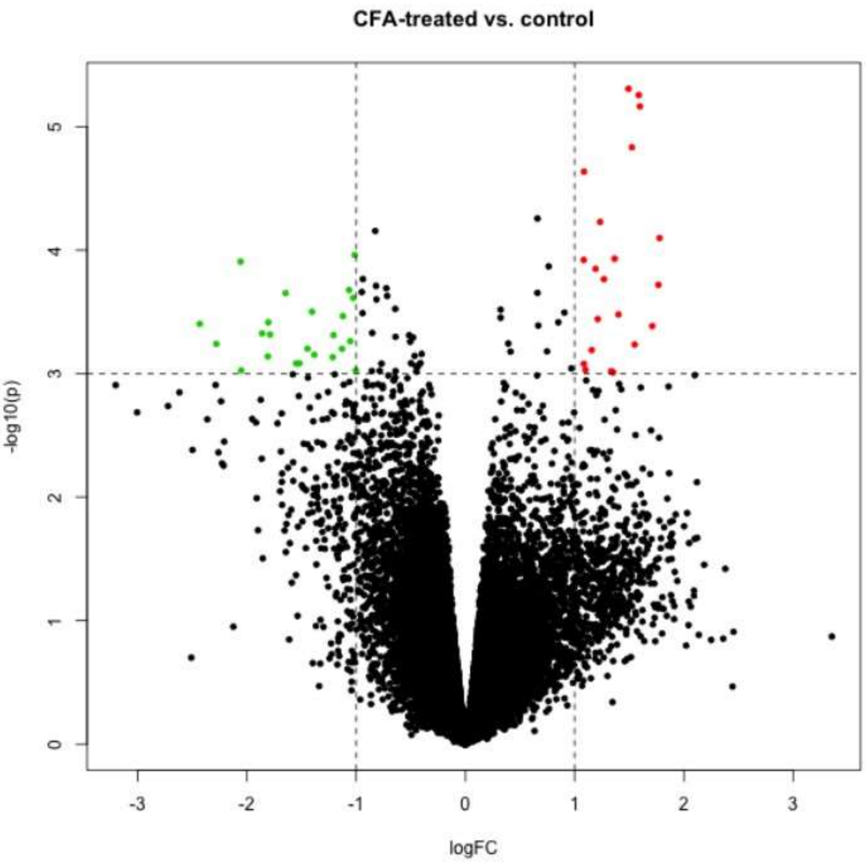

Supplementary Figure 1.

Results of the comparison as Volcano plot. The log10 of the p-values is on the y-axis and the logFC calculated for the CFA group vs. contralateral sample group is on the x-axis. In this plot it can be seen how the reliability values of the measurement features behave in relation to the fold change. The thresholds used in the filtering are marked in the plot with dashed lines, up-regulated genes are coloured red and down-regulated green.
