# Supplementary material for: Transcriptional Alterations in the Trigeminal Ganglia, Nucleus and Peripheral Blood Mononuclear Cells in a Rat Orofacial Pain Model
Source: Front Mol Neurosci. 2018 Jun 26;11:219. doi: 10.3389/fnmol.2018.00219 (PMC6028693; doi:10.3389/fnmol.2018.00219)
Supplement: Supplementary file 4 [file Image_2.PDF]

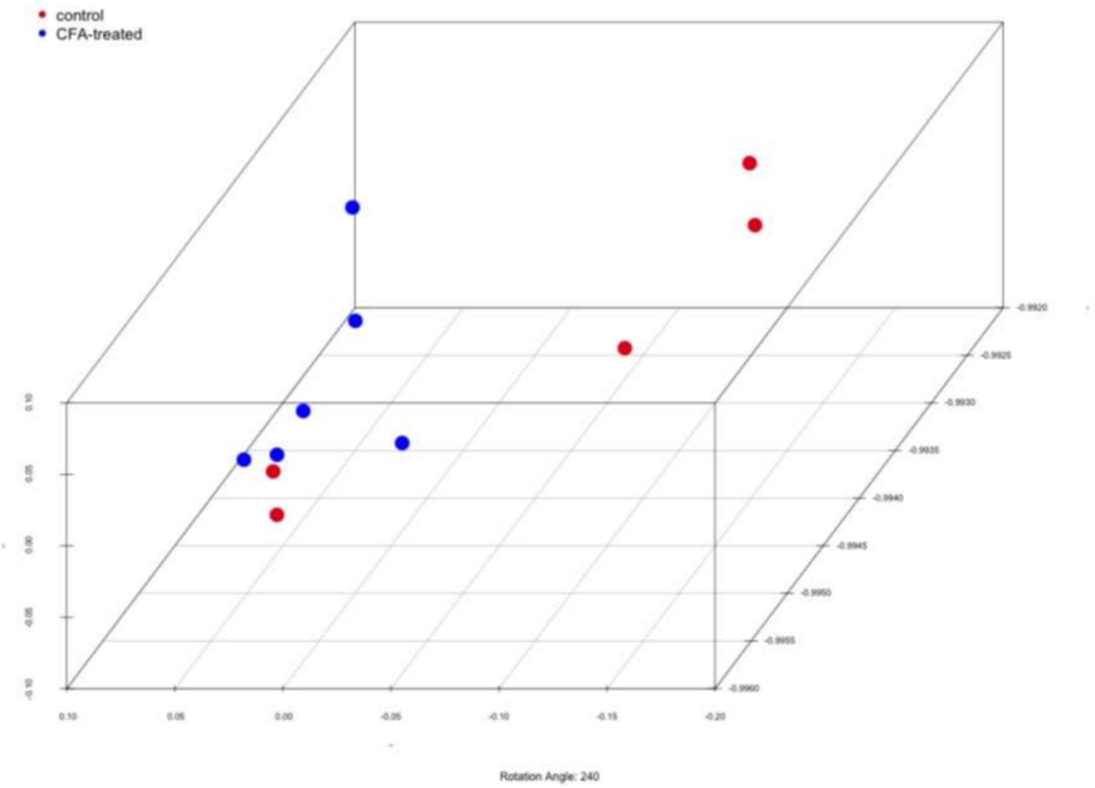

Supplementary Figure 2.

Principal Component Analysis (PCA) plot for all replicates. The sample relations studied by the means of PCA and visualized in three-dimensional space. PCA is an ordination technique complementary to clustering. Ordination orders objects so that similar objects are placed near each other and dissimilar objects are placed further from each other. In PCA analysis the sample relationships can be visualized in three-dimensional space.
